# Antibacterial Activity and Multi-Targeted Mechanism of Action of Suberanilic Acid Isolated from *Pestalotiopsis trachycarpicola* DCL44: An Endophytic Fungi from *Ageratina adenophora*

**DOI:** 10.3390/molecules29174205

**Published:** 2024-09-04

**Authors:** Juan Wen, Samuel Kumi Okyere, Shu Wang, Jianchen Wang, Ruya Huang, Ziyao Tang, Xiaoxuan Wang, Chenyang Shao, Yanchun Hu

**Affiliations:** 1 Key Laboratory of Animal Disease and Human Health of Sichuan Province, Sichuan Agricultural University, Chengdu 611130, China; juanwen881010@163.com (J.W.); samuel20okyere@gmail.com (S.K.O.); shuw0326@163.com (S.W.); wangjianchen01@163.com (J.W.); m18064929110@163.com (R.H.); 2022203063@stu.sicau.edu.cn (Z.T.); w9775121@163.com (X.W.); shaocy98@163.com (C.S.); 2 College of Animal Science, Xichang University, Xichang 615013, China; 3 Department of Pharmaceutical Sciences, School of Medicine, Wayne State University, Detroit, MI 48201, USA

**Keywords:** methicillin-resistant *Staphylococcus aureus*, suberanilic acid, antibacterial mechanism, proteomic analysis, parallel reaction monitoring

## Abstract

Methicillin-resistant *Staphylococcus aureus* (MRSA) is a highly threatening foodborne pathogen capable of causing severe organ and life-threatening diseases. Over the past years, various commercial antibiotics have been used to treat MRSA infections. However, these commercial antibiotics have not yielded efficient results and also cause other side effects; therefore, there is a need for the development of effective alternatives to replace these commercial antibiotics. Suberanilic acid, an amide alkaloid obtained from the endophytic fungus *Pestalotiopsis trachycarpicola* DCL44, has been identified as a significant antimicrobial agent. However, its antibiotic properties on multi-drug-resistant bacteria such as MRSA have not been fully explored. Therefore, to investigate the potential antimicrobial mechanism of suberanilic acid against MRSA, a quantitative proteomics approach using tandem mass tagging (TMT) was used. The results obtained in the study revealed that suberanilic acid targets multiple pathways in MRSA, including disruption of ribosome synthesis, inhibition of membrane translocation for nutrient uptake (ABC transporter system), and causing dysregulation of carbohydrate and amino acid energy metabolism. These results provide new insights into the mechanism of action of suberanilic acid against MRSA and offer technical support and a theoretical basis for the development of novel food antimicrobial agents derived from endophytic fungal origin.

## 1. Introduction

Food safety has received increased international attention, as unsafe food can cause more than 200 different diseases, from diarrhea to cancer. Each year, an estimated 600 million people worldwide (almost 1 in 10) fall ill from eating contaminated food, leading to 420,000 deaths and 33 million disability-adjusted life-years (DALYs) [1]. MRSA is known to cause various clinical infections, including necrotizing pneumonia, endocarditis, prosthetic joints, surgical sites, cardiovascular infections, and hospital-acquired bacteremia. It is also a major contributor to the current outbreaks of community-acquired foodborne diseases [2,3]. In addition to its resistance to methicillin, MRSA is also resistant to β-lactam antibiotics. This bacterium develops resistance to antimicrobial agents through mechanisms such as altering the target sites of antibiotics, producing modified enzymes, reducing membrane permeability, and producing large amounts of aminobenzoic acid [4]. The emergence of MRSA resistance has significant implications for the food chain, food-producing animals, and related foods. Recent studies have shown that the slaughter of MRSA-carrying animals and the handling of food by individuals infected with MRSA contributes to food contamination [5]. These resistant bacteria can be transferred to humans through food sources, posing a significant threat to human health, as food serves as a major reservoir and source of human MRSA infection.

Over the past two decades, microbiologists have focused on endophytic fungi, which are known to secrete bioactive compounds with unique structures that may help prevent the growth of foodborne pathogens such as MRSA. Compared to chemically synthesized antimicrobial agents, compounds derived from endophytic fungi are considered promising candidates for natural food antimicrobial agents due to their high safety profile. These properties support endophyte-fungus-derived compounds as alternative sources to synthetic chemicals [6,7,8]. The results of our preliminary studies have shown that suberanilic acid, an amide alkaloid isolated from the endophytic fungus *Pestalotiopsis trachycarpicola* DCL44 from *Ageratina adenophora*, exhibits antimicrobial activity against MRSA (unpublished data), indicating its potential for the development of novel natural food antimicrobial agents. While the compound may have multiple targets of action against MRSA, its mode of action remains unknown.

In this study, differentially expressed proteins (DEPs) between control and treatment groups were characterized using tandem mass spectrometry tagging (TMT) quantitative proteomics analyses. Bioinformatics was used to reveal their potential functions and the biological processes involved, and some TMT-selected target proteins were validated at the protein level using the parallel reaction monitoring (PRM) technique. In addition, scanning electron microscopy (SEM), transmission electron microscopy (TEM), confocal laser scanning microscopy (CLSM), and extracellular alkaline phosphatase activity (AKP) assay were used to investigate the possible antimicrobial mechanism of suberanilic acid against MRSA. This study aimed to elucidate the antibacterial mechanism of suberanilic acid and provide a theoretical basis for its further effective application in food and pharmaceuticals.

## 2. Results

### 2.1. Antimicrobial Effect of Suberanilic Acid

**Figure 1:**
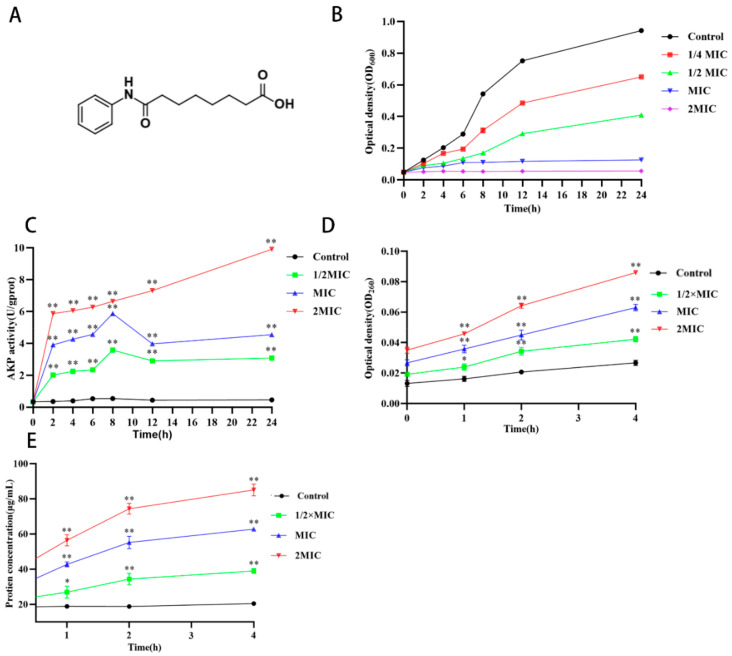
Antibacterial activity of suberanilic acid against MRSA. The structure of suberanilic acid (**A**), growth curve of MRSA (**B**), effect of suberanilic acid treatment of MRSA on AKP activity (**C**), nucleic acid (**D**), and protein leakage (**E**) of MRSA treated with different concentrations. Data are mean ± SD. ** indicates *p* < 0.01 and * indicate *p* < 0.05 compared with control.

The antimicrobial activity of suberanilic acid (Figure 1A) was evaluated by micro broth dilution and agar plate colony counting methods, as described by Rocha et al. (2020) [9]. Suberanilic acid showed strong antimicrobial activity against MRSA with MIC and MBC values of 32 μg/mL and 64 μg/mL, respectively. The 1/4 MIC, 1/2 MIC, MIC, and 2 MIC conditions of suberanilic acid on the growth of MRSA are shown in Figure 1B. The concentrations of suberanilic acid all had different degrees of inhibitory effects on MRSA, and 1/2 MIC concentration of suberanilic acid reduced the maximum number of cells of MRSA within 24 h. The MIC and 2 MIC of suberanilic acid completely inhibited the growth of MRSA within 24 h. The results showed that the suberanilic acid concentration of 1/2 MIC inhibited the growth of MRSA within 24 h. The above results indicate that the concentration and duration of action of suberanilic acid have a major influence on the antimicrobial effect.

The results of the antibiotic effects of suberanilic acid in combination with 12 antibiotics against MRSA ATCC43300 are shown in Table 1. Suberanilic acid showed partial synergistic or additive effects in combination with linezolid, clindamycin, flucytosine, ampicillin, gentamicin, tobramycin, and enrofloxacin. Suberanilic acid showed unrelated effects with FICI values of 1.25, 1.125, 2.25, and 2.25 in combination with benzoxiline, doxycycline, vancomycin, and amikacin, respectively. Moreover, several antibiotics (benzoxacillin, linezolid, tobramycin, and amikacin) showed a four-fold or more reduction in MIC values in combination with suberanilic acid, suggesting that suberanilic acid significantly enhances the antibacterial activity of antibiotics.

### 2.2. Disruption of MRSA Cell Wall Membranes by Suberanilic Acid

AKP exists between the bacterial cell wall and the cell membrane, and when the cell wall is damaged, AKP can leak from the cell [10]. Therefore, by detecting changes in AKP activity in the extracellular solution, it can be used as an indicator of changes in MRSA cell wall integrity by suberanilic acid. The AKP viability of the control supernatant was maintained at about 0.5 U. After treatment with 1/2 MIC, MIC, and 2 MIC of suberanilic acid, there was a dramatic increase of 4–19.8-fold over the control. It indicated that suberanilic acid disrupted the integrity of the MRSA cell wall and caused intracellular AKP leakage in a concentration-dependent manner (Figure 1C). SEM and TEM images revealed the morphological and ultrastructural changes of MRSA cells after the action of suberanilic acid.

The release of cytoplasmic components (e.g., nucleic acids and proteins) from MRSA supernatant was examined to study the permeability of MRSA cell membranes by the action of suberanilic acid (Figure 1D,E). The results showed that the nucleic acid content of MRSA supernatant (OD_260nm_ value) was positively correlated with the action time and concentration. Compared with untreated MRSA, the OD_260nm_ values of the supernatant were increased by 1.5-, 2.3-, and 3.2-fold after 4 h of 1/2 MIC, MIC, and 2 MIC treatments, respectively (Figure 1D). Consistent with the trend of supernatant protein leakage, the nucleic acid content was highly significantly increased in the MIC and 2 MIC treatment groups (*p* < 0.01) (Figure 1E).

**Figure 2:**
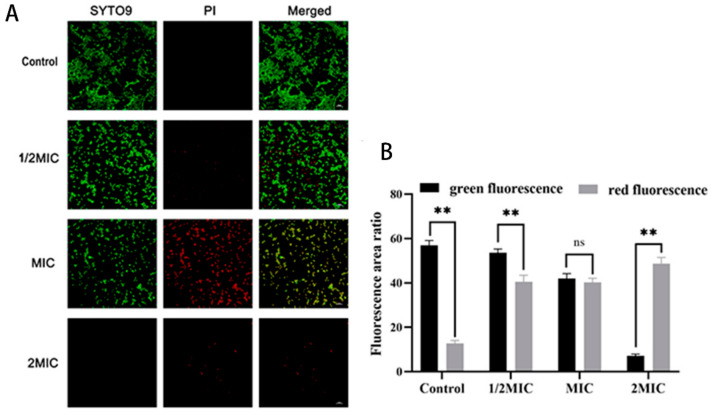
Effect of different concentrations of suberanilic acid on cell viability of MRSA. (**A**) CLSM analysis of SYTO9/PI staining (1000×). Cells stained with SYTO9 were labeled green, and cells stained with PI were labeled red. (**B**) Ratio analysis of fluorescence area of live (green fluorescence) and dead (red fluorescence) bacteria of suberanilic-acid-acting MRSA. Results are expressed as mean ± standard deviation (*n* = 3) for comparison between groups, ** indicates *p* < 0.01, and ns indicates *p* > 0.05.

In addition, the CLSM analysis of SYTO9/PI staining showed that the ratio of live to dead cells after treatment with various concentrations of suberanilic acid was reduced as the dose was increased (Figure 2A,B, *p* < 0.01).

### 2.3. Ultrastructural Changes in MRSA in the Presence of Suberanilic Acid

**Figure 3:**
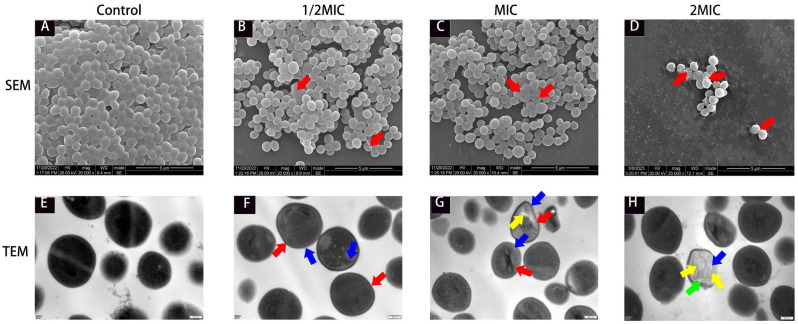
SEM and TEM images of MRSA cells treated with suberanilic acid for 12 h. SEM images (**A**–**D**) of control cells, 1/2-MIC-treated cells, MIC-treated cells, and 2 MIC suberanilic-acid-treated cells. TEM images (**E**–**H**) of control cells, 1/2-MIC-treated cells, MIC-treated cells, and 2 MIC suberanilic-acid-treated cells.

SEM and TEM were used to observe the morphological and ultrastructural changes in MRSA cells under the effect of suberanilic acid. In the control group, MRSA cells showed typical spherical or oval staphylococcal morphology, the extracellular matrix was interconnected to form cell clusters, the internal ultrastructure was clear and intact, the cell wall was intact and smooth, the cell membrane was continuous and clear, and the cytoplasm was uniformly and densely distributed (Figure 3A,E). MRSA cells in the treatment group showed bacteriophage irregular wrinkles (red arrows in Figure 3B–D), cytoplasmic wall separation (blue arrows), disappearance of the cell wall, rupture of the cell membrane (red arrows in Figure 3F–H), and even loss of cytoplasmic contents (orange arrows) to form an empty vesicle structure (Figure 3F–H).

### 2.4. TMT Proteomic Analysis of MRSA Action by Suberanilic Acid

**Figure 4:**
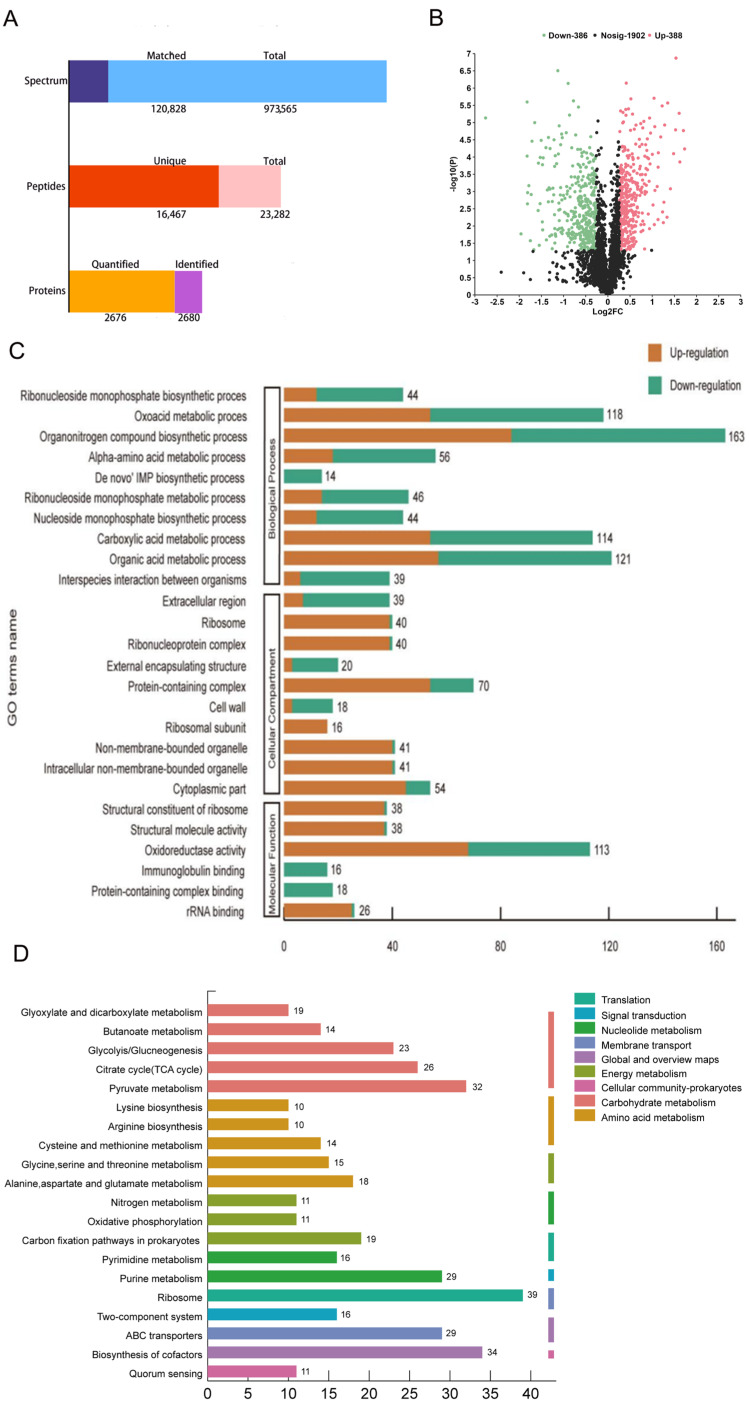
TMT quantitative proteomics analysis. Statistical histogram of the number of peptides matched to proteins and the number of proteins quantitatively identified (**A**); volcano plot of DEPs (**B**); gene ontology (GO) enrichment analysis of DEPs into biological processes, cellular components, and molecular functions (**C**); Kyoto Encyclopedia of Genes and Genomes (KEGG) enrichment analysis of DEPs (**D**).

TMT proteomics was used to analyze the DEPs between control and treatment groups to explore the potential molecular mechanism of the antimicrobial effect of suberanilic acid on MRSA. As shown in Figure 4A, the total number of secondary spectra obtained was 973,565, and the total number of database-matched spectra was 120,828, with a spectral utilization rate of 12.41%. Among them, the total number of peptides was 23,282, the total number of unique peptides was 16,467, 2680 proteins were identified by database matching, and 2676 proteins were quantified. Detailed data of the relevant proteins (protein score, coverage, number of peptides matched to individual proteins, and login number assigned to each identified protein) are shown in Supplementary Table S2. In addition, compared with untreated MRSA, a total of 774 DEPs were identified in the suberanilic-acid-treated group (Figure 4B), of which 388 protein expression levels were significantly upregulated, and 386 protein expression levels were significantly downregulated. The proteomics data reported in this paper have been deposited in the iProX of the China National Bioinformation Center for Bioinformatics Centre/Beijing Institute of Genomics, Chinese Academy of Sciences, under the accession number PXD048581, at http://www.iprox.org.

#### 2.4.1. Functional Classification of Differentially Expressed Proteins

DEPs were classified into functional groups by GO annotations to investigate their specific biological events in response to suberanilic-acid-acting MRSA DEPs. As shown in Figure 4C, 26 GO entries were obtained, including 10 bioprocess entries, 10 cellular component entries, and 6 molecular function entries. Among biological processes, 163 DEPs (21.48%) were associated with organic nitrogen compound biosynthesis; among cellular components, protein complexes (70 DEPs, 18.46%), cytoplasmic fractions (54 DEPs, 14.25%), non-membrane-structured organelles (41 DEPs, 10.82%), and ribonucleoprotein complexes (40 DEPs, 10.55%) were enriched with DEPs; in molecular functions, they were involved in oxidoreductase activity (113 DEPs, 45.38%), ribosomal structural components (38 DEPs, 15.26%) and structural molecular activities (38 DEPs, 15.26%).

#### 2.4.2. Kyoto Encyclopedia of Genes and Genomes Analysis of Differentially Expressed Proteins

KEGG pathway enrichment analysis was performed through 774 DEPs to investigate the key biological pathways they are involved in. As shown in Figure 4D, DEPs were associated with metabolic pathways, including carbohydrate metabolism (95 DEPs) and amino acid metabolism (67 DEPs).

Particularly in carbohydrate metabolism, suberanilic acid treatment of MRSA altered 32 proteins responsible for pyruvate metabolism, 26 citric acid cycle proteins, 23 glycolytic pathway proteins, and 14 butyric acid metabolism proteins (Table 2). The above metabolism-related proteins were associated with membrane transporter proteins (29 DEPs), such as ABC transporter proteins and phosphate-binding proteins. Carbohydrate metabolism in suberanilic-acid-treated MRSA was regulated by a variety of differentially expressed transporter proteins (pstS, cycB, modA, and hrtA), and the DEPs involved in the citric acid cycle (icd, fumC, sucD, and pdhA) appeared altered. In addition, we identified a few downregulated proteins involved in the metabolism of alanine, aspartate, and glutamate: adenylosuccinate synthetase (purA), argininosuccinate lyase (argH), and glutamine-fructose-6-phosphate aminotransferase (glmS).

Suberanilic acid treatment of MRSA altered the expression of DEPs involved in signaling such as the response regulator protein (VraR) fold change of 0.816 (Table 2).

In the synthesis of ribosomal components, suberanilic acid upregulated the expression of 30S ribosomal protein (rpsQ and rpsI) and 50S ribosomal protein (rpmD and rplC, except rpmG3).

### 2.5. PRM Analysis

From the results of TMT quantitative proteomics, we randomly selected 16 DEPs under suberanilic acid treatment for PRM analysis to verify the effect of their protein expression levels. These included aldA, pdhD, gcvT, icd, fumC, pdhA, purA, glmS, ilvE, arcA, argG, pstS, cycB, vraR, rpsQ, and rpmD. As shown in Table 3, the quantitative proteomic results of PRM and TMT were in complete agreement (Supporting Information S2), which more strongly supports the reliability of TMT quantitative proteomics data.

## 3. Discussion

In recent years, MRSA has developed resistance to various antibiotics and entered the food chain, leading to food poisoning in humans. With the increasing awareness of food safety and the frequent outbreaks of foodborne MRSA infection, the prevention and control of MRSA infection have attracted considerable attention. Therefore, there is an urgent need to develop antimicrobial agents with high efficacy, safety, and broad-spectrum activity to fight against MRSA. One promising strategy involves using the metabolites from endophytic fungi, which have similar metabolic compositions to those of their host plants and offer advantages such as bypassing long growth cycles and low yield associated with medicinal plants. As a result, they have gained recognition as an alternative to conventional antimicrobial drugs [11]. Endophytic fungal metabolites inhibit or kill multidrug-resistant bacteria by targeting bacterial cell wall synthesis, interacting with cell membranes, interfering with protein synthesis, and inhibiting nucleic acid replication and transcription, among other mechanisms [12].

Endophytic fungal extracts of *Ageratina adenophora* exhibit various pharmacological activities, such as antitumor, antimicrobial, antioxidant, and probiotic effects [13,14,15,16]. However, the antimicrobial activity and exact mechanism of action of endophytic fungi of *Ageratina adenophora* have not been fully elucidated. In our previous study, we confirmed that the ethyl acetate fraction of *Pestalotiopsis trachycarpicola* DCL44 possessed potent antimicrobial properties against MRSA [17], and an in-depth study of its mechanism of action is of practical significance. In addition, the results of the antibacterial assay of the ethyl acetate extract component of *Pestalotiopsis trachycarpicola* DCL44, amide alkaloids suberanilic acid, showed that the compound had strong antibacterial activity against MRSA (MIC and MBC values of 32 μg/mL and 64 μg/mL, respectively) and was comparable to that of linezolid, Clindamycin, Florfenicol, Ampicillin and Tobramycin showing a superimposed effect, which could restore the susceptibility of MRSA to a variety of antimicrobial drugs, suggesting its potential as a novel candidate for anti-MRSA therapy.

To the best of our knowledge, this present study is the first to report the antibacterial activity of the amide alkaloid suberanilic acid. Amide alkaloids possess a unique chemical backbone for inhibiting bacterial cell wall synthesis, altering cell membrane permeability, inhibiting bacterial metabolism, inhibiting nucleic acid and protein synthesis, and causing bacterial cell lysis [18]. The above study was consistent with the irregularly wrinkled cell and lysis we observed under the SEM. TEM observation also revealed intracellular dense, cohesive material (Figure 3F–H, blue arrows) resulting from MRSA nucleic acid cohesion and abnormal protein precipitation. Therefore, we speculated that suberanilic acid reduces MRSA activity by disrupting the integrity of the cell wall membrane, which allows suberanilic acid to be transported into the cytoplasm, where it acts directly or indirectly on various macromolecules. This leads to nucleic acid aggregation, abnormal precipitation of proteins, or intracellular substance release, ultimately disrupting the homeostasis of the intracellular environment of MSRA [19]. To further investigate the mechanism of action of suberanilic acid on MRSA, TMT quantitative proteomics was used to analyze the changes in the protein expression profile of MRSA after 12 h incubation with suberanilic acid. The results showed that suberanilic acid incubated in the incubator for 12 h was effective in the treatment of MRSA. The results showed that DEPs related to membrane transporter, carbohydrate metabolism, amino acid metabolism, signal transduction, and ribosome synthesis were significantly expressed after 12 h of MRSA incubation with suberanilic acid, suggesting that suberanilic acid may exert its antimicrobial effect through these pathways.

In the present study, suberanilic acid significantly altered carbohydrate metabolism, amino acid metabolism, and the expression of membrane transport proteins (e.g., phosphate-binding protein) in MRSA (Figure 4D and Table 2). Carbohydrate metabolism is interconnected with energy production, nutrient transport, stress response, and cell membrane integrity and has been identified as a novel strategy against drug-resistant pathogens. This may be related to the presence of unique bacterial enzymes and hypersensitivity to antimicrobial agents by altering their metabolism [20]. Among the carbohydrate metabolic pathways, glycogen isomerization, propionic acid metabolic pathway, and the citric acid cycle (icd, pdhD, and fumC) were upregulated in the present study, suggesting that MRSA requires more energy to maintain normal life activities under suberanilic acid stress. Membrane transporter proteins provide energy for a variety of cellular metabolisms through hydrolysis of ATP and are involved in cellular functions, such as nutrient transport, excretion of toxic and harmful substances, and drug resistance [21]. Quantitative proteomic analysis by TMT revealed that both molybdate ABC transporter substrate-binding protein (modA) and putative hemin import ATP-binding protein H (hrtA) were downregulated, indicating that suberanilic acid reduced the expression of ABC transporter proteins. Suberanilic acid reduced the expression of ABC transporter protein, which led to the reduction in bacterial virulence and the exocytosis of drugs and harmful substances so that it could better perform its bacteriostatic and bactericidal effects.

Amino acids are energy sources and repair materials for bacteria, among which arginine can provide energy for bacterial growth when carbohydrate metabolism is disturbed [22]. argG and argH are key enzymes for arginine biosynthesis; argG catalyzes the formation of arginine succinate from citrulline and aspartic acid, and argH catalyzes the formation of arginine from arginosuccinate [23]. arcA is the enzyme that catalyzes the hydrolysis of arginine in the arginine deiminase pathway, which protects bacteria from acidic environments to provide them with energy [24]. Treatment with suberanilic acid significantly downregulated arginine synthesis-related proteins (arcA, argG, and argH) and inhibited further arginine synthesis, resulting in arginine deficiency in amino acid metabolism and a reduction in energy for MRSA survival. Therefore, the survival of MRSA could not be maintained by amino acid metabolism under suberanilic acid stress.

Bacterial growth consumes about 50% of energy from proteins, and 20–40% of protein synthesis is directly used for ribosomes and translation factors [25]. Ribosomal proteins influence bacterial biofilms and antibiotic resistance; for example, 50S ribosomal proteins are binding sites for macrolides, lincosamides, and streptomycin, and 30S ribosomal proteins serve as targets for tetracyclines and aminoglycosides [26]. Suberanilic acid acted on the MRSA ribosomal pathway to enrich the highest number of DEPs. The expression of ribosome-associated proteins involved in transcription and genetic information processing, including rpsQ, rpmD, rpsI, and rplC (except for the 50S ribosomal protein L33 rpmG3), was significantly upregulated. We inferred that changes in the expression of the above-mentioned ribosomal proteins would most likely affect the transcription and translation of ribosomal proteins, leading to the disruption of protein synthesis in MRSA, thereby affecting the structure of the cell membrane, cytoplasm, and nucleus as well as inhibiting MRSA protein synthesis [26]. A study by Ning et al. [27] reported that the expression of the ribosomal structural proteins rpsC, rplV, rplU, rplM, rpsM, and rpsE was significantly upregulated in *Bacillus cereus* by phenyl lactic acid, which interferes with and disrupts the transcriptional and translational processes of *Bacillus cereus*, which is consistent with the results of this study.

## 4. Material and Methods

### 4.1. Compounds and Bacterial Cultures

Suberanilic acid (HPLC purity ≥ 98%) is an endophytic fungus from Ageratina adenophora, *Pestalotiopsis trachycarpicola* DCL44 (GenBank:MZ066737). Obtained by fermentation and extraction with ethyl acetate, identified by Xili Biotechnology Co., Ltd. (Yunnan, China), The NMR data and figure are available in Supplementary Figures S1–S10 and Supplementary Table S1. The compound was dissolved in dimethyl sulfoxide (DMSO; St. Louis, MO, USA), which formulated a stock solution for all experiments.

MRSA ATCC43300 was deposited in the College of Animal Medicine, Sichuan Agricultural University (Chengdu, China), and the strains were cultured on Mueller–Hinton (MH) agar plates (containing beef extract powder 6 g/L, soluble starch 1.5 g/L, acid hydrolyzed casein 17.5 g/L, agar 17 g/L, and pH = 7.3 ± 0.1) at 37 °C for 24–48 h. Thereafter, the strains were transferred to 5 mL of MH broth (Oxoid Ltd., Hampshire, UK) at 180 r/min for overnight incubation. Then, the single colonies of MRSA were collected and transferred to 5 mL of MH broth for overnight incubation at 37 °C and 180 r/min with shaking, and the OD_600_ optical density value of the bacterial solution was determined, and it was diluted to 1.0 × 10^5^ CFU/mL.

### 4.2. Antimicrobial Activity

#### 4.2.1. Determination of Minimum Inhibitory Concentration (MIC) and Minimum Bactericidal Concentration (MBC)

The MIC and MBC of suberanilic acid against MRSA ATCC43300 were determined using the micro broth dilution method as described previously [28]. Suberanilic acid was dissolved in sterile Mueller–Hinton Broth (MHB), and a suspension of MRSA (1.0 × 10^5^ CFU/mL) with a concentration of 1–1024 μg/mL of suberanilic acid was added to a 96-well plate and incubated at 37 °C for 18–24 h. The lowest concentration of suberanilic acid at which no bacterial growth was seen was the MIC. Based on the results of the MIC assay, 50 μL of culture medium from the wells in which no MRSA growth was seen was taken for counting of colonies in the agar plates, and the lowest concentration at which no bacterial growth was seen was the MIC on the MH agar plates. The minimum concentration of suberanilic acid at which no colony growth was seen was MBC, and florfenicol was used as an anti-MRSA positive control drug.

#### 4.2.2. Effect of Suberanilic Acid on Growth Curves

MRSA exponential phase suspensions (approximately 1.0 × 10^5^ CFU/mL) were added with suberanilic acid (final concentrations of 0 MIC, 1/4 MIC, 1/2 MIC, MIC, and 2 MIC, respectively) and placed in incubation for 24 h at 37 °C, 180 r/min. Selected time intervals (0 h, 2 h, 4 h, 6 h, 8 h, 12 h, and 24 h) OD_600_ values were determined by Microplate Reader (Thermo Fisher Scientific, Waltham, MA, USA).

#### 4.2.3. Fractional Inhibition Concentration Index of Antibiotics and Suberanilic Acid

The interaction between pentalic acid and antibiotics was studied by fractional inhibitory concentration, and the MIC of suberanilic acid and antibiotics were determined, respectively [29]. Different concentrations of the 2 antimicrobial agents and bacterial suspensions (1.0 × 10^5^ CFU/mL) were added to 96-well plates by checkerboard method and incubated at 37 °C for 18–24 h. The combined antimicrobial effect of suberanilic acid and antibiotics was determined based on fractional inhibition concentration index (FICI) values. The formula for calculating the FICI index and the criteria for interpreting it were as follows:

$$\mathrm{FICI}=\frac{MIC\;(suberanilic\;acid\;in\;combination)}{MIC\;(suberanilic\;acid\;alone)}+\frac{MIC\;(antibiotic\;in\;combination)}{MIC\;(antibiotic\;alone)}$$

when FICI index ≤0.5, it is synergistic; 0.5 < FICI ≤ 0.75 is partially synergistic; 0.75 < FICI ≤ 1 is additive; 1 < FICI ≤ 4 is irrelevant; and FICI index > 4 is antagonistic [30].

### 4.3. Effect of Suberanilic Acid on MRSA

#### 4.3.1. Detection of Alkaline Phosphatase Leakage

To assess the effects of suberanilic acid on cell wall integrity of MRSA, the extracellular AKP activity was determined in supernatants [31]. Supernatants were collected at time intervals (0 h, 2 h, 4 h, 6 h, 8 h, 12 h, and 24 h) as previously described, and the supernatant AKP was determined by reference to an AKP activity kit (Nanjing Jiancheng Institute of Biological Engineering, Nanjing, Jiangsu, China) to determine the supernatant AKP content.

#### 4.3.2. Nucleic Acid and Protein Leakage Detection

Cell membrane integrity was assessed by measuring cytoplasmic nucleic acids and proteins released from suberanilic-acid-treated MRSA [32]. MRSA suspensions were collected as previously described and at time intervals (1 h, 2 h, 3 h, and 4 h) and filtered through 0.22 µm BeyoGold filters. Nucleic acids were measured as OD_260_ values of the supernatants using a microplate reader (Thermo Fisher Scientific, Waltham, MA, USA), and protein leakage was quantified using a BCA protein concentration kit (Solarbio, Beijing, China).

#### 4.3.3. Effect of Suberanilic Acid on MRSA Viability

MRSA exponential phase (approximately 1.0 × 10^5^ CFU/mL) suspensions were added to different concentrations of suberanilic acid (final concentrations of 0 MIC, 1/2 MIC, MIC, and 2 MIC, respectively). A total of 0.85% saline was used to wash the cells three times, and the bacterial suspensions were stained by mixing equal amounts of SYTO 9 and propidium iodide (PI) at dark room temperature for 15 min. In total, 5 μL of the stained bacterial suspensions were pipetted onto slides, and CLSM (Olympus, Tokyo, Japan) was used to observe the images and analyze the fluorescence area ratio values using Aipathwell v2 software [33].

#### 4.3.4. SEM and TEM Observations

After incubation, the bacterial suspension was centrifuged at 4 °C for 10 min at 5000 r/min to collect the precipitate, which was fixed with 2.5% glutaraldehyde at 4 °C for 12 h; it was washed with PBS twice, each time for 5 min. Dehydration was carried out by passing it through different gradients of ethanol (30%→50%→70%→80%→90%→95%→100%). A small amount of bacterial suspension was aspirated and added dropwise to the slide, which was stuck on conductive adhesive, ion sputter sprayed, and observed using an FEI Inspect scanning electron microscope (Thermo Fisher Scientific, Waltham, MA, USA). The prepared bacterial suspension was centrifuged at 4 °C for 10 min at 5000 r/min to collect the precipitate as described previously, with minor modifications, and the samples were processed as described [34,35]. The sample sections were observed by JEM-1400 FLASH transmission electron microscopy (Jeol, Tokyo, Japan).

### 4.4. TMT Quantitative Proteomics Analysis of Differentially Expressed Proteins

MRSA ATCC43300 was cultured to exponential phase at 37 °C, 180 r/min with shaking and diluted to 1.0 × 10^5^ CFU/mL. It was then evenly divided into control and treatment groups, and suberanilic acid was added to the treatment group to make its final concentration 1/2 MIC (16 μg/mL), and the control group was added with an equal amount of MH broth, and 3 biological replicates were added in each group. The samples were incubated at 37 °C, 180 r/min for 12 h, centrifuged at 4 °C, 5000 rpm for 10 min, and the supernatant was discarded. The samples were washed three times repeatedly with PBS, and the samples were snap-frozen in liquid nitrogen and then immediately sent to Shanghai Applied Protein Technology Co., Ltd. (Shanghai, China) for TMT quantitative proteomics testing. Sample quality control, protein extraction, peptide digestion, TMT labeling, mixed peptide grading, and LC-MS/MS analyses were performed according to Shi et al. (2018) [36], and the analytical data were uploaded to the Zhongke New Life online cloud platform (https://bio-cloud.aptbiotech.com, accessed on 1 December 2022). Expression fold change thresholds (≥1.2 or ≤0.83) with *p*-value <0.05 were identified as DEPs.

### 4.5. PRM Analysis of Key Proteins

The samples after HPLC separation were analyzed by PRM mass spectrometry using a Q-Exactive HF mass spectrometer (Thermo Fisher Scientific). Analysis time: 60 min, detection mode: positive ions. MS1 scan range: 300–1800 *m*/*z*, MS resolution: 60000 (*m*/*z* 200), AGC target: 3e6, Maximum IT: 200 ms. Sixteen PRM scans (MS2 scans) were collected according to the Inclusion list after the first full MS scan. Isolation window: 1.6 Th, MS resolution: 30,000 (*m*/*z* 200), AGC target: 3e6, Maximum IT: 120 ms, MS2 Activation Type: HCD, Normalized collision energy: 27. The Skyline 3.5 software was used for relative quantitative analysis of PRM data (3 biological replicates).

## 5. Conclusions

In summary, suberanilic acid, a secondary metabolite from the endophytic fungus of *Ageratina adenophora*, demonstrated significant antimicrobial activity against MRSA. It exhibited clear antimicrobial properties, such as reducing the maximal bacterial population, decreasing bacterial viability, causing cell wall damage, and compromising cell membrane integrity. To further investigate how MRSA responds to suberanilic acid treatment, TMT quantitative proteomics was used, and the results revealed that suberanilic acid targets multiple MRSA pathways, such as dysregulation of carbohydrate metabolism, disruption of amino acid metabolism, and interference with ribosome synthesis. In addition, PRM validation of randomly selected DEPs was consistent with the results of TMT quantitative proteomics, confirming that the results of TMT quantitative proteomics are reliable. This study provides new insights into the antimicrobial mechanism of endophytic fungal secondary metabolites against MRSA and provides an important contribution to the development of novel antimicrobial agents against MRSA resistance. However, future studies should evaluate the efficacy and safety of suberanilic acid as an antimicrobial agent and its potential application to control the spread of MRSA in the food chain.

## Figures and Tables

**Table 1:** FICI values of suberanilic acid in combination with antibiotics.

Name of Antibiotic	MIC (µg/mL)	Drug Combination MIC		FICI	Result
		Antibiotics (µg/mL)	Suberanilic Acid (µg/mL)		
Oxacillin	128	32	32	1.25	I
Linezolid	2	0.125	32	0.5625	P
Vancomycin	0.5	1	32	2.25	I
Clindamycin	256	256	32	0.75	P
Sarafloxacin	64	128	32	2.125	I
Florfenicol	32	16	32	0.75	P
Ampicillin	64	32	32	0.75	P
Gentamycin	64	32	32	1	A
Doxycycline	0.5	0.5	32	1.125	I
Tobramycin	256	16	32	0.5625	P
Enrofloxacin	0.25	0.125	32	1	A
Amikacin	256	64	32	2.25	I

Note: P, partial synergy; A, additivity; I, irrelevant.

**Table 2:** Representative DEPs of MRSA treated with suberanilic acid.

Protein ID	Gene	Description	Fold Change	Change	*p*-Value
**Metabolism**					
Q2FK94	aldA	Putative aldehyde dehydrogenase AldA	1.948	Up	2.01521 × 10^−5^
Q5HGY8	pdhD	Dihydrolipoyl dehydrogenase	1.534	Up	0.000545771
A0A033V2M2	pfkA	ATP-dependent 6-phosphofructokinase	0.828	Down	0.020923953
P64225	gcvT	Aminomethyltransferase	1.857	Up	0.000233977
Q6GG12	icd	Isocitrate dehydrogenase (NADP)	2.548	Up	2.69982 × 10^−6^
Q6GFK5	fumC	Fumarate hydratase class II	1.462	Up	8.04802 × 10^−5^
Q6GHI9	sucD	Succinate–CoA ligase subunit alpha	1.307	Up	0.004931368
A0A8E6CNC9	sucB	Dihydrolipoyllysine-residue succinyltransferase	1.852	Up	0.000103329
A0A0U1MI53	pdhA	Pyruvate dehydrogenase E1 component subunit alpha	1.496	Up	0.001443745
P65884	purA	Adenylosuccinate synthetase	0.576	Down	0.000180435
Q6GAW6	argH	Argininosuccinate lyase	0.404	Down	5.68 × 10^−5^
A0A2S6D4J7	glmS	Glutamine-fructose-6-phosphate aminotransferase	0.799	Down	0.000120906
Q6GJB4	ilvE	Probable branched-chain-amino-acid aminotransferase	0.681	Down	0.000162841
A0A850FXK9	cysK	Cysteine synthase	0.707	Down	0.015964978
Q6GDG7	arcA	Arginine deiminase	0.301	Down	0.001221858
Q6GIC7	argG	Argininosuccinate synthase	0.147	Down	7.32963 × 10^−6^
Q6GAW6	argH	Argininosuccinate lyase	0.404	Down	5.68 × 10^−5^
**Membrane transport**					
Q6GH18	pstS	Phosphate-binding protein PstS	1.544	Up	0.009992197
A0A660A2T8	cycB	Maltodextrin-binding protein	0.799	Down	0.001032071
A0A0U1MSA4	modA	Molybdate ABC transporter substrate-binding protein	0.621	Down	0.00703426
A0A2S6DD31	hrtA	Putative hemin import ATP-binding protein HrtA	0.723	Down	0.008434244
**Signal transduction**					
Q5HEP0	vraR	Response regulator protein VraR	0.816	Down	0.017176601
**Translation**					
A6QJ83	rpsQ	30S ribosomal protein S17	1.443	Up	0.023323817
A8Z339	rpmD	50S ribosomal protein L30	1.511	Up	0.010285859
Q6GJD4	rpmG3	50S ribosomal protein L33 3	0.555	Down	0.01660196
P66645	rpsI	30S ribosomal protein S9	1.272	Up	0.001495131
A0A850G4M4	rplC	50S ribosomal protein L3	1.326	Up	6.12434 × 10^−6^

**Table 3:** Comparison of relative quantitation results between TMT and PRM.

No.	Accession	Gene	TMT Fold Change	PRM		
				*p*-Value	Fold Change	Trend
1	Q2FK94	aldA	1.948 ↑	0.002506652	3.468 ↑	Consistency
2	Q5HGY8	pdhD	1.534 ↑	0.024147859	1.965 ↑	Consistency
3	P64225	gcvT	1.857 ↑	0.007774184	3.685 ↑	Consistency
4	Q6GG12	icd	2.548 ↑	0.000980574	3.874 ↑	Consistency
5	Q6GFK5	fumC	1.462 ↑	0.000550982	2.589 ↑	Consistency
6	A0A0U1MI53	pdhA	1.496 ↑	0.00642461	2.194 ↑	Consistency
7	P65884	purA	0.576 ↓	0.023770336	0.390 ↓	Consistency
8	A0A2S6D4J7	glmS	0.799 ↓	0.932842293	1.017 ↓	Consistency
9	Q6GJB4	ilvE	0.681 ↓	0.088087529	0.723 ↓	Consistency
10	Q6GDG7	arcA	0.301 ↓	0.001021429	0.154 ↓	Consistency
11	Q6GIC7	argG	0.147 ↓	0.001274761	0.012 ↓	Consistency
12	Q6GH18	pstS	1.544 ↑	0.093222288	1.783 ↑	Consistency
13	A0660A2T8	cycB	0.799 ↓	0.199271957	0.797 ↓	Consistency
14	Q5HEP0	vraR	0.816 ↓	0.965351503	1.006 ↓	Consistency
15	A6QJ83	rpsQ	1.443 ↑	0.008784577	2.012 ↑	Consistency
16	A8Z339	rpmD	1.511 ↑	0.004055361	1.613 ↑	Consistency

Note: Consistency.

## Data Availability

Request from corresponding author with reasonable reason.

## Supplementary Materials

The following supporting information can be downloaded at: https://www.mdpi.com/article/10.3390/molecules29174205/s1.
