# Supplementary material for: Antibacterial Activity and Multi-Targeted Mechanism of Action of Suberanilic Acid Isolated from Pestalotiopsis trachycarpicola DCL44: An Endophytic Fungi from Ageratina adenophora
Source: Molecules. 2024 Sep 4;29(17):4205. doi: 10.3390/molecules29174205 (PMC11396930; doi:10.3390/molecules29174205)
Supplement: Supplementary file 1 [file molecules-29-04205-s001.zip › Supplementary Figures S1-S10.pdf]

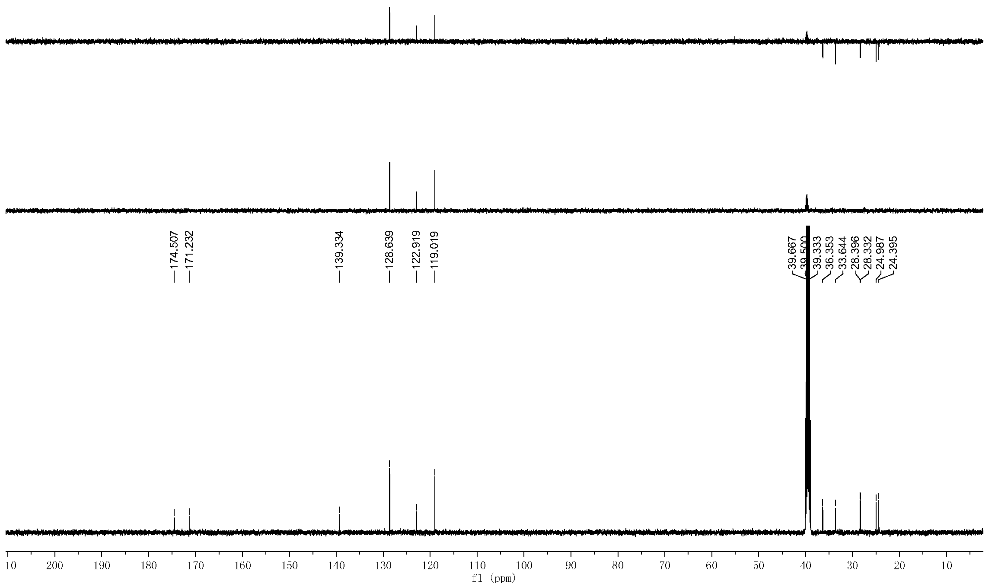

Supplementary Figure S1. ^{13}C NMR spectrum (125 MHz) of Suberanilic acid in DMSO-*d*$_6$

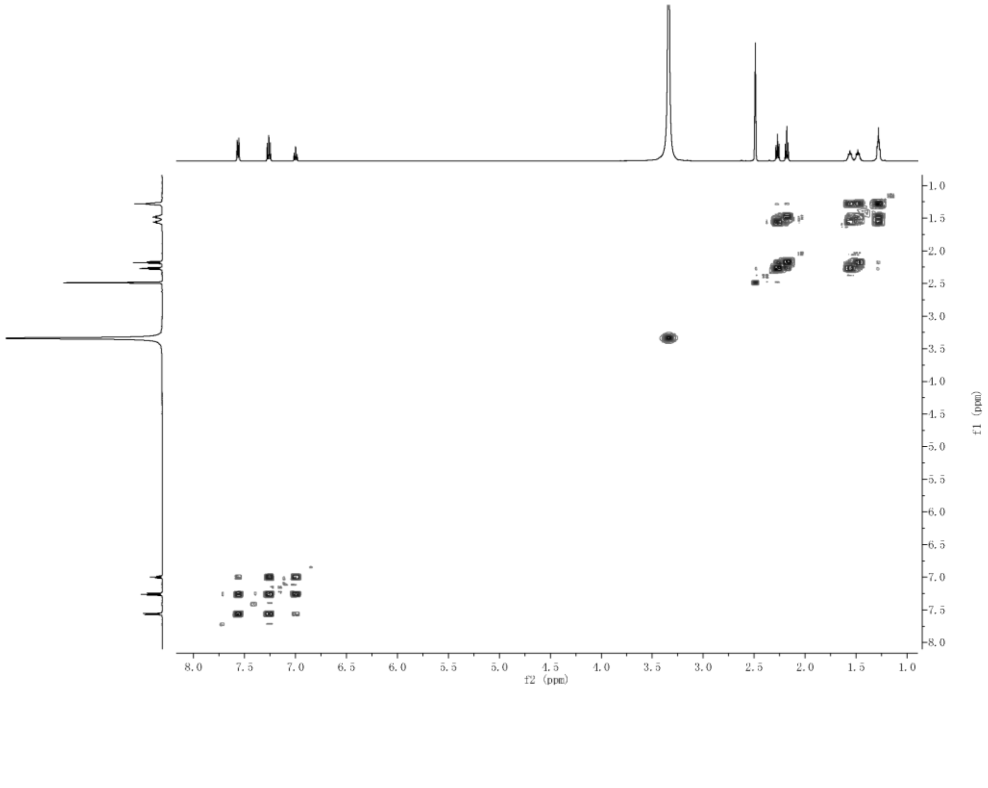

Supplementary Figure S2. ^{1}H-^{1}H COSY spectrum of Suberanilic acid in DMSO-*d*$_6$

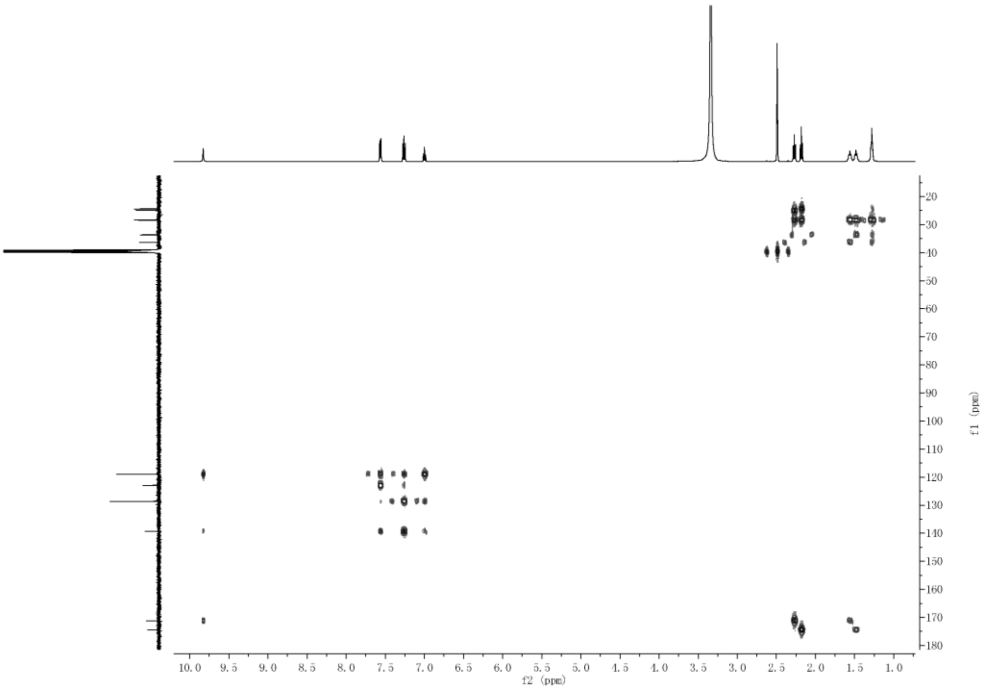

Supplementary Figure S3. HMBC spectrum of f Suberanilic acid in DMSO-d_6

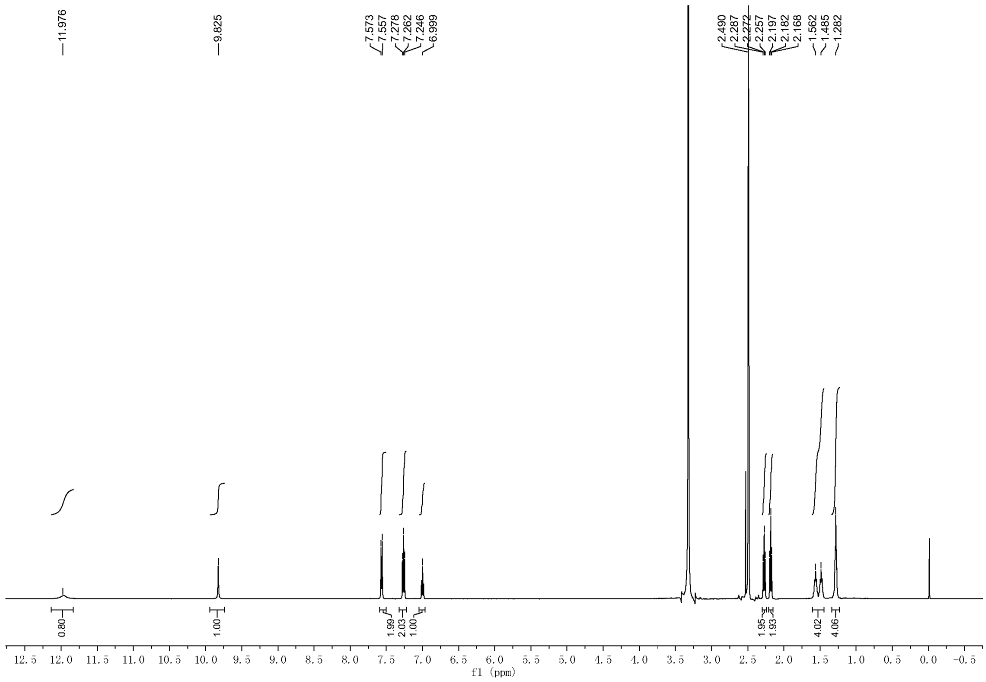

Supplementary Figure S4. ^{1}H NMR spectrum (500 MHz) of Suberanilic acid in DMSO-d_6

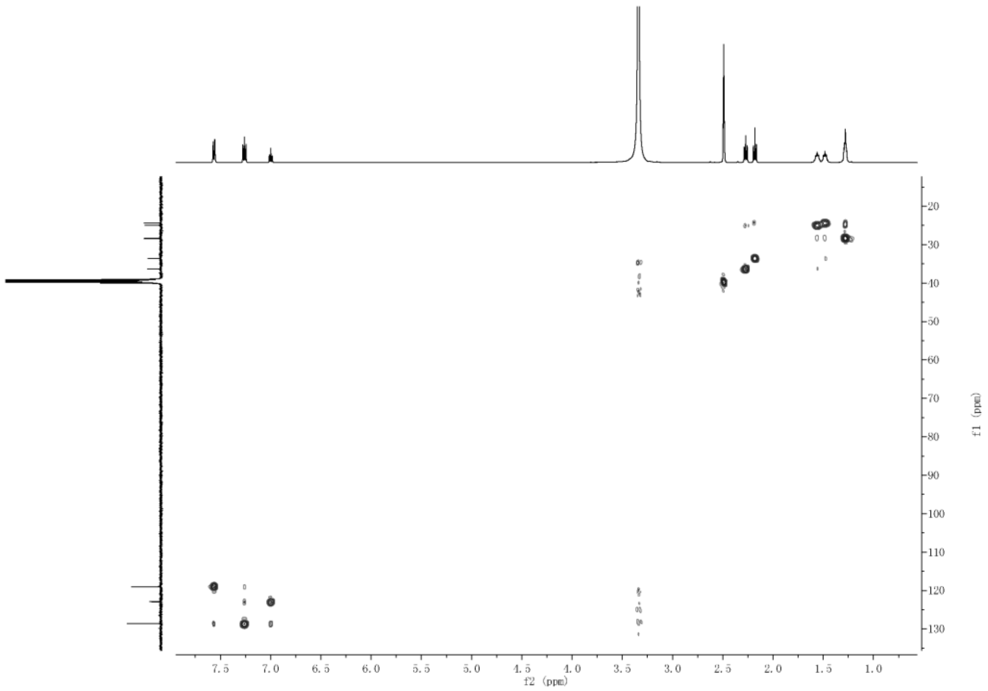

Supplementary Figure S5. HSQC spectrum of Suberanilic acid in DMSO-*d*$_6$

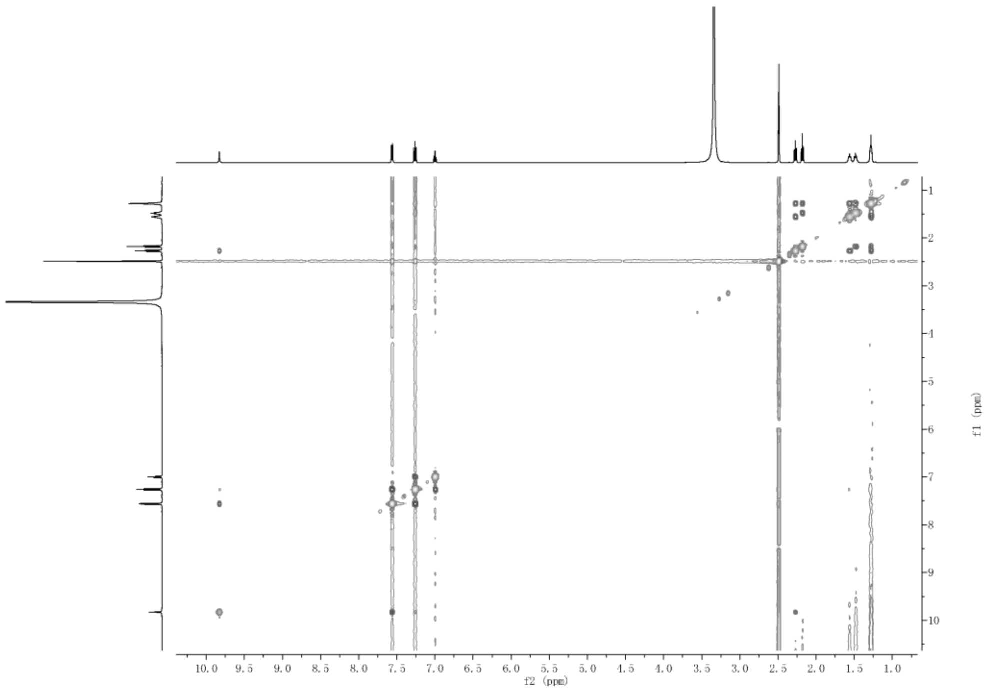

Supplementary Figure S6. ROESY spectrum of Suberanilic acid in DMSO-*d*$_6$

==== LCMSsolution Data Report ====

Sample Information

Acquired by	: Admin	
Date Acquired	: 2022/11/16 12:20:18	
Sample Name	: Ehr686	
Data File	: Ehr686.lcd	
Method File	: 阻尼管一级100-1500.lcm	

System Configuration
<<Instrument>> : LC-IT-TOF

<Spectrum>
Retention Time:0.587(Scan#:90)
Spectrum:Averaged 0.480-0.707(73-107)
Background:Averaged 0.000-0.470(1-71) MS Stage:MS Polarity:Pos Segment1 - Event1 Precursor:----- Cutoff:

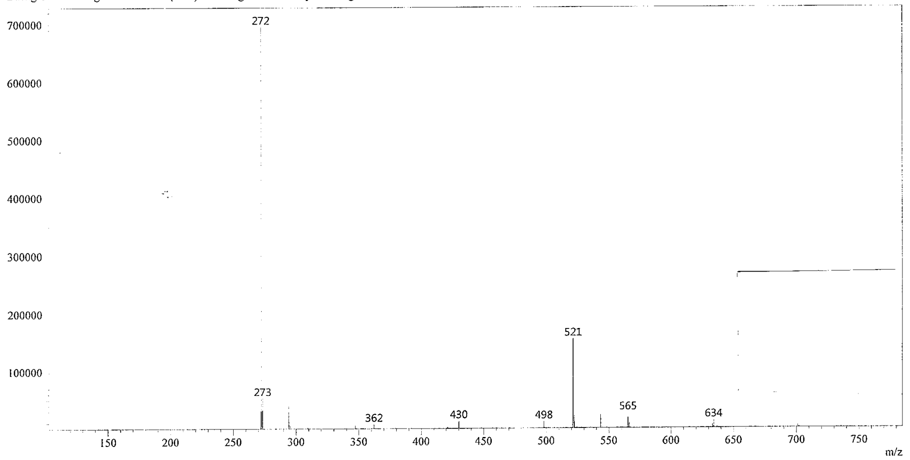

Supplementary Figure S7. ESI spectrum of Suberanilic acid

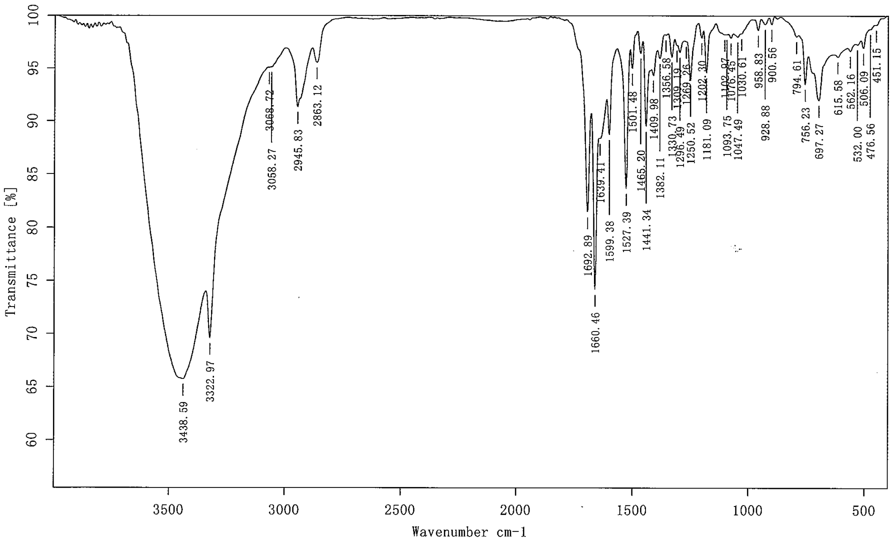

Sample Name: Ehr686	Resolution: 4	Beamsplitter Setting: KBr
Sample Form: KBr	Aperture Setting: 6 mm	Source Setting: MIR
Path of File: E:\data	Number of Background Scans: 16	Instrument Type: BRUKER VERTEX 70
Date of Measurement: 2022/11/21	Number of Sample Scans: 16	Soft Version: OPUS8.1

Supplementary Figure S8. IR spectrum of Suberanilic acid

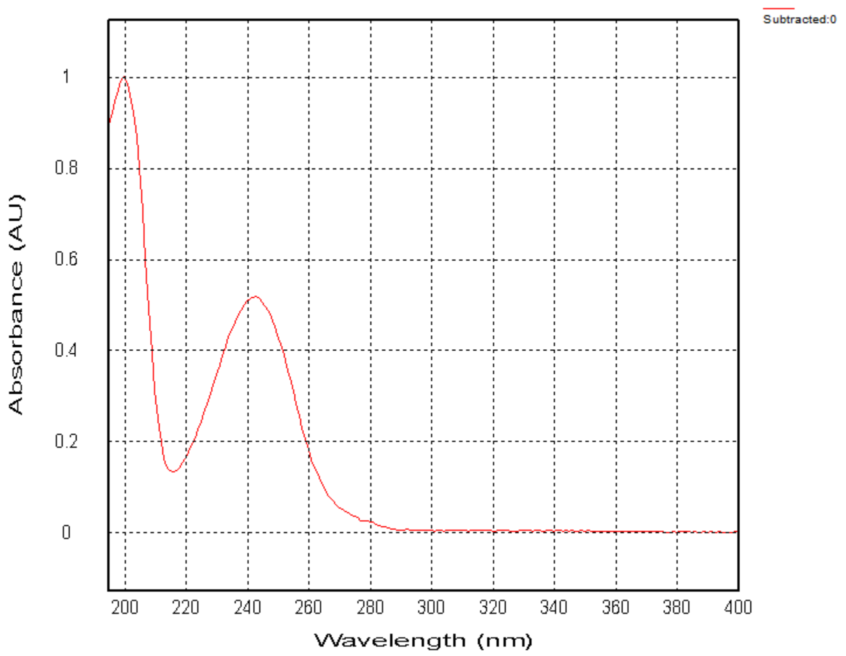

Supplementary Figure S9. UV spectrum of Suberanilic acid in CD_3OD

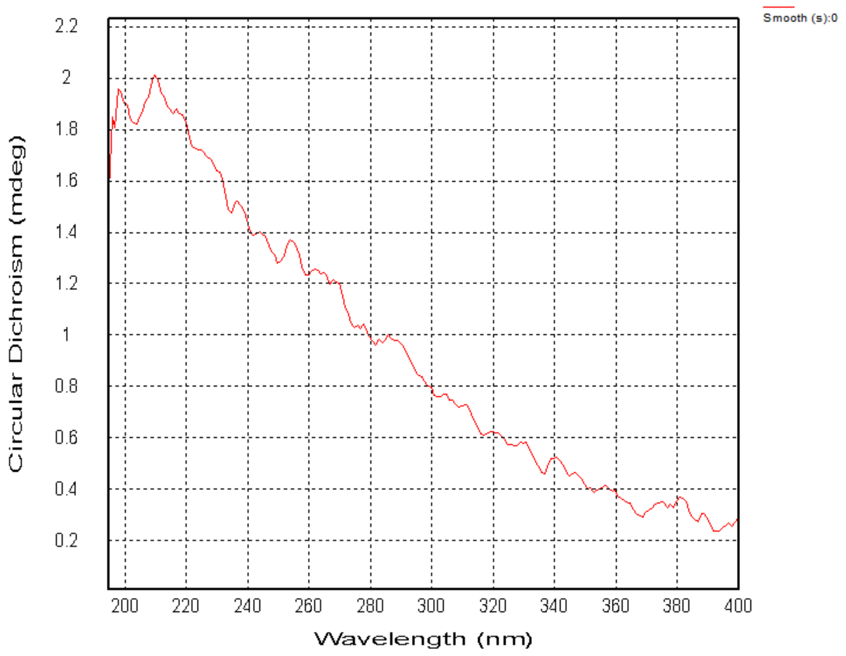

Supplementary Figure S10. ECD spectrum of Suberanilic acid in CD_3OD
