# Supplementary material for: Antibacterial Activity and Multi-Targeted Mechanism of Action of Suberanilic Acid Isolated from Pestalotiopsis trachycarpicola DCL44: An Endophytic Fungi from Ageratina adenophora
Source: Molecules. 2024 Sep 4;29(17):4205. doi: 10.3390/molecules29174205 (PMC11396930; doi:10.3390/molecules29174205)
Supplement: Supplementary file 1 [file molecules-29-04205-s001.zip › Supplementary Table S1.pdf]

Table S1 ^{1}H-NMR and ^{13}C-NMR Nuclear Magnetic data of 2.2 Suberanilic acid (500 MHz, *J* in Hz, δ)

Position	Suberanilic acid	
	δ_H	δ_C（ppm）
1		171.2 (s)
2	2.27 (2H, t, *J* = 7.4 Hz)	36.4 (t)
3	1.56 (2H, m)	25.0 (t)
4	1.29 (2H, m)	28.3 (t)
5	1.26 (2H, m)	28.4 (t)
6	1.49 (2H, m)	24.4 (t)
7	2.18 (2H, t, *J* = 7.5 Hz)	33.6 (t)
8		174.5 (s)
1'		139.3 (s)
2', 6'	7.56 (2H, d, *J* = 7.9 Hz)	119.0 (d)
3', 5'	7.26 (2H, t , *J* = 7.9 Hz)	128.6 (d)
4'	7.00 (1H, t, *J* = 7.9 Hz)	122.9 (d)
NH	9.83 (1H, s)	
COOH	11.98 (1H, br. s)	
$COONH_2$		
