# Supplementary material for: Antibacterial Activity and Multi-Targeted Mechanism of Action of Suberanilic Acid Isolated from Pestalotiopsis trachycarpicola DCL44: An Endophytic Fungi from Ageratina adenophora
Source: Molecules. 2024 Sep 4;29(17):4205. doi: 10.3390/molecules29174205 (PMC11396930; doi:10.3390/molecules29174205)
Supplement: Supplementary file 1 [file molecules-29-04205-s001.zip › Supplementary Table S2.pdf]

Accession	Protein Name	Gene Name	Function	Description	Coverage	Unique Peptides	Peptides
A0A8D9ZMU	Bifunctional a	SHAG_01005		Bifunctional a	73	2	75
A0A8D6TSX2	Formate acet	pflB		Formate acet	78	12	53
Q6GJC5	DNA-directed	rpoC	FUNCTION:	DNA-directed	72	46	89
A0A0D1JMM	Bifunctional a	QU38_05510		Bifunctional a	63	1	66
A0A0U1MHR	Bifunctional a	atl		Bifunctional a	66	1	69
A0A6M4QF7	Bifunctional a	atl_3		Bifunctional a	63	1	65
A0A6B5HNY	Bifunctional a	G0Z49_12525		Bifunctional a	63	1	66
A0A2S6DAD	Bifunctional a	atl_3		Bifunctional a	65	1	67
A7X0T9	Bifunctional a	atl	FUNCTION:	Bifunctional a	61	1	64
A0A8B1CRA	Bifunctional a	JYA66_0009		Bifunctional a	60	1	64
P68790	Elongation fa	fusA	FUNCTION:	Elongation fa	78	1	41
A0A8D9ZK8	Serine-aspart	SDAG_0192		Serine-aspart	57	40	63
A0A8E0EQ0	Aconitate hyd	SAZG_0105		Aconitate hyd	74	34	49
A0A454GZR	DNA-directed	rpoB	FUNCTION:	DNA-directed	64	13	72
A6QEK0	Elongation fa	tuf	FUNCTION:	Elongation fa	88	5	26
A0A2S6DMY	Pyruvate carb	cfiB_2	FUNCTION:	Pyruvate carb	69	60	60
A0A6B5AT15	Immunoglobu	spa		Immunoglobu	77	2	43
H2DFY9	Elongation fa	fusA		Elongation fa	78	1	33
A0A8G0YFB	ATP-depende	HLV54_0005		ATP-depende	63	1	50
A0A6B5LLP7	ATP-depende	CV021_09530		ATP-depende	60	1	46
A0A8D9WA8	Endopeptidas	SADG_0188		Endopeptidas	63	1	49
A0A8G1GB6	Pyrimidine-nu	KSF82_1172		Pyrimidine-nu	91	37	37
Q6GH64	Transketolase	tkt	FUNCTION:	Transketolase	74	3	42
Q6GF43	Chaperonin C	groEL	FUNCTION:	Chaperonin C	81	5	33
Q6GGZ5	2-oxoglutarat	odhA	FUNCTION:	2-oxoglutarat	60	4	43
A0A8F2LLM	Preprotein tra	secA		Preprotein tra	65	26	57
A0A5F0HHX	Transketolase	tkt	FUNCTION:	Transketolase	69	2	39
Q8NUR2	1-pyrroline-5-	rocA		1-pyrroline-5-	62	1	36
A0A8D9ZNY	Succinate de	SHAG_0110		Succinate de	80	16	40
A0A1Q8DCS	DNA-directed	rpoB	FUNCTION:	DNA-directed	52	1	54
Q70AB7	Protein A (Fra	spa		Protein A (Fra	90	1	32
Q6GG09	Pyruvate kina	pyk		Pyruvate kina	76	36	36
Q6GFZ6	Acetate kinas	ackA	FUNCTION:	Acetate kinas	87	10	31
A0A8D9Z4T	Pyruvate deh	SATG_0117		Pyruvate deh	75	12	22
Q08854	Chaperonin C	groEL	FUNCTION:	Chaperonin C	66	1	29
B5LV64	Cell division	ftsZ	FUNCTION:	Cell division	70	7	24
A0A0D1HKA	Chaperone p	dnaK	FUNCTION:	Chaperone p	76	20	43
Q2FHN5	Carbamoyl-pl	carB		Carbamoyl-pl	56	46	46
A0A8D9W5T	Chaperone C	SAAG_0132		Chaperone C	68	12	51
Q2G078	Ribonucleosi	SAOUHSC_0	FUNCTION:	Ribonucleosi	57	1	37
A0A7U4ERC	Immunoglobu	spa		Immunoglobu	68	1	39
A0A2S6DF8	ATP synthase	atpD	FUNCTION:	ATP synthase	78	16	30
A0A8D9ZM0	Formate dehy	SHAG_0009		Formate dehy	53	11	43
A0A1Q8DCQ	Elongation fa	fusA	FUNCTION:	Elongation fa	44	1	25
A0A2X2MAM	Formate acet	pflB_3		Formate acet	82	1	26
A0A2S6DUT	1-pyrroline-5-	pruA		1-pyrroline-5-	62	2	35
A0A850FVJ4	Ribonucleosi	nrdE		Ribonucleosi	58	1	37

A5IT93	Glycine--tRN/	glyQS	FUNCTION: (	Glycine--tRN/	85	35	35
Q6GGT5	30S ribosoma	rpsA	FUNCTION:	30S ribosoma	81	2	32
A0A6B5CZW	2-oxoglutarat	odhA		2-oxoglutarat	57	2	41
A6U2F0	Threonine--tF	thrS	FUNCTION: (	Threonine--tF	57	22	35
A0A6G4IUM9	Immunoglobu	spa		Immunoglobu	69	1	37
A0A380EC03	Immunoglobu	spa_2		Immunoglobu	76	1	33
A0A8E3XVJ7	Aldehyde deh	HK402_0067		Aldehyde deh	74	3	29
A0A1Q8DCS	Elongation fa	tuf	FUNCTION: 7	Elongation fa	58	1	17
Q6GA19	Isoleucine--tF	ileS	FUNCTION: (	Isoleucine--tF	59	1	46
Q6GDQ0	ATP-depende	clpL	FUNCTION:	ATP-depende	62	14	41
Q6GG85	Alanine--tRN/	alaS	FUNCTION: (	Alanine--tRN/	53	3	39
A7X1D8	Isoleucine--tF	ileS	FUNCTION: (	Isoleucine--tF	59	1	46
Q6GFV0	Putative dipe	SAR1836		Putative dipe	62	2	27
A0A8B0ZQE9	Probable quir	qoxA		Probable quir	64	23	23
A0A6B5EF64	Immunoglobu	spa		Immunoglobu	59	1	34
A0A7U8SPY4	Catalase	SAXG_02242		Catalase OS=	75	3	33
A0A7U9J3R4	Inosine-5'-mo	guaB		Inosine-5'-mo	73	32	32
Q6G669	Probable mal	mqo		Probable mal	76	25	32
A0A8D9ZQD	Putative mem	SHAG_01824		Putative mem	62	3	55
Q2UW15	Immunoglobu	spa		Immunoglobu	74	2	37
Q8NW23	Putative dipe	MW1694		Putative dipe	58	1	25
Q9RF06	Aspartyl/gluta	gatB	FUNCTION: /	Aspartyl/gluta	78	2	35
A0A2S6DGV	Glucose-6-ph	pgi		Glucose-6-ph	70	4	31
A0A6G4ISL2	ECM-binding	ebh		ECM-binding	8	7	74
A0A853PAE1	ATP-depende	A6762_0468(		ATP-depende	62	2	41
A8Z3T8	Elongation fa	tsf	FUNCTION: /	Elongation fa	76	5	24
A0A8B1CJD2	Mn(2+)-deper	sapep		Mn(2+)-deper	58	1	25
A0A8F8XLG(	Phosphopyru	eno		Phosphopyru	67	1	23
A0A8B1CJF8	30S ribosoma	rpsA		30S ribosoma	74	1	28
A0A8B0ZAM(	Alanine--tRN/	alaS		Alanine--tRN/	54	1	38
A0A0U1MJB(	Translation ir	infB	FUNCTION: (	Translation ir	56	33	33
Q6GG42	Valine--tRNA	valS	FUNCTION: (	Valine--tRNA	59	8	48
Q6GDG7	Arginine deim	arcA		Arginine deim	80	35	35
A0A5S9C5A4	Fructose-bisp	fda		Fructose-bisp	86	7	27
A0A7U8SQR	Dihydrolipoar	SAXG_01997		Dihydrolipoar	62	22	25
A0A8E0EHD3	Alkyl hydrope	SCAG_00861		Alkyl hydrope	72	2	29
A0A8B1CJZ6	Acetate kinas	ackA		Acetate kinas	72	1	22
Q5HGY8	Dihydrolipoyl	pdhD	FUNCTION:	Dihydrolipoyl	66	15	27
Q2FK94	Putative alde	aldA		Putative alde	73	2	28
A0A6B5G1X(	Succinate--Co	sucC		Succinate--Co	69	15	26
Q6GJB8	Putative pyric	SAR0555		Putative pyric	81	3	26
A0A2S6DCW	DNA repair p	CSC87_09015		DNA repair p	59	1	53
Q6GI15	Phosphoribos	purL	FUNCTION:	Phosphoribos	65	16	36
A0A6B0B0C6	Formate--tetr	fhs		Formate--tetr	63	12	25
A0A033UHR(	DNA-directec	rpoC	FUNCTION:	DNA-directec	32	1	42
A0A8D9ZCI2	DNA polymer	SAYG_00321		DNA polymer	59	42	42
A0A6B5DMS	Enolase	eno		Enolase OS=	67	1	23
A0A6N8IEG4	Immunoglobu	spa		Immunoglobu	67	1	36
A0A8D9ZLB(	D-lactate deh	SHAG_0030(		D-lactate deh	71	2	28
Q6GFF7	Glutamyl-tRN	gatA	FUNCTION: /	Glutamyl-tRN	64	21	23

A0A8D9Z5P4	Cell division p	SATG_02655		Cell division p	55	38	38
Q6GHD4	Aerobic glyce	glpD		Aerobic glyce	58	31	31
Q6GJB4	Probable brai	ilvE	FUNCTION: /	Probable brai	82	21	25
A0A8E0EDU	Uncharacteriz	SCAG_01312		Uncharacteriz	87	22	23
Q6GFR5	Phosphoenol	pckA	FUNCTION:	Phosphoenol	69	34	34
A1KE65	Immunoglobu	spa		Immunoglobu	85	1	35
A0A8D9ZME	N-acetylmura	SHAG_00429		N-acetylmura	75	38	43
A0A844QVN4	Formate deh	GO782_0933		Formate deh	49	1	32
H9BRN9	Immunoglobu	spa		Immunoglobu	61	1	35
A0A2S6D4J7	Glutamine--fr	glmS	FUNCTION:	Glutamine--fr	54	20	28
A0A0U1MI53	Pyruvate deh	pdhA	FUNCTION:	Pyruvate deh	73	3	19
A0A0H3K025	PBP2	pbp2		PBP2 OS=St	57	18	33
A0A8A7XGA	Aspartyl/gluta	gatB		Aspartyl/gluta	75	1	34
A0A8D9ZMD	Putative mem	SHAG_01710		Putative mem	64	14	40
A0A7R6SNW	D-lactate deh	SAJPND4_02		D-lactate deh	57	1	26
A0A6B5TE83	Alkyl hydrope	ahpF		Alkyl hydrope	68	2	28
A0A8B1CHM	Glutamate--tR	gltX		Glutamate--tR	69	27	31
A0A6B5H0R8	Cysteine synt	cysK		Cysteine synt	87	2	17
Q6GGG4	Probable glyc	gcvPB	FUNCTION:	Probable glyc	62	18	28
A0A6B5FYN	Glucose-6-ph	pgi		Glucose-6-ph	66	1	28
Q6GFZ0	Septation ring	ezrA	FUNCTION:	Septation ring	64	42	42
Q6GIN2	UvrABC syste	uvrA	FUNCTION:	UvrABC syste	54	43	43
Q6GJ61	Arginine--tRN	argS		Arginine--tRN	70	26	31
Q8NXZ0	Lysine--tRNA	lysS		Lysine--tRNA	64	1	33
A0A7Z8GCX	Catalase	E4U00_0434		Catalase OS=	71	1	31
P0A038	Glyceraldehy	gapA	FUNCTION:	Glyceraldehy	88	3	20
Q6GFZ8	Alanine dehy	ald2	FUNCTION:	Alanine dehy	75	1	24
A0A8E0EFZ2	Ornithine-oxo	SCAG_01301		Ornithine-oxo	67	1	22
P64225	Aminomethyl	gcvT	FUNCTION:	Aminomethyl	59	19	19
A0A8E0B1K4	Nitric oxide d	SCAG_00713		Nitric oxide d	73	8	25
Q53638	Lysine--tRNA	lysS		Lysine--tRNA	63	1	33
A0A0E1VPN	Fructose-bisp	fda		Fructose-bisp	68	2	20
A0A8D9YU89	Nitrate reduct	SASG_01162		Nitrate reduct	47	50	50
A0A2S6DSZ5	Alanine dehy	ald		Alanine dehy	73	1	23
A0A8D9YYN	Glycerol kina	SASG_00304		Glycerol kina	55	24	24
A0A2X2JUA8	L-threonine 3	NCTC7878_01177		L-threonine 3	75	24	24
A0A0D1JQ13	Putative pyric	QU38_09765		Putative pyric	71	1	24
A0A6B5HY64	Glutamate de	G0X69_03765		Glutamate de	70	8	25
P99075	Fructose-bisp	fba	FUNCTION:	Fructose-bisp	92	18	21
A0A8G2HYJ	Glycine dehy	gcvPA		Glycine dehy	63	21	23
A0A6G4IUV1	Pyruvate deh	pdhA		Pyruvate deh	73	3	19
A0A2S6DUA	Aldehyde deh	A6762_10755		Aldehyde deh	67	15	27
P63334	6-phosphoglu	gnd	FUNCTION:	6-phosphoglu	62	22	28
Q6GDS9	Fructose-1,6-	fbp		Fructose-1,6-	54	25	35
A0A850FXK9	Cysteine synt	cysK		Cysteine synt	86	1	16
A0A0E1X787	Asparagine--t	asnS		Asparagine--t	68	23	23
Q6GFU3	Leucine--tRN	leuS		Leucine--tRN	45	6	33
A0A033UHS	Elongation fa	tuf	FUNCTION:	Elongation fa	45	1	13
A0A8E0DRI0	Indolepyruvat	SHAG_00658		Indolepyruvat	64	6	23
A0A8D9ZQ2	Citrate (Si)-sy	SHAG_01669		Citrate (Si)-sy	65	21	21

A0A7H9C3A:	Thimet oligop	SA0759_007(		Thimet oligop	54	34	34
A0A6B5APV!	Phenylalanine	pheT		Phenylalanine	49	3	30
Q6GFF3	DNA ligase	ligA	FUNCTION:	DNA ligase C	57	38	38
A0A7U4ESV!	Aspartate--tR	aspS		Aspartate--tR	55	21	31
Q6GHG1	Polyribonucle	pnp	FUNCTION:	Polyribonucle	51	34	34
A0A8E0B1V4	Uncharacteri:	SCAG_0086(		Uncharacteri:	81	5	27
Q7WU24	Uncharacteri:	BN1321_460010		Uncharacteri:	67	33	33
A0A6B5PIT8	Lipoteichoic a	ltaS		Lipoteichoic a	46	15	22
D7RHA8	SpA IgG-bind			SpA IgG-bind	81	1	22
D7RHB1	SpA IgG-bind			SpA IgG-bind	75	1	19
A0A2X2JYP8	Formate acet	pflB_1		Formate acet	67	4	20
Q5HIQ6	GMP synthas	guaA	FUNCTION:	GMP synthas	63	10	25
A0A0E0VMB	SufBD_N dor	ST398NM01_0901		SufBD_N dor	60	18	26
A0A8E6CNC	Dihydrolipoyll	sucB		Dihydrolipoyll	47	12	21
A0A8D9ZLF1	SMC family, (	SHAG_0118		SMC family, (	39	44	46
Q2FGH0	Superoxide d	sodA	FUNCTION:	Superoxide d	93	16	17
A0A8D9ZLL4	Uncharacteri:	SHAG_0074		Uncharacteri:	62	37	37
A0A2X2KIF7	Valyl-tRNA sy	valS_1	FUNCTION:	Valyl-tRNA sy	62	1	37
A0A8F2XL11	Hyperosmola	ebh		Hyperosmola	6	1	55
A0A8E0DS9:	Nitrite reducta	SHAG_0019(		Nitrite reducta	53	35	38
A0A2S6D8D(	Proline--tRN/	proS	FUNCTION:	Proline--tRN/	60	27	27
Q5HGC3	Glutamine sy	glnA	FUNCTION:	Glutamine sy	70	19	23
Q6GKT6	Serine--tRNA	serS	FUNCTION:	Serine--tRNA	63	27	30
D2KNY6	LtrC-like prot	ltrC		LtrC-like prot	60	33	33
A0A8D9Z061	DNA segrega	SASG_0080!		DNA segrega	37	9	42
A0A7Z1S9R4	Trigger factor	tig		Trigger factor	53	20	20
A0A2S6DM2(	Ornithine ami	rocD2_2	FUNCTION:	Ornithine ami	59	1	19
A0A8D9ZIF9	Naphthoate s	SDAG_0098!		Naphthoate s	81	1	18
A0A380E0K8	Transketolas	tkt_3		Transketolas	72	1	22
A0A8E0EH2!	DNA gyrase,	SCAG_0054(		DNA gyrase,	38	30	36
A0A5S9I448	Alanine dehy	ald2		Alanine dehy	69	1	20
P0A0C1	Bifunctional /	aacA-aphD	FUNCTION:	Bifunctional /	68	30	30
A0A8B1CXP!	L-lactate deh	ldh		L-lactate deh	63	16	16
A0A8B1CZ1!	Acyl-CoA syn	acsA		Acyl-CoA syn	56	20	28
A0A8E0B13:	Phosphoenol	SCAG_0142!		Phosphoenol	42	24	26
A0A1Q8DD0	ATP synthase	atpD	FUNCTION:	ATP synthase	44	1	15
Q99R88	ATP-depende	clpL	FUNCTION:	ATP-depende	45	1	28
A6QEH1	Pyridoxal 5'-p	pdxS	FUNCTION:	Pyridoxal 5'-p	71	9	20
A0A7U8SUQ	SdrD protein	SFAG_0193(		SdrD protein	34	13	33
A0A8B1CG6(	ATP-depende	pcrA		ATP-depende	45	32	32
Q6GE15	Immunoglobu	sbi	FUNCTION:	Immunoglobu	66	32	32
A0A8B1CIG7	ATP-binding	JYA46_0007(		ATP-binding	52	25	25
Q2YXL1	Elongation fa	tsf	FUNCTION:	Elongation fa	62	2	21
Q6GG12	Isocitrate deh	icd		Isocitrate deh	62	12	23
A0A6A8F929	Leucine--tRN	leuS		Leucine--tRN	39	1	28
A0A8G2M9M	Phenylalanyl-	pheT_2		Phenylalanyl-	42	1	27
P67578	Methionine--t	metG	FUNCTION:	Methionine--t	61	13	32
A0A7Z1N66(	Oleate hydrat	CV021_0414		Oleate hydrat	57	1	30
A0A850G4M	50S ribosoma	rplC		50S ribosoma	60	2	15
Q6GAG7	1,4-dihydroxy	menB	FUNCTION:	1,4-dihydroxy	81	1	18

A0A380DHQ	Immunoglobu	spa_1		Immunoglobu	74	1	27
A0A8D9ZQK	Glycerophos	SHAG_0223		Glycerophos	65	21	21
P0A0K7	DNA gyrase s	gyrB	FUNCTION:	DNA gyrase s	53	26	34
A0A6B2IMU(	Fructose-bis	fda		Fructose-bis	69	1	21
A0A2Z4GSV	Glyceraldehy	gap		Glyceraldehy	87	1	17
A0A0E0VNP	Ribonuclease	rnj	FUNCTION:	Ribonuclease	53	27	27
A0A8E0B1M	Glycosyl trans	SCAG_0073		Glycosyl trans	58	5	28
A0A8F2XCR	NAD-depend	KRH49_0683		NAD-depend	67	27	27
Q6GHQ0	Cell division	ftsA	FUNCTION:	Cell division	58	24	24
A0A7Z8C9U	ECM-binding	ebh		ECM-binding	6	1	51
A0A6B5BU1	Phosphopent	deoB		Phosphopent	53	19	20
Q8NW24	D-alanine am	dat	FUNCTION:	D-alanine am	77	4	20
Q6GIB7	Coenzyme A	cdr	FUNCTION:	Coenzyme A	66	24	24
A0A8E0DRJ	S1 RNA bindi	SHAG_0250		S1 RNA bindi	53	34	34
A0A8D9YYI6	Triosephosph	SASG_0212		Triosephosph	77	1	19
A0A5S9C2Z	Oleate hydrat	TMSFP482_00620		Oleate hydrat	57	1	30
A5X5X2	DNA-directec	rpoB		DNA-directec	65	1	20
A0A6B5FUK	Penicillin-binc	G0Y65_07230		Penicillin-binc	53	3	36
Q6GHI9	Succinate--C	sucD	FUNCTION:	Succinate--C	75	2	15
A0A7Z8GCN	UvrABC syste	uvrB		UvrABC syste	55	40	40
A0A6B5HWD	Uncharacteri	G0Z03_10310		Uncharacteri	69	1	21
P99157	Alkaline shoc	asp23	FUNCTION:	Alkaline shoc	83	7	18
P67040	Phenylalanin	pheT		Phenylalanin	38	1	25
Q2YTD0	Putative univ	SAB1569		Putative univ	80	1	12
A0A6H4DXJ	Protein RecA	recA		Protein RecA	57	5	17
A0A133QBN	Signal peptid	HMPREF3211_00214		Signal peptid	65	1	20
Q9EZW7	Elongation fa	tuf		Elongation fa	61	1	14
Q2YUJ9	ATP synthase	atpA	FUNCTION:	ATP synthase	44	12	24
A0A8E0EFT	Polar amino	SCAG_0024		Polar amino	67	2	21
A0A8E0EFP	Phosphate ac	SCAG_0100		Phosphate ac	76	9	15
A0A1Q8DGS	Protein transl	secA	FUNCTION:	Protein transl	31	1	32
Q6GG08	ATP-depende	pfkA	FUNCTION:	ATP-depende	72	18	20
A0A8B1CG9	Universal stre	JYA46_0003		Universal stre	80	1	12
Q6GH30	Aminoacyltra	femB	FUNCTION:	Aminoacyltra	63	24	24
A0A5P6RAG	Glucose-6-ph	zwf		Glucose-6-ph	56	27	27
A0A5S9EVS	Putative urid	TMSFP482_20530		Putative urid	71	25	25
A0A380EFL3	Protein transl	fusA_1		Protein transl	74	1	12
A0A6B5BL61	NADP-depen	G0W76_12655		NADP-depen	58	2	20
Q2YYK9	50S ribosoma	rplE	FUNCTION:	50S ribosoma	80	16	16
A7WZS6	Phosphoglyc	pgk		Phosphoglyc	62	25	25
A0A7U9J4Y2	Thiamine pyr	SA1_118968		Thiamine pyr	49	4	23
A0A6N8I8X5	Fe-S cluster	sufD		Fe-S cluster	59	13	22
Q6GEC9	Putative 2-hy	SAR2389		Putative 2-hy	78	1	20
A6QI46	Glutamate-1-	hemL2		Glutamate-1-	69	21	21
Q6GEA4	Urocanate hy	hutU	FUNCTION:	Urocanate hy	56	28	28
A0A0D1K08	Amino acid A	CV021_14595		Amino acid A	63	1	20
Q6GFL5	Foldase prote	prsA	FUNCTION:	Foldase prote	61	31	31
A0A6B5IDL8	Tyrosine--tR	tyrS		Tyrosine--tR	64	20	23
A0A380DVX	Uncharacteri	NCTC6133_02088		Uncharacteri	76	13	19
A0A8E0EGT	Protoporphyri	SCAG_0222		Protoporphyri	59	5	22

A0A0U1MLT:	Penicillin-binc	pbpA		Penicillin-binc	52	1	34
A0A7U7EVP(	Nicotinate ph	SAR2006		Nicotinate ph	63	27	27
A0A8E0ED68	Peptidase	SCAG_0193		Peptidase OS	76	20	22
A0A5F0HIS2	Bifunctional p	folD	FUNCTION:	Bifunctional p	73	2	18
A0A8E0B2L7	Hydroxymeth	SCAG_0037(		Hydroxymeth	57	4	21
Q2G2D8	ABC transpor	SAOUHSC_00634		ABC transpor	62	2	23
A0A033V8H6	Cell division p	ftsZ	FUNCTION:	Cell division p	42	1	14
A0A8D9ZN78	FeS assembl	SHAG_0219(		FeS assembl	70	20	20
A0A6B5AQ2	50S ribosoma	typA		50S ribosoma	57	29	29
A0A6B0D0C	30S ribosoma	rpsB		30S ribosoma	77	17	20
A0A8E0DFL3	Ornithine carl	SASG_0017		Ornithine carl	67	2	16
A0A6G4N6U	DAK2 domair	G0Y31_1011		DAK2 domair	47	23	25
A0A8D9ZN1	Putative serir	SHAG_0170(		Putative serir	64	2	26
A0A8E0EQD	Hydrolase (H	SAZG_00531		Hydrolase (H	81	1	17
Q6GDH0	Carbamate ki	arcC2		Carbamate ki	67	22	22
A0A6G4IW65	NAD(P)/FAD-	G0X02_1056		NAD(P)/FAD-	65	21	21
A0A0D1JRF3	Delta-aminole	QU38_08615		Delta-aminole	65	2	18
A0A6B5I7K7	5'-nucleotidas	G0Y40_00340		5'-nucleotidas	56	2	16
Q7A8D1	Bleomycin re:	ble	FUNCTION:	Bleomycin re:	98	12	12
A0A384ZN40	Elongation fa	tufA		Elongation fa	82	1	12
A0A8B1CZ65	Porphobilinog	hemC		Porphobilinog	77	19	19
A0A6B0BXV	Bifunctional p	folD		Bifunctional p	73	0	17
A0A8B1CUW	2-hydroxyaci	JYA70_0023		2-hydroxyaci	81	1	20
A0A6B0BER	Transaldolas	GO793_13175		Transaldolas	86	1	19
A0A0A7LVG	Immunoglobu	spa		Immunoglobu	74	1	13
A0A0E1VTT1	Phosphometl	thiD		Phosphometl	67	14	14
A0A6B5IAN7	Pyruvate oxic	G0V76_01095		Pyruvate oxic	46	2	21
Q8NXX1	3-hexulose-6	MW0525	FUNCTION:	3-hexulose-6	87	13	13
A0A2S6D78	DNA transloc	sftA		DNA transloc	28	1	34
A0A7U8SRR	Probable mai	ppaC		Probable mai	61	19	19
Q2YUH3	DEAD-box A	cshA	FUNCTION:	DEAD-box A	52	10	22
A0A7R6SNF	Uncharacteri	SAJPND4_0		Uncharacteri	77	3	18
A0A8D9YUF	Helicase	SASG_0125		Helicase OS=	40	33	34
A0A8E0AWZ	D-alanine-D-a	SCAG_0245		D-alanine-D-a	61	19	21
Q9KWJ1	Protein A (Fra	spa		Protein A (Fra	47	1	13
A0A6B0BSM	Lactate dehy	GO793_15795		Lactate dehy	76	1	16
A0A8E0EDT	NADH dehyd	SCAG_0128		NADH dehyd	78	21	21
Q3T5M1	Mutant PBP2	mecA		Mutant PBP2	52	22	34
A0A5S9C4L5	Glucosamine	TMSFP482_17510		Glucosamine	80	3	18
A0A8G1GC3	Malate dehyd	mqo		Malate dehyd	59	1	26
A0A0H3JVA	Ribokinase	rbsK	FUNCTION:	Ribokinase C	64	16	16
A0A830YPB1	D-alanine am	dat		D-alanine am	72	1	17
A7WYW1	50S ribosoma	rplA	FUNCTION:	50S ribosoma	69	16	16
A0A0H3JNE	SA0182 prote	SA0182		SA0182 prote	43	1	18
A0A8E0DPE	Dynamin fam	SDAG_0141		Dynamin fam	29	30	30
A0A2S6DI83	Succinate--C	sucD	FUNCTION:	Succinate--C	70	1	14
A0A8D9ZMK	GTP-binding	SHAG_0083		GTP-binding	63	14	17
A0A0U1MQS	Adenylosucci	purB		Adenylosucci	64	28	28
A0A0D1HFZ	Glycosyl tran	QU38_14510		Glycosyl tran	51	1	24
A0A8E0DCH	Uncharacteri	SARG_0023		Uncharacteri	37	39	39

Q6GE66	Probable mal	mqo1		Probable mal	59	1	26
A0A8E0DR68	Alpha-glucosi	SHAG_01490		Alpha-glucosi	45	2	20
A0A7U4ERJ0	Uncharacteriz	M013TW_03		Uncharacteriz	62	1	20
Q5HDV8	50S ribosoma	rplC	FUNCTION:	50S ribosoma	62	1	14
Q6GIA3	3-oxoacyl-[ac	fabF	FUNCTION:	3-oxoacyl-[ac	51	15	15
A0A8F2LJS8	Lactonase fa	KQU62_0835		Lactonase fa	79	21	21
A0A6F9YXU0	Transaldolase	JICS137_253		Transaldolase	86	1	19
Q1HAN2	Clumping fac	clfA		Clumping fac	24	7	15
A0A0U1MUY	YhgE/Pip C-te	BN1321_430022		YhgE/Pip C-te	34	35	35
A0A380DX60	Hydroxymeth	mvaS		Hydroxymeth	61	3	20
Q6GFK5	Fumarate hyd	fumC	FUNCTION:	Fumarate hyd	41	15	15
A0A6B0BBR	Cof-type HAD	GO793_07295		Cof-type HAD	81	1	17
A0A641A525	NERD domai	D7S40_11660		NERD domai	75	22	22
A0A8D9WCV	Glutamate-1-	SADG_0169		Glutamate-1-	52	13	16
Q5HI63	Alcohol dehyd	adh		Alcohol dehyd	71	18	18
Q2FI15	Bifunctional p	folD	FUNCTION:	Bifunctional p	70	1	16
A0A6B5I245	Metal ABC tra	G0X69_11035		Metal ABC tra	60	1	22
A0A6B2IUZ7	3-oxoacyl-[ac	fabG		3-oxoacyl-[ac	68	11	13
A0A0U1MVU	Flavohemogl	YHB		Flavohemogl	51	1	18
Q2FZ27	GTP-sensing	codY	FUNCTION:	GTP-sensing	80	18	18
A0A2C9TSH9	D-alanine--D-	dltA	FUNCTION:	D-alanine--D-	42	17	17
A0A8E0AXI6	Aminopeptida	SCAG_02269		Aminopeptida	60	24	24
P65884	Adenylosucci	purA	FUNCTION:	Adenylosucci	67	24	24
A0A8G0IF57	Alpha-glucosi	JMO08_0016		Alpha-glucosi	43	2	20
A0A8E0EF00	Type-1 restric	SCAG_00677		Type-1 restric	35	1	31
A0A8F2LJP2	Proline dehyd	KQU62_0734		Proline dehyd	65	16	16
Q6GEX5	UDP-N-acety	murA1	FUNCTION:	UDP-N-acety	47	19	19
P60108	TelA-like prot	SA1238		TelA-like prot	69	27	27
A0A2X2JGY2	Cysteine--tRN	cysS		Cysteine--tRN	51	19	22
A0A8E0EME	Fibronectin-b	SIAG_01284		Fibronectin-b	32	19	25
A0A6B5BTN0	Serine protea	G0V17_07620		Serine protea	61	1	25
Q6GH60	Nuclease Sbc	sbcC	FUNCTION:	Nuclease Sbc	39	39	39
Q2FJN4	Alkyl hydrope	ahpC	FUNCTION:	Alkyl hydrope	87	5	13
A0A844RC09	Phosphogluc	glmM		Phosphogluc	53	21	21
A0A5F0HIX2	Ribonucleosi	nrdF		Ribonucleosi	65	17	17
A0A6B5CVP9	5'-nucleotidas	G0V17_05175		5'-nucleotidas	47	1	15
P66706	DNA-directed	rpoA	FUNCTION:	DNA-directed	66	19	19
Q6GDM0	Uncharacteriz	SAR2661		Uncharacteriz	69	17	17
Q5HHQ4	Thioredoxin r	trxB		Thioredoxin r	72	13	18
Q2FXA5	Coproporphyr	cgoX	FUNCTION:	Coproporphyr	50	2	19
M4M880	Immunoglobu	spa		Immunoglobu	56	1	19
A0A2S6D8P8	2-oxoacid fer	porA		2-oxoacid fer	40	20	20
A0A6B5I2Y0	Type I restric	G0X69_00605		Type I restric	35	1	29
A0A0E1X7Z4	Dihydroxyace	dhaL		Dihydroxyace	80	15	15
A0A8G0N5P0	AMP-binding	JMO10_0028		AMP-binding	56	25	25
A0A8D9YX02	DNA topoisom	SASG_00372		DNA topoisom	37	3	27
A0A1Q8DBS	Succinate de	BSZ10_11035		Succinate de	40	3	20
A0A1Q8DEL	Isoleucine--tR	ileS	FUNCTION:	Isoleucine--tR	29	1	21
A0A8B1CY08	Triosephosph	tpiA		Triosephosph	72	1	17
A0A6G4J790	Response reg	G0X15_0363		Response reg	75	6	20

A0A8D9YY72	Phosphoribos	SASG_00079		Phosphoribos	51	1	23
Q6GDG8	Ornithine carl	arcB	FUNCTION:	Ornithine carl	63	17	18
A0A0E1XAM	LPXTG-motif	HMPREF0769_10404		LPXTG-motif	44	5	24
A0A8E0EEJ7	Catabolite co	SCAG_02130		Catabolite co	71	21	21
A0A8G1GBR	NADP-depen	KSF82_1201		NADP-depen	51	1	19
Q5HGT9	Thioredoxin	trxA	FUNCTION:	Thioredoxin (	95	2	12
A0A454GY07	Transcription	rho	FUNCTION:	Transcription	65	29	29
A0A2S6DGE	UDP-N-acety	murC	FUNCTION:	UDP-N-acety	45	2	18
A0A380DRG	Probable mal	mqo1_2		Probable mal	56	1	20
A0A8E0B0E0	HAE1 family	SCAG_00086		HAE1 family	28	2	25
A0A0H3JTZ1	Transcription	nusA	FUNCTION:	Transcription	49	15	16
A1YZP1	Type I restric		FUNCTION:	Type I restric	33	1	29
A0A8E0B060	Transcription	SCAG_02555		Transcription	34	33	33
A0A8G1GBZ	Alpha-glucos	KSF82_1267		Alpha-glucos	31	13	16
A0A6B2IPB5	50S ribosoma	rplB		50S ribosoma	48	8	10
Q2YX50	Bifunctional p	purH		Bifunctional p	51	1	23
A0A8D9ZLU0	Succinate de	SHAG_01101		Succinate de	71	10	15
A0A8E0EFE3	Uncharacteri	SCAG_01673		Uncharacteri	73	2	19
A0A8F2LNH1	Elastin-bindin	ebpS		Elastin-bindin	46	8	22
A6U3U3	50S ribosoma	rplM	FUNCTION:	50S ribosoma	87	14	15
Q6GI62	Serine protea	SAR0992		Serine protea	41	31	31
A0A8E0AXA0	Uncharacteri	SCAG_02327		Uncharacteri	45	17	17
A0A6B5FQU	Cna B-type d	G0Y65_04285		Cna B-type d	35	28	28
A0A6G4QEK	Elongation fa	lepA		Elongation fa	47	22	22
A0A8F2XGH	Molybdopterii	KRH49_0959		Molybdopterii	55	18	18
A0A7R6PBR	Ornithine carl	SAJPND4_01		Ornithine carl	58	1	15
A0A8D9ZQL1	Oxidoreducta	SHAG_01930		Oxidoreducta	57	15	16
A0A8F2LN84	UDP-N-acety	murD		UDP-N-acety	55	22	22
A7X4V6	Uracil phosph	upp	FUNCTION:	Uracil phosph	84	15	15
A0A6G4N1I7	Beta sliding c	dnaN		Beta sliding c	54	19	19
A0A8B1CL85	Ribonuclease	rnj		Ribonuclease	46	24	24
A0A1Q8DEE	Ornithine ami	rocD	FUNCTION:	Ornithine ami	31	1	10
Q6GIC7	Argininosucci	argG		Argininosucci	51	21	21
A0A222UBE2	Protein RecA	recA	FUNCTION:	Protein RecA	45	1	13
A0A6B5LZM9	Type VII secr	esaA		Type VII secr	31	1	28
Q8NXH0	Probable cys	csd	FUNCTION:	Probable cys	64	19	19
A0A8G0IHL8	Efflux RND tr	JMO14_0024		Efflux RND tr	26	1	24
E0A8T2	ScaC (Fragm	scaD		ScaC (Fragm	70	2	9
Q6GIL5	2,3-bisphospl	gpmI	FUNCTION:	2,3-bisphospl	42	1	16
Q6GK53	Iron-sulfur clu	scdA	FUNCTION:	Iron-sulfur clu	66	13	14
A0A8D9ZKG	Uncharacteri	SHAG_00747		Uncharacteri	31	1	28
A0A8D9ZMC	Oligoendope	SHAG_01365		Oligoendope	43	24	24
A0A8G0ILD2	Serine hydro	JMO08_0022		Serine hydro	56	21	24
A0A8E0AXH3	ATP-binding	SCAG_02417		ATP-binding	43	1	27
Q6GJC8	50S ribosoma	rplL	FUNCTION:	50S ribosoma	88	10	10
A0A0A8IL53	Putative ATP	trsE		Putative ATP	39	25	25
A0A8G1GBV	FemA/FemB	fmhA		FemA/FemB	57	5	21
A0A0H3JMS9	Glucokinase	glcK		Glucokinase	64	11	14
A0A660A2T8	Maltodextrin-	cycB		Maltodextrin-	56	19	19
A0A1Q8DE60	Chaperone p	dnaK	FUNCTION:	Chaperone p	32	1	21

Q6GHR6	Carbamate ki	arcC1		Carbamate ki	58	5	17
O87364	ParM protein	BN1321_460004		ParM protein	60	20	20
Q6GET8	Deoxyribose-	deoC2	FUNCTION: (	Deoxyribose-	71	2	13
A0A2X2JXN(	Thioredoxin	trxA_1		Thioredoxin C	100	1	11
A0A8E0B2P(	PhnB protein	SCAG_00415		PhnB protein	95	1	13
A0A7U8SN3!	Lipase	SAXG_00813		Lipase OS=S	41	10	25
Q6GG60	GTPase Obg	obg	FUNCTION: /	GTPase Obg	59	11	24
Q6GDH2	Clumping fac	clfB	FUNCTION: (	Clumping fac	37	13	25
A0A8E0B1F9	tRNA:m(5)U-	SCAG_0159(		tRNA:m(5)U-	58	21	21
A0A8D9Z074	UDP-N-acety	SASG_02746		UDP-N-acety	49	15	15
Q6GEH2	Lipid II:glycin	femX	FUNCTION: (	Lipid II:glycin	58	23	23
A0A8E0AYJ2	Mercuric redu	SCAG_01007		Mercuric redu	56	6	18
Q6G8M6	Acetyl-coenzy	accD	FUNCTION: (	Acetyl-coenzy	64	17	17
Q5HDX9	Adenylate kin	adk	FUNCTION: (	Adenylate kin	86	21	21
A0A6A8FZ86	DNA topoisor	parC		DNA topoisor	37	1	25
A0A6G4N381	CTP synthas	G0Y31_0381		CTP synthas	51	24	24
A0A8E0AY48	Phenylalanyl-	SCAG_02136		Phenylalanyl-	86	16	16
A0A6B5CIE7	Delta-aminol	hemB		Delta-aminol	65	2	18
A0A8B1CJW	DNA polymer	polC		DNA polymer	26	3	36
O31211	UDP-N-acety	murC	FUNCTION: (	UDP-N-acety	38	1	17
A0A517JZ05	Multi-copper (	mco		Multi-copper (	47	24	24
Q6GEY2	Hydroxymeth	thiD	FUNCTION: (	Hydroxymeth	58	2	13
Q6GFZ4	Thiol peroxid	tpx	FUNCTION: T	Thiol peroxid	88	3	11
A0A2S6D518	tRNA-specific	mnmA	FUNCTION: (	tRNA-specific	55	18	18
P66552	30S ribosoma	rpsC	FUNCTION: I	30S ribosoma	69	20	20
A0A0U1MMI7	Putative FAD	ypdA		Putative FAD	64	15	15
Q2YTB5	ATP-depende	clpX	FUNCTION: /	ATP-depende	44	19	19
Q9Z4P5	MapN protein	mapN		MapN protein	39	18	30
A0A380EI98	Phosphoribos	purL_2	FUNCTION: I	Phosphoribos	45	1	20
A0A8G0YHD	Diaminopime	lysA		Diaminopime	51	17	17
A0A8E0EHD	Dihydroorota	SCAG_01539		Dihydroorota	50	17	17
T1YBR6	Phosphoeste	SAKOR_01799		Phosphoeste	55	18	18
A0A6B5HPP(	Biotin carbox	accC		Biotin carbox	65	22	22
A0A8E0B0E3	Molybdate AE	SCAG_00104		Molybdate AE	47	1	15
A0A8E0B1J1	Maltodextrin i	SCAG_00686		Maltodextrin i	45	17	17
A0A1Q8DF91	Dihydrolipoyl	BSZ10_04685		Dihydrolipoyl	32	1	13
A7X3V3	UPF0342 pro	SAHV_1830		UPF0342 pro	91	10	10
A0A8E0ASW	Fibronectin b	SAVG_00332		Fibronectin b	29	21	25
A0A8E0EEN	Phosphoman	SCAG_00317		Phosphoman	51	25	25
A0A8E0BJP4	Peptidase T-I	SIAG_00618		Peptidase T-I	51	16	16
Q6GKG7	Deoxyribose-	deoC1	FUNCTION: (	Deoxyribose-	64	2	13
A0A8D9ZMM	Putative lipop	SHAG_00862		Putative lipop	63	17	17
A0A6B5I5Y3	ABC-F type ri	abc-f		ABC-F type ri	40	1	26
A0A0E0VNB	Phosphate ac	plsX	FUNCTION: (	Phosphate ac	53	19	19
A0A8E0B1D4	2,3-bisphospl	SCAG_01191		2,3-bisphospl	42	1	16
Q6GFY8	30S ribosoma	rpsD	FUNCTION: (	30S ribosoma	78	13	13
A0A641A731	NAD(P)H-dep	D7S40_04480		NAD(P)H-dep	61	6	14
A0A8E0B159	Triacylglycerc	SCAG_00494		Triacylglycerc	42	24	27
A0A8B0Z620	Uncharacteriz	KAJ74_0097(		Uncharacteriz	73	2	19
A0A7Z8C5Z8	Uncharacteriz	E3A28_1387(		Uncharacteriz	43	1	23

A0A2S1XZ61	Enterotoxin C			Enterotoxin C	72	8	17
Q6GDB7	UPF0312 pro	SAR2769		UPF0312 pro	77	9	9
Q2YZB9	tRNA uridine	mnmG	FUNCTION:	tRNA uridine	46	27	27
A0A2S6DIM7	DNA-directed	CSC87_16030		DNA-directed	92	1	14
A0A0U1MML	Response reg	resD		Response reg	58	14	14
Q6GE17	2,3-bisphospl	gpmA	FUNCTION:	2,3-bisphospl	72	2	16
A0A8D9Z0W	DNA binding	SATG_00847		DNA binding	95	1	13
Q6GGC1	Chaperone p	dnaJ	FUNCTION:	Chaperone p	56	19	19
Q6GK57	Ribitol-5-phos	tarI1	FUNCTION:	Ribitol-5-phos	73	1	16
Q6GGY5	PTS system g	crr	FUNCTION:	PTS system g	63	8	8
A0A660A1C1	6-phosphoglu	FA040_01880		6-phosphoglu	56	16	16
A0A6B5JBL9	Nuclease Sb	GO690_03275		Nuclease Sb	40	27	27
A0A8E0DRB	Putative hydr	SHAG_02593		Putative hydr	66	3	15
A0A6B5EFL0	Methionyl-tRN	fmt		Methionyl-tRN	50	14	14
A0A380EGK8	Carbamate ki	arcC1_2		Carbamate ki	70	1	13
A0A0E1XBC9	NH(3)-depen	nadE	FUNCTION:	NH(3)-depen	51	4	14
A0A7U7EVV	UDP-GlcNAc	mnaA		UDP-GlcNAc	66	19	19
A0A0E0VSC4	Phytoene des	ST398NM01_2615		Phytoene des	42	16	16
A0A1Q8DFK	DNA-binding	BSZ10_03910		DNA-binding	61	1	15
A0A808JIJ7	RNA polymer	sigA		RNA polymer	49	21	21
A0A2S6D5B7	DUF1672 dor	CV021_08120		DUF1672 dor	65	12	21
A0A0E1X8L3	Sucrose-6-ph	HMPREF076	FUNCTION:	Sucrose-6-ph	45	19	19
A0A6B0ALN5	DNA polymer	polC		DNA polymer	23	1	34
A0A033V5H0	Succinate de	V070_00096		Succinate de	32	2	17
A0A8G2M73	Peptidoglycar	atl_1		Peptidoglycar	63	13	19
A0A5S9C444	DNA topoisor	parE		DNA topoisor	38	26	26
A0A7U8SW3	Ribose-phosp	prs		Ribose-phosp	51	15	15
A0A8G1GB5	ABC-F type ri	abc-f		ABC-F type ri	38	1	25
A0A8D9ZLR5	Betaine-aldeh	SHAG_00395		Betaine-aldeh	38	17	17
A0A8E0DGC	Undecapreny	SATG_00178		Undecapreny	53	16	16
A0A0E0VN59	Division spec	ST398NM01_1178		Division spec	31	21	21
A0A0U1MRF	Bifunctional h	thiD		Bifunctional h	54	1	12
A0A6B5IMF8	Putative expo	G0Y40_09320		Putative expo	36	2	20
A0A1Q8DE1	Aconitate hyd	BSZ10_0673	FUNCTION:	Aconitate hyd	21	1	15
A0A7U7ICM	Uncharacteri	SAR0248		Uncharacteri	45	15	22
A0A8G2CD9	Hypoxanthine	hpt		Hypoxanthine	75	14	14
Q2YV73	Ribitol-5-phos	tarI1	FUNCTION:	Ribitol-5-phos	73	1	16
P67277	Ribonuclease	rny	FUNCTION:	Ribonuclease	45	22	22
A0A8E0EGB	Uncharacteri	SCAG_02320		Uncharacteri	80	14	14
A0A7U8XPI8	ATP-depende	hslU		ATP-depende	40	12	19
A0A6B5AT40	PTS transpor	G0W76_08375		PTS transpor	30	13	14
Q2FE05	UTP--glucose	gtaB	FUNCTION:	UTP--glucose	69	15	15
A0A0E0VT22	Leukocidin S	ST398NM01_2471		Leukocidin S	57	14	14
A0A8G0N0D	Ribosome bio	der		Ribosome bio	44	6	15
A0A0H3KH34	Glutamyl-ami	NWMN_1638		Glutamyl-ami	63	14	14
A0A1Q8DB29	Threonine--tR	thrS	FUNCTION:	Threonine--tR	25	2	15
A0A8G0JA37	Aspartate car	JMO15_0012		Aspartate car	54	15	15
Q8GN49	Penicillin-binc	pbp2		Penicillin-binc	48	1	16
Q6GIM1	Epimerase fa	SAR0825		Epimerase fa	48	13	13
Q6GDV6	Uncharacteri	SAR2567		Uncharacteri	78	15	15

A0A6B5BTG(	Thiol peroxid:	tpx		Thiol peroxid:	88	1	9
A0A8B1CZ22	ABC-F family	JYA70_0012		ABC-F family	38	23	23
A0A7U3XIT7	Serine/threon	pknB		Serine/threon	38	23	23
A0A0U1MXH	Acetoacetyl-C	yqeF		Acetoacetyl-C	42	10	12
Q6GI10	Phosphoribos	purD		Phosphoribos	48	20	20
A0A454H1W	L-lactate deh	ldh	FUNCTION:	L-lactate deh	59	15	15
A0A1Q8DEA	Probable glyc	gcvPB	FUNCTION:	Probable glyc	30	1	11
Q6GGW9	Alanine dehy	ald1	FUNCTION:	Alanine dehy	56	17	17
A0A2S6DKC	Elongation fa	fusA	FUNCTION:	Elongation fa	15	1	10
A0A0U1MSA	Molybdate AE	modA		Molybdate AE	45	1	15
Q6GCZ8	Diacetyl redu	butA	FUNCTION:	Diacetyl redu	64	11	11
A7WZR9	ATP-depende	clpP	FUNCTION:	ATP-depende	46	4	10
A0A8E0DPT	Dehydrosqua	SHAG_0034		Dehydrosqua	47	18	18
A0A380EP58	Aminoacyltra	fmhA		Aminoacyltra	45	1	17
Q6GGU0	Demethylmer	menG	FUNCTION:	Demethylmer	66	14	14
A0A8E0AXA	UDP-N-acety	SCAG_0228		UDP-N-acety	48	4	19
A0A7Z1MZ3(	DNA-directec	CV021_1380		DNA-directec	64	2	12
Q2FFZ9	UPF0478 pro	SAUSA300_1685		UPF0478 pro	67	5	10
A0A8D9ZKJ(	DNA protecti	SHAG_0258		DNA protecti	90	8	8
A0A8D9YUR	DNA topoisor	SASG_0101(		DNA topoisor	37	23	25
Q6GH06	Cold shock p	cspA	FUNCTION:	Cold shock p	80	3	6
A0A0U1MUD	LPXTG-motif	BN1321_430113		LPXTG-motif	35	1	20
A0A8E0AYS	UDP-N-acety	SCAG_0245		UDP-N-acety	42	15	15
A0A8E0DSY	ABC transpor	SHAG_0218(		ABC transpor	67	1	18
Q6GER8	Mannitol-1-pł	mtlD		Mannitol-1-pł	55	15	15
A0A0U1MRV	Formimidoylg	hutG	FUNCTION:	Formimidoylg	62	2	14
A0A8D9ZN4	Fibronectin/fil	SHAG_0116(		Fibronectin/fil	47	22	22
A0A8D9YUY	Chromosome	SASG_0092		Chromosome	60	18	18
A0A380E1Z2	Ribonucleotic	nrdE_1		Ribonucleotic	58	1	11
A0A8A7XKB	ABC transpor	H7684_0499(		ABC transpor	40	1	19
A0A380E161	Dihydrolipoar	odhB_1		Dihydrolipoar	43	2	11
A0A8E0AYZ	N-acetylgluco	SCAG_0111		N-acetylgluco	48	15	15
A0A0E0VL93	Putative endo	ST398NM01_0530		Putative endo	36	7	7
D3X7W4	Amidohydrol:	BN1321_40004		Amidohydrol:	53	10	14
Q6GDN1	Probable trar	isaA	FUNCTION:	Probable trar	40	5	5
A0A1Q8DD1	ATP synthase	atpA	FUNCTION:	ATP synthase	26	1	13
P0A095	Transcription	nusG	FUNCTION:	Transcription	73	12	13
Q2FHI8	DNA topoisor	topA	FUNCTION:	DNA topoisor	35	22	22
Q6GFG4	Bacterial non	ftnA	FUNCTION:	Bacterial non	66	12	12
A0A380DW5	Alanyl-tRNA	alaS_1		Alanyl-tRNA	67	1	17
A0A6B5Q1X	Staphylococc	spa		Staphylococc	48	1	11
Q6GJR0	UPF0355 pro	SAR0405		UPF0355 pro	91	9	9
A0A8E0EGQ	Uncharacteri:	SCAG_0214(		Uncharacteri:	61	15	15
A0A8B1CH3	Replication-a	JYA46_0004		Replication-a	59	20	20
A0A8D9ZN7(	Signal recogr	SHAG_0119(		Signal recogr	42	18	19
A0A6B5EYM	Gfo/Idh/MocA	G0Z49_02230		Gfo/Idh/MocA	45	3	14
A0A8E0AYR(	Dihydroxyace	SCAG_0106		Dihydroxyace	40	14	14
A0A0U1MQY	Peptide chair	prfA	FUNCTION:	Peptide chair	48	15	18
A0A0H2X0L	Peptide ABC	SACOL2476		Peptide ABC	48	26	26
A0A6B5LUK	L-threonine d	tdcB		L-threonine d	48	12	12

A0A7U7EYU	Aminoacyltra	SAI7S6_100		Aminoacyltra	51	19	19
A0A8E0DS5	Iron compour	SHAG_0006		Iron compour	55	2	16
A0A5F0HGU	3'-5' exonucle	dinG	FUNCTION:	3'-5' exonucle	27	25	25
A0A6A8FX4	HPr kinase/pl	hprK		HPr kinase/pl	62	15	15
Q2FWY6	Uncharacteri	SAOUHSC_02121		Uncharacteri	53	22	22
A0A8G2I0R5	Phosphoglyc	pgm_2		Phosphoglyc	64	1	15
A0A0D1I6T9	RNA methyltr	QU38_13645		RNA methyltr	51	16	16
A0A6B5HT9	Hypoxanthine	purR		Hypoxanthine	57	12	12
A0A6K4C1E	Cadmium tra	cadA		Cadmium tra	26	18	18
A0A6B5AND	Glycerol-3-ph	gpsA		Glycerol-3-ph	43	14	14
A0A1Q8DEI	Formate--tetr	fhs		Formate--tetr	23	1	14
A0A2X2K097	Thiamine-pho	thiE	FUNCTION:	Thiamine-pho	71	11	11
A0A8E1Z4K	Uncharacteri	GZ156_0885		Uncharacteri	72	1	18
Q599H5	RsbU protein	rsbU		RsbU protein	57	18	18
Q2G0L7	FMN-depend	azo1	FUNCTION:	FMN-depend	74	8	8
Q6GER9	Mannitol-spe	mtlF	FUNCTION:	Mannitol-spe	92	10	10
A0A7Z8CAI8	Serine-protei	sigB		Serine-protei	42	9	18
A0A7U8SNQ	DNA repair p	SAXG_0115		DNA repair p	39	18	19
A0A1Q8DD8	GMP synthas	guaA	FUNCTION:	GMP synthas	30	1	13
A0A8B0ZEL	Uridylate kina	pyrH		Uridylate kina	53	11	11
Q6G990	DNA-binding	hup	FUNCTION:	DNA-binding	83	2	9
A0A6G4ITH4	UDP-N-acety	murE		UDP-N-acety	45	19	19
A0A8B1CYC	ABC transpo	JYA70_0013		ABC transpo	40	1	19
A0A7Z8FTF	Glyceraldehy	E3K14_0850		Glyceraldehy	55	15	15
A0A0E0VRW	Respiratory n	ST398NM01_2449		Respiratory n	43	17	17
A0A8E0AX9	Aldehyde deh	SCAG_0231		Aldehyde deh	41	11	14
Q6GDK4	3-methyl-2-o	panB	FUNCTION:	3-methyl-2-o	65	13	13
Q2FG96	Histidine--tR	hisS		Histidine--tR	41	17	17
A0A6N3ARR	Putative 3-hy	fadN		Putative 3-hy	35	1	25
A0A7U8SCX	3-hydroxyacy	SAXG_0072		3-hydroxyacy	37	2	26
Q2FY01	PhoH domair	SAOUHSC_01673		PhoH domair	63	15	15
A0A7U3XGG	Zinc-binding	HUW54_012		Zinc-binding	40	8	11
T1Y7U0	Lipoprotein	SAKOR_00820		Lipoprotein C	67	1	18
A0A033UUM	GMP synthas	guaA	FUNCTION:	GMP synthas	31	1	12
Q6GFM4	Coproporphy	cpfC	FUNCTION:	Coproporphy	59	2	15
Q2FHH9	Ribosome-re	frr	FUNCTION:	Ribosome-re	83	1	15
A0A7U7EU9	Site-specific	SAR0433		Site-specific	50	21	21
A0A380EFH	NAD-specific	gluD_2		NAD-specific	93	1	11
A0A7U7ID83	Putative com	cinA		Putative com	54	17	17
A0A6B5I1E6	Lipoyl syntha	lipA		Lipoyl syntha	46	14	14
A0A141HMM	DNA topoisor	pGO400_p12		DNA topoisor	36	25	25
A0A0E1VJQ	Uncharacteri	HMPREF0776_1618		Uncharacteri	61	2	10
A0A5S9I4G4	Multifunction	secD	FUNCTION:	Multifunction	25	17	17
A0A033UZD	Chaperone p	dnaK	FUNCTION:	Chaperone p	19	3	14
Q6GG00	UPF0173 me	SAR1785		UPF0173 me	71	10	10
A0A8D9ZQR	NADH-depen	SHAG_0223		NADH-depen	43	14	14
A0A1Q8DCQ	DEAD-box A	cshA	FUNCTION:	DEAD-box A	33	2	14
A0A8E0B0W	Uncharacteri	SCAG_0035		Uncharacteri	56	1	12
A0A8G2I1Z2	NH(3)-depen	nadE		NH(3)-depen	48	1	11
A0A6B5EIL1	Type IV secre	G0Y58_05120		Type IV secre	37	18	18

Q6GGH6	Exodeoxyribo	xseB	FUNCTION:	Exodeoxyribo	84	9	9
A0A7R6P4K6	IolE protein-li	SAJPND4_0(		IolE protein-li	35	8	8
A0A6B0BDT1	Aldehyde deh	GO793_09370		Aldehyde deh	60	1	13
Q7A1J9	HTH-type tra	mgrA	FUNCTION:	HTH-type tra	67	11	11
A0A0D1JK82	Cytosol amin	QU38_11325	FUNCTION:	Cytosol amin	37	3	15
A0A830YSG3	ATP synthase	atpG		ATP synthase	50	11	11
A0A2X2K7G3	EIIA	fruA_2		EIIA OS=Sta	26	12	13
A0A6B5CTH0	Ribosome-re	frr		Ribosome-re	82	1	15
A0A1Q8DGS	Ribonucleosi	BSZ10_0165	FUNCTION:	Ribonucleosi	23	1	14
A0A831EEE0	Transcription	lytR		Transcription	60	17	17
A0A8E0EGQ	Alcohol dehy	SCAG_0020		Alcohol dehy	58	2	15
A0A8E0B3Q7	Oxidoreducta	SCAG_0069		Oxidoreducta	53	16	16
Q6GI16	Phosphoribos	purQ	FUNCTION:	Phosphoribos	77	12	12
Q6GH18	Phosphate-bi	pstS	FUNCTION:	Phosphate-bi	42	2	12
A0A8B1CYH5	Phenol-solub	pmtC		Phenol-solub	55	1	15
A0A2I7Y7Y9	Dihydrolipoar	merA_1		Dihydrolipoar	40	1	13
A0A831E416	Secretory ant	ssaA2		Secretory ant	51	1	8
A0A7Z8G8Q4	EVE domain-	E4U00_1191		EVE domain-	41	14	14
A7X0K2	3-oxoacyl-[ac	fabH	FUNCTION:	3-oxoacyl-[ac	55	1	12
A0A6B2IQS1	3-deoxy-7-ph	aroF		3-deoxy-7-ph	43	2	15
A0A1Q8DF48	Elongation fa	tsf	FUNCTION:	Elongation fa	29	1	11
A0A8B1CFW	Dihydroptero	folP		Dihydroptero	58	15	15
A0A1Q8DEE	Glucose-6-ph	pgi		Glucose-6-ph	34	1	11
Q6GAQ7	Peptide chair	prfC	FUNCTION:	Peptide chair	34	16	16
Q5HFH9	Protein GrpE	grpE	FUNCTION:	Protein GrpE	59	1	11
A0A0B6XQ09	Phage capsic	BTN44_11395		Phage capsic	40	12	12
A0A0U1MPM	Lipid II isoglu	murT		Lipid II isoglu	41	1	16
A0A7U3XK49	Bifunctional (	HUW54_082		Bifunctional (	33	20	26
Q6GKU4	Chromosoma	dnaA	FUNCTION:	Chromosoma	53	14	24
A0A7U4AUY0	Phenol-solub	pmtC		Phenol-solub	55	1	15
A0A8D9ZMC	Anaerobic rib	SHAG_0039		Anaerobic rib	35	24	24
Q2FWB8	Purine nucleo	deoD		Purine nucleo	60	10	10
A0A6B5DC23	Imidazolonep	hutI		Imidazolonep	40	15	15
A0A8E0ELC9	Uncharacteri	SAZG_0044		Uncharacteri	56	16	16
A0A8G2M88	DNA polymer	dnaX		DNA polymer	38	19	19
P64284	Transcription	greA	FUNCTION:	Transcription	74	11	11
Q2G2F0	HIT domain-c	SAOUHSC_01968		HIT domain-c	72	10	10
A0A2X2K123	Hydroxyethyl	thiM	FUNCTION:	Hydroxyethyl	50	9	9
A0A8E0EHH	2-oxoglutarat	SCAG_0162		2-oxoglutarat	65	9	12
A0A1Q8DAY	Glutamine--fr	glmS	FUNCTION:	Glutamine--fr	18	1	9
A0A8E0AZS5	Signal recogr	SCAG_0157		Signal recogr	39	14	14
A0A0E1VNK9	30S ribosoma	rpsS	FUNCTION:	30S ribosoma	65	7	10
A0A7Z8FRT8	Probable dua	rlmN		Probable dua	45	14	14
A0A6B5FKT6	Elongation fa	efp		Elongation fa	70	11	11
Q5TKS1	Beta-ketoacy	fabH		Beta-ketoacy	57	1	12
A0A033V427	Aconitate hyc	V070_00405	FUNCTION:	Aconitate hyc	19	1	12
A0A8F7RLC3	Carbamoyl pl	KX797_0876		Carbamoyl pl	37	12	12
A0A1Q8DC0	Transaldolase	BSZ10_10645		Transaldolase	50	1	11
A0A7R6PD94	Aminopyrimic	SAJPND4_02		Aminopyrimic	51	10	12
A0A6G4IS94	NAD-depend	G0X02_0367		NAD-depend	36	4	16

O87361	MobA/MobL f	nes		MobA/MobL f	36	27	27
Q7A6I1	Putative pept	SA0815	FUNCTION:	Putative pept	65	9	10
A0A8E0AZF2	MutS2 protei	SCAG_01484		MutS2 protei	26	14	19
Q6GGB9	Protein GrpE	grpE	FUNCTION:	Protein GrpE	63	1	11
A0A8E0EDD	6,7-dimethyl-	SCAG_02159		6,7-dimethyl-	74	1	8
A0A380DV31	Cytosolic pro	ywpJ_2		Cytosolic pro	58	1	13
A0A133PZR9	Protein/nucle	hchA	FUNCTION:	Protein/nucle	49	2	13
Q6GHE0	DNA mismatc	mutS	FUNCTION:	DNA mismatc	30	23	23
A0A0H3K6Z5	Protein DltD	dltD		Protein DltD (	39	15	15
A0A6B5EEQ	Cytidylate kin	cmk		Cytidylate kin	61	11	13
A0A8D9Z430	Uncharacteri	SATG_01615		Uncharacteri	56	9	9
A0A5F0HM9	Triacylglycer	lip2		Triacylglycer	26	2	17
Q5HJN5	Superoxide d	sodM	FUNCTION:	Superoxide d	69	3	12
Q2FW21	50S ribosoma	rplF	FUNCTION:	50S ribosoma	54	3	9
P66580	30S ribosoma	rpsE	FUNCTION:	30S ribosoma	75	11	15
A0A1Q8DDX	30S ribosoma	BSZ10_06905		30S ribosoma	22	1	12
Q6GFI2	Uncharacteri	SAR1965		Uncharacteri	81	1	8
A0A6B5GZ6	50S ribosoma	rplY		50S ribosoma	35	3	6
A0A5F0HHE	Putative phos	E3A28_03650		Putative phos	61	9	9
A0A8E0BH8	Plasmid reco	SIAG_01537		Plasmid reco	41	21	21
A0A6B5F3F6	Glutamate ra	racE		Glutamate ra	74	1	15
A0A6B5CY51	ABC transpo	G0V17_00590		ABC transpo	53	1	15
A0A8E0B1R7	Sulfite reduct	SCAG_00442		Sulfite reduct	36	19	19
P0A0D0	Spectinomyci	ant1	FUNCTION:	Spectinomyci	61	15	15
Q6GFR6	S-adenosylm	metK	FUNCTION:	S-adenosylm	51	15	15
A0A8E0DFJ6	Peptidase T	SASG_02091		Peptidase T (	36	1	13
A0A6B5CFQ	Methionine a	map		Methionine a	49	10	10
Q2FC58	Beta-lactama	blaZ		Beta-lactama	60	1	13
A0A8B1CM6	PH domain-c	JYA66_00230		PH domain-c	69	1	7
Q6GJH3	Putative sept	spoVG	FUNCTION:	Putative sept	89	10	10
A7X1E5	Bifunctional p	pyrR	FUNCTION:	Bifunctional p	69	12	12
A0A6B5HZR	3-deoxy-7-ph	aroF		3-deoxy-7-ph	42	1	14
Q6GGE2	Probable end	nfo	FUNCTION:	Probable end	53	16	16
A0A8D9ZNC	Phosphoribos	SHAG_01018		Phosphoribos	85	15	18
Q6GHT5	Glutamate ra	murI	FUNCTION:	Glutamate ra	77	1	15
A0A6B2IR18	Restriction er	GZ116_08500		Restriction er	54	13	13
A0A380E4I1	Queuine tRN	tgt	FUNCTION:	Queuine tRN	50	15	15
A0A2D1GVD	Conserved vi	cvfB		Conserved vi	53	16	16
A0A2Y1TBB7	Beta-lactama	blaZ		Beta-lactama	43	1	15
A0A033UQ5	ATP synthase	atpA	FUNCTION:	ATP synthase	23	1	12
A0A8G2M8U	DegV family	NCTC7972_(		DegV family	38	11	11
Q6GIR8	Histidinol-pho	hisC		Histidinol-pho	35	11	11
Q6GI19	N5-carboxya	purK	FUNCTION:	N5-carboxya	44	3	14
A0A0E1VJD9	Transketolas	HMPREF0776_2552		Transketolas	34	10	10
A0A380DMP	Ornithine--ox	rocD2_3		Ornithine--ox	55	2	10
A0A7Z8C7K	Protein/nucle	hchA		Protein/nucle	47	1	12
A0A7U7ICE5	Uncharacteri	SAR0673		Uncharacteri	53	12	12
A0A8G2M9J	Malonyl-CoA-	fabD		Malonyl-CoA-	47	11	11
Q6G9R7	DNA mismatc	mutL	FUNCTION:	DNA mismatc	27	1	17
A0A8G0N7J9	Insulinase fa	JMO11_0013		Insulinase fa	44	16	16

Q2FXV2	Uncharacteri:	SAOUHSC_01729		Uncharacteri:	77	9	9
A0A033UK35	Pyridoxal 5'-p	pdxS	FUNCTION:	Pyridoxal 5'-p	37	1	12
A0A8E0B0C4	Glycosyl trans	SCAG_01805		Glycosyl trans	52	15	15
A0A8E0DF75	Alcohol dehyd	SATG_00626		Alcohol dehyd	55	1	14
Q6GAW6	Argininosucci	argH		Argininosucci	38	16	16
P68825	Peptide defor	def	FUNCTION:	Peptide defor	62	11	11
A0A8E0DCT5	Replication pr	SASG_01695		Replication pr	65	11	12
P66356	30S ribosoma	rpsK	FUNCTION:	30S ribosoma	65	5	8
A0A7U4ATJ8	Riboflavin bio	ribF		Riboflavin bio	62	16	16
A0A8D9ZN60	Branched-cha	SHAG_01501		Branched-cha	40	17	18
A0A8E0AZ12	D-isomer spe	SCAG_01274		D-isomer spe	36	3	10
A0A8E0AZH3	Lipoate-prote	SCAG_01370		Lipoate-prote	40	7	10
A0A8G2CDE	Vitamin B12-l	btuF		Vitamin B12-l	52	12	14
P67199	Putative pyru	SAV1563	FUNCTION:	Putative pyru	56	13	13
A0A6B0CUV	DNA mismatc	mutL		DNA mismatc	27	1	17
A0A7R6P114	Homoserine d	SAJPND4_01		Homoserine d	30	14	14
A0A0E1XBD0	DUF1801 dor	HMPREF0769_10696		DUF1801 dor	65	13	13
Q6GFE2	Nitric oxide sy	nos	FUNCTION:	Nitric oxide sy	52	17	17
A0A6B5ANK0	Exodeoxyribo	xseA		Exodeoxyribo	40	17	17
A0A380EHQ3	Thymidylate s	thyA	FUNCTION:	Thymidylate s	57	16	16
A0A8E0EG01	Peptide/nicke	SCAG_01331		Peptide/nicke	25	14	14
A0A8E0AYM	Mevalonate k	SCAG_01003		Mevalonate k	64	14	15
A0A0E0VP30	Oxidoreducta	ST398NM01_1566		Oxidoreducta	36	2	11
A0A0H3K4N2	Abhydrolase_	MW2456		Abhydrolase_	59	1	12
Q6GEI4	50S ribosoma	rplD	FUNCTION:	50S ribosoma	67	10	10
A0A8D9ZLQ2	Acetyltransfe	SHAG_02571		Acetyltransfe	55	15	15
A0A0U1MEX	6-phospho-3-	hxlB		6-phospho-3-	64	8	8
A0A8E0EHU	Short chain d	SCAG_00154		Short chain d	57	11	11
Q6GIQ3	UDP-N-acety	murB	FUNCTION:	UDP-N-acety	60	15	15
Q6GDP0	Copper chape	copZ	FUNCTION:	Copper chape	100	6	6
A0A8E0AYA2	Uncharacteri:	SCAG_02211		Uncharacteri:	33	17	17
A0A1Q8DF31	Succinate--C	sucC	FUNCTION:	Succinate--C	29	1	11
A0A8E0AYG	Glycosyl trans	SCAG_00976		Glycosyl trans	34	15	15
Q6GHG4	tRNA pseudo	truB	FUNCTION:	tRNA pseudo	62	2	17
Q5HEU2	Uroporphyrin	hemE	FUNCTION:	Uroporphyrin	49	12	12
A0A5S9C4T9	Probable DNA	rpoE	FUNCTION:	Probable DNA	56	8	8
A0A0E1VR62	Pseudouridin	HMPREF0776_2518		Pseudouridin	58	6	13
A0A8E0EFN3	Sodium trans	SCAG_00170		Sodium trans	55	15	15
A0A8D9ZL48	SAM binding	SHAG_01031		SAM binding	34	11	11
A0A8E0EHA1	Cobalt/nickel	SCAG_01419		Cobalt/nickel	33	15	15
A0A7U4AS95	Protein-argini	mcsB		Protein-argini	51	13	13
A0A8D9ZP28	MutT/NUDIX	SHAG_01652		MutT/NUDIX	65	10	13
Q2YUJ7	ATP synthase	atpF	FUNCTION:	ATP synthase	52	8	12
Q6GI14	Amidophosph	purF	FUNCTION:	Amidophosph	31	15	15
A0A8D9ZPM	Biotin-[acetyl-	SHAG_01442		Biotin-[acetyl-	45	12	12
A0A8E0EFV7	Ribonuclease	SCAG_01196		Ribonuclease	30	22	22
A0A8E0EG89	ABC-2 type tr	SCAG_00830		ABC-2 type tr	45	10	10
A0A6G4Q1C	Universal stre	G0Y27_0361		Universal stre	63	9	9
A0A8E0DSX8	RNAIII-activa	SHAG_01816		RNAIII-activa	80	10	10
A0A8G2HWF	Elastin bindin	ebpS		Elastin bindin	27	1	15

A0A1Q8DFA	Alpha-ketoac	BSZ10_04695		Alpha-ketoac	26	1	9
A0A8E0AZZ8	Uncharacteri:	SCAG_0175€		Uncharacteri:	45	9	9
A0A2S6DA08	Cell division p	CSC87_17520		Cell division p	81	1	6
A0A7U8SQT	ATP-depende	addA		ATP-depende	22	26	26
A0A6B0CKP:	Orotidine 5'-p	pyrF		Orotidine 5'-p	61	11	11
A0A380EKW	GTPase Era	bex	FUNCTION: ,	GTPase Era	49	14	14
Q7A079	50S ribosoma	rplV	FUNCTION: `	50S ribosoma	66	8	8
A0A7R6SMP	Uncharacteri:	SAJPND4_01		Uncharacteri:	58	8	8
A0A8D9T127	Tryptophanyl-	SAEG_00921		Tryptophanyl-	40	2	13
A0A7U4ASS:	MSCRAMM f	clfA		MSCRAMM f	12	1	9
A0A1Q8DDK	Methionine--t	metG	FUNCTION:	Methionine--t	25	1	15
A0A8G0MV7	Beta-lactam s	blaR1		Beta-lactam s	23	11	16
A0A8B1CJN:	Uncharacteri:	JYA46_0013:		Uncharacteri:	91	8	8
A0A033V0Q(	UPF0051 pro	V070_01009		UPF0051 pro	24	2	10
Q2YSV9	Transcription	sarA	FUNCTION: (	Transcription	65	1	12
Q6GCA7	30S ribosoma	rpsF	FUNCTION: I	30S ribosoma	78	10	10
Q6GIR5	Putative lipid	SAR0780	FUNCTION: I	Putative lipid	45	10	10
A0A8B1CI80	FAD-depende	JYA46_0024:		FAD-depende	43	13	13
A0A8G2I0H8	tRNA-i(6)A37	miaB_2		tRNA-i(6)A37	30	16	16
A0A229LSU3	Exported prot	A6762_02950		Exported prot	60	1	9
A0A6B5I2N6	DHOdehase	G0X69_10205		DHOdehase	43	13	13
A0A8D9ZPG	Peptidase, M	SHAG_0134€		Peptidase, M	54	16	16
A0A5S9I385	Enoyl-[acyl-ca	fabI		Enoyl-[acyl-ca	48	10	10
A0A5F0HMT	Beta-channel	E3A28_12350		Beta-channel	48	11	16
Q201G4	Ferrochelatas	hemH	FUNCTION: (	Ferrochelatas	60	1	14
A0A0U1MHV	N5-carboxyar	purK	FUNCTION: (	N5-carboxyar	42	1	12
A0A7U7IF63	Erythromycin	ermA		Erythromycin	46	12	12
A0A8E0EFL4	Acyl-CoA deh	SCAG_0011(		Acyl-CoA deh	49	17	17
A0A1Q8DDC	Alkyl hydrope	BSZ10_08350		Alkyl hydrope	24	1	9
A0A6B5LZL3	Leucyl amino	G0V76_06430		Leucyl amino	34	1	13
A0A8E0BD7:	Sensor protei	SAZG_0021:		Sensor protei	32	20	20
A0A8B1CN8!	ATP-depende	addB		ATP-depende	19	20	20
A0A8E0B2A2	CDP-glycerol	SCAG_0072		CDP-glycerol	40	14	14
A0A6B5DCR	Class I SAM-	G0X68_01380		Class I SAM-	75	13	13
A0A7Z8FV25	Immunodomi	isaB		Immunodomi	39	9	9
A0A7U4ATS(	Insulinase far	M013TW_12		Insulinase far	35	17	17
A0A8D9ZMV	Type I restric	SHAG_0090:		Type I restric	34	10	15
A0A0U1MSC	Uncharacteri:	BN1321_400017		Uncharacteri:	53	8	8
A0A8D9ZJM	Hydroxymeth	SHAG_0032		Hydroxymeth	38	14	14
A6QF73	Nucleotide-bi	NWMN_073:	FUNCTION: I	Nucleotide-bi	48	13	17
A0A8D9ZLN(	HAD-superfa	SHAG_0131:		HAD-superfa	41	4	8
A0A6B5CWE	6,7-dimethyl-	ribH		6,7-dimethyl-	74	1	8
A0A2X2LWR	Isochorismat	entC		Isochorismat	40	15	17
A0A0E1XB7(	D-lactate deh	HMPREF0769_10589		D-lactate deh	39	14	14
A0A8E0B2E2	Uncharacteri:	SCAG_0177€		Uncharacteri:	87	7	7
A0A7U4ERX	CDP-glycerol	M013TW_02		CDP-glycerol	36	10	17
A0A1D4Q69(	NADH-depen	ahpC_2		NADH-depen	84	1	8
A0A6A9GUI5	TIGR00282 fa	GAY51_07510		TIGR00282 fa	51	12	12
Q6GER3	Arginase	arg		Arginase OS=	51	15	15
Q2YUG0	tRNA N6-ade	tsaD	FUNCTION: I	tRNA N6-ade	40	11	11

A0A6B1RQB	ATP-binding	GO782_01205		ATP-binding	46	12	12
A0A0U1MQC	Succinyl-dian	BN1321_330030		Succinyl-dian	47	11	15
Q6GE41	Oxygen sens	nreB	FUNCTION:	Oxygen sens	54	16	16
A0A8E0EEK	2,5-diketo-D-	SCAG_0217		2,5-diketo-D-	47	3	14
A0A641A8C6	PH domain-c	D7S40_08865		PH domain-c	69	1	7
A0A6B5KAP	Peptidase T	pepT		Peptidase T	36	1	13
A0A8G0IYV0	50S ribosoma	rplO		50S ribosoma	71	10	10
Q2YSC2	50S ribosoma	rplJ	FUNCTION:	50S ribosoma	61	9	9
A0A8D9ZL47	Uncharacteri	SHAG_0103		Uncharacteri	51	11	11
A0A0E0VM1	Lipoprotein	ST398NM01_0529		Lipoprotein C	63	16	17
A0A380DLU	Triosephosph	tpiA_2		Triosephosph	56	1	9
Q2FVI6	Uncharacteri	SAOUHSC_02724		Uncharacteri	56	14	14
A0A2C9TUE	Coenzyme A	coaBC	FUNCTION:	Coenzyme A	51	2	15
Q2YVW6	Nucleoid-ass	SAB0428	FUNCTION:	Nucleoid-ass	59	7	7
Q6GEV1	UDP-N-acety	murA2	FUNCTION:	UDP-N-acety	43	14	14
A0A898CP4	HD domain-c	JX572_0294		HD domain-c	33	12	12
A0A2I7Y6R5	Quinol oxidas	qoxB		Quinol oxidas	16	1	9
A0A8E0B2Z	Uncharacteri	SCAG_0027		Uncharacteri	47	11	13
A0A0C6EXP	Helix-turn-hel	BTN44_01630		Helix-turn-hel	51	14	14
A0A8E0DFM	Pantoate-bet	SATG_0085		Pantoate-bet	53	1	13
A0A8D9ZPZ	SUF system	SHAG_0219		SUF system	66	9	9
A0A7Z8GCH	Aldo/keto red	E4U00_0102		Aldo/keto red	58	13	13
A0A8G2M7C	Glyoxalase/bl	NCTC7972_		Glyoxalase/bl	67	2	7
A0A8E0DQB	Uncharacteri	SHAG_0089		Uncharacteri	48	2	12
A0A8B1CLY	2-succinyl-5-	menD		2-succinyl-5-	39	20	20
A0A1Q8DEE	Glutamate de	BSZ10_06350		Glutamate de	34	1	10
A0A6B5AQ4	Glycerophos	G0W76_03015		Glycerophos	56	14	14
A0A6B8R16	Amidohydrola	SAHC1335_05448		Amidohydrola	39	9	9
Q8NV63	Formimidoylg	hutG	FUNCTION:	Formimidoylg	48	1	10
A0A2X2JV03	Probable nic	nadD	FUNCTION:	Probable nic	29	13	13
A0A7Z8GCM	UPF0354 pro	E4U00_0651		UPF0354 pro	36	10	10
A0A8E0BLP	Luciferase-lik	SIAG_01029		Luciferase-lik	46	14	14
A0A7U9P4S	DNA primase	dnaG		DNA primase	29	17	17
A0A380DNW	NADH-depen	ycjS_3		NADH-depen	36	1	12
A0A0E0VNM	tRNA pseudo	truB	FUNCTION:	tRNA pseudo	57	1	16
O87367	Putative repli	rep		Putative repli	35	13	14
A0A6B5EE9	Coproheme	G0Y40_11920		Coproheme	41	8	8
A0A227LZW	Lipoprotein	A6762_03430		Lipoprotein C	54	12	12
A0A808JHW	Coagulase	BTN44_0106		Coagulase O	27	15	15
A0A8G2I0C3	Chaperonin (	hslO		Chaperonin (	36	9	9
Q6G9D8	Peptide meth	msrB		Peptide meth	82	7	7
A0A133Q104	Pantothenate	panC	FUNCTION:	Pantothenate	52	1	13
D2JG89	Kanamycin n	SAP015B_002		Kanamycin n	41	6	6
A0A8B1D9Z	Putative cyst	bshC		Putative cyst	42	23	23
A0A6B5JPT4	Phosphate-bi	G0Y40_10255		Phosphate-bi	38	1	11
A0A2S6DGT	Signal peptid	lepB		Signal peptid	66	16	16
A0A6B5EJC	Riboflavin bic	ribB		Riboflavin bic	54	18	18
A0A8E6CQK	FMN-binding	J3R87_0267		FMN-binding	35	17	17
A6QEJ8	30S ribosoma	rpsG	FUNCTION:	30S ribosoma	55	9	9
A0A380DRZ	RND multidru	swrC_1		RND multidru	35	1	11

Q2UX84	Clumping fac	clfB		Clumping fac	43	1	13
A0A8D9ZLH4	Fructokinase-	SHAG_02477		Fructokinase-	35	2	9
A0A141HMD	TrsF	pGO400_p09		TrsF OS=Sta	46	17	17
A0A033V311	Valine--tRNA	valS	FUNCTION: (	Valine--tRNA	14	1	8
A0A8E0DS94	Uncharacteri:	SHAG_01735		Uncharacteri:	90	6	6
Q2FXI6	Thioredoxin c	SAOUHSC_01860		Thioredoxin c	78	8	8
A0A0U1MMF	Cell cycle pro	gpsB	FUNCTION: I	Cell cycle pro	69	8	8
A0A8E0AX78	Uncharacteri:	SCAG_02529		Uncharacteri:	44	12	12
A0A8D9ZI61	Chorismate s	SDAG_01438		Chorismate s	38	12	12
A0A8D9ZLM3	Acetyltransfe	SHAG_00993		Acetyltransfe	65	10	10
A0A8D9ZPQ	Replication-a	SHAG_02023		Replication-a	63	19	19
A0A380DJ88	Methionyl-tRI	metS_2		Methionyl-tRI	56	1	10
A0A8F2LJJ1	Anti-sigma fa	KQU62_0901		Anti-sigma fa	65	8	8
A0A8D9ZJK7	ABC transpor	SHAG_00296		ABC transpor	41	8	8
A0A6B5LVZ4	Ribosome bic	ylqF		Ribosome bic	57	15	15
A0A1Q8DFC	Phosphoribos	purL	FUNCTION: I	Phosphoribos	21	2	10
A0A2C9THQ	Uncharacteri:	EQ90_14570		Uncharacteri:	79	1	7
A0A0E0VUT3	tRNA modific	mnmE	FUNCTION: I	tRNA modific	40	15	15
Q93EP0	50S ribosoma	rplU	FUNCTION: T	50S ribosoma	70	8	8
Q6GFZ9	Uncharacteri:	SAR1786		Uncharacteri:	38	12	13
Q2FX91	Uncharacteri:	SAOUHSC_01986		Uncharacteri:	84	4	4
A0A8E0EFA8	Uncharacteri:	SCAG_01630		Uncharacteri:	100	8	8
A0A8D9ZKG	Fructosamine	SHAG_00370		Fructosamine	41	10	10
A0A8D9ZLR7	Aspartate-sei	SHAG_01374		Aspartate-sei	65	17	17
A0A286N637	Enterotoxin C			Enterotoxin C	39	1	10
Q6GE99	Ribose-5-pho	rpiA	FUNCTION: (	Ribose-5-pho	36	10	10
A0A1Q8DG3	Aspartate--tR	aspS	FUNCTION: (	Aspartate--tR	20	1	11
Q6GJR6	NADPH-depe	nfrA	FUNCTION: I	NADPH-depe	30	8	8
A0A8E0ENN	Uncharacteri:	SAZG_00417		Uncharacteri:	17	22	22
A0A6H3Q9H	IMPACT fami	yigZ		IMPACT fami	69	12	14
A0A8G2CEY	Pyrroline-5-ca	proC		Pyrroline-5-ca	51	12	12
A0A8E0BL04	Non-ribosoma	SIAG_00878		Non-ribosoma	12	24	24
Q6GJQ9	Xanthine pho	xpt	FUNCTION: (	Xanthine pho	54	11	11
A0A8E0EEE8	30S ribosoma	SCAG_00062		30S ribosoma	48	9	9
A0A1Q8DEH	Acyl-CoA syn	BSZ10_06045		Acyl-CoA syn	17	1	9
A0A0D1JIG0	Threonine sy	QU38_13005	FUNCTION: (	Threonine sy	36	10	10
A0A1Q8DH4	Fructose-1,6-	fbp		Fructose-1,6-	16	2	12
Q6GGY8	Probable Ctp	SAR1432		Probable Ctp	34	15	15
A0A6B0BGH	DJ-1/PfpI/Yhl	GO793_02290		DJ-1/PfpI/Yhl	70	1	8
A0A1Q8DEH	Uncharacteri:	BSZ10_06015		Uncharacteri:	46	1	6
Q5HDW0	50S ribosoma	rplW	FUNCTION: (	50S ribosoma	75	9	9
A0A8E0ED84	MiaB tRNA m	SCAG_01979		MiaB tRNA m	30	9	11
A0A5S9C522	Arginine ABC	TMSFP482_22810		Arginine ABC	60	4	12
A0A8E0B259	Uncharacteri:	SCAG_01626		Uncharacteri:	85	8	8
A0A0E1VRA1	Recombinase	HMPREF0776_0152		Recombinase	50	13	13
A0A8E0AZZ5	4-hydroxyber	SCAG_01698		4-hydroxyber	78	11	11
A0A8E0EE94	Succinyl-dian	SCAG_02396		Succinyl-dian	29	3	9
Q2G2T3	50S ribosoma	rplI	FUNCTION: I	50S ribosoma	66	12	12
Q9AFA9	Leukocidin Lu			Leukocidin Lu	41	12	12
A6U0Q8	DNA-directec	rpoY		DNA-directec	78	7	7

A0A6B5GT3(	Oxygen-depe	betA		Oxygen-depe	38	17	17
A0A2X2JZ55	Tryptophan--t	trpS		Tryptophan--t	43	1	12
A0A5F0HIE2	dITP/XTP pyı	E3A28_0289(	FUNCTION:	dITP/XTP pyı	49	9	9
A0A6B0CV4(	Pseudouridin	GJH15_05690		Pseudouridin	52	12	12
A0A7U3XK5(	Adenine phos	HUW54_082		Adenine phos	56	1	6
A0A1Q8DFH	Uncharacteriz	BSZ10_04425		Uncharacteriz	82	6	6
A0A8D9Z3X1	Phosphopant	SATG_0129		Phosphopant	46	1	14
A0A2S6DFE	Probable tran	AS852_03380		Probable tran	49	8	8
A0A8E0ENY	Iron complex	SIAG_02003		Iron complex	42	16	16
A0A141HME	Single-strand	pGO400_p16		Single-strand	75	11	11
A7X428	Aspartyl/gluta	gatC	FUNCTION:	Aspartyl/gluta	91	9	9
A0A8D9ZHM	Primosomal p	SDAG_0115		Primosomal p	27	16	20
A0A8G0JJ32	Monofunction	sgtB		Monofunction	44	10	10
A0A8E0EII4	Uncharacteriz	SCAG_0085		Uncharacteriz	47	12	12
A0A6F9YTF7	Response reg	JICS137_075		Response reg	53	10	10
A0A8G2HWE	Rod shape-de	NCTC7972_(		Rod shape-de	39	11	11
A0A8G2HZQ	Acetyl-coenzy	accA		Acetyl-coenzy	47	12	14
Q6RYZ3	Superoxide d	sodA	FUNCTION:	Superoxide d	85	1	10
T1Y7K1	GTP pyropho	SAKOR_00922		GTP pyropho	43	9	9
A0A1Q8DAZ	Fe-S cluster a	BSZ10_12540		Fe-S cluster a	21	1	10
A0A8E0B1R	Reductase	SCAG_0080		Reductase O	39	3	10
A0A6A8FX5	Ribosome hib	raiA		Ribosome hib	43	8	8
A0A8E0AX3	Uncharacteriz	SCAG_0251		Uncharacteriz	65	3	12
A0A7Z8FQ9	S-ribosylhom	luxS		S-ribosylhom	44	7	7
A0A2Z4GSR	Glyceraldehy			Glyceraldehy	82	1	8
A0A8E0AYB	Thiamine bios	SCAG_0211		Thiamine bios	41	15	15
Q6GEP3	Zinc-type alc	SAR2277		Zinc-type alc	34	2	11
A0A5S9C3L	NAD-depend	sfcA		NAD-depend	32	1	13
A0A8D9ZRI1	Osmoprotecta	SHAG_0208		Osmoprotecta	28	13	13
A0A033V062	Glutamate de	V070_01062		Glutamate de	17	1	6
A0A7I8NNL9	Redox-sensir	rex		Redox-sensir	45	10	10
Q5HIS8	Single-strand	ssb	FUNCTION:	Single-strand	68	7	9
A0A8E0EQZ	Osmoprotecta	SIAG_01239		Osmoprotecta	39	14	15
A0A8D9ZQB	Type I restric	SHAG_0179		Type I restric	38	11	16
M1XJV2	Methicillin res	mecR1		Methicillin res	23	13	13
A0A8F2LIN6	Class I SAM-	KQU62_0705		Class I SAM-	42	11	11
A0A6B0CHF	Dephospho-C	coaE		Dephospho-C	57	14	14
A0A8E0BDJ	Peroxiredoxir	SAZG_0255		Peroxiredoxir	70	11	11
A0A0H3JM4	DEAD-box AT	cshB	FUNCTION:	DEAD-box AT	34	2	14
A0A8E0EFU	Iron complex	SCAG_0115		Iron complex	39	12	12
A0A0E1VKV(	Cell envelope	HMPREF0776_2046		Cell envelope	38	16	16
A0A8A7XFM	Quinol oxidas	qoxB		Quinol oxidas	14	1	9
A0A8D9ZQK	Hydrolase, al	SHAG_0194		Hydrolase, al	43	11	11
A0A6B5CJ27	NAD(P)H-dep	G0W85_12770		NAD(P)H-dep	38	1	9
T1Y7J0	50S ribosoma	rplK	FUNCTION:	50S ribosoma	49	7	7
A0A8E0AXT	Sensor protei	SCAG_0189		Sensor protei	33	16	16
A0A8B1CWF	Adenine phos	apt		Adenine phos	53	1	6
A0A6B5GWJ	Zinc ABC trai	adcA		Zinc ABC trai	37	12	16
A0A2C9TIR1	4-hydroxy-tet	dapA	FUNCTION:	4-hydroxy-tet	41	11	12
A0A8E0EEY	Uncharacteriz	SCAG_0120		Uncharacteriz	89	4	10

A0A6B1RIZ7	Cell division p	divIB		Cell division p	26	12	12
A0A0H3JN87	SA2129 prote	SA2129		SA2129 prote	36	10	10
A0A8B1CIC8	HAD family p	JYA46_0011		HAD family p	45	1	11
A0A1Q8DG2	GTPase Obg	obg	FUNCTION:	GTPase Obg	33	1	14
A0A8D9W4U	Uncharacteri	SAAG_00653		Uncharacteri	42	10	10
A0A6B5FQJ3	Acetyl-CoA c	G0Y65_07960		Acetyl-CoA c	39	13	13
A0A6B5H222	Aldo/keto red	G0Y99_04315		Aldo/keto red	33	1	10
Q6GB23	Glycine cleav	gcvH	FUNCTION:	Glycine cleav	52	5	5
A0A0U1MMC	HU family DN	hbs	FUNCTION:	HU family DN	83	1	8
A0A7U9J6T3	Acid sugar ph	SA1_141039		Acid sugar ph	42	9	9
A0A8E0EEL7	2-dehydropar	SCAG_00272		2-dehydropar	38	8	8
A0A8D9T176	Phosphoribos	SAEG_00997		Phosphoribos	40	10	10
A0A380ENV	Formiminoglu	hutG_1	FUNCTION:	Formiminoglu	43	1	7
A7WXT5	D-ribose pyra	rbsD	FUNCTION:	D-ribose pyra	79	9	9
A0A8E0B176	N-acetyltrans	SCAG_00521		N-acetyltrans	47	1	9
Q6G7J0	50S ribosoma	rpmE2		50S ribosoma	79	6	6
A0A8D9ZNP	Uncharacteri	SHAG_0179		Uncharacteri	27	14	17
A0A6B5D8I6	SDR family o	G0X68_04165		SDR family o	38	1	10
A0A6H0DTK	Protein A (Fra	spa		Protein A (Fra	58	2	15
A0A641A7B0	Alpha/beta hy	D7S40_08580		Alpha/beta hy	53	14	14
A0A6B0CXK	Cyclic-di-AMP	gdpP		Cyclic-di-AMP	28	17	17
A0A380E1P4	Arsenate redu	spxA_2		Arsenate redu	64	7	7
A0A0H3JU66	Uncharacteri	SA1296		Uncharacteri	29	10	10
A0A229LUA6	HlyD family s	A6762_12015		HlyD family s	46	9	9
A0A7U3XJ74	LCP family pr	HUW54_065		LCP family pr	44	12	13
A0A133Q1L5	Uncharacteri	HMPREF3211_01085		Uncharacteri	48	15	16
A0A7U4ET84	D-3-phospho	M013TW_17		D-3-phospho	31	12	12
A0A6A9H1N	Glucosamine	nagB		Glucosamine	46	11	11
A0A7Z1MZG	Formate acet	CV021_1443		Formate acet	25	1	7
A0A8B0Z8Y8	HAD family h	KAJ74_0457		HAD family h	39	8	8
Q2UWN8	MHC class II	map		MHC class II	42	1	13
A0A7Z8FTH6	HAD family p	E3K14_0471		HAD family p	45	1	11
A0A7U7EUB	Uncharacteri	SAR0526		Uncharacteri	55	13	13
A0A6F9YWR	Nitroreductas	JICS137_215		Nitroreductas	54	10	10
A0A1Q8DGV	Lipoteichoic a	BSZ10_0172	FUNCTION:	Lipoteichoic a	15	1	8
A0A5F0HMU	Thymidine kir	tdk		Thymidine kir	57	8	8
A0A0E0VQY	2-oxoisovaler	ST398NM01_1583		2-oxoisovaler	51	9	9
A0A8B1CZA	GTP cyclohyd	JYA73_0014		GTP cyclohyd	39	13	13
A0A8D9Z229	CMP-binding	SATG_00603		CMP-binding	49	14	14
A7X1Q2	Ribosome-bir	rbfA	FUNCTION:	Ribosome-bir	60	7	7
A0A8E0AZ77	RNA pseudou	SCAG_0223		RNA pseudou	64	14	14
A0A7U8F5H	Translation ir	infC		Translation ir	47	8	8
A0A8E0BEJ8	Cysteine des	SAZG_0164		Cysteine des	36	10	10
A0A0E1XA81	Alanine racer	alr	FUNCTION:	Alanine racer	27	10	10
A0A133QBP	Accessory re	sarH1		Accessory re	33	1	9
A0A7U8XJG	HTH-type tra	SAXG_00611		HTH-type tra	34	1	9
A0A1Q8DC9	ATP-depende	clpP	FUNCTION:	ATP-depende	39	1	7
A0A830Z3F2	HTH-type tra	SAHC1335_(		HTH-type tra	46	6	9
G9FR64	DNA-directed	rpoB		DNA-directed	58	1	10
A0A0E8GJ7	Octanoyltrans	lplA	FUNCTION:	Octanoyltrans	33	2	7

A0A0E0VNP(	Hydrogen pe	ST398NM01_0750		Hydrogen pe	33	10	10
A0A8E0BH0	Deoxynucleo	SAZG_0052		Deoxynucleo	51	9	9
Q2G243	DNA repair p	radA	FUNCTION:	DNA repair p	30	12	12
Q6GEW9	ATP synthas	atpH	FUNCTION:	ATP synthas	68	10	10
A0A8E0DR6	PTS system,	SHAG_0093		PTS system,	21	10	10
A0A1Q8DD5	Isocitrate de	BSZ10_08400		Isocitrate de	29	2	12
A0A8E0EFP	Acetyltransfe	SCAG_0020		Acetyltransfe	68	9	9
A0A1Q8DE0	GTPase Der	der	FUNCTION:	GTPase Der	29	1	10
A7X160	Phenylalanin	pheS		Phenylalanin	34	10	11
Q07707	TraA	trsA		TraA OS=Sta	41	13	13
A0A6G4IXZ2	DUF1672 do	G0X02_1391		DUF1672 do	29	1	10
A0A8E0AZQ	Uncharacteri	SCAG_0156		Uncharacteri	66	6	6
A0A5S9I460	Oligoribonucl	TMSFP482_16140		Oligoribonucl	31	7	7
A0A6B5EJR	Protein phos	G0Y40_03435		Protein phos	55	2	11
A0A229LVN	Bacillithiol sy	A6762_07060		Bacillithiol sy	45	7	8
A0A1D5ABI2	50S ribosom	rplF_2	FUNCTION:	50S ribosom	71	1	7
A0A8E0B123	Phosphoribo	SCAG_0141		Phosphoribo	94	6	6
Q2G0F0	Uncharacteri	SAOUHSC_00619		Uncharacteri	50	9	9
A0A8E4CAH	U32 family p	HCU70_1122		U32 family p	32	10	10
G8I0W9	Regulatory p			Regulatory p	45	15	15
A0A8D9ZMQ	Glycosyl tran	SHAG_0189		Glycosyl tran	40	15	15
A0A6F9YRE	DEAD-box A	cshB		DEAD-box A	34	1	13
A0A6C1ZVQ	D-ornithine--	iucC_1		D-ornithine--	26	15	15
A0A8E0DN8	Uncharacteri	SDAG_0004		Uncharacteri	33	13	13
A0A6B1RJD	Alpha/beta fo	GAY51_06250		Alpha/beta fo	62	14	14
A0A8D6P4E	Histidine am	hutH		Histidine am	27	13	13
A0A6M1X8M	Elongation fa	tuf		Elongation fa	43	1	5
A0A0E0VNM	FtsK	ST398NM01_1278		FtsK OS=Sta	25	20	20
A0A033UTK	DNA gyrase	gyrB	FUNCTION:	DNA gyrase	18	2	10
A0A0E1VLW	ABC transpo	HMPREF0776_1636		ABC transpo	38	8	8
Q6GGU5	3-phosphosh	aroA	FUNCTION:	3-phosphosh	32	12	12
A0A380EPY	Copper-expo	copA		Copper-expo	20	13	13
A0A0E1VU8	AHS1 domai	HMPREF0776_1722		AHS1 domai	44	9	9
A0A8E0EHQ	Uncharacteri	SCAG_0001		Uncharacteri	35	7	7
A0A6G4IW9	GMP reducta	guaC		GMP reducta	41	2	12
A0A6B5I927	Pyridoxal 5'-p	pdxT		Pyridoxal 5'-p	64	10	10
A0A2X2JUY	Ser-Asp rich	bbp		Ser-Asp rich	9	5	12
A0A7U8SSV	Single-strand	SFAG_01667		Single-strand	19	12	12
A0A8G2M9I1	Glycine betai	proV		Glycine betai	35	9	9
A0A0H3JUD	Late compete	comEB		Late compete	60	8	8
A0A8E3XYL	YtxH domain	HK402_0965		YtxH domain	60	7	7
A0A7U4ETG	YhgE/Pip do	M013TW_23		YhgE/Pip do	29	13	13
Q2FZ83	Uncharacteri	SAOUHSC_01158		Uncharacteri	34	7	7
A0A8G1LP1	ABC transpo	KSF82_0104		ABC transpo	31	7	13
A0A8D9T121	rRNA methyl	SADG_0189		rRNA methyl	50	10	10
Q2FX13	UPF0435 pro	SAOUHSC_02093		UPF0435 pro	85	1	6
A0A6B5LHV	ATP-depend	recD2		ATP-depend	19	13	13
A0A8G1LNP	5'-3' exonucl	KSF82_0706		5'-3' exonucl	34	7	7
Q2FZG5	Uncharacteri	SAOUHSC_01039		Uncharacteri	40	13	13
A0A0H3K9Z	VOC family p	NWMN_2164		VOC family p	47	10	10

A0A8D9YWZ	Uncharacteri:	SASG_0035(		Uncharacteri:	35	13	13
A0A0H2HUL!	Cold shock p	BSZ10_04385		Cold shock p	80	3	6
A0A2X2JT08	Phosphoribos	purN	FUNCTION: (	Phosphoribos	59	10	10
A0A2S6DJN2	Isocitrate deh	CSC87_14005		Isocitrate deh	26	2	11
A0A8G2MA5	Phosphomev	mvaK2		Phosphomev	34	12	12
Q2FJ74	GTP cyclohy(	folE2	FUNCTION: (	GTP cyclohy(	45	9	12
A0A6A9GX7	Uncharacteri:	GAY51_08805		Uncharacteri:	52	10	10
X2JBG4	Phosphate ac	pta		Phosphate ac	86	1	6
A0A0H2HWH	50S ribosoma	rplN	FUNCTION:	50S ribosoma	65	11	11
A0A0U1MMK	DUF1672 fan	BN1321_260124		DUF1672 fan	24	1	8
A0A8D9ZL89	DNA-depend	SHAG_0109!		DNA-depend	33	16	16
A0A8E0BD84	Pantothenate	SAZG_02291		Pantothenate	59	10	10
A7X2H3	Nucleoside d	ndk	FUNCTION:	Nucleoside d	47	1	5
A0A1D5AAV	Uncharacteri:	NCTC5664_03517		Uncharacteri:	81	1	7
A0A8D9ZM5	Uncharacteri:	SHAG_0017		Uncharacteri:	51	9	9
A0A6B0CTN	NAD(P)H-bin	GO974_03100		NAD(P)H-bin	40	9	12
Q6GJB6	Ribulokinase	araB		Ribulokinase	28	12	12
A0A0H3JQ54	VOC domain-	SA1340		VOC domain-	55	1	6
A0A380DVY2	Alkaline shoc	asp23_3		Alkaline shoc	82	1	7
A0A114L1E3	Hydrolase	NCTC7878_01789		Hydrolase OS	46	13	13
A0A8E0B2M	Acetyltransfe	SCAG_0092		Acetyltransfe	75	1	9
Q5HEP0	Response re	vraR	FUNCTION:	Response re	62	11	11
A0A7U4AUB	LLM class fla	M013TW_16		LLM class fla	47	14	14
A0A8E0EF58	Phosphoglyc	SCAG_0085		Phosphoglyc	61	1	9
P0A0E3	Phosphocarri	ptsH	FUNCTION: (	Phosphocarri	57	2	3
A0A640NRP	Peptide meth	msrA1		Peptide meth	55	3	8
A0A844QYD	DUF1641 doı	GO782_1432		DUF1641 doı	41	7	7
A0A8E0DRU	Adenylate cy	SHAG_0095		Adenylate cy	45	10	10
A0A6B5BSR	GMP reducta	guaC		GMP reducta	39	1	11
A0A8E3X545	Replicative D	dnaB		Replicative D	24	10	10
A0A8E0AY81	Uncharacteri:	SCAG_0205		Uncharacteri:	56	10	10
A0A6B5CPD	ESAT-6-like	esxA		ESAT-6-like	90	7	7
A6U3W8	50S ribosoma	rplP	FUNCTION:	50S ribosoma	49	1	6
A0A141HMK	TrsH	pGO400_p11		TrsH OS=Sta	53	9	9
Q2G101	Phosphoglyc	SAOUHSC_00359		Phosphoglyc	61	1	9
A0A380DKQ	Staphylococc	sarA		Staphylococc	84	1	12
A0A8D9ZNA	Glycosyl tran	SHAG_0098		Glycosyl tran	26	14	14
A0A8F2LN23	CAP domain-	KQU62_0397		CAP domain-	38	11	11
A0A0U1MIN	Pyridoxal pho	ylmE	FUNCTION:	Pyridoxal pho	38	8	8
A0A6B5L029	UPF0473 pro	G0W85_01080		UPF0473 pro	65	1	5
A0A8G0NI64	Zinc-binding	JMO16_1190		Zinc-binding	32	1	10
A0A033UXT	Ribonucleosi	V070_00870	FUNCTION:	Ribonucleosi	13	1	8
Q2YXI3	Ribonuclease	rnc	FUNCTION:	Ribonuclease	40	8	8
A0A0E1VI94	Ribosomal R	mraW	FUNCTION:	Ribosomal R	32	8	8
A0A8G2HZ1	Pyruvate forn	pflA		Pyruvate forn	51	9	9
A0A8B1D3W	N5-carboxyaı	purE		N5-carboxyaı	44	6	6
Q2FY29	Lactamase_E	SAOUHSC_01644		Lactamase_E	55	7	7
A0A8E0B2W	Uncharacteri:	SCAG_0021		Uncharacteri:	43	7	7
A0A7Z8C5W	N-acetyltrans	E3A28_0640		N-acetyltrans	75	1	9
Q6GGX8	Conserved vi	cvfC	FUNCTION:	Conserved vi	38	11	13

A0A0E1VIX5	Primosomal p	HMPREF0776_2717		Primosomal p	35	8	10
A0A8E6CLU	tRNA threony	J3R87_1228		tRNA threony	39	9	9
A0A0U1MS9	Molybdenum	moaE		Molybdenum	69	10	10
Q2G1Y1	Uncharacteri	SAOUHSC_02125		Uncharacteri	82	5	5
A0A1Q8DHH	Formate deh	BSZ10_00245		Formate deh	11	2	11
A0A0D1JL38	Lipoprotein	QU38_09485		Lipoprotein C	24	0	10
A0A8E0DH6	N-acetyltrans	SATG_00957		N-acetyltrans	47	1	9
A0A8D9ZPF	Uncharacteri	SHAG_0133		Uncharacteri	73	6	6
Q5HG10	Uncharacteri	SACOL1445		Uncharacteri	37	10	10
A7X320	Uridine kinas	udk		Uridine kinas	52	9	9
A0A141HMD	Antitoxin Maz	pGO400_p02		Antitoxin Maz	73	5	5
A0A8E0DRG	Putative toxin	SHAG_0177		Putative toxin	40	7	7
A0A2X2LY12	Ser-Asp rich	sdrD_2		Ser-Asp rich	47	1	4
A0A7I8NK34	Putative lipop	SAMEA7024		Putative lipop	23	0	9
A0A8E0B308	Uncharacteri	SCAG_0028		Uncharacteri	95	5	7
A0A8D9Z5G	2-C-methyl-D	SATG_0148		2-C-methyl-D	52	8	12
A0A8G1GD2	Acyl-CoA deh	KSF82_0100		Acyl-CoA deh	28	3	10
A0A6B0CFB	Uncharacteri	DQV20_09835		Uncharacteri	39	6	6
A0A8G1GBK	GNAT family	KSF82_1232		GNAT family	28	6	6
A0A8F2LJM	LPXTG-anch	sasC		LPXTG-anch	10	7	16
A0A6M1X1F	VOC family p	G6X35_0207		VOC family p	29	7	7
Q6GK25	Type VII secr	essB	FUNCTION:	Type VII secr	28	10	10
P66645	30S ribosom	rpsI		30S ribosom	52	4	4
A0A6B5G5Z	SDR family o	G0V17_10390		SDR family o	56	1	10
A0A6B5FBP	NAD(P)-depe	CV021_06675		NAD(P)-depe	35	1	10
A0A1Q8DDL	Cysteine synt	BSZ10_07795		Cysteine synt	27	1	6
A0A141HMI7	Uncharacteri	pGO400_p28		Uncharacteri	87	5	5
A0A2C9TWV	MBL fold met	yycJ		MBL fold met	49	10	10
A0A7U7EUD	Uncharacteri	SAR0915		Uncharacteri	54	7	7
A0A8E0B0Z1	M23/M37 fam	SCAG_0003		M23/M37 fam	40	11	11
D2J675	TraD	traD		TraD OS=Sta	46	9	9
Q2YYL3	50S ribosom	rplR	FUNCTION:	50S ribosom	54	8	8
A0A8B1CS7	5-methyltetra	metE		5-methyltetra	23	15	15
A0A8E0B2W	ParB family c	SCAG_0053		ParB family c	37	8	9
A0A6B5HZT	Dihydrolipoyl	lpdA		Dihydrolipoyl	19	7	7
A0A8B1CGW	DUF961 fami	JYA46_0005		DUF961 fami	81	7	7
Q8VTM7	Co-chaperon	groES	FUNCTION:	Co-chaperon	74	8	8
Q6GKA4	FMN-depend	azoR	FUNCTION:	FMN-depend	49	8	8
A0A8D9ZPL	Conserved d	SHAG_0134		Conserved d	63	2	3
B2Y855	Uncharacteri		FUNCTION:	Uncharacteri	54	1	8
A0A2X2JXH	Putative lipop	NCTC7878_01914		Putative lipop	46	10	10
A0A8A7XLX	Ribosomal R	rsmG		Ribosomal R	44	5	12
A0A2W2NSK	GTP-binding	yqeH		GTP-binding	43	13	13
A0A5C8XBS	Iron-sulfur cl	HUW54_04345		Iron-sulfur cl	53	4	4
A0A2X2KCP	Segregation a	rluB	FUNCTION:	Segregation a	34	4	11
A0A8E0B34	Isochorismat	SCAG_0067		Isochorismat	53	9	9
A0A898CN2	3-hydroxyacy	fabZ		3-hydroxyacy	51	6	6
Q2YT29	5'-methylthio	mtnN	FUNCTION:	5'-methylthio	40	8	8
A0A8B1D1B	SDR family o	JYA73_0023		SDR family o	46	9	9
A8Z0B0	NAD kinase	nadK	FUNCTION:	NAD kinase	40	8	8

A0A8E0DRH	Transcription	SHAG_0246		Transcription	39	10	10
A0A0E1XJT2	Tandem lipop	HMPREF0769_10038		Tandem lipop	37	6	10
Q6GHH3	Putative zinc	SAR1238		Putative zinc	34	11	11
D1MWR0	Elongation fa	tuf		Elongation fa	24	2	6
A0A8D9ZN65	Uncharacteriz	SHAG_0234		Uncharacteriz	46	11	11
Q2YU29	Diacylglycero	dagK	FUNCTION:	Diacylglycero	33	9	9
A0A454H1R4	MurR/RpiR fa	C7P97_04505		MurR/RpiR fa	46	9	9
A0A8G2CG9	30S ribosoma	rpsM		30S ribosoma	50	9	9
A0A6K5C8K8	Glyoxylate/hy	ghrB_2		Glyoxylate/hy	31	10	10
A0A6B5HXY9	N-acetyldiam	G0X69_11920		N-acetyldiam	29	9	9
A7X1D2	Cell division p	sepF	FUNCTION:	Cell division p	39	7	7
A0A8B1CHH	YfcC family p	JYA46_0009		YfcC family p	14	9	9
A0A8D6P6G	Pathogenicity	SAMEA1965		Pathogenicity	68	10	10
A0A6H3QA2	Glycerate 2-k	garK_1		Glycerate 2-k	27	1	10
A0A8E6CKD	Carbon-nitrog	J3R87_0042		Carbon-nitrog	33	9	9
A8Z339	50S ribosoma	rpmD		50S ribosoma	83	7	7
A0A6B5I1R6	Lipid II isoglu	gatD		Lipid II isoglu	53	9	9
A0A6B5HR9	Endoribonucl	ybeY		Endoribonucl	57	2	7
A0A1Q8DBR	Thioredoxin	BSZ10_11020		Thioredoxin	56	2	7
Q6GIZ3	Arsenate red	arsC	FUNCTION:	Arsenate red	47	6	6
A0A0U1MUH	YycH protein	BN1321_430110		YycH protein	26	9	9
A0A8F2RLC	FAD/NAD(P)-	KQU62_0736		FAD/NAD(P)-	28	11	11
Q6GDB5	tRNA uridine(	trhO		tRNA uridine(	33	8	8
A0A8E0DPM	TagF domain	SHAG_0015		TagF domain	23	13	13
A0A0E1X5A6	L-asparagina	HMPREF0769_12265		L-asparagina	26	7	7
A0A1Q8DBT	Succinate de	BSZ10_11040		Succinate de	38	2	7
A0A7U4ERQ	FtsX-like perr	M013TW_02		FtsX-like perr	29	9	9
A0A8D9ZMK	6-phospho-be	SHAG_0073		6-phospho-be	33	11	11
A0A6B5DEW	23S rRNA (U	rlmD		23S rRNA (U	27	11	11
A0A1Q8DCI2	ABC transpor	BSZ10_09760		ABC transpor	17	1	11
A0A8D9W24	ABC-type gly	SAAG_0027		ABC-type gly	35	12	12
A6U473	Isopentenyl-d	fni	FUNCTION:	Isopentenyl-d	31	10	10
A0A8B1D689	Phosphatidylg	mprF		Phosphatidylg	12	10	10
A0A7U7EV84	Putative pept	SAR1692		Putative pept	35	1	8
A0A6B0AF58	DNA-(apurini	mutM		DNA-(apurini	41	11	11
A0A7R6PBA4	Glycerate kin	SAJPND4_0		Glycerate kin	26	1	10
A0A8E0B0U	Uncharacteriz	SCAG_0018		Uncharacteriz	14	10	10
A0A454H1B3	Transposase	C7P97_04855		Transposase	21	13	13
A0A0H3JM04	Uncharacteriz	SA1321		Uncharacteriz	25	1	10
Q2FC28	Beta-lactama	blaZ		Beta-lactama	46	1	10
A0A8D9ZJJ8	Glycerophosp	SHAG_0053		Glycerophosp	51	10	10
A0A8D9ZLJ3	Putative GTP	SHAG_0028		Putative GTP	44	1	12
A0A8E0AXH	Uncharacteriz	SCAG_0230		Uncharacteriz	27	8	8
A0A6B5CJT3	Epoxyqueuos	queH		Epoxyqueuos	38	9	9
A0A8D9ZM0	Uncharacteriz	SHAG_0151		Uncharacteriz	53	6	6
A7X5G6	30S ribosoma	rpsJ	FUNCTION:	30S ribosoma	60	7	7
A8Z4C5	30S ribosoma	rpsT	FUNCTION:	30S ribosoma	46	5	5
A0A5P6RB94	Staphyloferrir	sfaB		Staphyloferrir	23	13	13
A0A8E0BHY	Cardiolipin sy	SIAG_02624		Cardiolipin sy	31	14	14
Q2YXF8	DNA-directec	rpoZ	FUNCTION:	DNA-directec	93	6	6

A0A6G4QIQ4	CsbD family	G0X55_0042		CsbD family	60	5	6
Q8NW95	5-oxoprolinas	pxpA	FUNCTION:	5-oxoprolinas	32	8	8
A0A7U7EY59	Similar to pho	SAI7S6_1008		Similar to pho	55	1	10
A0A380E5H5	Aldo/keto red	yvgN_2		Aldo/keto red	37	1	12
Q6GJ11	Response re	graR	FUNCTION:	Response re	38	8	8
A0A8E0AZM	Uncharacteri	SCAG_0144		Uncharacteri	26	10	10
A0A133Q3H	Isochorismat	HMPREF3211_00934		Isochorismat	49	8	8
A0A6B5HXP	Molybdenum	G0X69_02865		Molybdenum	57	9	9
Q2FY73	Transcription	SAOUHSC_01592		Transcription	63	1	9
A0A7Z2BXN	Probable inor	dabA		Probable inor	13	11	11
A0A5F0HJ27	TatD family d	E3A28_06310		TatD family d	29	7	7
A0A6B5D6E	Na/Pi cotrans	G0V17_12900		Na/Pi cotrans	18	9	9
A0A831EAJ0	Siderophore-	sirA		Siderophore-	28	10	10
A0A8E0EF78	Ribosomal R	SCAG_0155		Ribosomal R	26	8	8
A0A8D9ZJH	Uncharacteri	SHAG_0049		Uncharacteri	39	3	10
A0A8D9ZLT	Uncharacteri	SHAG_0085		Uncharacteri	58	11	11
A0A8D9ZMR	Putative cycl	SHAG_0082		Putative cycl	47	8	11
A0A2X2JVI0	Nucleoside d	ndk_2		Nucleoside d	91	1	5
A0A8E0B1P	Uncharacteri	SCAG_0137		Uncharacteri	29	2	2
A0A380EGC	Pyrimidine nu	nupC_2		Pyrimidine nu	19	7	7
A0A8B1D4T	Membrane-a	JYA73_0022		Membrane-a	29	14	14
A0A6B0CRX	Uncharacteri	GO941_09105		Uncharacteri	29	1	8
A0A0E1VRB	Uncharacteri	HMPREF0776_2511		Uncharacteri	26	1	8
Q6GEA0	Lysostaphin r	lyrA		Lysostaphin r	23	9	9
A0A6B5FSQ	ABC transpo	G0Y65_06465		ABC transpo	20	10	10
A0A8D9ZCZ	Metallo-beta-	SAYG_0037		Metallo-beta-	22	5	5
Q2YU47	UPF0435 pro	SAB1812c		UPF0435 pro	85	1	6
A0A641A6K2	Tagatose-6-p	pfkB		Tagatose-6-p	36	6	6
A0A133PU1	Nucleoside d	ndk	FUNCTION:	Nucleoside d	46	1	5
A0A133Q0M	Isoprenyl tra	HMPREF321	FUNCTION:	Isoprenyl tra	34	10	10
A0A8G2I0E9	Thymidylate	tmk		Thymidylate	51	8	8
A0A0E0VRU	ABC transpo	ST398NM01_2507		ABC transpo	52	10	10
I2FI09	DUF3883 do	SA0024		DUF3883 do	23	9	9
X2F7H7	Staphylococc	spa		Staphylococc	68	2	14
A0A8D9ZMG	Uncharacteri	SHAG_0111		Uncharacteri	60	4	4
Q99UT4	Uncharacteri	SA1019		Uncharacteri	41	6	6
A0A831E2K6	N utilization s	nusB		N utilization s	60	6	6
A0A8E2CR69	Acyl carrier p	GZ156_0963		Acyl carrier p	51	1	6
A0A6A8FZT6	DUF1292 do	GF572_08675		DUF1292 do	58	1	5
A0A8D9ZCY	Transcription	SAYG_0013		Transcription	63	1	9
A0A390QYZ	RNA polymer	sigB	FUNCTION:	RNA polymer	35	1	10
A0A8E0AXP	Uncharacteri	SCAG_0241		Uncharacteri	43	2	2
A0A7I8NLS5	Cytosolic pro	A6762_0290		Cytosolic pro	76	10	10
A0A133PWG	Uncharacteri	HMPREF3211_02129		Uncharacteri	70	6	6
A0A8B1CKG	U32 family pe	JYA46_0004		U32 family pe	37	1	8
A0A7Z8C6U	ABC transpo	E3A28_0378		ABC transpo	23	10	11
A0A8D9SQ1	Alpha,alpha-	SAAG_0092		Alpha,alpha-	24	11	11
A0A8B1CZL1	Octanoyltran	lipM		Octanoyltran	29	1	6
P60378	Global transc	spx	FUNCTION:	Global transc	62	2	8
A0A8E0EGA	Uncharacteri	SCAG_0161		Uncharacteri	42	1	8

A0A6B2IMD9	DUF3388 doı	GZ116_01905		DUF3388 doı	31	8	8
A0A8B1CFF(	Amino acid A	JYA46_0023:		Amino acid A	34	1	8
A0A8D9ZK12	ABC transpoı	SHAG_0077:		ABC transpoı	27	2	7
A0A7U4AV1:	DUF2750 doı	M013TW_20		DUF2750 doı	29	7	7
A0A141HML(	Uncharacteri:	pGO400_p26		Uncharacteri:	38	9	9
A0A8D9ZFB(	Dimethylader	SAYG_0255(		Dimethylader	27	8	8
Q2FHM9	Guanylate kir	gmk	FUNCTION:	Guanylate kir	30	6	6
A0A380EMB	Glyoxalase	NCTC10702_03985		Glyoxalase O	27	2	5
Q2FXW0	UPF0297 pro	SAOUHSC_01721		UPF0297 pro	72	6	6
A0A8E0BDL(	Uncharacteri:	SAZG_01221		Uncharacteri:	31	9	9
A0A8E0DFD!	PTS system (	SATG_00791		PTS system (	12	8	9
A0A8E0BKB	Uncharacteri:	SIAG_00104		Uncharacteri:	42	3	5
A0A7Z1N5Y8	50S ribosomı	CV021_0293		50S ribosomı	25	1	4
A0A7U8XRP	DNA-3-methy	SFAG_01688		DNA-3-methy	54	8	8
A0A0D1JVB8	Phosphonate	QU38_15065		Phosphonate	42	8	14
A0A6B5HD8!	Cysteine desı	CV021_05200		Cysteine desı	29	8	8
Q6GBC4	Response re(	saeR	FUNCTION:	Response re(	38	6	6
Q6GGI4	UPF0403 pro	SAR1592		UPF0403 pro	53	5	5
A0A6B5JW8	Peptide chair	prfB		Peptide chair	27	10	10
A0A8E0AY18	Replication in	SCAG_0208:		Replication in	22	9	9
A0A0H3JSG(	Similar to fru(	SAV2040		Similar to fru(	29	1	8
A0A6B0CZR:	HAD hydrolas	GO941_04850		HAD hydrolas	33	1	5
A0A0U1MEX	Uracil-DNA g	ung	FUNCTION:	Uracil-DNA g	56	10	10
Q6GH48	Protein GlcT	glcT		Protein GlcT	32	9	9
A0A8B1CSR	Aldehyde red	JYA70_0016!		Aldehyde red	31	1	8
A0A141HMK	TrsG	pGO400_p10		TrsG OS=Sta	29	7	7
A0A7U7ICT2	Putative acet	SAR1431		Putative acet	46	6	6
A0A7Z8C558	Cyclic pyrano	moaC		Cyclic pyrano	35	5	5
A0A8D9W0V	Farnesyl-diph	SAAG_0143(		Farnesyl-diph	34	1	8
A0A8E0EIA3	Transcription	SCAG_0061!		Transcription	41	7	7
A0A8E0AZT8	Uncharacteri:	SCAG_0166(		Uncharacteri:	82	3	3
A0A6G4IUQ1	N-acetyltrans	G0X02_0901		N-acetyltrans	78	6	6
A7X0M9	UPF0738 pro	SAHV_0999		UPF0738 pro	47	5	5
A0A8E0EMF	Methylated-D	SIAG_01332		Methylated-D	49	6	6
A0A8D9ZKN(	Putative Zn-d	SHAG_0026(		Putative Zn-d	35	7	7
A0A0H2HU8(	Acyl carrier p	acpP	FUNCTION:	Acyl carrier p	51	1	6
P67317	Transcription	nrdR	FUNCTION:	Transcription	52	8	8
A0A8E0B0K!	ABC-2 type tı	SCAG_0019		ABC-2 type tı	39	8	8
A0A6N8I9U3	Ribosomal RI	rlmH		Ribosomal RI	55	1	8
A0A7U4ESI7	BCCT family	M013TW_12		BCCT family	10	8	8
A0A8B1CHM	SDR family N	JYA46_0021!		SDR family N	38	7	7
A0A853PC7:	S-formylgluta	A6762_1349!		S-formylgluta	42	2	7
A0A0H3JY44	Cytokinin ribc	SAV0680		Cytokinin ribc	39	7	7
Q2G111	30S ribosomı	rpsR	FUNCTION:	30S ribosomı	58	7	7
Q2XPT6	Beta-lactama	blaZ		Beta-lactama	26	2	9
A0A1Q8DAU	Arginine--tRN	argS		Arginine--tRN	14	2	7
A0A7Z8GC8!	Tautomerase	E4U00_0451		Tautomerase	52	1	2
A0A2W3GS2	Uncharacteri:	A6762_03295		Uncharacteri:	44	12	12
A0A2S6DLA:	Amino acid A	glnQ		Amino acid A	31	9	9
Q2FDM8	HTH-type tra	arcR	FUNCTION:	HTH-type tra	40	8	8

A0A0H2XJC8	Endoribonucl	SAUSA300_1971		Endoribonucl	44	9	9
A0A8E0DUD	SdrD protein	SHAG_02711		SdrD protein	82	1	6
A0A8E0EGF3	Carboxyleste	SCAG_00903		Carboxyleste	42	8	8
A0A8E0ED88	Uncharacteri	SCAG_01994		Uncharacteri	61	8	8
A0A8G2I235	PTS system,	scrA		PTS system,	19	6	6
A0A8E0DQH	Nitrite reduct	SHAG_00189		Nitrite reduct	55	2	3
A0A1Q8DE3	Transketolas	BSZ10_0677	FUNCTION:	Transketolas	10	2	4
A0A2C9TQE	Multidrug AB	EQ90_00615		Multidrug AB	23	9	9
A0A8D9ZHP	50S ribosom	SDAG_01183		50S ribosom	52	8	8
A0A2X2K1G	DNA-directed	dnaE_1		DNA-directed	17	13	13
A0A7Z1N025	Alpha-ketoac	CV021_1245		Alpha-ketoac	32	1	3
A0A6A9GTG	DUF86 doma	GAY51_03420		DUF86 doma	35	5	5
A0A8E0DRV	NifU domain-	SHAG_02213		NifU domain-	84	4	4
A0A8D9ZQI5	LMBE-relatec	SHAG_01895		LMBE-relatec	67	10	10
Q2FZ61	Thiamine dip	SAOUHSC_01190		Thiamine dip	54	9	9
A0A7H9CDW	Holliday junct	ruvB		Holliday junct	31	8	8
A0A380EM72	GTP pyropho	ywaC		GTP pyropho	44	1	12
P0A0G7	30S ribosom	rpsL	FUNCTION:	30S ribosom	58	9	9
A0A6B5Q4S	Response re	G0Y66_00180		Response re	53	10	10
A0A2S6DU3	MurR/RpiR fa	ybbH_1		MurR/RpiR fa	36	8	8
A0A8B0Z5A4	Uncharacteri	KAJ74_09670		Uncharacteri	69	8	8
A0A8E0EEF8	HTH-type tra	SCAG_02027		HTH-type tra	51	5	5
A0A8D9ZLD3	Formate/nitrit	SHAG_00193		Formate/nitrit	19	4	4
A0A1Q8DGP	Thioredoxin r	BSZ10_01475		Thioredoxin r	22	1	6
A0A0E0VPC3	Dimethylallylt	ST398NM01_1587		Dimethylallylt	33	1	8
A0A033UPJ3	Chaperonin C	groEL	FUNCTION:	Chaperonin C	14	3	7
Q2FX98	HTH cro/C1-t	SAOUHSC_01979		HTH cro/C1-t	57	7	7
A0A8G1LPH	Bi-componen	hlgB		Bi-componen	28	9	9
Q2YX97	50S ribosom	rpmF		50S ribosom	51	2	2
A0A8D9ZPA5	PhnB protein	SHAG_01159		PhnB protein	55	7	7
A0A6B5FLF3	DUF1292 do	G0Y99_13885		DUF1292 do	55	1	5
A0A1Q8DDV	Probable mal	mqo		Probable mal	15	1	8
A0A8B1CI02	GntR family t	JYA46_00081		GntR family t	42	1	8
A0A8E0EEW	Carboxyleste	SCAG_01195		Carboxyleste	38	3	8
A7X358	Holliday junct	ruvA	FUNCTION:	Holliday junct	48	8	8
A0A7U7IEC9	PSP1 C-term	SAI7S6_1003		PSP1 C-term	39	11	11
A0A8E0AYR3	YbaK/EbsC fa	SCAG_01111		YbaK/EbsC fa	44	9	9
A0A8G2M8P	Nucleoside tr	mazG		Nucleoside tr	19	8	8
A0A658BHP9	Iron transpor	DQV20_12035		Iron transpor	31	9	9
A0A6A8G111	Heptaprenyl	GF572_12275		Heptaprenyl	48	8	8
A0A0E0VQH	Arginine repr	argR	FUNCTION:	Arginine repr	42	7	7
Q6GFV3	tRNA (guanin	trmB	FUNCTION:	tRNA (guanin	40	6	6
A0A0D1I8V4	Uncharacteri	QU38_08420		Uncharacteri	28	11	11
A0A0U1MQZ	Probable suc	dapE		Probable suc	17	1	7
A0A0U1MLZ	HTH-type tra	malR		HTH-type tra	29	9	9
A0A8D9ZP12	Aluminum res	SHAG_01260		Aluminum res	24	8	8
A0A8D9ZKR	Phage major	SHAG_02378		Phage major	35	7	7
A0A659ZZW	CsbD family	ELP52_02795		CsbD family	59	7	8
A0A380DX03	OxaI/YidC m	oxaI		OxaI/YidC m	21	7	7
A0A8E0DGE	Uncharacteri	SATG_00030		Uncharacteri	34	1	7

A0A8E0AZJ3	Uncharacteri	SCAG_0140(		Uncharacteri	24	10	10
A0A033UVT2	Global transc	spx	FUNCTION:	Global transc	55	1	7
A0A8E0B0I1	Haloacid deh	SCAG_0014(		Haloacid deh	43	7	7
A0A059VP30	Protein A (Fra			Protein A (Fra	78	1	8
A0A7Z1N3T3	Probable nitr	CV021_0757		Probable nitr	26	8	8
Q2YXK2	Ribosome ma	rimM	FUNCTION:	Ribosome ma	47	6	6
Q5HGM7	Orotate phos	pyrE	FUNCTION:	Orotate phos	38	6	6
A0A7U7EW3	Putative sma	SAR2475		Putative sma	38	5	5
A0A6B5TT87	Acetyltransfe	G0004_06620		Acetyltransfe	19	11	11
A0A380EJN8	Glucokinase	glcK_1		Glucokinase	98	1	4
A0A0H3KAN	MarR family t	NWMN_2527		MarR family t	50	8	8
A0A8D9YX79	Penicillinase	SASG_0237(		Penicillinase	53	8	8
A0A0H3KH6(	HTH-type tra	NWMN_1655	FUNCTION:	HTH-type tra	39	1	6
Q2FVN4	Uncharacteri	SAOUHSC_02668		Uncharacteri	38	5	5
A0A6B5HZC	Probable cell	whiA		Probable cell	22	8	8
A0A8E0EG57	Alcohol dehy	SCAG_0072		Alcohol dehy	25	1	7
A0A8E0DR4	Sodium:dicar	SHAG_0087		Sodium:dicar	19	6	6
A0A6N8I6Q0	30S ribosoma	rpsP		30S ribosoma	57	4	4
A0A6B5EV33	L-lactate perr	G0Z62_01680		L-lactate perr	9	3	4
Q7A2W2	Teichoic acid	tagH	FUNCTION:	Teichoic acid	26	9	9
A0A7U4ESC	ABC transpo	M013TW_10		ABC transpo	22	7	7
A0A7Z1SD04	Peptidase M2	CV021_0726		Peptidase M2	20	4	4
A0A8D9ZLM	Glutamyl-ami	SHAG_0024		Glutamyl-ami	26	7	7
A0A1D5ACH	30S ribosoma	rpsE_2		30S ribosoma	75	1	5
A0A8D9ZM1	Uncharacteri	SHAG_0011(		Uncharacteri	38	6	6
A0A1Q8DC3	Autolysin	BSZ10_10670		Autolysin OS	19	1	7
Q6GF05	Endoribonucl	mazF	FUNCTION:	Endoribonucl	79	7	7
A0A5S9I518	Choloylglycin	TMSFP482_18250		Choloylglycin	20	6	6
A0A8D9Z0Q	Deoxyribodip	SASG_0203(		Deoxyribodip	20	9	9
A0A0E1VKN	Uncharacteri	HMPREF0776_2070		Uncharacteri	80	8	8
T2AUI9	Penicillin-bin	mecA		Penicillin-bin	14	3	8
Q6GGV2	CCA-adding	cca	FUNCTION:	CCA-adding	19	6	6
A0A1Q8DBT	50S ribosoma	rplP	FUNCTION:	50S ribosoma	49	1	6
A0A6D2H0L(	Glycerol upta	glpP		Glycerol upta	39	7	7
A0A7I0YLW8	RNA binding	yaaA		RNA binding	91	6	6
A0A8E0EHH	GTP-binding	SCAG_0164		GTP-binding	25	9	9
A0A0E1XFD	Ribosomal R	HMPREF076	FUNCTION:	Ribosomal R	33	7	7
A0A8D9ZN4	Riboflavin sy	SHAG_0174		Riboflavin sy	51	10	10
A0A6B0AAI2	4,4'-diapophy	GO661_05190		4,4'-diapophy	31	8	8
A0A2X2K6R	Acetyltransfe	NCTC7878_03071		Acetyltransfe	30	4	4
A0A380DRH	N-acetylmura	NCTC5664_01081		N-acetylmura	82	1	6
A0A8E0AY42	Uncharacteri	SCAG_0250		Uncharacteri	51	8	8
A0A8E0EE52	Teichoic acid	SCAG_0226		Teichoic acid	22	9	9
Q2FF68	Ketol-acid re	ilvC	FUNCTION:	Ketol-acid re	24	7	7
A0A0E1XAC	HAD hydrola	HMPREF0769_11760		HAD hydrola	59	7	7
A0A6B1RTN	D-glycerate d	GAY51_03410		D-glycerate d	28	1	8
Q2G2L0	HTH dtxR-typ	SAOUHSC_00638		HTH dtxR-typ	37	6	8
A0A2S6DFQ	DUF4176 do	esaC		DUF4176 do	47	5	5
Q6GGY3	Peptide meth	msrA2	FUNCTION:	Peptide meth	36	7	7
A0A6B5HX9	2,3,4,5-tetrah	dapD		2,3,4,5-tetrah	35	8	8

A0A6G4N7S:	Cardiolipin sy	cls		Cardiolipin sy	20	9	9
A0A133PYK7	Lactose phos	BTN44_13400		Lactose phos	32	8	8
A0A8E0DSF:	Hydrolase, C	SHAG_0037(		Hydrolase, C	21	11	11
A0A8D9ZN7(	MutT/NUDIX	SHAG_0093:		MutT/NUDIX	50	6	6
A0A7U4ET8:	Uncharacteri:	M013TW_20:		Uncharacteri:	32	4	4
A0A844QTX:	Elongation fa	fusA		Elongation fa	12	1	4
A0A380EFX8	Ribonucleotic	nrdE_3		Ribonucleotic	59	1	3
A0A8E6FJK1	HD domain-c	J3R87_0774!		HD domain-c	39	8	8
A0A8E0B0C!	Dihydroxyace	SCAG_0106!		Dihydroxyace	37	4	4
A0A8D9ZKR(	HAD-superfa	SHAG_0030!		HAD-superfa	35	8	8
Q6GGU4	3-dehydroqui	aroB	FUNCTION: (	3-dehydroqui	32	9	9
A0A7U7EU9(	AHS2 domair	SAR0771		AHS2 domair	31	10	10
Q2G1N9	L-lactate perr	SAOUHSC_(	FUNCTION: '	L-lactate perr	14	6	7
Q7BTD5	Cell division (	ftsZ		Cell division (	79	1	4
A0A7Z1N39(	RNA-binding	CV021_0774		RNA-binding	29	3	5
A0A0U1MSM	Molybdopterii	moaD		Molybdopterii	61	5	5
A0A0D6HKK:	Farnesyl pyrc	hepT		Farnesyl pyrc	29	9	9
A0A6A8G0N	Uncharacteri:	GF572_10425		Uncharacteri:	52	3	4
A0A6B5SXJ1	Ntox21 doma	G0W85_10445		Ntox21 doma	29	1	8
A0A0D1HIT5	Glycosyltrans	QU38_10220		Glycosyltrans	26	9	9
Q2FVV6	Lactose phos	SAOUHSC_02585		Lactose phos	34	8	8
A0A6G4Q2U	DNA polymer	holA		DNA polymer	30	9	9
A0A5F0HI22	7-cyano-7-de	queC	FUNCTION: (	7-cyano-7-de	41	7	7
Q6GG32	Probable GTI	engB	FUNCTION: I	Probable GTI	41	6	6
Q2YYV2	Urease acces	ureG	FUNCTION: I	Urease acces	47	7	7
A0A811I4C6	Aldehyde-alc	adhE		Aldehyde-alc	17	13	13
A0A6F8P0C1	Bacteriocin in			Bacteriocin in	16	9	9
Q2YT49	Ribosomal pr	prmA	FUNCTION: I	Ribosomal pr	24	7	7
A0A8E0DTD(	Inositol mono	SHAG_0105{		Inositol mono	21	6	6
A0A6B5UC3	RidA family p	G0X17_13645		RidA family p	51	6	6
Q2FY19	Ferric uptake	fur	FUNCTION: /	Ferric uptake	63	1	6
A0A1Q8DD7	Alanine dehy	BSZ10_08475		Alanine dehy	13	1	3
A0A1Q8DFL:	Chromosoma	dnaA	FUNCTION: I	Chromosoma	21	1	11
A0A7U4ASN	Bacillithiol sy:	ytxJ		Bacillithiol sy:	65	5	5
A0A7U7EVJ(	Putative mer	SAR1973		Putative mer	20	7	7
A8Z1J3	D-alanyl carri	dltC	FUNCTION: (	D-alanyl carri	59	4	4
A0A8B1CUN	Esterase fam	JYA66_0009:		Esterase fam	34	8	8
A0A6B5FC9(	NAD-depend	cobB		NAD-depend	27	5	5
Q6GDM3	HTH-type trai	SAR2658		HTH-type trai	38	7	7
A0A6B5M4H	Response re	G0V76_07330		Response re	43	7	7
A0A1Q8DD3	ATP synthase	atpF	FUNCTION: (	ATP synthase	29	1	5
A0A6B5EX98	LPXTG-anch	sasC		LPXTG-anch	5	1	10
A0A8E0AYT:	Acetyltransfe	SCAG_0109:		Acetyltransfe	38	6	6
A0A0E1VNB!	Kinase, PfkB	HMPREF0776_1056		Kinase, PfkB	29	9	9
P21222	30 kDa neutr		FUNCTION: I	30 kDa neutr	54	1	2
A0A2X2JWP	Peptide-meth	msrA1		Peptide-meth	49	1	6
A0A6B5TWP	Uncharacteri:	G0004_13555		Uncharacteri:	38	5	5
A0A6B5AR9:	Tetratricopep	G0W76_05465		Tetratricopep	19	8	8
A0A8E0DG7(	GAF domain-	SASG_0078		GAF domain-	47	7	7
A0A0U1MUC	Ribosomal RI	rsmG	FUNCTION: :	Ribosomal RI	31	1	8

A6QJ70	Translation ir	infA	FUNCTION: (	Translation ir	78	4	4
A5IV07	50S ribosoma	rplQ		50S ribosoma	46	5	5
A0A8D9ZP65	Heat shock p	SHAG_01207		Heat shock p	40	1	5
A0A2X2K372	tRNA-dihydro	dus	FUNCTION: (	tRNA-dihydro	30	8	8
A0A380DVR3	Endoribonucl	ybeY	FUNCTION: S	Endoribonucl	30	1	6
A0A2Z6BCC0	Superantigen	sel		Superantigen	28	8	8
A0A6B5F1N4	Beta-channel	G0Z62_12290		Beta-channel	32	9	9
A0A1Q8DF55	ATP-depende	hslU	FUNCTION: A	ATP-depende	13	1	8
A0A0D1JUE1	Glutamate AE	QU38_03955		Glutamate AE	21	9	9
A0A7I8NKQ3	Membrane lip	lpl9_1		Membrane lip	27	4	8
A0A0E0VT73	50S ribosoma	rpmC		50S ribosoma	59	5	5
Q2G0G4	N-acetyltrans	SAOUHSC_00605		N-acetyltrans	32	6	6
A0A8E0DS70	Putative mem	SHAG_00129		Putative mem	21	8	8
A0A8D6SML	7,8-dihydro-8	nudG		7,8-dihydro-8	61	7	7
A0A0E1VM11	Chorismate b	HMPREF0776_1719		Chorismate b	22	8	8
A0A8E0BCD4	Tra8	SAZG_00473		Tra8 OS=Sta	32	5	5
A0A6B0BNK5	GLOBIN dom	GO793_16175		GLOBIN dom	40	5	5
A0A7Z8GB73	Alpha/beta hy	E4U00_1268		Alpha/beta hy	32	10	10
A0A8E0DSZ5	HAD-superfa	SHAG_02645		HAD-superfa	24	5	5
A0A0D1JRI9	Allophanate h	QU38_08890		Allophanate h	29	8	8
A8Z500	ATP synthase	atpC	FUNCTION: I	ATP synthase	48	4	4
A0A8G2M8N	Shikimate 5-c	aroE		Shikimate 5-c	34	6	6
A0A8D9ZNX5	Uncharacteriz	SHAG_02293		Uncharacteriz	79	4	4
A0A8E4DBZ0	TIGR01212 fa	AS852_09090		TIGR01212 fa	27	8	8
A0A133PUJ7	Glucokinase	HMPREF3211_02503		Glucokinase	28	7	7
A0A1Q8DAS	Alkaline shoc	BSZ10_12870		Alkaline shoc	28	1	6
A0A8G2HZA	Acetyltransfe	NCTC7972_0		Acetyltransfe	43	5	5
A0A8G2I009	Accessory re	NCTC7972_0		Accessory re	38	7	7
A0A8B1CUF2	DUF21 doma	JYA66_00082		DUF21 doma	22	6	6
A0A033V7A1	Succinate--C	sucC	FUNCTION: S	Succinate--C	11	1	5
Q2FXJ7	1-acyl-sn-glyc	SAOUHSC_01837		1-acyl-sn-glyc	37	8	8
A0A380ELE1	PTS system,	glvC_2		PTS system,	22	1	4
A0A0H3JT43	Glycine cleav	SAV0324	FUNCTION: I	Glycine cleav	57	6	6
A0A6B0BUI7	Peptidase M2	GO651_10480		Peptidase M2	24	8	8
Q2FXY2	CRM domain	SAOUHSC_01698		CRM domain	46	5	5
A0A6G4IVX4	Glutathione S	G0X02_0997		Glutathione S	41	5	5
A0A8D9Z1G9	ADP-ribose p	SATG_00258		ADP-ribose p	53	8	8
A0A8B1CGX	Cystatin-like f	JYA46_00060		Cystatin-like f	48	6	6
Q6GJ34	N-acetylgluco	tarA	FUNCTION: (	N-acetylgluco	28	5	5
A0A8E0EEM	Lipoprotein	SCAG_00287		Lipoprotein C	38	7	7
A0A6B5CHF4	Cold shock p	G0W85_08050		Cold shock p	89	1	7
A0A8D9ZP86	HAD superfa	SHAG_01128		HAD superfa	36	2	9
A0A8D9ZM94	Transcription	SHAG_02289		Transcription	70	7	8
A0A8E0BHF2	HTH-type tra	SIAG_02115		HTH-type tra	37	5	5
A0A517IV62	Tandem-type	FP479_14580		Tandem-type	30	2	8
A0A8D9ZMH	O-succinylbe	SHAG_01770		O-succinylbe	32	9	9
A0A7Z8DES0	Glyoxalase fa	E4U00_0963		Glyoxalase fa	27	8	8
Q6GEP0	6-phospho-be	lacG		6-phospho-be	20	8	8
Q6GKE0	Heme oxyger	isdI	FUNCTION: A	Heme oxyger	46	5	5
A0A8G1GCE	Amidohydrola	KSF82_0038		Amidohydrola	27	8	8

A0A380DLA5	Glutaryl-CoA	NCTC5664_00654		Glutaryl-CoA	26	1	8
A0A8E1Z5G7	DnaD domain	GZ156_1173		DnaD domain	29	7	7
A0A6B5UY90	Ribulose-pho	rpe		Ribulose-pho	32	5	5
Q2G2W8	Uncharacteri:	SAOUHSC_0	FUNCTION:	Uncharacteri:	63	5	5
A0A5S9I470	Tryptophan s	trpB	FUNCTION:	Tryptophan s	23	7	7
A0A0U1MY76	Staphyloxantl	ssaA2_1		Staphyloxantl	24	6	6
A0A8D9ZQE	Rhodanese fa	SHAG_0151		Rhodanese fa	47	7	7
Q6GG27	50S ribosoma	rplT	FUNCTION:	50S ribosoma	36	6	6
A0A7U3XMU	PTS sugar tra	HUW54_135		PTS sugar tra	10	6	6
A0A8D9ZLD2	Capsular poly	SHAG_0063		Capsular poly	27	7	7
A0A033UKX8	Cysteine--tRN	cysS		Cysteine--tRN	11	1	4
A0A2X2M9U	Fibrinogen-bi	fib_2		Fibrinogen-bi	32	4	4
A0A380ENP7	30S ribosoma	rpsK_1		30S ribosoma	55	2	5
A0A6F9YVD	Amino acid A	JICS137_168		Amino acid A	21	4	4
A0A8D9YYK	Transcription	SASG_0064		Transcription	29	5	5
Q2FWB0	HTH arsR-typ	SAOUHSC_02388		HTH arsR-typ	47	4	4
A0A7U7IDR6	Respiratory n	narJ		Respiratory n	29	5	5
A0A133PXV0	S-adenosylm	queA	FUNCTION:	S-adenosylm	24	4	8
Q9L3N4	Cytosolic pro	BTN44_01455		Cytosolic pro	32	2	6
Q7WU34	Uncharacteri:	GQX37_05695		Uncharacteri:	79	5	5
A0A8E3XZK9	ABC transpo	HK402_0345		ABC transpo	23	5	5
Q6GJE7	Transcription	ctsR	FUNCTION:	Transcription	41	5	5
A0A2S6D322	tRNA pseudo	truA	FUNCTION:	tRNA pseudo	24	7	7
A0A6B5I615	ABC transpo	G0X69_11875		ABC transpo	15	5	5
Q99WC3	Recombinatio	recR	FUNCTION:	Recombinatio	35	6	6
F8SPH2	DNA topoisor	gyrA		DNA topoisor	59	1	7
A0A6B5DNA	UvrABC syste	uvrC		UvrABC syste	11	7	7
Q5HIG6	tRNA(Ile)-lysi	tilS	FUNCTION:	tRNA(Ile)-lysi	25	11	11
A0A8D9ZGY	Uncharacteri:	SDAG_0251		Uncharacteri:	25	8	8
A0A8H2KZG	DUF1027 dor	DQV20_075		DUF1027 dor	35	5	5
A0A7D5PT64	Transposase	SA0759_020		Transposase	56	9	9
A0A8E0EG99	Uncharacteri:	SCAG_0086		Uncharacteri:	18	8	8
A0A7U8XIJ1	Carboxylic es	SAXG_0027		Carboxylic es	15	7	7
A0A8D9SED	Insertion elen	SAAG_0263		Insertion elen	24	2	6
A0A7R6SM6	Aminotransfe	SAJPND4_0		Aminotransfe	18	6	6
A0A380EHC	30S ribosoma	rpsB_1		30S ribosoma	58	1	4
A0A8E0DRN	Putative phos	SHAG_0110		Putative phos	32	4	4
A0A6B0CUG	Histidine kina	GO974_05070		Histidine kina	25	9	9
A0A6B5QL60	4-hydroxy-tet	dapB		4-hydroxy-tet	38	6	6
A0A8D9ZN53	Central glyco	SHAG_0214		Central glyco	27	8	8
Q2FY42	Biotin carbox	SAOUHSC_0	FUNCTION:	Biotin carbox	29	4	4
X2FBG7	Staphylococc	spa		Staphylococc	66	2	10
A0A0E1XLU0	Oxygen regul	nreC		Oxygen regul	33	5	5
A0A1Q8DG0	Glutamate-1-	hemL		Glutamate-1-	14	1	4
A0A6B5DDD	Dihydrofolate	G0Y26_13270		Dihydrofolate	60	2	7
A0A8E4CLK	YtxH domain-	HK402_1011		YtxH domain-	35	7	7
A0A0D1K2R	Energy-coupl	ecfA1_1	FUNCTION:	Energy-coupl	25	6	6
A0A2S6DHA	PTS glucose	ptaA		PTS glucose	14	5	5
A0A0H3KI87	Uncharacteri:	NWMN_2160		Uncharacteri:	72	5	5
A0A033V0D5	Thioredoxin r	V070_00908		Thioredoxin r	12	1	4

A0A1Q8DF5(	Ribosome-re	frr	FUNCTION:	Ribosome-re	33	2	6
A0A8E0B162	Uncharacteri:	SCAG_0036(		Uncharacteri:	35	4	4
F4NA89	Penicillin binc	pbp4		Penicillin binc	19	6	6
Q9LBY7	MaoC-like do			MaoC-like do	25	4	4
A0A7U4ASI7	NAD(P)-depe	M013TW_06		NAD(P)-depe	21	6	6
A0A454H274	HTH-type trai	C7P97_03370		HTH-type trai	52	1	6
A0A0D1HHH	Small ribosor	rsgA	FUNCTION:	Small ribosor	32	7	7
A0A0H3JV76	MW0784 pro	MW0784		MW0784 pro	32	3	3
A0A830YPN1	Diadenylate c	dacA		Diadenylate c	28	7	7
A0A8D9ZQG	Secretory ext	SHAG_0216		Secretory ext	19	7	7
A0A8D9Z152	Morphine 6-d	SATG_0212(		Morphine 6-d	20	6	6
A0A7U4CFM	Dihydrofolate	folA		Dihydrofolate	60	2	7
A0A6B5HUF	Micrococcal r	G0X69_10460		Micrococcal r	31	6	6
A0A8G0MSN	Tandem-type	JMO13_000C		Tandem-type	27	0	7
A0A7R6P2T	Transcription	SAJPND4_02		Transcription	24	4	4
Q2FW65	Uncharacteri:	SAOUHSC_02442		Uncharacteri:	38	3	3
F8WKJ6	Amidohydro-r			Amidohydro-r	23	1	5
P64147	Dihydroneopt	folB	FUNCTION:	Dihydroneopt	61	5	5
A0A7R6NZR	50S ribosoma	rpmA		50S ribosoma	51	7	7
A0A6B0BH4(	D-aminoacyl-	dtd		D-aminoacyl-	31	5	5
A0A033UTS1	Ribosome-bir	ychF	FUNCTION:	Ribosome-bir	13	1	4
A0A7Z1SDW	Branched-chi	CV021_0176		Branched-chi	15	1	4
A0A6B0CTS	Class A sorta	srtA		Class A sorta	31	5	5
A7X2X5	30S ribosoma	rpsU		30S ribosoma	50	6	6
A0A6B5TTI0	TrkA family p	G0004_01555		TrkA family p	31	6	6
A0A8D9ZJD	Mannose-6-p	SHAG_0042		Mannose-6-p	26	8	8
A0A8B1CZW	Serine acetyl	cysE		Serine acetyl	27	6	6
A0A0B6XNL	AI-2 transpor	yhhT_2		AI-2 transpor	12	6	6
A0A6B5I4P2	Energy-coupl	G0Y40_01145		Energy-coupl	32	6	6
A0A8B1CG1	PepSY doma	JYA46_0003		PepSY doma	35	4	4
A0A2I7Y8S0	Alpha/beta hy	BTN44_11290		Alpha/beta hy	39	5	8
A0A6B5I833	Uncharacteri:	G0X69_10470		Uncharacteri:	26	6	6
A0A7U3XIP6	Noncanonica	HUW54_055		Noncanonica	33	1	8
A0A0D1JUL1	Acetolactate	alsS		Acetolactate	17	8	8
A6QJ81	50S ribosoma	rplX	FUNCTION:	50S ribosoma	52	4	4
Q2G2X2	Glycerol-3-ph	tarD	FUNCTION:	Glycerol-3-ph	40	7	7
A0A8E0EDR	Nuclease	SCAG_0120		Nuclease OS	32	7	7
A0A8D9W1L	Diphosphome	SAAG_0101		Diphosphome	20	5	5
A0A8D9ZJL7	L-serine dehy	SHAG_0031		L-serine dehy	25	6	6
A0A8E0B0M	Uncharacteri:	SCAG_0014		Uncharacteri:	30	5	5
A0A8D9ZM2	ABC transpo	SHAG_0154		ABC transpo	31	8	8
Q99T18	Peroxide-res	perR	FUNCTION:	Peroxide-res	43	5	5
A0A8E0DQD	Inositol mono	SHAG_0009		Inositol mono	27	9	9
A0A0U1MLW	Transcription	zur		Transcription	63	1	6
A0A8F8R74	Copper-trans	copB		Copper-trans	11	6	6
A0A6G4N7K	Regulatory pr	recX		Regulatory pr	27	6	6
A0A7G3L2E1	Plasmid segr			Plasmid segr	39	4	4
Q2G2T9	Uncharacteri:	SAOUHSC_00711		Uncharacteri:	14	5	5
A0A8G2HY7	Teichoic acid	tagB		Teichoic acid	24	8	8
A0A033V6U	Glutamine sy	V070_00363		Glutamine sy	13	1	5

A0A6B5HT04	Serine protea	G0X69_01670		Serine protea	27	5	5
Q6GKK1	Uncharacteri:	SAR0107		Uncharacteri:	10	6	6
A0A0E1VSP(	Raf-like prote	HMPREF0776_1969		Raf-like prote	40	5	5
A0A7Z1SDT3	Putative 5'(3')	CV021_0897		Putative 5'(3')	26	4	4
A0A8D9YUP	Esterase	SASG_0138(		Esterase OS=	35	1	6
A0A222UC06	Methicillin res	mecA		Methicillin res	42	2	6
A0A6B0CNJ(	NADP-depen	GO793_09360		NADP-depen	98	1	5
A0A6A8G0L4	Response reg	GF572_11640		Response reg	17	4	4
A6QJ83	30S ribosoma	rpsQ	FUNCTION: (	30S ribosoma	49	5	5
A0A8E0EEK(	Riboflavin bio	SCAG_02162		Riboflavin bio	23	7	7
A0A7U7ETI4	Acetoacetyl-C	fadA		Acetoacetyl-C	25	7	7
A6U2X1	UPF0374 pro	SaurJH1_1955		UPF0374 pro	46	6	6
Q2FZF6	Uncharacteri:	SAOUHSC_01074		Uncharacteri:	30	4	4
A0A0H3K9X8	Alpha-acetola	alsD		Alpha-acetola	26	5	6
A0A6B5M28(	Histidine kina	G0V76_11540		Histidine kina	22	6	6
A0A6B5QLP3	DUF5085 fan	G0W85_08695		DUF5085 fan	37	5	5
A0A1J0JBT5	Type VII secr	EP54_00270		Type VII secr	75	6	6
A0A6B5HT84	AraC family tı	rsp		AraC family tı	11	8	8
A0A8E0B0S1	Uncharacteri:	SCAG_00309		Uncharacteri:	53	5	5
A0A7R6P3A3	Ribosome ma	rimP		Ribosome ma	17	3	3
A0A8E0B1V2	Uncharacteri:	SCAG_00854		Uncharacteri:	69	4	4
Q2G2M5	Uncharacteri:	SAOUHSC_00514		Uncharacteri:	45	8	8
A0A0H3JL94	SA0734 prote	SA0734		SA0734 prote	33	1	6
A0A8E0AYI0	Uncharacteri:	SCAG_01009		Uncharacteri:	20	10	10
A0A8E0DI19	Long-chain fa	SATG_01469		Long-chain fa	13	6	6
A0A8B0ZMR	Phenol-solub	pmtD		Phenol-solub	18	4	4
A0A0U1MSI8	DsbA family p	BN1321_400055		DsbA family p	31	8	8
A0A033UHQ	Glutamate--tF	gltX	FUNCTION: (	Glutamate--tF	12	1	5
A0A2S6DLZ5	50S ribosoma	rplB	FUNCTION: (	50S ribosoma	14	1	3
A0A6B5F9Z4	Putative hem	lolD_1		Putative hem	26	1	6
A0A8F8TPT7	Cell division p	zapA		Cell division p	68	6	6
Q99W51	tRNA-specific	tadA	FUNCTION: (	tRNA-specific	38	5	5
A0A0E0VNV4	Ribonuclease	rnhC	FUNCTION: I	Ribonuclease	17	5	5
A0A8E0EED	Peptidase	SCAG_01952		Peptidase OS	13	7	7
A0A6A8FEL(	EIICB-Mtl	GJH15_12780		EIICB-Mtl OS	11	6	6
A0A8B1CHE	FMN-depend	JYA46_0014(		FMN-depend	38	7	7
A6U1G3	UPF0154 pro	SaurJH1_1431		UPF0154 pro	45	5	5
A0A6B0BMQ	Signal transd	arlS		Signal transd	17	6	6
A0A0D1JZB6	Aminotransfe	QU38_08320		Aminotransfe	21	8	8
A0A346CFZ8	Staphylococc	spa		Staphylococc	77	1	5
Q6GHK8	ATP-depende	recG	FUNCTION: (	ATP-depende	10	7	7
A0A380DPI0	YycH family p	yycI		YycH family p	29	5	5
A0A2X2M7Y1	Pyrimidine nu	nupG_2		Pyrimidine nu	19	5	5
A0A6B5L9X9	Alpha-acetola	budA		Alpha-acetola	25	5	6
A0A8D9ZMZ	Putative trans	SHAG_02565		Putative trans	21	2	2
A0A5S9I433	Response reg	arlR		Response reg	26	4	5
A0A8E0DRZ9	Neutral zinc r	SHAG_01446		Neutral zinc r	18	2	3
A0A8F2LJW1	Tandem-type	KQU62_1116		Tandem-type	21	3	5
A0A8G0ICS8	MurR/RpiR fa	JMO08_0003		MurR/RpiR fa	23	5	5
A0A0U1MRR	DUF4870 doı	BN1321_380138		DUF4870 doı	21	3	3

A0A0E0VQK	Putative cyto:	ST398NM01_1625		Putative cyto:	16	3	3
Q2FX89	Putative tRN/	SAOUHSC_(	FUNCTION:	Putative tRN/	47	6	6
A0A6B5KBI3	Deoxynucleo:	G0Y30_13250		Deoxynucleo:	46	1	4
A0A6B5AUH	Uncharacteri:	G0W76_11195		Uncharacteri:	11	4	4
A0A6G4Q0L	Biofilm opero	G0Y27_0359		Biofilm opero	25	4	4
A0A0U1MRL	Molybdopteri	moeB		Molybdopteri	12	4	4
A0A6B8R7F1	DUF5082 doi	SAGV69_00327		DUF5082 doi	39	7	7
A0A8E0DEV	Uncharacteri:	SASG_01712		Uncharacteri:	17	7	7
A0A380DL24	3-ketoacyl-Co	NCTC6133_00395		3-ketoacyl-Co	34	1	3
A0A0U1MI49	UPF0637 pro	yktB		UPF0637 pro	25	6	6
A0A0U1MGC	Putative proli	proP		Putative proli	12	5	5
A0A2X2M3Q	Cell-division i	NCTC7878_00032		Cell-division i	33	6	6
A0A2X2JS99	Nitroreductas	NCTC7878_00568		Nitroreductas	30	4	5
A0A6B5AS1	Cell-wall-ancl	sasF		Cell-wall-ancl	16	10	10
A0A8D9Z40	Uncharacteri:	SATG_01632		Uncharacteri:	20	4	4
A0A8E0B0Z	Deoxynucleo:	SCAG_0096		Deoxynucleo:	25	1	4
A0A8F7WY5	Cell division	ftsL		Cell division	30	4	4
A0A8D9ZMN	Conserved hy	SHAG_0076		Conserved hy	29	0	5
A0A8E0B124	4,4'-diaponeu	SCAG_0038		4,4'-diaponeu	16	5	5
A0A1L6C092	Sensor protei	vraS		Sensor protei	20	8	8
A0A8E0EFA	Uncharacteri:	SCAG_0189		Uncharacteri:	13	7	7
A0A7Z8GB9	Protein-tyrosi	E4U00_1205		Protein-tyrosi	32	4	4
A0A7U4ERP	ADP-depend	nnrD		ADP-depend	19	5	5
A0A1Q8DGQ	Nucleotide-bi	BSZ10_0147	FUNCTION:	Nucleotide-bi	14	1	5
A0A033UZE	FAA_hydrola:	V070_01073		FAA_hydrola:	13	3	4
A0A8D9YWN	Uncharacteri:	SASG_0253		Uncharacteri:	41	7	7
A0A8E0AZ84	Uncharacteri:	SCAG_0137		Uncharacteri:	49	4	4
Q2FXF3	Uncharacteri:	SAOUHSC_01896		Uncharacteri:	78	4	4
A0A8D9YU7	Antibiotic trar	SASG_01131		Antibiotic trar	11	3	3
A0A8D9WCT	Phosphate tra	SAEG_0131		Phosphate tra	27	6	6
A0A8E0DL7	DeoR family	SAYG_0057		DeoR family	23	6	6
A0A6B0BT51	Bifunctional f	GO793_16675		Bifunctional f	14	5	5
A0A6B5GLE	Gluconokinas	gntK		Gluconokinas	9	4	4
A0A2S6D87	Magnesium tı	mgtE	FUNCTION:	Magnesium tı	21	8	8
A0A8D9ZN7	Hypothetical	SHAG_0153		Hypothetical	32	3	3
A0A033UB1	Glutamyl-tRN	gatA	FUNCTION:	Glutamyl-tRN	9	1	3
A0A8E0AZ9	5'-nucleotidas	SCAG_0136		5'-nucleotidas	11	5	5
A0A2X2K0C	Putative hem	macB		Putative hem	33	5	5
A0A033V8M	Thioredoxin	V070_00093		Thioredoxin	31	1	3
A0A8E0AZ24	Methionine in	SCAG_0122		Methionine in	20	5	5
A0A831E8C	Acyl-CoA thic	SAGV51_021		Acyl-CoA thic	51	6	6
A0A380EHK	Protein-expo	NCTC10702_	FUNCTION:	Protein-expo	38	2	2
A0A8E0B1S	Uncharacteri:	SCAG_0074		Uncharacteri:	38	5	5
A0A8E0DS1	Uncharacteri:	SHAG_0237		Uncharacteri:	30	3	3
A0A8D9ZQ0	Putative acet	SHAG_0159		Putative acet	25	3	3
A0A6B5I8Q7	dUTPase	G0Y40_02140		dUTPase OS	25	2	5
Q6GHS9	Fibrinogen-bi	fib	FUNCTION:	Fibrinogen-bi	30	1	5
A0A2X2K0G	Mannose-6-p	gmuF		Mannose-6-p	25	6	6
A0A380EK0	Aldehyde del	aldH_3		Aldehyde del	60	1	4
A0A1Q8DHS	Zinc ABC trai	BSZ10_00730		Zinc ABC trai	13	1	5

A0A6B5GH3	ATP-depende	hslV		ATP-depende	40	1	5
A0A8E0DTV	Uroporphyrin	SHAG_0164		Uroporphyrin	33	2	6
D2JD73	Beta-lactama	blaR1		Beta-lactama	9	1	6
A0A8E0B05	Uncharacteri	SCAG_0179		Uncharacteri	45	3	3
A0A8B0ZBL	Bi-componen	lukG		Bi-componen	19	1	6
A0A033V5K4	Isoleucine--tR	ileS	FUNCTION:	Isoleucine--tR	6	2	7
A0A8B1CVH	DNA polymer	JYA70_0015		DNA polymer	19	5	5
A0A7Z8C6G	Uncharacteri	E3K14_0024		Uncharacteri	21	7	7
A0A7U3XMG	MarR family t	HUW54_128		MarR family t	44	7	7
A0A0E1VKE	Uncharacteri	HMPREF0776_1397		Uncharacteri	10	4	4
A0A8E0DT8	Conserved d	SHAG_0245		Conserved d	49	3	3
A0A0E0VTJ1	Uncharacteri	ST398NM01_2403		Uncharacteri	25	5	5
A0A8E0DRA	Uncharacteri	SHAG_0042		Uncharacteri	26	5	5
A0A8E0B0P	Formate deh	SCAG_0010		Formate deh	21	6	6
A7X140	Phosphopant	coaD	FUNCTION:	Phosphopant	29	5	5
A0A1Q8DDZ	HTH-type tra	BSZ10_07220		HTH-type tra	18	1	4
Q84GA7	Surface prote	sasD		Surface prote	30	3	3
A0A0E1X9Y	EIIA	HMPREF0769_11001		EIIA OS=Sta	9	7	7
A0A346CG9	Staphylococc	spa		Staphylococc	59	1	4
A0A8D9YYX	Uncharacteri	SASG_0263		Uncharacteri	23	4	7
A0A380E0Q	Succinyl-CoA	sucC_2		Succinyl-CoA	60	1	3
A0A6B5MJH	DUF3885 do	G0Z61_05405		DUF3885 do	38	6	6
A0A1Q8DFF	2-oxoglutarat	odhA	FUNCTION:	2-oxoglutarat	5	1	3
A0A380EHQ	Succinate de	sdhA_2		Succinate de	60	1	5
A0A7D9N3J	Penicillin bin	mecA		Penicillin bin	23	1	5
A0A0E0VPU	NAD(FAD)-ut	ST398NM01_1808		NAD(FAD)-ut	12	6	6
A0A8G2HZW	Prevent-host-	relJ		Prevent-host-	65	1	3
A0A2S6DJA	Fructose-1,6-	fba		Fructose-1,6-	21	1	4
Q2G242	TRAM domai	SAOUHSC_00508		TRAM domai	26	7	7
A0A641AB4	DUF2294 do	D7S40_07605		DUF2294 do	43	5	5
Q2YUT0	Uncharacteri	SAB0040		Uncharacteri	20	1	6
A0A641A3V2	Heavy-metal-	D7S40_13115		Heavy-metal-	46	2	2
A0A6B5LYU	DUF1024 fan	G0X68_13620		DUF1024 fan	48	4	4
Q6GBY6	Peptidyl-tRN	pth	FUNCTION:	Peptidyl-tRN	38	5	8
A0A8B1CXS	Lipoteichoic	yfhO		Lipoteichoic	6	5	5
A0A1Q8DBV	Endonucleas	mutS2	FUNCTION:	Endonucleas	7	1	6
A0A8D9SP0	Chromosome	SAAG_0084		Chromosome	21	5	5
A0A2X2MDU	Phosphocarri	ptsH_1		Phosphocarri	85	1	2
A6QJJ8	HTH-type tra	tcaR	FUNCTION:	HTH-type tra	39	5	5
A0A8G2HYP	Transposon-	NCTC7972_		Transposon-	24	1	5
A0A2X2K0P	Membrane s	NCTC7878_02816		Membrane s	17	5	5
A0A8E0EPM	Uncharacteri	SIAG_01733		Uncharacteri	31	4	4
A0A6A8FYA	Uncharacteri	GF572_07260		Uncharacteri	22	1	5
A0A7U4ETQ	Histidine kina	hssS		Histidine kina	14	5	5
A0A7U7IF36	5-formyltetra	SAI7S6_101		5-formyltetra	26	4	4
Q2TF08	Putative bact			Putative bact	30	3	3
A0A8D9Z39	Uncharacteri	SATG_0188		Uncharacteri	49	4	4
A0A033V401	Ferredoxin	V070_00532		Ferredoxin O	17	1	2
A0A8E0DRQ	Uncharacteri	SHAG_0087		Uncharacteri	25	6	6
A0A0E1XEH	Putative 4-di	ispE	FUNCTION:	Putative 4-di	30	6	6

A0A6B5CKQ	Molybdopteri	mobB		Molybdopteri	28	4	4
O87357	Helix-turn-hel	res		Helix-turn-hel	27	5	5
A0A7I8NJC0	Sensor protei	phoR		Sensor protei	15	1	6
A0A6B5HSZ8	N-acetyltrans	G0X69_07870		N-acetyltrans	27	3	3
A0A2S6DHM	L-threonine d	ilvA	FUNCTION:	L-threonine d	18	6	6
A0A0E0VQK	Acylphosphat	ST398NM01_1406		Acylphosphat	38	2	2
A0A0E0VP34	Ribonuclease	ST398NM01_1433		Ribonuclease	35	4	4
A0A6G4IVT1	Amino acid p	G0X02_0910		Amino acid p	10	7	7
A0A8E0AYN	Uncharacteri	SCAG_0106		Uncharacteri	21	4	4
A0A2X2JZ38	Integral mem	NCTC7878_02549		Integral mem	13	3	3
A0A8B1D42	Tandem-type	JYA73_0003		Tandem-type	15	1	5
A0A6B5I3J7	ABC transpo	G0X69_09670		ABC transpo	29	5	5
A0A808JIC5	UPF0291 pro	BTN44_0589		UPF0291 pro	47	4	4
Q8NXQ4	Undecapreny	uppP	FUNCTION:	Undecapreny	18	1	5
A0A6B0BUI5	Enolase	eno		Enolase OS=	12	1	3
A0A6H3Q8X	Putative N-ac	nanE		Putative N-ac	21	4	4
Q2YU83	Uncharacteri	SAB1876c		Uncharacteri	14	1	5
A0A1Q8DCP	Branched-ch	BSZ10_09190		Branched-ch	9	1	5
A0A0D1HK5	Probable cell	QU38_08770	FUNCTION:	Probable cell	18	5	5
A0A2S6DMC	Glutaredoxin	A6762_05235		Glutaredoxin	32	2	2
A0A6B5IBH9	Uroporphyrin	G0V76_07715		Uroporphyrin	27	1	5
A0A8G0JKQ	dUTP diphos	JMO15_0015		dUTP diphos	20	1	4
A0A0E0VRJ	UPF0316 pro	ST398NM01_2006		UPF0316 pro	30	6	6
A0A8G2HYV	Na(+)-linked	NCTC7972_(		Na(+)-linked	7	2	2
A0A8F2RN0	Amidohydrola	KQU62_0139		Amidohydrola	18	6	6
A0A173MSL	Fibronectin-b	fnbA		Fibronectin-b	8	1	3
A0A6H3QBI2	5-bromo-4-ch	DD547_0143		5-bromo-4-ch	28	9	9
A0A6B5Q55	Restriction er	G0Z61_13490		Restriction er	14	2	6
A0A8E0DDN	Tn554-like, tr	SASG_02381		Tn554-like, tr	34	5	5
Q2FFH9	Sodium-depe	sdcS	FUNCTION:	Sodium-depe	11	5	5
A0A1Q8DD9	Alkyl hydrope	BSZ10_0834	FUNCTION:	Alkyl hydrope	25	2	3
A0A133PXV	UPF0735 AC	HMPREF3211_01795		UPF0735 AC	35	6	6
A0A8E4DCE	F0F1 ATP sy	AS852_1098		F0F1 ATP sy	7	2	2
A0A8E0EFY	Uncharacteri	SCAG_0047		Uncharacteri	12	6	6
A0A6B5D74	Cytidine dear	cdd		Cytidine dear	44	4	4
A0A1Q8DFT	Lipoate--prot	BSZ10_03550		Lipoate--prot	15	1	4
A0A6B5M2D	Polysacchari	G0X68_06895		Polysacchari	16	5	5
A0A1Q8DCH	Peptidase M	BSZ10_09605		Peptidase M	11	1	3
A0A8E0DRP	Uncharacteri	SHAG_0234		Uncharacteri	32	3	3
A0A6B1RLI6	Sensor protei	GAY51_07095		Sensor protei	14	1	6
A0A6G4Q50	Aquaporin fa	G0Y27_1003		Aquaporin fa	11	3	3
A0A6G4N3B	Class I SAM-	G0Y31_0377		Class I SAM-	18	4	4
A0A0E1XI62	DUF402 dom	HMPREF0769_11661		DUF402 dom	22	4	4
Q6G9Y9	50S ribosom	rpmB		50S ribosom	71	6	6
A0A6B1RIF6	SWIM zinc fi	GAY51_08025		SWIM zinc fi	30	4	4
A0A6B5H7X	IS3-like elem	G0W76_13645		IS3-like elem	12	1	5
A0A0H3JL70	SA0209 prote	SA0209		SA0209 prote	16	5	5
A0A380EHB	Catalase	katA_2		Catalase OS=	75	1	3
A0A8E0EEJ	Glycerophos	SCAG_0211		Glycerophos	27	6	6
A0A7Z8DAJ	Holo-[acyl-ca	acpS		Holo-[acyl-ca	47	5	5

A0A8E0BI27	N-acetyltrans	SIAG_01190		N-acetyltrans	35	5	5
A0A6B5AT07	Purine nucleo	deoD		Purine nucleo	21	3	3
A0A7U8XRA	Probable cell	SFAG_01253		Probable cell	14	5	5
A0A2X2JSP3	Putative phag	NCTC7878_00454		Putative phag	35	1	6
A0A0U1MRW	Magnesium tr	corA	FUNCTION:	Magnesium tr	17	5	5
A0A6B5HZX8	Urease subur	ureC		Urease subur	8	4	4
A0A8E0AY49	Uncharacteriz	SCAG_02005		Uncharacteriz	13	5	5
A0A380DVR9	Putative phag	NCTC5664_01988		Putative phag	36	1	6
A0A2X2KF32	ABC superfar	gsiB		ABC superfar	26	1	7
A0A8B1CHV3	GntR family t	JYA46_00223		GntR family t	24	5	5
A0A0E1XG67	Nuclease Sbc	sbcD	FUNCTION:	Nuclease Sbc	20	6	6
D2JA73	DNA recombi	SAP069A_032		DNA recombi	43	5	5
A0A380DLA1	Cytosolic pro	NCTC5664_00529		Cytosolic pro	14	1	5
A0A8E0DDL5	Lipoate-prote	SASG_01803		Lipoate-prote	14	4	4
A0A2S6DIG2	Phosphate AI	pstB3	FUNCTION:	Phosphate AI	16	6	6
A0A8E0B119	Uncharacteriz	SCAG_00431		Uncharacteriz	32	5	5
A0A1D4NWD	Immunoglobu	spa_1		Immunoglobu	36	1	3
A0A8E0DR59	Putative mem	SHAG_00597		Putative mem	17	3	3
A0A8E0DQF	HD domain p	SHAG_02529		HD domain p	31	5	5
A0A0E0VM91	6-carboxy-5,6	ST398NM01_0788		6-carboxy-5,6	41	4	4
A0A8A7XNT0	Diguanylate c	H7684_05520		Diguanylate c	12	4	4
A0A8E0B2Z1	HTH-type tra	SCAG_00580		HTH-type tra	21	4	4
A0A7U7ETU5	Uncharacteriz	SAR0411		Uncharacteriz	26	5	5
A0A0H3JMB0	SA0984 prote	SA0984		SA0984 prote	17	4	4
A0A6B2IZY8	Uncharacteriz	GZ116_08505		Uncharacteriz	52	3	3
A0A8E3Y1K5	BglG family tr	HK402_1414		BglG family tr	15	7	7
P68862	DNA replicati	recF	FUNCTION:	DNA replicati	17	7	7
A0A5S9EUL2	Haloacid deh	TMSFP482_16340		Haloacid deh	14	4	4
A0A2S6DIN8	Alpha-ketoac	CSC87_16020		Alpha-ketoac	17	1	2
Q6GIR3	NADPH-depe	queF	FUNCTION:	NADPH-depe	25	4	4
A0A8D9Z9X2	Secretory ant	SAYG_01214		Secretory ant	33	3	3
A0A8D9ZMF	Uncharacteriz	SHAG_00754		Uncharacteriz	11	6	6
A0A8D9ZMY	Replication p	SHAG_02019		Replication p	22	7	7
A0A6B5QKY	Capsular poly	G0Y30_11355		Capsular poly	14	6	6
A0A380DQ52	Nucleic-acid-	NCTC6133_01546		Nucleic-acid-	42	4	4
A0A2X2KJX4	Pseudouridin	rsuA		Pseudouridin	12	3	4
Q2FWY2	Pyrazinamida	SAOUHSC_02139		Pyrazinamida	31	5	5
A0A8E0DD54	Antibiotic AB	SASG_01308		Antibiotic AB	7	5	5
Q2YV12	PTS system I	SAB0132	FUNCTION:	PTS system I	12	5	5
A0A0E0VSA2	Probable mol	mobA	FUNCTION:	Probable mol	29	5	5
A0A6B5GI25	Nitrite reduct	nirD		Nitrite reduct	34	1	2
A0A7U7EW6	Threonylcarb	SAR2204		Threonylcarb	22	6	6
A0A8B1CTV8	Aspartate 1-c	panD		Aspartate 1-c	30	3	3
A0A0H3JS03	Uncharacteriz	SA2376		Uncharacteriz	40	5	5
A0A8G2FK94	Tyrosine reco	xerD_3		Tyrosine reco	27	6	6
Q6GID9	Na(+)/H(+) ar	mnhD1	FUNCTION:	Na(+)/H(+) ar	7	4	4
A0A1Q8DEV	Phosphonate	BSZ10_05635		Phosphonate	18	1	7
W8UWY3	Export protei	ytgP_1		Export protei	8	3	4
A0A0E1VIG1	ABC transpor	HMPREF0776_2095		ABC transpor	18	6	6
A0A6N8I8J5	30S ribosoma	rpsS		30S ribosoma	35	1	4

A0A8E0DQX	Uncharacteri	SHAG_0064(		Uncharacteri	72	5	5
A0A5A4RH2(	Protein kinas			Protein kinas	14	7	7
A0A8B1D2Q	MarR family t	JYA73_0022!		MarR family t	26	4	4
A0A5S9I2U4	Choloylglycin	TMSFP482_02270		Choloylglycin	13	4	4
A0A8E0DRY	S4 domain pr	SHAG_0268(		S4 domain pr	49	5	5
Q6GKM5	Uncharacteri	SAR0080		Uncharacteri	8	6	6
A0A1Q8DFZ(	DNA mismat	BSZ10_02935		DNA mismat	15	1	4
A0A033V2Z2	(p)ppGpp syr	V070_00692	FUNCTION:	(p)ppGpp syr	7	1	7
A0A8D9ZJQ	5' nucleotidas	SHAG_0061(		5' nucleotidas	9	4	4
A0A8D9ZP7	30S ribosom	SHAG_0122		30S ribosom	35	3	3
A0A8E0B0A	7, 8-dihydro-(	SCAG_0256		7, 8-dihydro-(	18	4	4
A0A8E0B0N	Uncharacteri	SCAG_0016		Uncharacteri	23	4	4
A0A6B5CHB	Putative hem	G0W85_12910		Putative hem	22	4	4
A0A380ELU	Protein-tyrosi	ptpA		Protein-tyrosi	34	1	4
A0A6A8FGU	Ribonuclease	rnmV		Ribonuclease	27	4	4
A0A8D9ZFE	Peptidase M	SDAG_0015		Peptidase M	29	6	6
A0A7Z1S9C	tRNA preQ1(	CV021_1679		tRNA preQ1(	23	1	5
A0A0H3JLF1	Uncharacteri	SA0349		Uncharacteri	15	3	3
A0A6B5HYZ	Acyl-CoA/acy	G0V76_04780		Acyl-CoA/acy	16	4	4
A0A8B1CIH1	tRNA (guanin	trmD		tRNA (guanin	16	3	3
A0A133PTE	Putative cyto	HMPREF3211_02765		Putative cyto	35	5	5
A0A033ULU	Serine hydro	glyA	FUNCTION:	Serine hydro	16	1	4
A0A7U7ID47	AraC family r	SAR1583		AraC family r	11	3	3
A0A0E1VTC	Uncharacteri	HMPREF0776_1910		Uncharacteri	19	4	4
A0A0E0VPC	30S ribosom	rpsO	FUNCTION:	30S ribosom	38	2	2
A0A0E0VQW	Uncharacteri	ST398NM01_2126		Uncharacteri	46	2	2
A0A0S3CW1	Fibronectin-b	fnbB		Fibronectin-b	14	1	3
Q2FXX9	Uncharacteri	SAOUHSC_01701		Uncharacteri	15	4	4
A0A6B5G8P(	Tandem-type	G0W85_01315		Tandem-type	15	1	4
A0A0E8H82	Capsular poly	capG		Capsular poly	13	5	5
A0A380ELV	Transporter y	yvqF		Transporter y	57	4	4
A0A7Z1SEW	Transcription	nusA		Transcription	15	1	2
A0A6B5QIQ1	CDP-glycerol	G0Z61_13925		CDP-glycerol	33	1	2
A0A033V2U	MiaB-like tRN	V070_00637		MiaB-like tRN	9	1	3
A0A8E0EH3	Uncharacteri	SCAG_0057(		Uncharacteri	29	3	3
A0A8E0EP9	Uncharacteri	SIAG_02171		Uncharacteri	29	5	5
A0A641A922	Bifunctional c	metB		Bifunctional c	16	5	5
A0A0H3JP1	Cold shock p	cspB		Cold shock p	52	3	4
Q931K4	Single-strand	ssb	FUNCTION:	Single-strand	23	1	3
A0A8B1CMB	Gluconate pe	JYA66_0025(		Gluconate pe	8	3	4
A0A8E0AYZ	Uncharacteri	SCAG_0124		Uncharacteri	23	2	2
A0A0E0VPH	Aspartokinas	ST398NM01_1329		Aspartokinas	14	5	5
A0A3A3AVI9	ABC transpo	ybbM		ABC transpo	12	3	3
Q6GEX9	Probable tran	sceD	FUNCTION:	Probable tran	27	4	4
A0A8D9YVX(	Peptide-meth	SASG_01421		Peptide-meth	20	3	3
A0A0E0VTC(	Integral mem	ST398NM01_2561		Integral mem	6	4	4
Q2FV43	Uncharacteri	SAOUHSC_02896		Uncharacteri	27	3	3
A0A8E0EM8	Siroheme syr	SAZG_0008		Siroheme syr	27	2	3
A0A0U1MLC	Ferredoxin	fer		Ferredoxin O	17	1	2
A0A0E0VPZ	Large-condu	mscL	FUNCTION:	Large-condu	31	3	3

A0A1Q8DE0:	Zinc metallop	BSZ10_06975		Zinc metallop	10	1	2
A0A8D9YW7	Uncharacteri:	SASG_02432		Uncharacteri:	20	6	6
A0A8E0BJF1	Uncharacteri:	SIAG_00405		Uncharacteri:	42	3	3
A0A7Z1MXU	Phosphoenol	CV021_1591		Phosphoenol	16	1	2
A0A7Z1SCJ5	DNA helicase	recQ		DNA helicase	11	6	6
A0A7U4ASP:	DNA-binding	M013TW_06		DNA-binding	25	3	3
A0A2U0IIC7	Purine nucle	pgeF		Purine nucle	20	5	5
A0A1Q8DEM	Primosomal	priA	FUNCTION:	Primosomal	7	1	5
Q7A6J6	UPF0349 pro	SA0800		UPF0349 pro	18	1	1
C3VI01	Immunoglobu	spa		Immunoglobu	92	1	5
A0A8D9Z3L8	Cytochrome	SATG_01145		Cytochrome	9	2	2
A0A8E0EF50	2-succinyl-6-l	SCAG_01388		2-succinyl-6-l	20	4	4
A0A8E0DQQ	ABC transpo	SHAG_00406		ABC transpo	20	4	4
A0A033V3E5	Acyl carrier p	acpP	FUNCTION:	Acyl carrier p	31	1	3
A0A6B5G8R:	MFS transpo	G0W85_12825		MFS transpo	9	5	5
A0A385EL44	Replication p	rep(RC)_1		Replication p	16	5	5
A0A0H3JSG	t(6)A37 threo	SAV2052		t(6)A37 threo	32	4	4
A0A7D5PLH8	CPA1 family	SA0759_020		CPA1 family	6	5	5
A0A8E0EH66	S1 RNA-bind	SCAG_01299		S1 RNA-bind	24	4	4
A0A6G4N5A:	Helix-turn-hel	G0Y31_0710		Helix-turn-hel	30	3	3
A0A6B5I5C2	Homoserine l	thrB		Homoserine l	23	6	6
X5EF25	65 kDa meml	map_1		65 kDa meml	29	4	4
A0A380E2S4	Tyrosyl-tRNA	tyrS_4		Tyrosyl-tRNA	47	1	4
A0A8F2RN8:	Nucleoside h	KQU62_1013		Nucleoside h	18	5	5
A0A6B5HU6:	Uncharacteri:	G0X69_06605		Uncharacteri:	9	3	3
A0A8E0B1X9	Uncharacteri:	SCAG_00765		Uncharacteri:	12	3	3
A0A0E1VIU2	Uncharacteri:	HMPREF0776_2045		Uncharacteri:	14	2	2
A0A2S6D4R:	HTH-type tra	gabR_1		HTH-type tra	12	5	5
A0A8D9ZME	L-serine deh	SHAG_00313		L-serine deh	20	4	4
A0A033UQ38	D-alanine--D-	ddl	FUNCTION:	D-alanine--D-	8	2	4
A0A2S6DAG	Phosphopent	CSC87_17010		Phosphopent	25	2	3
A0A7U4SWF	Protein transl	secY		Protein transl	10	4	4
Q6GI30	Putative acet	SAR1027	FUNCTION:	Putative acet	28	3	3
A0A2X2M4W	DnaI	dnaI_1		DnaI OS=Sta	46	1	3
A0A8E6FJN2	Na+/H+ antip	mnhA1		Na+/H+ antip	5	4	4
A0A8G2HY6	Multimodular	sgtA		Multimodular	13	4	4
A0A8E0EQT8	Teichoic acid	SIAG_02548		Teichoic acid	16	5	5
A0A8B0Z4Q6	LysR family t	KAJ74_08690		LysR family t	14	4	4
A0A8E3Y1S7	Sodium/proli	putP		Sodium/proli	5	2	2
A0A830YU84	Membrane p	SAGV69_031		Membrane p	39	1	1
A0A5S9ETL4	N-acetylneur	nanA		N-acetylneur	10	3	3
A0A6B5I4T8	ATP-binding	G0V76_00050		ATP-binding	16	3	3
Q6GEG6	GTP 3',8-cycl	moaA	FUNCTION:	GTP 3',8-cycl	13	5	5
A0A8F2LM1:	Lipoate--prot	KQU62_0159		Lipoate--prot	19	5	5
A0A8E0DRN	Uncharacteri:	SHAG_00815		Uncharacteri:	29	2	2
A0A0U1MPJ8	Uncharacteri:	BN1321_310030		Uncharacteri:	9	3	3
A0A8B1D130	Truncated he	JYA70_0010:		Truncated he	50	4	4
A0A8E0AY61	Uncharacteri:	SCAG_02022		Uncharacteri:	20	5	5
A0A6B0ABW	Segregation	scpA		Segregation	19	4	4
A0A0U1MHD	Aminotransfe	BN1321_170048		Aminotransfe	21	5	5

Q6GGJ4	Ribonuclease	rnz	FUNCTION:	Ribonuclease	19	4	4
Q5HHN6	SsrA-binding	smpB	FUNCTION:	SsrA-binding	31	4	4
A0A7U7EWX	Similar to xyli	SAI7S6_1001		Similar to xyli	18	1	5
A0A1Q8DDH	Metal-depenc	BSZ10_07935		Metal-depenc	23	1	4
A0A380E3B0	Undecapreny	uppP	FUNCTION:	Undecapreny	20	1	5
A0A2S6DI07	Heat-inducibl	hrcA	FUNCTION:	Heat-inducibl	13	4	4
A0A6A8FZP8	ABC transpoi	GF572_09775		ABC transpoi	18	3	3
A0A380E2B8	Preprotein tra	yajC		Preprotein tra	28	2	2
A0A6B8R5N5	Uncharacteri:	SAGV69_02971		Uncharacteri:	37	2	2
A0A1Q8DEF	Peptidyl-proly	BSZ10_0633	FUNCTION:	Peptidyl-proly	13	2	3
A0A033UDJ4	S1 motif dom	V070_02582		S1 motif dom	20	1	3
A0A8D9ZMT	Putative mem	SHAG_02287		Putative mem	8	3	3
A0A0E1VIV0	Cobalt transp	HMPREF0776_2067		Cobalt transp	13	4	4
A0A641A8B3	TIGR01741 fa	D7S40_06850		TIGR01741 fa	30	1	4
A0A641A6N6	CHY-type doi	D7S40_10490		CHY-type doi	45	5	5
A0A6B5I3M6	Peptide MFS	G0X69_09865		Peptide MFS	8	4	4
A0A7Z8D8P7	tRNA (Adeno	tsaB		tRNA (Adeno	16	3	3
A0A7I8NN34	Fur-regulatec	isdB		Fur-regulatec	8	4	4
A0A8F2LMV	DUF420 dom	KQU62_0396		DUF420 dom	10	1	1
A0A380E5V9	Protein-tyrosi	ptpA		Protein-tyrosi	31	1	4
A0A7U9P2M	Uncharacteri:	SA1_56089		Uncharacteri:	24	4	4
A0A1D5A9F2	Acid phospha	NCTC10702_00545		Acid phospha	35	1	1
A0A0H3JK0C	Uncharacteri:	SA0539		Uncharacteri:	33	3	3
A0A7U4AVW	Alkaline phos	M013TW_26		Alkaline phos	11	5	5
A0A6B5ARG	Multidrug effl	mepR		Multidrug effl	36	3	3
A0A2X2MF53	Amino acid p	NCTC7878_03506		Amino acid p	9	6	6
A0A7U8SV25	Transcription	SFAG_00342		Transcription	16	4	4
A0A033UK86	GTP cyclohyc	folE2	FUNCTION:	GTP cyclohyc	11	1	4
A0A8D9Z0H	Amino acid tr	SATG_00693		Amino acid tr	4	2	2
A0A2X2JY78	3-dehydroqui	aroC_1	FUNCTION:	3-dehydroqui	15	3	3
A0A6B5L3N7	DUF177 dom	G0W85_10140		DUF177 dom	20	4	4
A0A8E0AZR	AAT family ai	SCAG_02092		AAT family ai	7	2	2
A0A1Q8DEG	UDP-N-acety	murC	FUNCTION:	UDP-N-acety	7	1	3
A7X5C8	50S ribosoma	rpmJ		50S ribosoma	24	1	1
A0A7U4ETH	DUF4467 doi	M013TW_23		DUF4467 doi	29	3	3
Q6GFF1	Heptaprenylg	pcrB	FUNCTION:	Heptaprenylg	17	1	2
A0A7U8SRZ	Fibrinogen-bi	SFAG_01157		Fibrinogen-bi	30	1	5
A0A2X2JRF5	Tetrapyrrole (	NCTC7878_00054		Tetrapyrrole (	31	2	2
A0A8D9Z2I2	Endonucleas	SATG_00212		Endonucleas	21	3	3
A0A380E440	6-phosphoglu	gnd_2		6-phosphoglu	32	1	3
A0A8D9ZPE	Short chain d	SHAG_01234		Short chain d	21	5	5
A0A8E0EF53	Uncharacteri:	SCAG_00842		Uncharacteri:	43	3	3
Q6GHW0	tRNA(Met) cy	tmcAL	FUNCTION:	tRNA(Met) cy	11	4	4
A0A8D9ZQ38	L-2-haloalkar	SHAG_01941		L-2-haloalkar	24	5	5
A0A7U4CF26	Uncharacteri:	SAHC1335_0		Uncharacteri:	14	3	3
A0A6B5ASH	Aminotransfe	G0W76_07305		Aminotransfe	9	4	4
A0A133Q553	Acetyl-CoA C	HMPREF3211_00735		Acetyl-CoA C	7	3	3
A0A6B5EE04	2-isopropylma	leuA		2-isopropylma	6	3	3
A0A8G1GB1	DUF1108 fan	EDCC5055_0		DUF1108 fan	38	3	3
A0A8E0DDE	Manganese t	SASG_00110		Manganese t	9	3	3

A0A8F2XFX(	Organic hydr	KRH49_0259		Organic hydr	19	2	2
A0A8B1D0Y4	C39 family pe	JYA73_0023		C39 family pe	26	5	5
A0A6B5FQQ	LacI family tra	G0Y99_08455		LacI family tra	13	4	4
A0A1Q8DCY	Epimerase	BSZ10_08905		Epimerase O	8	1	3
A0A7R6NTI6	Uncharacteri	SAJPND4_0(		Uncharacteri	25	3	3
A0A8E0AXV4	O-methyltran	SCAG_0201(		O-methyltran	22	4	4
A0A6G4QMA	ABC transpor	G0X55_0754		ABC transpor	10	5	5
A0A224B1H5	Phage protei	A6762_04235		Phage protei	46	3	3
A0A033V2M2	ATP-depende	pfkA	FUNCTION:	ATP-depende	13	1	3
Q2FUW7	UDP-N-acety	gtfA	FUNCTION:	UDP-N-acety	10	4	4
A0A7Z8GBS	Nucleoside h	E4U00_0759		Nucleoside h	9	2	2
A0A380DNJ9	Phosphoribos	purC_2		Phosphoribos	41	1	4
A0A2I7Y6S7	1,4-dihydroxy	menA	FUNCTION:	1,4-dihydroxy	6	2	2
A0A7Z8DDG	Glutathione p	E4U00_0413		Glutathione p	30	4	4
A0A2W3AHG	ABC-type tra	NCTC7878_01330		ABC-type tra	7	4	4
A0A033UUV	Aldo_ket_red	V070_01574		Aldo_ket_red	7	1	2
A0A6A8FXK8	Extracellular	GF572_03060		Extracellular	15	4	4
A0A0U1MJB	Uncharacteri	BN1321_240142		Uncharacteri	28	3	3
A0A6B5GRY	1-phosphatid	G0Y66_10620		1-phosphatid	7	1	1
A7X1Z4	LexA repress	lexA	FUNCTION:	LexA repress	15	3	3
A0A8E0B069	Uncharacteri	SCAG_0000		Uncharacteri	8	4	4
Q8NXC3	Processive di	ugtP	FUNCTION:	Processive di	11	4	4
A0A6B5AUV	Aminotransfe	G0W76_11535		Aminotransfe	8	3	3
A0A7Z8C3Y1	PTS sugar tra	E3A28_0709		PTS sugar tra	30	3	3
A0A6H3Q3G	Galactitol-spe	gatB_1		Galactitol-spe	23	2	2
A0A8D9ZL02	Uncharacteri	SHAG_0252		Uncharacteri	42	1	3
A0A8G0JLN8	Magnesium t	JMO16_1291		Magnesium t	14	5	5
A0A2S6DJA9	Seryl-tRNA s	CSC87_14545		Seryl-tRNA s	14	1	4
A0A0E0VQB	Transcription	ST398NM01_2055		Transcription	30	5	5
A0A8F2RP3(	Aldo/keto red	KQU62_0211		Aldo/keto red	13	4	4
A0A6B5Q689	Inorganic pho	G0V60_12930		Inorganic pho	9	2	2
A0A8D9ZPY	Putative conj	SHAG_0127(		Putative conj	6	4	4
A0A7Z8DC7(	Nucleoside tr	ytkD		Nucleoside tr	17	3	3
A0A8E0AYU(	Uncharacteri	SCAG_0108		Uncharacteri	21	2	2
A0A224AUC(	NupC/NupG f	nupC_1		NupC/NupG f	9	3	3
A0A5F0HLQ(	HAMP domai	nsaS		HAMP domai	14	4	4
A0A6B0CUI2	Nucleotide py	GO793_12430		Nucleotide py	42	4	4
A0A7Z1SB15	Beta-ketoacy	CV021_0120		Beta-ketoacy	24	1	3
Q2G034	Uncharacteri	SAOUHSC_00793		Uncharacteri	11	3	3
A0A6B5M47	Amino acid A	G0V76_06445		Amino acid A	9	4	4
A0A7Z8CCJ5	MFS transpor	E3K14_1161(		MFS transpor	10	4	4
A0A033UTN	EIIA	V070_01519		EIIA OS=Sta	5	1	2
A0A8D9ZQJ8	ComA2 famil	SHAG_0222		ComA2 famil	16	2	2
A0A2S1FUK	Restriction er	hsdS		Restriction er	9	1	4
A0A2S6D9S9	Elongation fa	tuf		Elongation fa	61	1	2
A0A8E4FY83	XRE family tr	AS852_0638		XRE family tr	31	2	2
A0A380DV25	ABC transpor	ecsA_2		ABC transpor	8	2	2
A0A2S6D9T9	Ferredoxin	CSC87_17895		Ferredoxin O	24	1	1
A0A033V243	D-alanine am	V070_00813	FUNCTION:	D-alanine am	9	1	2
A0A8E0EDB	Glutamyl-tRN	SCAG_0206		Glutamyl-tRN	10	5	5

A0A6B0BL30	Homoserine a	GO793_15350		Homoserine a	24	4	4
A0A2X2JYN4	ESAT-6 secre	essA		ESAT-6 secre	13	2	2
A0A6B5FHJ6	Ribonuclease	rnhB		Ribonuclease	12	3	3
A0A8D9ZNY0	Putative Na+/	SHAG_00959		Putative Na+/	7	3	3
A0A0U1MGI6	Site-specific r	BN1321_150062		Site-specific r	23	4	4
A0A831E3J6	Tryptophan s	trpA		Tryptophan s	11	2	2
A0A8G0IVA4	LysR family tr	JMO11_0004		LysR family tr	14	4	4
A0A033V0L1	Cytidylate kin	cmk		Cytidylate kin	11	1	3
A0A380E4K6	Molecular cha	dnaK_3		Molecular cha	45	1	1
Q6GFR0	2-succinylber	menE	FUNCTION: (	2-succinylber	6	3	3
A0A7Z1S9U8	2-oxoacid fer	CV021_1738		2-oxoacid fer	69	1	3
A0A6M1XDS	Phosphoenol	G6X35_1683		Phosphoenol	9	1	2
Q2YX63	UPF0358 pro	SAB0977		UPF0358 pro	38	4	4
A0A6B5CWH	1-(5-phospho	hisA		1-(5-phospho	17	3	3
A0A8E0AYG	Uncharacteri	SCAG_0220(		Uncharacteri	21	4	4
A0A7U7EZW	Lipoprotein	SAI7S6_1018		Lipoprotein C	12	1	4
A0A6B0B2C7	Uncharacteri	G0W76_02790		Uncharacteri	29	2	2
A0A6A9GRV	Uncharacteri	GAY51_05780		Uncharacteri	30	2	2
A0A2S6DJ67	50S ribosoma	CSC87_14705		50S ribosoma	28	1	2
A0A6G4IWQ	YxeA family p	G0X02_1104		YxeA family p	18	2	2
A0A0E1XBI7	Beta-lactama	HMPREF0769_10704		Beta-lactama	9	5	5
A0A2S6DJG1	Nitrite reducta	CSC87_14360		Nitrite reducta	12	1	4
A0A380EGF9	2-amino-3-ke	kbl_1		2-amino-3-ke	16	1	1
A0A8F7RM5	Tyrosine reco	xerC		Tyrosine reco	8	3	3
X5DT77	Uncharacteri	DD547_00996		Uncharacteri	45	2	2
A0A033UWU	Ornithine ami	rocD	FUNCTION: (	Ornithine ami	7	1	3
A0A0E0VL43	Putative mem	ST398NM01_0488		Putative mem	16	3	3
A0A5F0HGP	Divalent meta	E3A28_00455		Divalent meta	7	4	4
A0A8D9ZEQ	Capsular poly	SAYG_01308		Capsular poly	14	2	2
A0A6B5T7R1	Protein NrdI	nrdI		Protein NrdI (	30	4	4
A0A2X2JTC2	Clumping fac	clfA_5		Clumping fac	43	1	3
A0A7R6NUY	Transcription	SAJPND4_0(		Transcription	5	2	2
A0A6G4IVE3	AraC family tr	G0X02_1014		AraC family tr	17	2	2
A0A7U7IE79	UPF0413 pro	SAI7S6_1007		UPF0413 pro	13	4	4
A0A380EJG8	50S ribosoma	rpmG2		50S ribosoma	28	1	1
A0A7U7EVS	Hypothetical	SAR2103		Hypothetical	24	2	2
A0A2X2JWJ4	Nitric oxide re	norD_1		Nitric oxide re	7	4	4
A0A6A8FXB1	Zinc-binding	GF572_03950		Zinc-binding	29	1	2
A0A033ULS8	Peptide chain	prfA	FUNCTION:	Peptide chain	11	1	4
A0A8E0EFM	Uncharacteri	SCAG_0014(		Uncharacteri	13	3	3
A0A2S6DD1(	DNA-binding	hssR		DNA-binding	17	3	3
A0A7U4ARP	SDR family o	M013TW_01		SDR family o	12	2	2
A0A033V6I7	Chromosome	smc	FUNCTION:	Chromosome	2	1	3
A0A8F7WX4	Division/cell v	mraZ		Division/cell v	16	2	2
A0A8G0NAN	LysR family tr	JMO12_0025		LysR family tr	11	4	4
A0A6B5J1Z2	S9 family pep	G0W76_01805		S9 family pep	16	3	3
A0A6B0CJV(	Elongation fa	tuf		Elongation fa	12	3	4
A0A1Q8DCV	Bifunctional a	BSZ10_08980		Bifunctional a	2	1	3
A0A831E358	Putative mem	SAGV69_016		Putative mem	11	5	5
A0A8E0AY26	Uncharacteri	SCAG_02109		Uncharacteri	5	1	1

A0A830YQD!	Cytochrome (	SAGV69_01!		Cytochrome (	8	3	3
A0A6B5DH2	Amino acid p	CV021_11850		Amino acid p	5	3	3
A0A133QCN	Glycerate kin	HMPREF3211_00078		Glycerate kin	12	4	4
A0A8E3XZ1!	Copper-sensi	csoR		Copper-sensi	40	1	3
A0A8D9ZNC	Allophanate h	SHAG_0159:		Allophanate h	15	3	3
Q0Q2J6	Putative antip	mnhE2	FUNCTION:	Putative antip	14	2	2
P0A051	Transposase	tnpA1	FUNCTION:	Transposase	7	2	3
A0A033V3I5	Probable glyc	gcvPA	FUNCTION:	Probable glyc	6	1	3
A0A0U1MV8	UDP-N-acety	mnaA		UDP-N-acety	9	3	3
A0A033V6Y8	Uncharacteri:	V070_00342		Uncharacteri:	17	1	3
A0A0D1K0L6	DNA mismatc	QU38_06665		DNA mismatc	8	4	4
Q2G0J5	UPF0382 me	SAOUHSC_00567		UPF0382 me	9	1	1
A0A0H3JVS(	MTS domain-	MW0442		MTS domain-	7	2	2
A0A6N8IAA2	30S ribosoma	rpsC		30S ribosoma	7	1	1
A0A380DHP(	Immunoglobu	spa_2		Immunoglobu	27	1	2
O33590	AgrC	agrC		AgrC OS=Sta	8	3	3
A0A033V2N!	Acetate kinas	ackA	FUNCTION:	Acetate kinas	8	1	2
A0A7U4AW2	Anaerobic rib	nrdG		Anaerobic rib	14	2	2
A0A8D9ZMC	Capsular poly	SHAG_0062:		Capsular poly	12	3	3
A0A1Q8DD7	Acetyl-coenzy	accA	FUNCTION:	Acetyl-coenzy	12	1	3
A0A033UYK(	DNA topoisor	topB	FUNCTION:	DNA topoisor	4	1	3
A0A8B1D7J7	GNAT family	JYA73_0002		GNAT family	19	3	3
A0A6C2A1C:	GNAT family	DQV20_07425		GNAT family	12	2	2
A0A2X2LZD	DNA binding	NCTC7878_01641		DNA binding	11	1	1
A0A6G4JHV:	UPF0223 pro	G0Y35_0722		UPF0223 pro	35	1	3
A0A8D9ZEP:	Uncharacteri:	SAYG_0196		Uncharacteri:	10	2	2
A0A2X2K9C(	Urease acces	ureE	FUNCTION:	Urease acces	29	3	3
A0A2S6DD3:	Putative hem	hrtA		Putative hem	17	3	3
A0A7U4ASL:	Uncharacteri:	M013TW_06		Uncharacteri:	8	2	2
A0A0E1VRZ(	Uncharacteri:	HMPREF0776_2313		Uncharacteri:	28	2	2
A0A033UZ6(	Oligopeptide	V070_01088		Oligopeptide	6	1	2
A0A033V5W	DhaL domain	V070_00166		DhaL domain	5	1	3
A0A8E0EE1	Uncharacteri:	SCAG_0244!		Uncharacteri:	16	3	3
A0A5F0HJJ5	Type 1 glutar	E3A28_07660		Type 1 glutar	13	2	2
A0A2X2JXP(	Metallothiol tr	fosB2	FUNCTION:	Metallothiol tr	20	3	3
A0A1Q8DCI6	DNA repair p	BSZ10_0965	FUNCTION:	DNA repair p	6	1	2
A0A641A7U6	Putative DNA	D7S40_09685		Putative DNA	17	2	2
A0A1Q8DCL	Aminopyrimic	BSZ10_0930	FUNCTION:	Aminopyrimic	13	1	3
A0A6A8G10	Glycerol-3-ph	plsY		Glycerol-3-ph	7	1	1
A0A2X2K0K6	TetR family re	NCTC7878_03074		TetR family re	25	2	2
A0A6B5B0U:	Abi family pro	G0X68_00995		Abi family pro	8	3	3
A0A8E0AZW	Uncharacteri:	SCAG_0230:		Uncharacteri:	40	2	2
A0A0S2JAW	Trehalose op	treR		Trehalose op	16	3	3
A0A2S6D3B	Nitric oxide re	norB_3		Nitric oxide re	6	4	4
Q6GK35	Glycyl-glycine	lytM	FUNCTION:	Glycyl-glycine	11	3	3
A0A380DX97	Membrane-b	NCTC6133_02554		Membrane-b	11	3	3
A0A8E0B09(	Cell surface p	SCAG_0003!		Cell surface p	16	3	3
A0A0C5BK7!	Conjugal tran	traJ		Conjugal tran	9	1	1
A0A2S6DIS8	Branched-cha	CSC87_15720		Branched-cha	21	1	3
Q6GJD4	50S ribosoma	rpmG3		50S ribosoma	40	3	3

K7ZRV8	Uncharacteri:			Uncharacteri:	17	3	3
A0A6C2A2K4	Autolysin	DQV20_00900		Autolysin OS	15	2	3
A0A850FZI2	Transposase	GQX37_0914		Transposase	25	3	3
A0A808JGU8	Uncharacteri:	BTN44_0188		Uncharacteri:	17	2	3
A0A8E0EGC	Transcription	SCAG_0082		Transcription	27	2	2
A0A0U1MGV	Bifunctional n	BN1321_190046		Bifunctional n	4	2	2
A0A7U7ICF4	LysR family r	SAR0717		LysR family r	10	3	3
Q6G7Z2	Staphylococc	scn	FUNCTION:	Staphylococc	9	1	1
Q6GHJ9	UPF0122 pro	SAR1212	FUNCTION:	UPF0122 pro	24	3	3
A0A5S9C3B2	Uncharacteri:	TMSFP482_03930		Uncharacteri:	21	2	2
A0A1Q8DB0	ABC transpor	BSZ10_12450		ABC transpor	14	1	3
A0A8D9ZLK4	Transcription	SHAG_0072		Transcription	12	2	2
A0A8D6XL77	Autolysis res	lytR_2		Autolysis res	10	3	3
A0A8B0ZMR	YolD-like fam	KAJ74_1152		YolD-like fam	27	2	2
A0A133QAW	Uncharacteri:	HMPREF3211_00259		Uncharacteri:	5	3	3
A0A898CQC	Succinate de	JX572_0538		Succinate de	5	1	1
A0A8E0DCP	MFS transpor	SASG_0156		MFS transpor	5	3	3
D2J7W1	Resolvase/int	bin		Resolvase/int	11	2	2
A0A6B5K648	Adapter prote	mecA		Adapter prote	14	4	4
Q2YSG7	Phosphatidyl	lgt	FUNCTION:	Phosphatidyl	11	3	3
A0A8E0EG4	Uncharacteri:	SCAG_0145		Uncharacteri:	33	2	2
A0A8G0IC19	Glutathione p	JMO09_0028		Glutathione p	12	2	2
A0A5S9C312	Peptide ABC	TMSFP482_01560		Peptide ABC	5	3	3
A0A7U3XHL	GTP-binding	HUW54_035		GTP-binding	6	2	2
A0A2X2K0G	Transcription	nusG_2	FUNCTION:	Transcription	33	1	2
A0A1Q8DDM	DtxR family t	BSZ10_07520		DtxR family t	12	1	3
A0A0E1VNG	Efem/EfeO fa	HMPREF0776_1088		Efem/EfeO fa	9	2	2
A0A8E0AZK	Uncharacteri:	SCAG_0200		Uncharacteri:	12	1	1
A0A0E0VP7	Cytochrome	ST398NM01_1084		Cytochrome	9	3	3
A0A6B5IJS7	YuzD family	G0Y40_12125		YuzD family	21	2	2
A0A2X2LUW	TetR family r	NCTC7878_00187		TetR family r	11	2	2
A0A6B5T3A	Ferredoxin--N	CV021_12145		Ferredoxin--N	10	2	2
A0A8D9ZLA	Putative DNA	SHAG_0112		Putative DNA	52	3	3
A0A0E0VM8	Uncharacteri:	ST398NM01_2847		Uncharacteri:	43	2	2
A0A8E0DRB	Uncharacteri:	SHAG_0075		Uncharacteri:	18	2	2
A0A811I3T2	Protein-ADP-	ymdB		Protein-ADP-	12	3	3
A7X1C2	Phospho-N-a	mraY	FUNCTION:	Phospho-N-a	5	2	2
A0A8B1CFQ	GAF domain-	JYA46_0023		GAF domain-	21	4	4
A0A0E1VII5	Uncharacteri:	HMPREF0776_2891		Uncharacteri:	9	1	1
D7URS2	Transposase	tnpB		Transposase	5	3	3
Q2G027	Uncharacteri:	SAOUHSC_00800		Uncharacteri:	9	1	1
A0A0U1MQL	Site-specific	BN1321_330058		Site-specific	9	3	3
Q6T1P3	Surface prote	sasE		Surface prote	19	3	3
A0A8E0AZ2	Uncharacteri:	SCAG_0116		Uncharacteri:	8	2	2
A0A8E0DUE	Veg protein	SHAG_0267		Veg protein C	34	2	2
Q9XBA2	DNA-binding	kdpE		DNA-binding	9	2	2
A0A8D9Z2D	Uncharacteri:	SATG_0020		Uncharacteri:	8	3	3
A0A8E0DCZ	VraD ABC tra	SASG_0145		VraD ABC tra	21	3	3
A0A0U1MW	Putative Histi	BN1321_60030		Putative Histi	4	2	2
A0A7R6SNI8	Uncharacteri:	SAJPND4_0		Uncharacteri:	15	1	2

A0A7U7ETM	Putative PTS	SAR0235		Putative PTS	4	2	2
A0A6H5D6T(	Integral mem	SAMEA2076		Integral mem	3	1	1
A0A7Z2H558	Putative pre-:	ruvX		Putative pre-:	12	2	2
A0A8G2I1B5	Mobile eleme	NCTC7972_(		Mobile eleme	9	2	2
A0A0H2WX	NADH dehyd	nuoF		NADH dehyd	6	3	3
A0A0S3CVX	Fibronectin-b	fnbA		Fibronectin-b	7	1	2
A0A8D9ZQR	Arsenical res	SHAG_0200		Arsenical res	17	2	2
A0A8G2M9N	Uncharacteri:	NCTC7972_(		Uncharacteri:	22	1	1
A0A0D1JYC	7-carboxy-7-(	queE	FUNCTION:	7-carboxy-7-(	14	3	3
A0A6B5BAX	Neutral metal	aur		Neutral metal	4	2	2
Q2YYZ2	Putative 3-me	SAB2222		Putative 3-me	11	2	2
A0A8D9SN8	Uncharacteri:	SAAG_0032		Uncharacteri:	19	2	2
A0A380E042	Cell division	NCTC5664_02366		Cell division	29	2	2
A0A6B5KVE	TIGR01741 f	G0X68_12340		TIGR01741 f	17	1	3
A0A8B1CHI0	Helix-turn-hel	JYA46_0006		Helix-turn-hel	7	2	2
A0A6B5CEM	TetR/AcrR fa	G0W85_06970		TetR/AcrR fa	10	2	2
A0A1Q8DD8	Peptidase M	BSZ10_08470		Peptidase M	8	1	2
A0A0D1HMS	Urease subu	ureB		Urease subu	7	1	1
A0A5S9C778	Pyrrolidone-c	pcp	FUNCTION:	Pyrrolidone-c	8	3	3
A0A8G2CGI	Malate-2H(+)	mleN_2		Malate-2H(+)	6	3	3
A0A811HMH	Amino acid p	pheP		Amino acid p	6	1	3
A0A811I6W4	Phosphotrans	SAMEA7014		Phosphotrans	14	3	3
A0A1S5YP4	MerR family t	adhR		MerR family t	14	2	2
A0A8E0EGU	Uncharacteri:	SCAG_0261		Uncharacteri:	23	3	3
A0A6B0BLQ(	Multidrug effl	norA		Multidrug effl	6	2	2
A0A7U9P2B	Hydrolase, al	SA1_125320		Hydrolase, al	17	1	4
A0A0H2XI17	Fe-S protein	sufT	FUNCTION:	Fe-S protein	35	1	1
A0A6G4JLY	Histidine kina	G0Y35_1366		Histidine kina	4	1	1
A0A8F2XND	Alpha/beta hy	KQY23_0315		Alpha/beta hy	7	2	2
A0A8E0EG8	Glycerol-3-ph	SCAG_0081		Glycerol-3-ph	9	3	3
A0A8E0EF5	Prephenate d	SCAG_0230		Prephenate d	8	2	2
A0A8E0B0P(	Acetoin utiliz	SCAG_0212		Acetoin utiliz	18	3	3
A0A6B5EVG	ABC transpo	G0Y30_10575		ABC transpo	4	2	2
A0A033UL77	L-lactate deh	ldh	FUNCTION:	L-lactate deh	3	1	1
A0A0E0VQK	Phage protei	ST398NM01_2922		Phage protei	28	1	1
A0A0E0VMC	Putative cyto	ST398NM01_0910		Putative cyto	24	3	3
A0A5F0HKM	Flavin reduct	E3A28_08895		Flavin reduct	14	3	3
A0A6B1RHW	DUF3899 do	GAY51_03130		DUF3899 do	11	1	1
A0A1Q8DDK	Peptidyl-tRN	pth	FUNCTION:	Peptidyl-tRN	18	1	4
A0A380DQB	2-dehydropa	panE_1	FUNCTION:	2-dehydropa	10	4	4
A0A6B0D24	Tyrosine-type	GO974_10310		Tyrosine-type	8	2	3
P60693	Na(+)/H(+) a	mnhF1	FUNCTION:	Na(+)/H(+) a	10	1	1
A0A2S6DE7	Iron-regulate	isdC		Iron-regulate	7	1	1
A0A1Q8DDI	Uncharacteri:	BSZ10_07645		Uncharacteri:	21	1	2
A0A0E1XAU	Acetolactate	ilvB		Acetolactate	4	2	2
A0A0E1X50	DNA repair p	recO	FUNCTION:	DNA repair p	11	3	3
A0A8D9Z03	Cadmium effl	SATG_0268		Cadmium effl	19	2	2
A0A6B5I8J4	Uncharacteri:	G0V76_05280		Uncharacteri:	9	2	2
A0A229LUE1	Sodium/gluta	gltS	FUNCTION:	Sodium/gluta	7	2	2
A0A8E3XZV	Accessory Se	asp1		Accessory Se	4	3	3

A0A8E0DSF9	Uncharacteri	SHAG_01275		Uncharacteri	18	1	1
A0A033UPA9	ATP synthase	atpF	FUNCTION:	ATP synthase	9	1	3
A0A6B0CW7	Heptaprenylg	pcrB		Heptaprenylg	17	1	2
A0A830YUE9	Accessory ge	agrA		Accessory ge	10	3	3
A0A6B5FI40	Isochorismate	G0Y99_11315		Isochorismate	20	1	3
A5JJ19	Toxic shock s	tst		Toxic shock s	12	2	2
A0A850FZB4	Uncharacteri	GQX37_0735		Uncharacteri	39	2	2
A0A6B5ATT8	Release facto	prmC		Release facto	13	2	2
A0A7U7ICJ8	Putative mem	SAR1013		Putative mem	15	2	2
A0A6G4Z9A1	Radical SAM	yfkAB		Radical SAM	4	3	3
A0A6M1XFK	CBS domain-	G6Y24_1095		CBS domain-	18	1	2
A0A8E0B3P8	Transcription	SCAG_00676		Transcription	9	3	3
A0A033V324	Translation ir	infC	FUNCTION:	Translation ir	7	1	1
A0A1Q8DFH	Conserved vi	BSZ10_04520		Conserved vi	9	1	3
A0A0H2XH49	Phi77 ORF02	SAUSA300_1944		Phi77 ORF02	37	3	3
A0A033UQ79	AAA_27 dom	V070_01982		AAA_27 dom	2	2	3
A0A7Z8DAK5	Arginine repr	argR		Arginine repr	14	2	2
A0A380DNV0	Membrane pr	NCTC6133_01327		Membrane pr	14	2	2
A0A8B1CKB8	Heme chaper	JYA46_00049		Heme chaper	8	2	2
A0A8B1CE92	Alpha/beta hy	JYA46_00222		Alpha/beta hy	8	2	2
A0A7K3MZ88	Glycosyltrans	GZ128_0143		Glycosyltrans	3	1	1
A0A659FLB6	Pathogenicity	E3K14_10720		Pathogenicity	8	2	2
Q2G0C4	Major facilitat	SAOUHSC_00681		Major facilitat	2	1	1
A0A0E0VL57	Uncharacteri	ST398NM01_0327		Uncharacteri	4	2	2
A0A1Q8DG2	S-adenosylm	queA	FUNCTION:	S-adenosylm	7	1	3
A0A831E1Y8	Putative TfoX	SAGV69_001		Putative TfoX	15	1	1
A0A6B5GZ45	DUF443 dom	G0Z42_09825		DUF443 dom	6	1	1
A0A6F9YV09	Bcr/CflA fami	JICS137_166		Bcr/CflA fami	4	2	2
Q53631	Replication p	rep		Replication p	8	2	3
A0A6B3J3E1	DUF1700 dom	G0004_13560		DUF1700 dom	11	1	2
A0A8E0EHB	Luciferase	SCAG_00801		Luciferase OS	5	2	2
A0A1Q8DHI1	Hydroxyacid	BSZ10_00220		Hydroxyacid	8	1	2
A0A1Q8DF45	Phosphatidat	BSZ10_05080		Phosphatidat	8	2	2
A0A6B5I4H3	GNAT family	G0V76_10995		GNAT family	5	1	1
Q2YZ91	Imidazoleglyc	hisB		Imidazoleglyc	11	2	2
A7X5L9	Urease subu	ureA		Urease subu	19	2	2
A0A6B5UDP	Amino acid p	G0X17_10500		Amino acid p	5	2	2
A0A6B5EUF1	Precorrin-2 d	G0W85_04660		Precorrin-2 d	17	1	2
A0A8E0EI29	Uncharacteri	SCAG_00409		Uncharacteri	20	2	2
A0A6B5DKI0	Exotoxin	sei		Exotoxin OS=	8	2	2
A0A6B0CTL8	GTP-binding	GO941_08470		GTP-binding	4	1	1
A0A8E0EE40	Iron complex	SCAG_02520		Iron complex	8	2	2
A0A7U8IAW2	Histidine kina	SABG_02472		Histidine kina	2	2	2
A0A1Q8DGR	YigZ family p	BSZ10_01560		YigZ family p	16	1	3
A0A2S6D5C0	Holliday junct	recU	FUNCTION:	Holliday junct	10	2	2
A0A5F0HPU8	Iron chelate u	E3A28_00695		Iron chelate u	3	1	1
A0A033UPC5	Uncharacteri	V070_02122		Uncharacteri	4	1	3
A0A380EK26	Bacteriophag	NCTC10702_03144		Bacteriophag	4	2	2
A0A7U7IBZ0	Uncharacteri	SAR0290		Uncharacteri	16	2	2
A0A6B3ISW5	Alanine racer	G0004_12965		Alanine racer	6	2	2

A0A2S6D9W	Phosphoribos	CSC87_17750		Phosphoribos	42	1	1
A0A1D4HUM	Holin	EDCC5055_01913		Holin OS=Sta	18	1	1
A0A1Q8DDI3	Bifunctional p	glmU	FUNCTION: (	Bifunctional p	2	1	1
A0A6B5F7V2	TIGR01741 fa	CV021_07205		TIGR01741 fa	17	0	3
A0A6N8IEQ8	CHAP domai	GO788_1313		CHAP domai	9	1	1
A0A6B5PTX2	UPF0346 pro	G0V60_10710		UPF0346 pro	18	1	1
A0A0E0VTK(	Uncharacteriz	ST398NM01_2710		Uncharacteriz	11	2	2
A0A641A7V2	Bifunctional tı	sbnI		Bifunctional tı	7	2	2
A0A8B1CZ76	50S ribosoma	rpmI		50S ribosoma	26	2	2
A0A6B0B376	YaiI/YqxD far	GO793_00905		YaiI/YqxD far	11	2	2
A0A6B0D5Z1	N-acetylmura	murQ		N-acetylmura	6	2	2
A0A6B2ITJ9	Uncharacteriz	GZ116_13250		Uncharacteriz	12	1	2
A0A6B5PVE8	Amino acid p	G0V60_10635		Amino acid p	20	1	3
A0A1Q8DCT	Metallophosp	BSZ10_09165		Metallophosp	6	1	1
A0A2S6DPC	Dihydroxy-ac	ilvD		Dihydroxy-ac	3	2	2
A0A6B5GS79	ABC transpor	G0Y66_09255		ABC transpor	6	1	1
A0A6B5FVX6	Uncharacteriz	G0Y65_13245		Uncharacteriz	9	1	2
A0A6B5F3F8	Ribose transp	rbsU		Ribose transp	5	2	2
A0A0H3JSC1	Truncated res	truncated-res		Truncated res	19	1	1
A0A8E0B353	Uncharacteriz	SCAG_00361		Uncharacteriz	7	1	1
A0A0E1VJ41	Methyltransfe	HMPREF0776_2626		Methyltransfe	12	2	2
A0A0E0VM17	Proton_antipo	ST398NM01_0705		Proton_antipo	3	1	1
A0A8B1CGJ1	Shikimate kin	aroK		Shikimate kin	10	2	2
A0A8G1GC6	DNA-binding	KSF82_1455		DNA-binding	9	1	1
A0A033UHL6	Branched-cha	V070_02233		Branched-cha	7	1	2
A0A033V3L0	Dihydrolipoar	V070_00558		Dihydrolipoar	3	1	2
A0A6B5KYX2	DUF4889 dor	G0V17_06785		DUF4889 dor	20	2	2
A0A8B1CIX0	Helix-turn-hel	JYA46_00100		Helix-turn-hel	6	1	1
A0A8E0EEP9	Large subuni	SCAG_00953		Large subuni	16	2	2
A0A8E8QSG	TetR/AcrR fa	KMZ21_1260		TetR/AcrR fa	9	2	2
A0A380EBB3	Membrane sp	NCTC10702_00407		Membrane sp	38	2	2
A0A033UTT5	ParB domain	V070_01710		ParB domain	5	1	2
A0A0E0VSM	Uroporphyrin	ST398NM01_2451		Uroporphyrin	8	3	3
A0A2S6DJJ8	AraC family tı	CSC87_14070		AraC family tı	5	2	2
A0A8E0DR89	Glycerophosp	SHAG_00689		Glycerophosp	5	3	3
A0A8E0DSW	Putative exot	SHAG_00892		Putative exot	7	1	1
Q2FV98	N-acetyltrans	SAOUHSC_02836		N-acetyltrans	11	2	2
A0A8B1CXK	tRNA dimethy	miaA		tRNA dimethy	7	2	2
A0A7U8XJX5	Binding-prote	SAXG_00703		Binding-prote	3	1	1
A0A8B1D7I9	VOC family p	JYA73_00099		VOC family p	10	1	1
D2KNZ6	Putative mem	traB		Putative mem	10	1	1
A0A7U4AU20	Phage portal	M013TW_14		Phage portal	2	1	1
Q6GA82	High-affinity h	isdE	FUNCTION:	High-affinity h	7	2	2
A0A1Q8DFA	Phosphoribos	purM		Phosphoribos	5	1	1
A0A0E1VMG	Uncharacteriz	HMPREF0776_1409		Uncharacteriz	12	2	2
A0A133Q0F7	Lipoprotein si	lspA	FUNCTION:	Lipoprotein si	16	2	2
A0A0E1XJ06	Uncharacteriz	HMPREF0769_11154		Uncharacteriz	27	1	1
A0A7U4ASC	DUF1806 fan	M013TW_05		DUF1806 fan	10	1	1
A0A8E0B1P8	Capsular poly	SCAG_00645		Capsular poly	10	1	1
A0A6B5HPC	DUF2129 dor	G0Z03_13355		DUF2129 dor	25	1	1

A0A8G2M9X	Transposon-r	NCTC7972_(		Transposon-r	6	1	1
Q9KW82	Coagulase (F	coa		Coagulase (F	14	1	1
Q6GHM0	Peptide defor	SAR1191		Peptide defor	15	2	2
A0A6B5I6N5	ABC transpor	G0Y40_04040		ABC transpor	5	2	2
A0A8G0JJC4	DUF4930 fan	JMO16_0880		DUF4930 fan	6	1	1
A0A8E0B251	Cation efflux	SCAG_00652		Cation efflux	5	1	1
A0A7Z1N8L1	Alcohol dehy	adhP		Alcohol dehy	15	1	1
A0A6G4IYN8	Sodium:proto	G0X02_1381		Sodium:proto	3	1	1
A0A033UY91	50S ribosoma	rplC	FUNCTION: (	50S ribosoma	10	1	2
A0A0U1MQX	Uncharacteri	BN1321_330021		Uncharacteri	19	1	1
A0A8E0B0Z0	N-acetylmura	SCAG_00368		N-acetylmura	6	1	1
A0A2S6DIZ0	Aldehyde deh	CSC87_15290		Aldehyde deh	6	1	1
A0A380DVP9	Membrane pr	NCTC6133_02486		Membrane pr	5	1	1
A0A1Q8DGP	Ribokinase	rbsK	FUNCTION: (	Ribokinase C	3	1	1
A0A5F0HM9	Hemolysin III	E3A28_11525		Hemolysin III	4	1	1
A0A0E1VKL1	Heme A synt	ctaA	FUNCTION: (	Heme A synt	3	1	1
D2J8Q5	Replication-a	repA		Replication-a	3	1	2
A0A6B0CI57	CDP-diacylgl	GO941_05750		CDP-diacylgl	18	2	2
A0A380E788	Histidine pho	gpmA_2		Histidine pho	10	2	2
D2JB55	Similar to Tei	SAP080A_031		Similar to Tei	5	2	2
A0A6B5APC	FRG domain-	G0W76_01860		FRG domain-	3	1	1
A0A1Q8DF0	Uncharacteri	BSZ10_05335		Uncharacteri	23	1	2
A0A8D9T487	Capsular poly	SAEG_00632		Capsular poly	4	1	1
A0A6B5GHK	DEAD/DEAH	G0Y66_04550		DEAD/DEAH	0	1	1
A0A033V772	LytR_cpsA_p	V070_00422		LytR_cpsA_p	5	1	2
Q6GJ90	Protein VraX	vraX		Protein VraX	27	1	1
A0A8E0EN7	Uncharacteri	SIAG_01700		Uncharacteri	6	1	1
A0A380EKR	CAAX amino	NCTC10702_03221		CAAX amino	9	1	1
A0A7U4ASX	Mnh complex	M013TW_08		Mnh complex	10	1	1
A0A1Q8DG1	Cell shape-de	BSZ10_0308	FUNCTION:	Cell shape-de	8	1	1
A0A8B1D4S	Y-family DNA	JYA73_0012		Y-family DNA	4	2	2
A0A0D1I6K8	Anthranilate s	QU38_13210		Anthranilate s	2	1	1
A0A1Q8DEY	Ferritin	BSZ10_0523	FUNCTION:	Ferritin OS=S	5	1	1
Q6GH70	30S ribosoma	rpsN	FUNCTION:	30S ribosoma	16	2	2
A0A033V3M	UPF0122 pro	V070_00260	FUNCTION:	UPF0122 pro	7	1	1
A0A380E0T3	Fatty acid bio	fapR		Fatty acid bio	12	2	2
A0A0U1MSA	Tagatose 1,6	lacD		Tagatose 1,6	5	2	2
A0A7Z1SCU	Gluconate pe	CV021_0539		Gluconate pe	8	1	2
A0A6B5EEE	Energy-coupl	ecfT		Energy-coupl	4	1	1
A0A7R6SNY	GCN5-relate	SAJPND4_0		GCN5-relate	11	1	1
A0A7Z8C27	DUF739 fami	E3A28_0204		DUF739 fami	12	1	1
A0A8G2I0H9	Superantigen	set14		Superantigen	5	1	1
A0A499S9T7	Replication p	BJL67_f0011		Replication p	17	1	2
A0A380DL20	Ribitol-5-pho	NCTC5664_00623		Ribitol-5-pho	35	1	1
A0A1Q8DCC	Mevalonate k	BSZ10_09920		Mevalonate k	5	1	2
A0A6B5DAN	Ring-cleaving	G0X68_00840		Ring-cleaving	3	1	1
A0A6B0A7M	Iron-regulate	GO651_13740		Iron-regulate	4	1	1
A0A0D1JV40	PTS glucose	QU38_14470		PTS glucose	5	1	1
A0A1Q8DFU	DUF2187 do	BSZ10_03545		DUF2187 do	28	1	1
Q6GIE0	Na(+)/H(+) ar	mnhE1	FUNCTION:	Na(+)/H(+) ar	5	1	1

A0A2S6DIY3	Carbamoyl pl	CSC87_15260		Carbamoyl pl	20	1	1
A0A2S6D9V1	Phosphopent	CSC87_17840		Phosphopent	14	1	1
A0A6B5G365	Signal peptid	lepB		Signal peptid	9	2	2
Q2G2G1	Uncharacteri:	SAOUHSC_00716		Uncharacteri:	10	2	2
A0A115DC26	Cation:dicarb	gltT		Cation:dicarb	2	1	1
A0A7R6P741	Uncharacteri:	SAJPND4_01		Uncharacteri:	9	1	1
A0A033UX85	Glyceraldehy	V070_00917		Glyceraldehy	5	1	2
A0A0A8IKE3	Putative mem	trsC		Putative mem	6	1	1
A0A8E0B0S0	Uncharacteri:	SCAG_00237		Uncharacteri:	19	1	1
A0A0E0VRK2	Putative mem	ST398NM01_2113		Putative mem	5	1	1
A0A6H4QG3	Siderophore :	iucC_1		Siderophore :	1	1	1
W8UY14	CcdC family		nasE		CcdC family		6	1	1
A0A7Z8GB18	Uncharacteri:	E4U00_0416		Uncharacteri:	20	1	1
A0A7Z8D9K7	EIIA	E4U00_0705		EIIA OS=Sta|	4	2	2
A0A033USB3	Uncharacteri:	V070_01571		Uncharacteri:	3	1	1
A0A6B5GWS	Uncharacteri:	G0Y66_11215		Uncharacteri:	10	1	1
A0A380EF83	Prophage L54	int3_1		Prophage L54	19	1	1
Q79LN3	Biofilm-assoc	bap		Biofilm-assoc	1	1	1
A0A6N8IBG5	Adenylosucci	purA		Adenylosucci	4	1	1
A0A1Q8DDZ	UPF0398 pro	BSZ10_07040		UPF0398 pro	4	1	1
Q2G2W0	Adenine DNA	SAOUHSC_0	FUNCTION: /	Adenine DNA	3	1	1
A0A6B5LYS3	YeiH family p	G0V76_00505		YeiH family p	2	1	1
A0A380DV01	HetN	NCTC6133_02008		HetN OS=Sta	6	1	1
A0A7Z1S9T3	Phenylalanine	pheS		Phenylalanine	13	1	2
A0A1Q8DFH	DNA-binding	BSZ10_04455		DNA-binding	9	1	2
G8I0X6	Uncharacteri:			Uncharacteri:	5	1	1
A0A7U7EXE7	Alcohol dehy	gutB		Alcohol dehy	6	2	2
A0A808JMR7	Putative hem	BTN44_1428		Putative hem	3	1	1
A0A8B1CY65	Uncharacteri:	JYA70_00235		Uncharacteri:	13	1	1
A0A6M1XCU	Signal recogr	G6X35_1218		Signal recogr	17	1	2
A0A6G4JEU0	Uncharacteri:	G0Y35_0278		Uncharacteri:	18	1	1
A0A6B5HVM	YSIRK-type s	G0X69_06125		YSIRK-type s	1	1	1
A0A6B0CU44	YibE/F family	GO941_02040		YibE/F family	4	1	1
A0A5A4RTZ7	Uncharacteri:			Uncharacteri:	12	1	1
A0A6M1XR9	2-oxoacid_dh	G6Y24_1470		2-oxoacid_dh	10	1	1
A0A033UPX0	UDP-N-acety	V070_02074		UDP-N-acety	2	1	1
A0A8G2M9H	Iron compour	yusV		Iron compour	9	2	2
A0A1Z3GB69	Mini-ribonucle	mrnC		Mini-ribonucle	7	1	1
A0A2S6DDZ0	Amino acid A	CSC87_16605		Amino acid A	16	1	2
A0A033UXT2	Uncharacteri:	V070_01382		Uncharacteri:	2	1	2
A0A1Q8DFK	Uncharacteri:	BSZ10_04515		Uncharacteri:	10	1	2
A0A8E0AY82	Lipoprotein	SCAG_02186		Lipoprotein C	7	1	1
A0A6B5I453	Branched-cha	brnQ		Branched-cha	4	2	2
A0A1Q8DCN	Antitoxin Maz	BSZ10_09395		Antitoxin Maz	16	1	1
A0A229LUZ8	Putative cyste	AS852_10330		Putative cyste	16	2	2
A0A2S6DD76	YeeE/YedE fa	C7P97_09715		YeeE/YedE fa	2	1	1
A0A8D9YT10	Uncharacteri:	SARG_00720		Uncharacteri:	4	1	1
A0A1Q8DD84	Carboxyleste	BSZ10_08220		Carboxyleste	4	1	1
A0A8B1D0M	Putative mem	yidD		Putative mem	12	1	1
A0A2S6DA54	Elongation fa	CSC87_17200		Elongation fa	18	1	1

A0A380DWK	Nitrate reduct	narI_1		Nitrate reduct	8	1	1
A0A844RB39	Lactonase dr	GO942_1204		Lactonase dr	4	1	1
A0A1I9S2X2	Recombinase	G6X35_06350		Recombinase	2	1	1
A0A8D9ZN16	Conserved do	SHAG_02081		Conserved do	11	1	1
A0A380DNS5	Putative DUF	NCTC6133_01342		Putative DUF	9	1	1
A0A380EMQ	Ribosomal-pr	NCTC10702_03251		Ribosomal-pr	10	1	1
A0A6B5FY10	L-2,3-diamino	sbnE		L-2,3-diamino	2	1	1
A0A0E0VQI8	Permease	ST398NM01_1383		Permease OS	8	1	1
A0A0E0VMH	ABC transpor	ST398NM01_0432		ABC transpor	5	1	1
Q2FZZ1	ABC transme	SAOUHSC_00843		ABC transme	3	1	1
Q6GEW7	ATP synthase	atpE	FUNCTION:	ATP synthase	10	1	1
A0A7U4AUW	Delta-hemoly	M013TW_19		Delta-hemoly	18	1	1
A0A6B0D5S1	Uncharacteriz	GO788_04540		Uncharacteriz	6	2	2
A0A1Q8DBD	Nitroreductas	BSZ10_11845		Nitroreductas	9	1	2
A0A033UB59	Aminopyrimic	V070_02801	FUNCTION:	Aminopyrimic	3	1	1
A0A0U1MVB	D-lactate deh	FDH		D-lactate deh	5	1	1
C4TFC5	FtsK/SpoIIIE			FtsK/SpoIIIE	2	1	1
A0A033UTI3	HTH marR-ty	V070_01525		HTH marR-ty	12	1	1
T1YCI5	Rod shape-de	SAKOR_02049		Rod shape-de	3	1	1
A0A033UZH1	Cysteine desu	V070_00681		Cysteine desu	4	1	1
A0A6G4IUA1	ABC transpor	G0X02_0702		ABC transpor	7	1	1
A0A380EP09	Na(+)/dicarbo	citT		Na(+)/dicarbo	6	1	1
A0A033UAJ6	5-dehydro-2-d	iolC	FUNCTION:	5-dehydro-2-d	2	1	1
A0A7U4AS18	Purine perme	M013TW_03		Purine perme	2	1	1
A0A0H3JKV9	Uncharacteriz	SA0424		Uncharacteriz	14	1	1
A0A8B1CJC1	Undecapreny	JYA46_00120		Undecapreny	3	1	1
A0A2S6D5G5	Aromatic acid	CV021_08000		Aromatic acid	2	1	1
A7X5E4	30S ribosoma	rpsZ	FUNCTION:	30S ribosoma	11	1	1
A0A8G2HZV	Aminobenzoy	abgT_2		Aminobenzoy	4	2	2
A0A5S9ET74	Uncharacteriz	TMSFP482_03780		Uncharacteriz	7	1	1
A0A033UDH9	NERD domai	V070_02739		NERD domai	2	1	1
A0A7Z1N0N4	Flavin reducta	CV021_1265		Flavin reducta	5	1	1
A0A380EC56	Capsular poly	capL_2		Capsular poly	8	1	1
E4PYE9	Conjugal tran	SUM_0019p2		Conjugal tran	2	1	1
A0A6B5M0T0	Putative hem	G0V76_05435		Putative hem	4	1	1
D0VDZ6	Cassette chro	ccrA		Cassette chro	2	1	1
A0A8E0AZW	Anthranilate s	SCAG_01714		Anthranilate s	6	1	1
A0A8H2KXY1	DUF309 dom	DQV20_1242		DUF309 dom	4	1	1
A0A6A9GWE	SACOL1771	GAY51_07245		SACOL1771	5	1	1
A0A850FYW	Uncharacteriz	GQX37_0796		Uncharacteriz	15	1	1
A0A7Z1S9N8	YycI domain-	CV021_1574		YycI domain-	5	1	1
A0A7I8NPC5	Regulatory pr	pfoS/R		Regulatory pr	3	1	1
A0A0U1MQH	Uncharacteriz	BN1321_330041		Uncharacteriz	8	1	1
A0A2S6DA49	Carbamoyl ph	CSC87_17210		Carbamoyl ph	18	1	1
A0A1Q8DGG	Putative N-ac	nanE	FUNCTION:	Putative N-ac	5	1	1
A0A7U4ASS9	CHAP domai	M013TW_07		CHAP domai	5	1	1
A0A8G2HW5	ESAT-6 famil	NCTC7972_0		ESAT-6 famil	11	1	1
A0A8D9ZFK0	Cation efflux	SDAG_00247		Cation efflux	5	1	1
A0A6M1XGC	Phosphopyru	eno		Phosphopyru	21	1	1
A0A380DRP1	Protein of unc	NCTC5664_0		Protein of unc	26	1	1

A0A7I8NJY3	Phage protei	SAMEA7024!		Phage protei	6	1	1
A0A1Q8DEM	RNA-binding	BSZ10_05795		RNA-binding	3	1	1
A0A1Q8DD5	Glucosamine	BSZ10_08510		Glucosamine	4	1	1
A0A033UDX	33 kDa chape	hslO	FUNCTION:	33 kDa chape	4	1	1
A0A0E1VKF	Uncharacteri:	HMPREF0776_1402		Uncharacteri:	9	1	1
A0A8G2HXK	KtrB	ktrB_2		KtrB OS=Sta	2	1	1
A0A033UZT	Tyrosine--tRI	tyrS	FUNCTION:	Tyrosine--tRI	3	1	1
M1XHV4	Histidine kina	kdpD		Histidine kina	1	1	1
A0A0E0VNA	ShaG	ST398NM01_0929		ShaG OS=St	8	1	1
A0A8B1CXG	Putative pyric	JYA70_0019		Putative pyric	3	1	1
A0A8D9W5E	Phage minor	SACG_0260		Phage minor	1	1	1
A0A6B5GFB	AAA domain-	G0W85_10650		AAA domain-	2	1	1
Q2FVB0	Uncharacteri:	SAOUHSC_02823		Uncharacteri:	3	1	1
A0A6B0AIK0	IDEAL domai	GO690_07795		IDEAL domai	11	1	1
A0A8E0EFR	Iron complex	SCAG_0106		Iron complex	3	1	1
A0A2S6D021	DUF1381 do	CV021_18340		DUF1381 do	13	1	1
A0A0C2HXZ	Uncharacteri:	BN1321_190005		Uncharacteri:	15	1	1
I7GY16	Integrase	int		Integrase OS	2	1	1
A0A6G4N2A	Transcription	G0Y31_0192		Transcription	9	1	1
A0A146HSH	Immunoglobu	spa		Immunoglobu	14	1	1
A0A844QT47	Alkaline shoc	GO782_1264		Alkaline shoc	4	1	1
A0A1Q8DAY	Glycine cleav	gcvH	FUNCTION:	Glycine cleav	19	1	1
A0A033US91	PTS EIIB typ	V070_01616		PTS EIIB typ	2	1	1
A0A380DJQ	Cytosolic pro	NCTC5664_00024		Cytosolic pro	10	1	1
A0A853PBS1	Antiporter	A6762_0300		Antiporter OS	7	1	1
A0A5F0HKL	DUF4064 do	E3A28_07240		DUF4064 do	5	1	1
D2J7N0	Uncharacteri:	SAP030A_022		Uncharacteri:	2	1	1
A0A2I7Y937	DNA-directec	dnaE_2		DNA-directec	1	1	1
A0A6B0CEU	Glyceraldehy	gap		Glyceraldehy	5	1	1
A0A0D1JGB	EIIA	QU38_14135		EIIA OS=Sta	6	1	1
A0A6G4JLD	Oligoendope	G0Y35_1406		Oligoendope	3	1	1
A0A6B5ICE9	Uncharacteri:	G0V76_12920		Uncharacteri:	31	1	1
A0A380EJZ8	Amino acid p	steT_1		Amino acid p	3	1	1
A0A7U4ARZ	Lysostaphin	M013TW_01		Lysostaphin	4	1	1
A0A033UQY	Coproporphy	V070_01970	FUNCTION:	Coproporphy	2	1	1
A0A380DPP	Iron(III) dicitr	yhfQ_1		Iron(III) dicitr	3	1	1
A0A6N8I8K6	30S ribosom	rpsH		30S ribosom	5	1	1
A0A0D1K5L	Uncharacteri:	QU38_00240		Uncharacteri:	11	1	1
Q6GCL0	Antiholin-like	lrgA	FUNCTION:	Antiholin-like	5	1	1
A0A0D1K3U	Uncharacteri:	QU38_02635		Uncharacteri:	5	1	1
S0DH59	Mobilization	mob		Mobilization	2	1	1
A0A033UX2	Aldehyde del	V070_01536		Aldehyde del	4	1	1
A0A033UIP3	3-hexulose-6	V070_02167		3-hexulose-6	7	1	1
A0A6B5ELQ	Na+/H+ antip	G0Y58_13785		Na+/H+ antip	2	1	1
A0A033UYT	Uncharacteri:	V070_00806		Uncharacteri:	7	1	1
A0A8D9YU9	Uncharacteri:	SARG_0068		Uncharacteri:	3	1	1
A0A3A5LQI3	Putative mRI	DQV20_01750		Putative mRI	8	1	1
A0A033UYE	HTH gntR-typ	V070_01214		HTH gntR-typ	3	1	1
A0A0H3JVB	CzrB protein	czrB		CzrB protein	3	1	1
A0A6G4N5X	Uncharacteri:	G0Y31_0830		Uncharacteri:	6	1	1

A0A8E0AXP7	Uncharacteriz	SCAG_02361		Uncharacteriz	9	1	1
A0A0D1HYB	Uncharacteriz	QU38_01915		Uncharacteriz	5	1	1
A0A6B5HCX	Capsular bios	CV021_03870		Capsular bios	3	1	1
A0A033UTS2	ThuA domain	V070_01496		ThuA domain	7	1	1
A0A6B5I0C0	Sirohydrochlo	G0X69_06950		Sirohydrochlo	5	1	1
A0A6B0BLN7	NitT/TauT fan	GO793_00470		NitT/TauT fan	9	1	1
A0A0U1MQK	Putative DNA	BN1321_330049		Putative DNA	2	1	1
A0A6B5G9C9	GNAT family	G0W85_10670		GNAT family	4	1	1
A0A7Z1MZ31	Uncharacteriz	CV021_1747		Uncharacteriz	5	1	1
A0A6K2RPY0	Uncharacteriz	SALFYP88_0		Uncharacteriz	18	1	1
A0A0D1IM29	Uncharacteriz	QU38_02315		Uncharacteriz	4	1	1
A0A8F8TI38	ABC transpor	KXJ66_13665		ABC transpor	5	1	1
U3U9Y8	Partitioning p			Partitioning p	4	1	1
A0A8E0ED14	Uncharacteriz	SCAG_02363		Uncharacteriz	18	1	1
A0A1Q8DBE	Uncharacteriz	BSZ10_11755		Uncharacteriz	2	1	1
F8WK72	Site-specific I			Site-specific I	1	1	1
A0A7X3FDQ	Quaternary a	qacC		Quaternary a	7	1	1
A0A5S9I325	Uncharacteriz	TMSFP482_07500		Uncharacteriz	5	1	1
A0A0D1HYH	Uncharacteriz	QU38_01655		Uncharacteriz	5	1	1
A0A7Z8GDC	Rod shape-de	mreD		Rod shape-de	5	1	1
A0A8E0BH29	Cationic amir	SAZG_00558		Cationic amir	3	1	1
A0A033UZK5	Uncharacteriz	V070_01077		Uncharacteriz	5	1	1
A0A8D9YUP	Uncharacteriz	SASG_01378		Uncharacteriz	1	1	1
A0A1Q8DDT	Pyruvate dec	BSZ10_07320		Pyruvate dec	3	1	1
A0A8D9SLE4	Phage protein	SAAG_02485		Phage protein	10	1	1
A0A0D1HYF4	GDSL family	QU38_01740		GDSL family	8	1	1
A0A8F2XD33	DUF1542 dor	KRH49_0897		DUF1542 dor	1	1	1
A0A033UB69	Monofunction	mgt	FUNCTION:	Monofunction	3	1	1
A0A6M1XBK	Amino acid A	G6X35_0988		Amino acid A	7	1	1
Q2G0Z3	Uncharacteriz	SAOUHSC_00368		Uncharacteriz	24	1	1
A0A6B1RX00	Uncharacteriz	GAY51_11545		Uncharacteriz	6	1	1
A0A6B0BZ89	30S ribosoma	rpsK		30S ribosoma	12	1	1
Q4JF93	Xylose opero	xylr		Xylose opero	3	1	1
G8H1P3	Coagulase (F	coa		Coagulase (F	4	1	1
A0A454H0Y6	ThrE domain-	C7P97_05270		ThrE domain-	4	1	1
A0A0U1MRH	Hypoxanthine	pbuG		Hypoxanthine	2	1	1
A0A0D1K3P2	Uncharacteriz	QU38_02680		Uncharacteriz	4	1	1
A0A1Q8DBF	Uncharacteriz	BSZ10_11670		Uncharacteriz	4	1	1
A0A6B5IW15	Relaxase/mo	G0Y40_11985		Relaxase/mo	2	1	1
A0A7U9J4F0	Resolvase pr	SA1_75329		Resolvase pr	2	1	1
A0A844QR30	50S ribosoma	rplJ		50S ribosoma	7	1	1
A0A6B3IMK0	Site-specific i	G0004_06330		Site-specific i	2	1	1
A0A0Z0U4M8	AAA family A	dnaA_1		AAA family A	3	1	1
Q2YYJ5	Tagatose-6-p	lacC		Tagatose-6-p	2	1	1
A0A1Q8DC5	DoxX family p	BSZ10_10255		DoxX family p	6	1	1
A0A2C9TY88	DNA polymer	dinB	FUNCTION:	DNA polymer	2	1	1
A0A1Q8DFN	LysR family tr	BSZ10_04120		LysR family tr	4	1	1
A0A033V776	Immunodomi	V070_00236		Immunodomi	1	1	1
A0A6B0CU09	Uncharacteriz	GO941_13060		Uncharacteriz	10	1	1
A0A1Q8DE09	AI-2E family t	BSZ10_06695		AI-2E family t	2	1	1

A0A0H3JPI0	PseudoU_syı	SA0866		PseudoU_syı	2	1	1
A0A033UEH(	Allantoinase	allB	FUNCTION: (	Allantoinase (	2	1	1
A0A8G2I087	Outer surface	NCTC7972_(		Outer surface	3	1	1
A0A5S9I4W4	N-acetyltrans	TMSFP482_21540		N-acetyltrans	3	1	1
Q93IE5	ORFID:MW0	SAMEA70146418_01605		ORFID:MW0	2	1	1
A0A033UPE3	Uncharacteri:	V070_01816		Uncharacteri:	1	1	1
A0A1Q8DBH	Transcription	BSZ10_11520		Transcription	8	1	1
A0A033V082	Uncharacteri:	V070_00960		Uncharacteri:	2	1	1
A0A844QP71	50S ribosoma	rplL		50S ribosoma	7	1	1
A0A1Q8DDY	3-phosphosh	aroA	FUNCTION: (	3-phosphosh	4	1	1
A0A0D1IMX(	Uncharacteri:	QU38_01255		Uncharacteri:	5	1	1
Q53749	Replication p	rep		Replication p	5	1	1
A0A1Q8DDH	Cystathionine	BSZ10_07950		Cystathionine	2	1	1
A0A6B5I713	Ornithine ami	rocD		Ornithine ami	2	1	1
A0A6N3EHJ1	Indole-3-glyc	trpC		Indole-3-glyc	3	1	1
A0A830YSM	Uncharacteri:	SAGV69_02(		Uncharacteri:	4	1	1
A0A8D9SLX3	Phage proteiı	SAAG_02101		Phage proteiı	8	1	1
A0A0D1K0F5	Uncharacteri:	QU38_00540		Uncharacteri:	5	1	1
A0A033USL5	Polyphosphat	V070_01828		Polyphosphat	2	1	1
A0A7H9CF57	Uncharacteri:	SA950122_0:		Uncharacteri:	28	1	1
A0A6H3QFK	YozE_SAM_l	DD547_0264		YozE_SAM_l	10	1	1
M9UUA3	Uncharacteri:			Uncharacteri:	3	1	1
A0A6G4IR25	Nickel ABC tı	G0X02_0043		Nickel ABC tı	1	1	1
A0A8G2M7I5	FemC, factor	femC		FemC, factor	7	1	1
A0A6M1XR2	Uncharacteri:	G6Y24_0774		Uncharacteri:	10	1	1
A0A8B1CPT3	Polysaccharic	JYA66_0018:		Polysaccharic	1	1	1
A0A033UQK	ADP-depende	nnrD	FUNCTION: (	ADP-depende	4	1	1
A0A6B0BC09	Uncharacteri:	GO793_06305		Uncharacteri:	2	1	1
A0A033UP92	Imidazole gly	hisH	FUNCTION:	Imidazole gly	4	1	1
P63952	Histidinol deh	hisD	FUNCTION: (	Histidinol deh	2	1	1
A0A6B5L3S(	Uncharacteri:	G0W85_02525		Uncharacteri:	10	1	1
A0A033UTY4	EIIA	V070_01468		EIIA OS=Sta	1	1	1
A0A033UTU2	Ribonuclease	rnpA	FUNCTION:	Ribonuclease	6	1	1
A0A6J9VNF2	Arginine-ornit	arcD		Arginine-ornit	2	1	1
A0A033UM4:	Cytosine-spe	V070_02173		Cytosine-spe	3	1	1
A0A380DTS2	Mobile eleme	NCTC5664_01972		Mobile eleme	5	1	1
A0A033V168	Fe/B12 peripl	V070_00876		Fe/B12 peripl	2	1	1
A0A6N8I8H7	YggS family p	GO788_0613		YggS family p	3	1	1
A0A7Z1N3W	AP_endonuc_	CV021_0634		AP_endonuc_	5	1	1
A0A7Z8DDS:	AAA_5 doma	E4U00_0839		AAA_5 doma	1	1	1
A0A033UD42	Probable Ctp	V070_02758		Probable Ctp	1	1	1
A0A1Q8DDW	GTP-binding	BSZ10_07120		GTP-binding	2	1	1
A0A6B5Q153	Putative mRN	G0Z61_03795		Putative mRN	8	1	1
Q5PU79	Tetracycline (	tet38		Tetracycline (	2	1	1
A8Z150	Putative antip	mnhG2		Putative antip	5	1	1
A0A7I8NMJ9	Conjugative t	SAMEA7024!		Conjugative t	1	1	1
A0A3Q8H2T(	VirB4 family (	detF		VirB4 family (	1	1	1
A0A8E0AYW	Secreted von	SCAG_0120‹		Secreted von	2	1	1
I7HAS4	Pahtogenicity	SA950122_02529		Pahtogenicity	2	1	1
A0A0D1IMK(	Uncharacteri:	QU38_01705		Uncharacteri:	5	1	1

A0A6B0B8Y3	Uncharacteri:	GO793_06315		Uncharacteri:	4	1	1
A0A7Z1SEX9	Transglycosy	CV021_0082		Transglycosy	6	1	1
A0A8E0DR34	Cystathionine	SHAG_00827		Cystathionine	2	1	1
A0A0E0VR38	EIIA-Lac	ST398NM01_2251		EIIA-Lac OS=	8	1	1
D0EVA0	Vga(A)LC			Vga(A)LC OS	1	1	1
A0A1Q8DGJ	Dihydrolipoar	BSZ10_02265		Dihydrolipoar	2	1	1
A0A033UYN1	Peripla_BP_4	V070_01197		Peripla_BP_4	3	1	1
A0A6B0ATW	Uncharacteri:	GO690_03175		Uncharacteri:	1	1	1
A0A2X2KJE1	DNA primase	NCTC7878_03467		DNA primase	5	1	1
A0A033V6A8	Uncharacteri:	V070_00178		Uncharacteri:	8	1	1
A0A6B5IA92	Type VI secre	tssG		Type VI secre	3	1	1
A0A7Z1N116	Tagatose-6-p	lacC		Tagatose-6-p	2	1	1
A0A2S6D2Y7	Inactivated su	CV021_12800		Inactivated su	1	1	1
A0A8E4FYV7	Phage tail pro	AS852_0938		Phage tail pro	1	1	1
A0A898CKL4	EIICB-Lac	JX572_1128		EIICB-Lac OS	3	1	1
A0A8E0EF77	Glutamate sy	SCAG_00917		Glutamate sy	1	1	1
A0A033UD63	DUF418 dom	V070_02745		DUF418 dom	2	1	1
A0A8D9ZR37	DltB protein	SHAG_0221(		DltB protein (	2	1	1
A0A2C9TQV	Adenylosucci	EP54_15245		Adenylosucci	9	1	1
Q9F0J7	Integrase-like			Integrase-like	2	1	1
Q2G1M6	2-[(L-alanin-3	sbnH	FUNCTION:	2-[(L-alanin-3	6	1	1
A0A380DTR9	Nickel transp	gsiD_3		Nickel transp	29	1	1
A0A2X2K2M8	Dihydroxyace	NCTC7878_01899		Dihydroxyace	8	1	1
A0A2S6DA24	Lysine--tRNA	CSC87_17410		Lysine--tRNA	10	1	1
A0A811I7Y4	Phage protein	SAMEA7014(		Phage protein	2	1	1
A0A6B5LY34	ABC transpor	G0V76_10040		ABC transpor	2	1	1
A0A6M1XDG	EIIA-Lac (Fra	G6X35_1103		EIIA-Lac (Fra	29	1	1
A0A033V369	Uncharacteri:	V070_00787		Uncharacteri:	1	1	1
A0A6B2IWU7	Persulfide dic	cstB		Persulfide dic	5	1	1
A0A6B5R91(	Immunodomi	G0V60_05820		Immunodomi	1	1	1
A0A033V238	Uncharacteri:	V070_00493		Uncharacteri:	8	1	1
A0A033V0M2	Nucleotidase	V070_00611		Nucleotidase	7	1	1
A0A6B5LQN1	ATP-depende	CV021_08165		ATP-depende	2	1	1

PSMs	AAs	MW [kDa]	calc. pI	Ctrol-01	Ctrol-02	Ctrol-03	Treat-01
1493	1257	137.4	9.61	1.53962077	1.06449295	1.38096688	0.73224457
1208	749	84.8	5.48	1.50599276	1.28942082	1.4255164	0.71573819
583	1207	135.3	6.95	0.93114134	0.82738564	0.90880301	1.08969445
1289	1259	137.6	9.61	1.21130395	1.15229142	1.21568571	0.83800285
1392	1254	137	9.61	1.12996578	0.7102866	0.97929127	1.11989881
1214	1254	137.1	9.6	1.51141617	1.23447985	1.47674817	0.6370306
1287	1259	137.7	9.6	1.24135163	1.30087833	1.19844449	0.7902454
1344	1248	136.6	9.6	1.29356621	1.83609779	1.2898211	0.63325919
1277	1255	137.5	9.61	1.50537107	1.0459685	1.34929813	0.73592083
1272	1256	137.3	9.61	1.02068255	0.9638064	1.10026627	0.96756494
848	693	76.6	4.88	1.03484822	0.93195961	1.02311731	1.00250318
322	1374	148.8	4.39	0.96198243	1.03584724	1.41808294	0.95267141
655	901	98.9	5.02	0.59939582	0.59594522	0.6060883	1.38759178
435	1183	133.2	5.03	0.9239725	0.8446047	0.89543778	1.10322483
1327	394	43.1	4.86	1.03742619	1.01273401	0.97807501	1.01663286
274	1150	128.5	5.25	1.27170886	1.09828186	1.27559448	0.83067644
1767	516	56.3	5.72	1.56219349	1.6024517	1.31390356	0.63016067
646	580	64	4.79	0.88856819	0.9084318	0.95439831	1.1145398
441	818	91	5.78	0.82512413	0.79240573	0.81636297	1.16694465
443	818	91	5.83	0.70885794	1.22901889	0.8311028	1.05888911
459	818	91	5.73	0.77454114	1.07514698	0.9516836	1.03648367
293	433	46.4	5.03	1.16775495	1.1011703	1.12235086	0.91849839
511	662	72.3	5.03	1.04811017	0.95761549	0.96357869	1.02828941
531	538	57.6	4.65	0.92286984	0.89454422	0.92200873	1.07222065
263	932	105.3	5.66	0.69160113	0.64375861	0.71150068	1.29668377
303	843	96	5.21	1.03060555	0.92235557	0.97117264	1.05029166
465	662	72.3	5.06	0.91392874	1.04192266	0.93446765	1.05441492
657	514	56.8	5.07	0.77226572	0.66246058	0.75616543	1.26242343
397	588	65.5	5.63	0.72204704	0.69164988	0.72836182	1.26580755
329	1183	133.2	5	0.91870259	1.02466125	1.00528157	0.98235513
1505	312	35.1	4.94	1.19252716	1.45716106	1.26455183	0.75267051
409	585	63.1	5.3	1.0454355	0.98649681	1.05139105	0.98689154
465	400	44.1	6	1.07901334	1.07762629	1.08844433	0.93942576
644	325	35.3	4.73	0.87988249	0.87634204	0.86332835	1.11451856
417	539	57.8	4.75	0.87664831	1.17059955	1.01173035	0.89056235
408	390	41	4.98	1.02656558	1.01393883	1.04239493	0.99662497
356	610	66.3	4.78	0.99733015	0.86669478	0.92054474	1.0822301
267	1057	117.1	4.97	1.67020838	1.31413746	1.54541782	0.58171893
224	869	98.2	5.05	1.04287098	1.13321584	1.0298442	0.95866891
233	721	82.6	5.52	0.9884981	0.91027403	0.99406016	1.04663752
1357	484	52.9	5.8	1.32347986	1.24331493	1.12212658	0.8007366
388	470	51.4	4.82	1.14340276	0.97739439	1.07156315	0.95961247
222	984	111.3	5.24	0.95339885	0.94919088	0.97200226	1.05481349
452	693	76.7	4.89	1.06813014	1.17731036	1.05012776	0.93788179
501	304	34.2	5.67	1.55665301	1.03642231	1.28951249	0.77931663
672	514	56.8	5.14	0.78261754	0.96709142	0.83087744	1.12224649
229	701	80.1	5.4	1.05096277	1.5254366	1.21993639	0.70416373

272	463	53.6	5.12	1.14565558	1.00669242	1.16502293	0.92587711
373	391	43.3	4.59	1.00727338	0.9515158	0.98628311	1.0276005
239	932	105.3	5.66	0.48441478	2.04101727	0.83132817	1.01363285
395	645	74.4	5.39	1.4455251	1.20696025	1.39489497	0.71127557
1362	507	55.4	5.71	1.15391074	1.39153301	1.20125188	0.73553995
1416	362	40.6	6.25	1.26319754	1.56001058	1.39619121	0.68128922
396	495	53.6	5.22	0.61488144	0.52926607	0.60589707	1.37270295
920	394	43.1	4.83	0.92655628	0.94948143	0.93838016	1.02927128
253	917	104.8	5.44	0.9769015	0.89160707	0.96421291	1.05143457
186	701	77.9	5.14	1.43125292	1.60101381	1.40313776	0.6387164
197	876	98.5	5.14	1.0390115	1.02295044	1.04033861	0.9751997
243	917	104.8	5.47	1.0065357	0.92347237	0.96423479	1.0153977
335	469	52.8	4.74	1.06349661	1.0304117	1.03577953	0.9733668
337	366	41.8	8.44	1.2426863	0.91167926	1.1886755	0.92737805
1307	516	56.4	5.54	1.46967415	1.07893795	1.15642843	0.82193727
464	507	58.6	5.6	0.93703999	0.81223913	0.91211195	1.15744366
394	491	53.2	5.88	1.24660902	1.09447545	1.28129022	0.84757839
458	498	56	6.55	1.26149064	0.9049078	1.12958119	0.95566748
166	978	114.4	5.35	0.99921812	0.99380404	0.98238156	1.02479842
1244	467	51.1	5.22	1.58205454	1.87789287	1.36232748	0.60051596
313	469	52.8	4.7	1.12104963	0.95099993	1.09946453	0.94905933
326	475	53.6	5.14	0.97471517	0.84054255	0.91286792	1.08690669
336	443	49.8	4.91	1.10350679	0.78988999	0.99461193	1.05549936
103	10611	1142	6.29	1.15815026	1.26768068	1.13765367	0.84946455
205	722	81.4	5.06	1.04110029	1.14844756	0.92322771	0.98784724
743	293	32.5	5.12	0.99187394	0.87540143	0.91633859	1.09914852
313	469	52.8	4.7	1.23062313	0.98958616	1.06969403	0.95656005
659	434	47.1	4.65	1.06949596	0.95729925	1.01086307	0.98622875
299	391	43.3	4.6	0.91654255	1.18905712	1.01823971	0.92240127
188	876	98.5	5.12	1.18441284	0.94434488	1.05647928	0.97521819
260	713	78.8	5.2	1.00078676	0.91874049	1.00762393	1.03430224
165	876	101.7	5.1	1.06134107	0.92390068	1.01983017	1.01698523
428	411	46.9	5.25	1.85980571	1.37902389	1.6389732	0.48290442
373	296	32.9	5.06	0.98929083	0.92263487	1.00249744	1.05634691
476	430	46.4	5	0.81218945	0.77812495	0.78192692	1.19751341
394	507	54.6	4.82	0.91164927	0.7822436	0.8893289	1.15903104
366	400	44	6	1.04450616	1.11449437	1.16229999	0.9356053
722	468	49.4	5.02	0.79083058	0.75929527	0.76682756	1.23937229
423	495	53.6	5.27	0.61684161	0.64563787	0.70699695	1.27425462
496	388	42.1	4.98	0.94678173	0.85830987	0.94144402	1.07964092
324	395	42.8	5.29	0.97064618	0.86339852	0.93402765	1.10638202
158	978	114.3	5.36	0.87728667	1.14392914	0.77361038	1.06921203
135	729	79.5	4.93	1.60867922	1.26321714	1.50754787	0.62223045
409	555	59.8	6.04	0.88002459	0.80627254	0.85086646	1.1654988
256	1205	134.8	7.06	1.22812789	1.07707194	0.98641827	0.98980163
135	876	99.1	5.17	0.91341137	0.89624643	0.92092991	1.08550358
579	434	47.1	4.65	1.1071898	0.8877311	0.99991974	1.00796611
1156	484	52.9	5.67	1.23984158	1.36407046	1.27677288	0.71694941
1283	351	39.3	5.47	0.89874669	0.75284851	0.88024856	1.14588293
244	485	52.8	5.15	0.91971886	0.82559576	0.9229663	1.09412154

208	697	77.8	5.58	1.01760023	0.93424086	0.94844634	1.02678242
317	557	62.4	6.83	0.87190295	0.81872175	0.86681228	1.1077385
252	358	40	4.89	1.23685462	1.16799659	1.25004505	0.83772057
211	300	33.1	5	0.94224119	0.83447957	0.92140425	1.10863105
199	530	59.4	6.11	1.1419262	1.00202735	1.12446315	0.94991272
1229	360	39.4	5.48	1.05146028	0.91365032	1.12799273	1.00001844
155	619	69.2	6.3	0.89902145	0.91095657	0.87979792	1.07259315
181	843	95.2	5.02	0.98575798	1.01241552	1.03616546	0.97313521
1220	454	49.3	5.91	1.41405195	2.07938207	1.32405834	0.56225397
243	601	65.8	5.03	1.14975478	1.10010331	1.14026605	0.90002672
593	370	41.4	5.05	0.84637816	0.70797328	0.79424577	1.20870415
145	727	80.4	8.73	1.12933034	0.85107831	1.0041271	1.03870998
302	475	53.6	5.17	0.86239814	1.1053207	0.97749067	1.02144706
209	396	43.5	9.58	0.94593105	0.77058673	1.05983363	1.07900427
1222	354	39.7	5.47	0.84581622	1.05192589	0.98273342	1.03069886
381	507	54.7	4.82	1.01221516	1.29552871	1.06256057	0.94089937
168	484	56.3	5.31	1.01519738	0.995177	1.02130626	0.99885992
329	310	33	5.45	1.23802312	1.03085239	1.19872564	0.9245099
252	490	54.8	5.59	0.76967586	0.70642287	0.77085389	1.24228633
309	443	49.8	4.93	1.00455185	1.23813687	1.01860452	0.81203804
229	564	66.2	4.87	1.03989739	0.90953632	1.00799132	1.01386534
121	948	105.3	6.44	0.90640601	0.87420201	0.86147528	1.10329143
215	553	62.3	5.22	0.96192402	1.06669098	0.99086326	0.993935
206	495	56.7	5.25	1.02617491	0.99600116	1.04041555	0.99603157
436	505	58.3	5.54	1.074615	1.04457224	1.078898	0.95986275
614	336	36.3	5.05	1.00944531	0.83998542	0.9708573	1.05435448
219	372	40.1	5.99	0.78665416	0.74276546	0.7720164	1.20363959
461	396	43.4	5.53	0.68458214	0.72563925	0.7124257	1.25754306
207	363	40.4	4.93	0.72460034	0.59796135	0.70557069	1.30411061
277	381	42.8	5.38	1.25168244	1.02667042	1.13394042	0.93348916
202	495	56.9	5.34	1.13605056	0.92752603	1.02002038	1.00667879
335	296	33	4.94	1.03133772	1.09398863	1.02231896	0.97472585
119	1229	139.8	6.21	1.28894907	1.25410967	1.25153702	0.79926731
200	372	40.1	6.01	1.18656091	1.22210805	0.98620756	0.90566742
270	498	55.6	5.12	0.915913	0.83782068	0.88475072	1.11469296
283	388	44.1	4.96	1.01132317	0.9383108	1.01699602	1.01014188
297	395	42.9	5.44	0.92631899	1.21076657	1.01686385	0.96585696
313	414	45.7	5.29	0.79707573	0.66909405	0.78143035	1.24151121
289	286	30.8	5.1	1.13023997	1.10636401	1.11016695	0.92982684
170	448	49.7	5.2	0.65537136	0.57564134	0.64348983	1.3165622
584	370	41.4	5	0.78613598	0.91816378	0.87476076	1.13163749
175	475	52	5.17	1.05356978	0.94158963	1.0534328	0.99786557
209	468	51.8	5.14	1.06937329	0.86744402	1.01431878	1.03016269
179	654	76.2	6.43	0.96538948	0.85241262	0.94458458	1.09737414
310	310	33	5.45	1.07351333	1.32560972	1.22289984	0.85804346
193	432	49.4	5.4	0.98132305	0.93251859	1.01179661	1.0268415
136	804	91.6	5.14	0.99056878	1.07350348	0.97855209	1.01623737
714	396	43.3	4.79	1.02304551	0.93052065	1.00960639	1.03196239
164	546	60.5	5.21	0.97272195	0.92232786	0.97908093	1.05380499
262	373	42.6	5.6	0.52340208	0.61065319	0.54741497	1.3826735

216	602	69.8	5.26	1.04288805	0.92954125	1.03056948	1.0129063
96	800	88.9	4.83	1.02892551	0.94488888	0.9312003	1.0393272
131	667	75	5.24	0.91304224	0.86046805	0.85330239	1.11921206
140	588	66.6	5.1	1.12514873	1.08627139	1.16760193	0.88887945
181	698	77.3	4.97	1.00854896	0.96247164	1.0193397	0.99940631
148	318	35.6	4.83	0.92188123	1.10291797	0.92012242	1.0273156
152	538	63.8	4.58	0.89904438	0.89184881	0.91218814	1.10989403
170	646	74.4	9.04	0.73988022	0.77326488	0.7510091	1.24726179
1145	233	26.2	5.06	1.13099134	1.48762653	1.2754379	0.78957949
1047	233	26.3	4.98	1.04603598	1.25464867	1.0865614	0.87910261
440	283	32.8	5.77	1.35152237	1.22607999	1.38266687	0.75625564
144	513	58.2	5.12	1.07838836	1.10156214	1.14964265	0.89164459
259	468	52.9	5.12	0.88747746	0.99155361	0.90160824	1.0680355
247	423	46.8	5.01	0.71666152	0.62733364	0.69078665	1.30538652
89	1188	136.5	5.47	0.95191486	0.91885811	0.96849336	1.03318742
470	199	22.7	5.19	0.47173636	0.40084754	0.45675761	1.5102743
146	507	57.9	7.84	0.90733064	0.82626331	0.9686859	1.12616466
126	655	76	4.93	0.94958656	1.21511021	1.20074666	0.85408066
73	10544	1134.6	6.29	0.92800116	1.52343189	1.07339004	0.84272752
109	801	88.6	5.39	1.213943	1.22569362	1.16656593	0.84047528
146	567	63.8	5.21	1.0593802	0.90356425	1.03604395	0.99646018
183	446	50.8	5.2	1.11410948	1.03597784	1.13600808	0.9151146
265	428	48.6	5.11	1.36611853	1.05536795	1.22337402	0.81977182
113	574	66.3	5.03	0.89520249	0.97031466	0.88006418	1.09716979
100	1274	144.4	5.19	1.00760236	1.02277262	0.99754101	1.00791087
194	433	48.6	4.42	1.04121789	0.93336952	0.953738	1.03984297
337	396	43.4	5.44	0.61542478	0.76459521	0.71241041	1.21444761
255	273	30.4	5.45	1.01190596	0.90048021	0.99095023	1.01884919
262	338	37	5.03	1.05080631	0.77525474	0.94788112	1.00043529
158	886	99.2	5.14	0.93619768	0.96077857	0.9381927	1.05342911
178	372	40.1	5.85	0.70534786	0.64494442	0.80018429	1.28021424
160	479	56.8	4.84	1.19062349	1.03005023	1.15602325	0.90987732
302	319	34.4	4.97	1.5661875	1.44064376	1.52527508	0.60860034
130	568	64.4	5.16	0.75184785	0.86569541	0.76156446	1.1996874
233	572	63.3	4.75	1.0617189	0.93579733	0.99979712	1.00518222
231	470	51.5	4.74	1.23702563	0.85904042	0.99945309	0.99982269
114	701	77.8	4.98	1.32332274	2.42238139	1.42123125	0.43975639
280	295	32	5.19	0.99913375	1.03159765	1.03519042	0.98977261
98	1153	125.3	4.46	0.93365948	1.19037924	1.2379913	0.92152414
117	730	84	5.86	0.84120323	0.85743403	0.84596979	1.12637003
295	437	50.2	9.42	1.24372461	1.0422273	1.3842988	0.80461578
138	533	60.1	4.96	1.01446726	1.02310838	1.03625427	0.96259665
610	293	32.5	5.17	0.90830037	1.20110694	1.02819707	1.01305936
471	422	46.5	4.98	0.557674	0.50595862	0.55048665	1.39481846
113	804	91.6	5.14	0.8527879	1.06075664	0.95951557	1.02020465
83	800	88.9	4.87	0.9578459	1.12722483	1.09299998	0.93591129
178	657	74.8	5.22	1.02441061	0.84279204	0.98580868	1.03931302
150	591	67.5	5.25	1.04598424	1.4520852	1.17627537	0.81452726
193	220	23.7	9.8	0.86183755	0.83909697	0.84883626	1.11578041
206	273	30.4	5.59	0.87547264	1.13481347	0.94670604	1.00731238

806	327	35.7	5.49	1.22047653	1.44716148	1.19805938	0.72503241
220	309	35.3	8.87	0.77215626	0.81997854	0.72745846	1.2291693
161	644	72.5	5.88	0.97140616	0.93398912	0.94712545	1.06694849
267	296	32.9	5.11	1.02729592	1.06938236	1.02957658	0.93405046
469	282	30.3	4.92	0.84564539	1.6028988	0.99398273	0.88912467
178	565	62.6	7.47	1.00751479	0.91959513	1.00729853	1.03117455
171	574	66.3	6.7	0.83157354	0.72782261	0.81408088	1.19966193
121	409	44.2	5.2	1.0673863	1.07154895	1.06321858	0.94097008
159	468	52.6	4.6	1.17035857	0.97616237	1.1790226	0.90444629
75	10515	1131.5	6.39	1.20113434	1.19846603	1.16006612	0.89477842
161	392	43.7	5.15	1.04956579	0.85820214	0.98631362	1.02863222
204	282	31.9	5.03	1.04472914	1.01209946	1.02266568	0.97335776
126	438	49.3	5.48	1.06579816	0.98765263	1.08433574	0.97560484
104	716	80.9	6.58	0.93910358	1.01831745	0.94514307	0.96631726
294	253	27.3	4.87	0.96352817	1.02973403	0.96146036	1.01409973
131	591	67.6	5.25	1.46153242	1.39083085	1.39251673	0.71770916
136	313	34.7	5.14	0.95922438	0.96228747	0.93367196	1.04570002
93	691	77.1	9.19	0.97143811	1.03612142	1.01582508	0.9931992
239	302	31.5	5.67	0.90889797	0.76792559	0.88202973	1.14336476
127	661	76.6	5.4	0.87405261	0.8797787	0.87122324	1.11482579
109	318	35.6	4.86	0.91074713	0.96691606	0.91339994	1.03273625
1069	169	19.2	5.2	1.19148151	1.09704252	1.22974816	0.89501539
76	800	88.9	4.81	0.8625176	0.79985496	0.87803196	1.08477204
533	166	18.5	5.87	1.15572649	0.96566627	1.06889634	0.97642286
132	347	37.6	5.21	1.00738309	0.81672679	0.95890741	1.08464627
986	296	32.9	5.54	1.06318042	1.01989557	1.05644571	1.02115824
779	249	27.3	4.58	0.99867178	0.79458602	1.0026968	1.10293552
312	502	54.5	5.01	1.12442176	1.04601772	1.08425071	0.94435356
149	259	28.8	9.36	1.10681261	0.99980527	1.14226616	0.98779421
158	328	35	4.88	0.94370818	0.91363138	0.95947905	1.08288843
151	845	96.2	5.34	0.8715464	1.29625203	1.03948484	0.9439617
154	322	34.8	5.92	1.05215665	0.92739827	1.03945682	1.00345908
490	166	18.5	5.59	1.44648146	1.0780153	1.34642909	0.78047837
93	419	49.6	5.68	1.03029151	0.97321538	1.03367658	1.00807202
160	494	57	5.52	1.01445059	1.08174856	1.02536035	0.97027562
117	395	45	5.01	0.97274345	0.88456397	0.96191211	1.03816109
192	235	26.2	4.61	1.22280162	1.09227035	0.79915369	0.87315912
103	333	36.3	5.35	0.97150613	0.82167328	0.94456954	1.08403447
253	179	20.3	9.32	0.95690868	0.84284916	1.02462091	1.02973587
212	396	42.6	5.33	0.99636864	0.8418752	0.98393807	1.06195287
134	579	63.7	7.36	1.0255228	1.02241912	1.06602687	1.00500213
198	435	48.5	5.52	0.97132843	0.95731462	0.96051004	1.0211739
145	317	34.7	5.29	1.18112096	1.30507845	1.18708378	0.85130518
93	429	46.7	5.25	1.06556439	1.0239506	1.06233357	0.96586062
117	553	60.6	5.54	1.08321903	0.93994941	1.08363572	0.98663453
140	259	28.9	9.25	0.78866367	1.99542558	1.03476316	0.72611306
198	320	35.6	9.11	1.17915148	1.17271412	1.11245288	0.86384578
102	420	47.6	5.39	1.09049993	0.8513438	1.03260481	1.04170299
214	283	30.4	5.73	1.02316995	1.04163502	1.06454546	0.99665897
69	466	52	7.12	1.09139433	1.02972766	1.04331318	0.96066374

87	691	77.2	9.22	0.82520604	1.31240135	0.86317861	1.0334896
99	489	55.9	5.86	1.05303596	1.06287889	1.02720384	0.99767815
115	353	39.3	5.33	0.9529647	0.85196708	0.92808645	1.10004845
160	286	30.8	5.58	0.93596731	0.81478449	0.87785598	1.12077579
154	388	43.2	5.1	1.06800675	0.846025	1.02138639	1.01915214
185	312	35	8.66	1.15059884	0.92623039	1.2401155	0.96162972
271	385	40.5	4.94	1.02199517	0.8462292	0.96251527	1.10011061
179	253	28.3	4.97	0.92244482	0.85034808	0.89797826	1.09139558
125	615	69.1	5.01	0.9829255	0.92583366	0.98562161	1.03769718
261	255	29.1	5.4	0.97021646	0.84428654	0.9202635	1.05845545
238	333	37.5	5.19	1.80246199	1.54286964	1.64993608	0.47068528
151	544	60	4.77	1.03772398	0.97939755	0.99544765	1.017064
107	424	45.7	9.23	0.99803963	0.89598271	0.99321691	1.02940515
128	289	31.8	5.03	0.93518987	0.94834322	0.97212692	0.98483941
140	313	34.3	5.26	1.91128769	1.43059076	1.61927762	0.46845962
193	402	44.1	5.48	1.01534393	0.78308385	0.98071624	1.04448376
108	324	36.6	5.41	0.97971592	0.88576478	0.93901573	1.07886008
265	296	33.3	9.5	1.43669312	2.02953717	1.64165464	0.46553071
306	132	14.9	4.98	0.86310383	0.63320071	0.79439314	1.1924245
726	165	18	4.82	1.25856361	0.73596777	1.0839422	1.03354931
79	308	34.4	5.64	0.96152297	1.0103623	0.96581272	1.01997955
156	286	30.8	5.74	0.95183891	0.93493775	0.92577539	0.92604004
144	317	34.7	5.29	1.11457719	0.86061956	1.05938959	1.03038439
325	236	25.6	4.83	0.95090794	0.95241248	0.91765212	1.08083876
815	163	18.4	4.6	1.02683973	1.99856964	1.08374598	0.73931994
118	276	29.9	5.01	1.00711918	1.12933485	1.04638361	0.95970936
95	579	63.6	7.87	0.9930098	0.98249129	1.16576259	0.94343406
254	210	22.4	4.72	0.92405171	1.08336836	0.94305036	1.03497332
81	1274	144.4	5.19	1.03778857	0.87117815	0.86695275	1.10528579
187	311	34.3	4.86	1.08369603	0.98773551	1.08489783	0.95780709
85	506	57	9.48	0.96948091	0.96816091	0.95019594	1.04410577
257	208	23.4	5.73	1.08775847	0.81484826	0.98996065	1.05466969
79	953	109.8	6.46	1.03442087	1.05211311	1.02041363	0.98054633
111	356	40.2	5.01	0.89375151	0.76689795	0.84854488	1.14573048
815	198	21.8	6	0.99910238	1.42157506	1.04241481	0.87863024
793	175	19.6	8.25	1.05683034	0.88082561	0.98020247	1.04142616
78	354	39.4	6.37	0.71538726	0.60946507	0.68141374	1.31168898
99	668	76	8.72	0.93591778	1.08876407	1.00919178	0.98739644
256	199	22.3	5.71	1.06116632	0.89113612	1.10777086	1.00359239
126	492	54.8	6.42	0.88320163	0.80481658	0.86582155	1.14941129
266	304	32.4	4.98	0.73056693	0.64344953	0.67838209	1.35179735
198	288	32.5	5.16	1.07746995	0.95683459	1.05670884	0.98126474
173	230	24.7	9.01	1.04804516	0.73438226	0.90190204	1.10460774
136	546	60.5	5.31	1.29957187	0.70008123	1.12404241	0.98744116
70	1146	132.8	5.24	0.98077627	1.17884396	1.01971297	0.9502915
198	302	31.6	5.67	0.90059771	1.19209669	1.05550902	0.97331918
106	365	40.6	4.72	1.10248549	1.15467284	1.11454769	0.90652714
151	431	49.6	5.86	1.137152	1.11674209	1.16574226	0.88663308
151	573	66.2	6.57	0.77823853	0.68782872	0.80476786	1.17941264
73	1050	121.7	6.35	1.09089186	1.014067	1.06131521	0.98033855

125	492	54.8	6.57	0.86175714	0.86519525	0.99469498	1.12630934
123	549	63.8	4.67	0.90515601	0.96772244	0.88961553	1.09460655
122	318	35.6	4.84	0.98320897	2.02357724	1.15033347	0.72017491
168	209	22.5	9.66	0.98246191	0.93047422	1.01021668	0.96413292
94	414	43.7	5.14	0.98603818	0.87209988	0.90184528	1.10103292
136	342	38.5	5.31	0.96214584	1.03283833	0.9513178	1.02324779
348	237	25.7	4.83	0.98192846	1.47857229	1.00453711	0.92279425
103	993	103.2	3.8	0.93535828	1.36468171	1.74846164	0.77319945
82	993	108.7	9.31	1.04359748	1.02075978	0.93592267	1.01406845
139	392	43.4	5.08	1.08158454	1.16238309	1.07259058	0.86956088
136	461	51.1	5.31	0.82087318	0.76270322	0.80724168	1.18504796
113	289	31.8	5.1	0.66428474	0.81866839	0.67998258	1.24072656
123	302	35	6.47	1.08049405	0.86658399	1.01987421	1.0197157
116	428	46.3	5.58	1.02628847	1.02461304	1.01767112	0.99898445
96	336	36	5.52	1.66340533	1.44334398	1.54434746	0.54543509
138	286	30.8	5.58	0.94158691	1.17445186	0.94897119	1.00066028
155	309	34.7	8.79	1.09522175	0.90044392	1.0909957	0.98428693
87	244	25.9	5.48	1.04972542	1.01612598	0.95966121	1.01874332
191	381	43	5.19	0.9654544	1.17156051	1.04002696	0.92979251
251	257	28.7	6.19	1.25707556	1.09400149	1.24663917	0.84667804
102	485	54.6	4.92	0.85560807	0.84046169	0.85838846	1.1238476
84	418	47.2	4.93	0.98946396	1.07382239	1.02109948	0.98743204
127	427	47.5	5.22	1.36434644	1.22908466	1.33742843	0.74871445
119	549	63.8	4.73	1.03246826	0.79801603	0.94588752	1.10323945
84	929	109.2	6.61	1.01559218	0.95222506	1.05230232	1.0196434
105	333	38.1	7.64	0.70274493	0.6250582	0.70857294	1.26077148
116	421	45	5.94	1.06166584	1.03281307	1.03446477	0.97637898
108	378	43.4	6.1	0.92327813	0.96620641	0.88130598	1.0852615
80	466	53.7	5.44	1.10633602	1.02895726	1.09893518	0.94763087
74	965	105.6	4.72	0.70882541	0.73041641	0.94725093	1.26649312
99	424	45.8	9.36	0.50081682	3.1650309	0.93668208	0.53468828
70	1009	117.4	5.17	0.88130156	0.94105331	0.90075879	1.07682876
757	189	21	5.05	0.85311881	0.85367701	0.86678921	1.15411481
170	451	49.3	4.77	1.08804738	0.77920313	0.97802672	1.10130675
170	323	37.5	4.94	1.09650706	1.07579452	1.05324906	0.95437035
269	296	33.3	9.5	1.27505956	1.50574003	1.34915011	0.71933054
174	314	35	4.75	0.96695315	0.87309819	0.93843562	1.05788353
91	276	31	4.92	0.90629532	0.95245855	0.91095022	1.07313481
120	311	33.6	5.38	1.03873331	0.79058918	0.95068902	1.0862438
64	466	52	6.9	1.06684466	0.89204277	0.98210029	1.04046475
719	373	40.3	5.99	1.24917627	1.16347467	1.15254544	0.90604833
91	586	64.5	5.44	0.62107318	0.65557332	0.62135901	1.31756801
74	929	109.1	6.77	1.01999778	1.33524636	1.24725048	0.801424
137	197	21.6	5.02	0.85891632	0.94473903	0.85553769	1.11421184
94	532	59.7	5.74	1.04254952	0.90201553	1.05473807	1.02007864
71	800	91	6.49	0.96918517	0.9803227	0.99794756	0.98778541
178	588	65.5	5.72	0.78848016	1.04781565	0.89960235	1.02756527
112	916	104.8	5.53	0.92617787	1.10188056	0.99067216	1.00090373
254	253	27.2	4.92	0.91906555	0.95246245	0.91164425	1.0684198
98	233	27.2	5.12	1.05611236	1.06925224	1.06695422	0.96548787

102	493	54.4	6.16	1.6335686	1.30059888	1.53539598	0.59963779
275	336	37.7	5.3	2.28131412	1.21278766	1.54043494	0.43896573
75	780	84.2	9.28	1.17634821	1.01293061	1.19648917	0.94312382
127	329	36	6.44	1.0586484	0.94372092	1.06847284	0.97244016
88	333	36.3	5.58	0.92343586	0.86310882	0.82057018	1.1185887
920	104	11.4	4.55	1.14559252	1.14096087	1.05533238	0.89400502
91	438	49.9	8.09	0.98891827	0.97580717	0.99108763	1.02848542
97	437	49.1	5.15	0.91950521	0.85240919	0.872068	1.10073388
102	376	42	6.67	0.91008337	0.88908449	0.90429727	1.02480966
63	1055	114.7	9.07	1.01608335	0.87902615	0.99012503	1.05680765
119	391	43.7	4.67	0.99953111	0.93751034	0.96918931	1.02240354
77	929	109.3	6.61	1.02006451	1.09778738	0.94605427	1.02067003
63	1168	134.1	5.22	1.00214037	0.93964681	0.99293411	1.01899023
101	534	59.4	8.18	0.92691004	0.78883597	0.99768955	1.05074166
139	277	30.1	10.77	0.80173209	0.47779977	0.80751089	1.27403814
95	492	54.3	6.02	1.18260669	2.58522367	1.383082	0.4445358
180	271	30.6	7.93	0.80726471	0.74426018	0.80515342	1.21041096
88	284	32.8	7.31	1.15617539	1.14315583	1.19491517	0.87488014
80	486	53	6.34	1.37311507	1.14295718	1.06949515	0.90275632
210	145	16.3	9.31	0.88891956	0.76207393	0.87289191	1.12271821
55	769	86.3	6.89	1.07499321	1.07744024	1.0325536	0.98354771
83	428	48.1	5.49	1.05654509	0.94405934	1.00697373	1.00581206
69	996	111.8	6.65	1.29366103	1.17374769	1.5496843	0.72139331
74	607	68.2	4.98	1.09875138	1.04395009	1.11824472	0.92490262
74	419	44.9	5.52	1.12163676	1.09067622	1.16134314	0.90993206
189	336	37.9	5.33	0.9474259	0.96550967	0.96810579	1.02511305
82	312	35.5	4.96	0.97482841	0.99845376	0.9799884	1.02960752
76	449	49.9	5.74	1.06698003	0.9698972	0.98777044	1.02806223
182	209	23	6.55	1.03520435	1.0851822	1.05454447	0.97466635
153	376	41.7	4.79	1.00145935	1.08097704	1.00628758	0.98940231
132	557	62.6	6.19	1.04284737	0.96110873	1.08747804	0.97291106
253	396	43.3	5.19	0.77128864	0.80961645	0.73512221	1.1993332
149	401	44.5	5.05	2.0011706	1.94446068	1.82077355	0.26014332
104	332	35.7	5.25	0.94613672	1.36277919	1.18100831	0.87816542
49	1009	114.8	6.58	0.92350012	0.98728623	0.91775942	1.0492071
93	413	46.2	5.57	0.9339124	0.91152209	0.98754941	1.04138346
56	1055	114.6	9.07	0.90359135	1.01229044	1.12847033	0.98325672
119	240	26.7	8.63	1.07574926	0.82418101	1.07473953	1.04099161
88	505	56.4	4.86	0.91461312	1.06557732	0.96707925	0.99392241
75	224	25.5	5.24	1.65778378	1.49907337	1.54766915	0.55511213
48	1009	114.7	6.64	1.1101091	1.10736601	1.13966295	0.91752525
59	604	69.3	4.83	1.14013093	0.89902831	1.06033477	0.9999025
134	412	45.1	6	1.03532848	0.82270973	0.98367691	1.07693774
71	642	74.4	5.41	1.03041434	1.03363434	1.00589374	0.99254509
664	122	12.7	4.65	0.91545894	0.95298565	0.9556022	1.02681288
57	672	77.8	5.29	1.0031894	0.9854062	1.06250203	0.99201296
74	416	48.9	7.71	0.81299451	0.76126801	0.7693044	1.17981347
76	328	35.1	5.38	1.05351319	1.07078569	1.01064533	0.97546972
64	423	47.9	9.04	1.15204581	1.1139434	1.12266996	0.92629669
178	611	66.2	4.73	0.86035005	0.86477623	1.08561169	1.01298944

112	310	33.6	5.4	1.72345073	1.67638944	1.53775504	0.50425045
106	346	38.8	5.38	1.03862501	1.16439912	1.0512735	0.95214521
113	220	23.2	4.86	1.07688827	1.13776127	1.07734917	0.92041025
667	71	7.8	4.54	1.23605362	1.57435898	1.51880634	0.61771304
132	149	16.9	4.59	0.72544469	0.84682965	0.83164655	1.12155672
69	655	72.6	8.94	0.95273801	0.97590385	0.88687219	1.05140368
82	430	47.2	5.17	0.98616654	0.97544259	0.96725745	1.02498519
61	873	92.9	4.16	1.02848977	1.19434638	1.51254379	0.80504038
62	435	48.3	5.78	1.00750315	0.98961041	0.92836102	1.03326609
45	450	48.6	5.53	1.05588765	1.1533114	1.03357605	0.97620501
77	421	48.5	8.62	0.97345084	0.96743007	0.98186077	1.0304039
50	440	48.3	6.01	1.07353056	0.88070593	0.97873275	1.04554752
63	285	31.9	8.38	0.9761916	1.01445586	0.97019421	1.00300838
136	215	24	4.93	0.90202263	0.96307988	0.92373433	1.04074192
58	760	86.5	6.93	1.00174345	1.01675626	1.00044412	1.03868746
119	535	59.8	5.1	1.08220164	0.90290276	1.01962914	0.9986467
89	198	21.6	4.75	1.00083784	0.79057068	0.92854388	1.08015424
87	324	36.6	5.41	0.99232559	0.9564974	0.96083239	1.09352859
53	1436	162.4	5.4	0.98136486	0.96882844	0.97123243	1.03156265
94	437	49.1	5.26	0.94892972	1.16822225	1.0798041	0.97611186
74	477	54.4	9.36	1.11519258	1.0313099	1.08790571	0.95546462
111	276	30.2	6.47	0.97910045	0.97946502	0.99435539	1.04046515
161	164	18	4.75	0.87627259	0.97166532	0.83946263	1.11399441
63	372	42.1	5.5	0.98035547	0.88887067	0.98263402	1.05918417
297	217	24.1	9.76	0.94752831	0.84255333	0.96624406	1.064441
86	328	36.7	5.64	0.88454442	0.80942843	0.84444537	1.15703555
83	420	46.3	4.63	1.07840315	0.81965674	0.98599346	1.05476148
88	689	76.8	9.98	1.11773054	0.78967574	1.32422814	0.89915774
69	541	59	4.79	1.04423141	1.69589924	0.85426813	0.94228074
73	421	47	5.85	0.86915981	1.03716556	0.92851349	1.05286632
103	425	46.5	5.2	1.56591082	1.75504862	1.50563865	0.52390765
94	398	46.2	5	1.01314275	0.96038382	0.97558454	1.03336904
71	451	50	5.48	0.96670284	0.88094994	0.9581226	1.06714581
105	260	29.1	9.33	1.13049157	1.42639826	1.37818214	0.73882692
75	365	41.3	6.92	1.21466801	1.31701569	1.20610205	0.82658755
369	468	49.5	5.03	1.01501082	0.51566168	0.84150052	1.2472466
323	114	13.3	4.37	0.89620705	1.10348098	0.93175049	1.03552464
73	886	97.5	4.75	0.9250029	1.13641172	1.58279988	0.83397219
57	602	68.4	6.49	1.08429232	0.910939	1.02559342	1.01821491
61	415	44.6	5.24	0.94213971	0.89708305	0.94668756	1.07644597
119	220	23.5	4.74	1.0911386	1.02189363	1.0179966	0.98729373
172	190	21.3	5.47	1.44766982	1.44234356	1.51411273	0.67068727
67	642	74.3	5.36	1.01678981	1.04711348	1.00702347	0.96766311
102	335	36.2	5.99	1.04454374	1.02630216	1.05648479	0.95803473
85	505	56.4	4.86	0.90145108	0.98914242	0.91987554	1.01817405
126	200	23	10.02	0.94285319	0.68795493	0.91340836	1.14100902
75	223	25.3	5.83	1.01238889	0.76696212	0.98563251	1.09331724
61	681	76.7	8.06	0.65289488	0.69125172	0.65179428	1.26738574
76	284	32.9	6.81	1.22169627	1.1226887	1.11804103	0.90899998
125	454	49.8	9.35	0.95541539	1.29470052	1.16090323	0.80245329

55	239	27.6	7.11	1.62908991	1.48996933	1.20605235	0.65958051
52	171	18.7	4.83	0.97675286	1.0833413	1.02798776	1.0160762
77	625	70.1	5.54	0.99852011	1.02893604	0.98065425	1.02621383
88	136	15.1	7.42	1.0676203	0.98331221	0.78763662	1.065874
81	241	28.1	5.35	1.10247437	0.9758515	1.05873299	0.99927061
90	228	26.7	5.3	1.112435	0.88066059	1.03696733	1.04376946
119	149	16.9	4.59	0.68476336	0.57753344	0.70911004	1.30612124
61	379	41.7	6	0.91285282	0.84436797	0.89777639	1.08278237
115	238	26.6	5.68	1.01402206	0.97832613	0.96725944	1.06397621
230	166	18	4.65	1.11677792	0.92595358	0.99348872	0.99960044
87	360	40.7	4.94	1.17580951	0.93444903	1.12581946	0.93528738
47	647	75.1	5.76	1.0487117	0.88868598	0.95378355	1.07731025
58	285	32.3	5.41	1.03928221	0.94305267	0.98914096	1.02957009
58	311	34.2	5.58	1.05484742	0.9187956	1.03805067	1.01725625
85	233	25.7	4.89	1.16392428	1.01852327	1.03740023	1.0410709
80	281	31.7	6.01	1.00260856	0.93854447	1.00776147	1.011178
53	377	42.6	6.1	1.00590666	1.01769387	1.00590425	1.00613464
57	500	57.5	7.8	1.00650529	0.93604874	0.99822432	1.07480257
75	233	27.1	5.19	1.03213229	0.93480614	1.1112162	0.96976541
89	368	42.1	5.11	0.86296831	1.18806923	0.93711365	1.00262493
63	310	35.3	6.35	0.96064568	0.92440985	0.92027955	1.07841681
69	497	58.2	5.38	0.94618921	0.91048743	0.98710897	1.17521301
49	1436	162.4	5.36	1.17201076	1.57091657	0.94095407	0.83560339
184	587	65.3	5.92	0.94505842	1.40722023	1.02365642	0.90837547
51	284	32.5	9.36	0.97545844	0.91668094	0.95127002	1.07703839
57	665	74.5	6.92	0.9972796	1.00366936	1.01910854	1.00284416
77	321	35.3	6.25	0.98024679	0.8418259	0.93298238	1.09189549
63	642	74.4	5.38	1.42028579	1.35217627	1.43305473	0.6439819
49	496	54.6	5.07	0.83795005	0.82152373	0.89787699	1.11975879
70	356	39.7	8.94	1.12395965	1.11245383	1.17798144	0.8933556
53	744	82.7	9.29	1.07762516	1.03823548	1.07605507	0.95251805
87	276	30.2	6.34	1.16642001	0.98149706	0.94037995	1.00320216
104	450	49.2	9.31	1.12891713	0.61098505	0.89503497	1.22857353
114	901	98.9	4.91	1.02660484	1.05259977	0.81064604	1.55632704
87	564	65.9	6.51	1.01989146	0.86181158	1.02693177	1.05283084
86	179	20.1	5.19	1.0155019	1.01571919	1.04259855	0.98052601
106	238	26.6	5.5	1.01419021	0.82980102	0.9707886	1.07446029
64	519	58.5	5.27	0.98695269	1.046308	1.02387874	0.99181848
115	187	21.9	5.34	1.02948996	0.84914513	0.94066258	1.08139804
81	467	52.3	5.36	1.14065524	1.08932523	1.14193317	0.92115304
73	681	73.9	6.37	1.04705795	0.84468729	1.04081968	1.02995611
65	288	32.4	5.5	1.06800353	0.84911617	0.98057822	1.0507082
65	321	36.3	9.7	1.67033643	1.02535528	1.59254179	0.62122006
68	436	49	5.36	1.08963576	1.00395641	1.0379861	0.96153226
49	358	39.8	5.49	1.06313669	1.05354972	1.08348177	0.96916316
141	645	74.6	5.34	1.02995147	1.68443752	1.15892198	0.71373473
109	293	33.2	5.9	1.70109608	1.38979066	1.42517456	0.59601884
59	424	46.2	6.3	1.21181767	1.13813549	1.32625764	0.79807568
45	300	34.2	9.45	0.92039938	0.78730314	0.95027384	1.13293717
54	231	24.6	5.64	1.15029218	1.09216174	1.11874433	0.92053861

150	164	18	4.68	0.59679159	3.00630793	0.90259513	0.65828905
41	627	72.6	5.53	1.08475835	1.29885872	1.11050744	0.86371566
60	664	74.3	6.07	1.10475447	0.80450392	0.93556082	1.09209451
49	393	41.7	5.38	1.00666628	1.01573165	1.01206045	0.99624832
78	415	45.8	5.15	1.45580584	1.4201924	1.44444469	0.65581669
90	317	34.5	5.07	1.64130269	1.40903382	1.60369337	0.55276502
96	492	54.9	5.29	0.91422637	0.87159694	0.86158952	1.14636271
65	372	40.2	5.24	1.48728996	1.5509621	1.3742077	0.63895316
177	696	76.6	4.93	0.71503109	1.84509641	1.05871212	0.78477171
95	260	29.1	9.41	1.3453752	1.07622738	1.35827645	0.79221758
66	258	27.2	5.16	1.18090189	1.33013185	1.2810702	0.77155802
188	195	21.5	5.26	0.99421712	0.83816496	0.91834843	1.10663818
52	502	56.6	5.43	0.97383509	1.12230679	1.02795247	0.98254047
65	416	48.9	7.3	0.5033066	0.33448504	0.54543509	1.45215052
52	241	27.5	8.46	1.0726597	0.86563213	1.01401216	1.02120115
63	437	49.2	6.81	1.12746934	0.99530179	1.05122004	0.96907364
73	227	25.4	8.84	0.91924445	1.06128098	0.95652126	1.04621422
413	163	18	7.5	1.00976166	0.68552728	1.02133983	1.10516041
103	147	16.7	4.64	0.97597595	0.79470196	0.84970629	1.20651929
48	711	81.5	9.51	1.05232387	1.15890391	1.06003509	0.94820156
727	66	7.3	4.56	1.4059846	1.52380064	0.91864787	0.79936114
59	768	83.2	9.33	1.152979	0.84067357	1.11025262	0.9861268
70	452	49.9	5.15	0.81682136	0.94016105	0.87001363	1.06353224
52	273	30.3	9.04	1.25009113	1.08047279	1.44459373	0.78187813
64	368	40.8	5.14	1.07645678	1.02937517	1.02536149	1.00431078
62	311	34.5	5.63	0.97376275	0.97034395	1.00223626	1.04282667
43	565	65.8	8.75	0.94170881	1.01226939	0.94647753	1.04554178
99	354	38.4	5.31	0.90307845	0.78756268	0.90177211	1.12703224
75	208	24.5	5.43	1.06596725	0.87296327	1.00878946	1.04334101
58	575	64	6.83	0.89777394	0.86412482	0.90236957	1.10054859
134	286	32.2	8.98	0.78213288	0.64738465	0.73003582	1.29644824
51	393	43.2	5.71	1.17954507	0.95675917	1.03782617	0.98022392
72	341	36.8	9.55	0.98160066	0.7290351	0.99345556	1.13678048
88	323	36.9	4.88	0.98357089	0.8992775	0.92150981	1.08977007
292	233	24.2	6.62	0.99327177	0.57442271	0.37054573	1.41285458
237	502	54.6	4.93	0.81073179	0.62320582	0.76660631	1.19283335
75	182	20.7	5.11	1.04609827	0.97156602	1.01788974	1.00608566
49	689	79.1	9.03	1.01486164	0.93901468	0.99791883	1.02328164
423	166	19.6	4.72	1.35970817	0.88075062	1.26545383	0.8997445
79	272	29.9	5.05	0.97888857	1.4824135	1.24109813	0.68333167
634	174	19.1	5.58	1.07804038	1.39100846	1.16231411	0.83222195
229	135	15	4.89	0.9505512	1.06096037	1.12072069	0.964311
214	140	15.7	8.9	1.19781055	1.1616001	1.03556178	0.94154586
43	426	47.5	8.47	0.99734602	0.98434457	0.99361464	1.02141002
89	455	50.7	9.31	0.93372337	1.07425122	0.92262276	1.03788789
61	358	39.2	5.15	1.25534726	0.85049413	1.15100992	0.99720734
115	322	35	5.01	0.87404871	0.76758168	0.80654861	1.16586498
83	358	40.3	4.91	0.96597271	0.92373162	0.89654236	1.0768132
48	532	60	8.7	1.05736502	0.91435159	1.01502592	1.01549645
55	346	37.3	6.7	1.33757988	1.19633502	1.33745061	0.76789178

71	433	50.6	7.66	1.06021896	0.77906686	0.9388609	1.10521971
48	302	34	9.14	0.96445974	0.93121224	1.00937071	1.01207023
48	897	104.2	7.02	1.02878183	0.88673832	0.971386	1.04782362
76	310	34.4	6.13	1.00701777	1.01342137	0.99902299	1.02163654
55	399	45.4	6.38	0.80995968	0.86372063	0.79603181	1.1526889
81	228	26.7	5.3	1.06799188	1.27643798	1.21518825	0.81774569
59	381	43.3	5.15	1.00078835	0.97399243	1.02826998	0.99648581
49	274	30.4	8.59	1.00445823	0.89232179	0.98892323	1.03197611
46	726	78.3	5.47	1.10022818	1.05576095	1.1790426	0.91261269
63	332	36.1	6.33	1.06933935	1.00739398	0.98660136	1.00877142
198	555	59.6	5.54	1.01089857	0.91579264	0.9189211	1.07100908
54	211	23.2	5.39	0.93441696	0.85058752	0.95418997	1.08190981
106	168	18.7	7.24	0.90741621	1.17261796	0.92664271	0.99663325
57	333	38.4	5.71	1.04007093	0.93675197	1.01182768	1.03157745
63	188	20.9	6.14	0.57946119	0.55732727	0.5895269	1.34249441
70	144	15.5	4.51	0.99985548	0.98289833	1.04144208	0.99010556
90	407	46.4	5.45	1.06816714	1.0300452	1.04903288	0.96523109
49	559	64.1	5.1	1.0849616	1.16800747	1.09321572	0.91547496
96	513	58.2	4.86	1.07820152	1.25636514	1.13169392	0.94515489
101	240	26.2	6.1	0.92980171	0.81085149	0.91909395	1.10650992
274	90	9.6	9.52	0.96095023	0.93280538	1.04439252	1.0317467
45	494	54.1	5.54	0.9676778	1.05468076	0.98674553	1.0134187
50	575	64	7.03	0.83065053	0.77923061	0.83805593	1.14366847
79	341	37	6.4	1.30428876	1.29090653	1.32868974	0.74781068
46	519	59.5	5.78	1.22539945	1.13879081	1.15605427	0.87055061
47	459	51.7	7.11	1.01466033	0.99403442	1.06186681	0.97609925
57	272	29.3	6.16	1.14434937	0.96866081	1.04988722	0.96901278
65	420	48.3	5.38	1.0822877	1.24970631	1.15320193	0.87722691
47	753	84.6	5.92	0.88971293	0.89065991	1.01330842	1.05879855
47	753	84.6	6.05	0.89177391	0.85449208	0.94852186	1.07324855
56	315	34.9	6.98	0.98883048	1.07066272	1.00924504	0.98200976
66	341	38.4	5.12	1.02298747	0.88038592	1.01078235	1.03827764
50	273	30.3	8.94	1.3131144	0.89399759	1.29557822	0.85490958
92	514	58.2	4.87	1.07003592	0.89591287	1.10831259	0.98040273
68	307	35	5.05	1.06171807	0.92560631	0.99461078	1.03185713
166	184	20.3	5.15	1.34978531	0.59645724	0.94183577	1.12099528
58	518	59.4	4.82	1.1006971	0.8793656	1.0326598	0.98438967
103	177	19.1	4.81	0.891327	1.06262736	0.94308844	0.99224428
43	383	43.1	5.91	0.91945669	0.77866299	0.86187194	1.15506094
70	305	34.8	8.32	0.90687639	0.84702484	0.88452478	1.12645814
45	700	80.7	8.07	1.05942947	0.96764942	1.02336414	1.02206609
63	168	18.6	9.16	1.19967189	1.27933819	1.07553804	0.86771712
60	759	84.2	9.64	0.94414996	0.8602485	0.91290679	1.0980804
141	611	65.9	4.68	0.93507519	0.97429802	0.95181094	0.99705055
70	229	25.3	5.25	0.80102343	0.8845657	0.81809382	1.15141689
41	375	42.1	5.41	0.82680568	0.86786973	0.86269422	1.15584935
61	503	57	9.47	1.03179033	0.6882513	1.00309136	1.14397642
46	322	36.9	8.87	0.93244282	0.80893946	0.97679064	1.07651396
65	273	30.6	5.26	0.95589534	1.04408168	1.01818482	0.94603642
38	546	62.6	7.68	1.13314318	1.19538975	0.97291116	0.9499412

125	76	8.8	4.37	1.33368529	1.54498979	1.09458581	0.78961945
89	336	38.3	5.01	1.1382156	1.06833077	1.19062848	0.9132071
72	223	24.4	4.92	0.96874039	1.57800892	1.19610995	0.77585953
96	147	17.1	7.43	1.04938145	1.09042319	0.99941149	0.98161658
55	491	54.1	6.07	1.03095702	0.92251125	1.0043246	1.02041931
87	272	30.3	5.52	1.13805691	1.04024758	1.1023768	0.95864023
78	652	68.8	6.28	0.93403008	0.80064952	0.9462292	1.07581859
157	184	20.4	5.26	1.10556354	0.97149749	0.96479931	1.00132961
61	701	80.1	5.49	0.99918052	0.87249004	0.99134694	1.06528244
45	294	32.3	7.27	1.16903977	1.05263812	1.03498757	0.9492372
57	348	38.1	6.74	1.08594171	1.1002756	1.09210258	0.9357902
43	346	38.5	6.58	1.09126824	0.92223786	1.05207123	1.07251416
53	223	24.5	5.15	1.35640285	1.32532818	1.41988299	0.71542706
46	327	36.1	8.24	0.75620993	0.74548525	0.79904013	1.29617728
78	290	33	7.59	1.06445349	1.1292973	1.01368517	0.98651817
37	440	48.4	5.72	1.01218192	0.69529952	0.90085092	1.18219445
113	267	29.3	8.94	0.84120038	1.04609325	1.08883387	0.96859672
39	457	53	6.14	0.97937719	0.74459418	0.89143118	1.14021378
63	313	33.9	5.06	0.97024853	0.85767521	0.86833797	1.1049552
53	363	40.6	6.37	1.18070078	0.9968771	1.04335682	0.95600389
324	292	32.4	5.19	0.95429961	1.01982301	0.98238278	0.9697943
44	267	29.5	5.22	1.12330691	0.99671018	1.04546575	0.96914069
104	443	49.6	5.07	0.97763525	1.07157268	0.95573403	0.91764222
56	520	59.5	5.02	0.99750939	0.95719792	0.98611648	1.03943018
55	208	24	4.56	1.15673583	0.96177572	1.02952112	0.96958284
42	381	42.2	5.34	1.01822205	1.2030697	0.96898418	0.97190967
54	437	49.1	6.48	0.72450749	1.48903003	0.97665856	0.9015746
74	729	83.7	8.31	1.035198	1.30211941	1.17758578	0.87458982
76	453	51.9	5.43	1.03321463	0.9653499	1.03536851	0.99380959
68	290	32.9	7.2	0.95978135	0.86219855	0.95065801	1.08913092
43	616	70.5	6.62	1.08639543	1.40415693	1.07856874	0.86679094
93	236	25.9	4.96	1.07459267	0.97498561	1.00430878	1.00029629
60	412	45	5.38	1.05443892	1.01925367	1.09631271	0.95004643
51	307	33	5.71	1.53773129	1.34252379	1.44999559	0.66154558
37	565	63.4	5.63	0.99717787	0.95510504	0.9760816	1.04871105
127	158	17.7	4.59	1.01437561	1.10464451	0.96400527	0.98352284
80	140	15.9	5.03	1.075028	0.70909665	0.95743378	1.06374408
54	226	23.8	4.68	1.00604366	0.90194155	0.98221814	1.05563161
53	288	31.3	5.63	0.62054272	0.80083224	0.65464991	1.3057074
60	601	66	5.11	1.06127942	1.57223477	0.97704785	0.87770787
48	416	46.6	4.77	1.03240169	0.91393293	0.95436185	1.06584601
96	108	12.3	10.05	0.81961691	0.67901558	0.78828477	1.20726912
46	376	43.3	6.87	0.88293825	0.83580247	0.88118465	1.14194681
109	185	20.6	4.82	1.01394887	0.910062	0.91803018	1.04440547
54	302	32.7	4.89	0.86154464	0.99377111	0.72058352	1.05844094
90	902	99.1	4.98	0.89852256	1.10793877	1.0008922	1.09007106
63	366	40.4	6.24	1.52787492	1.38419465	1.39045224	0.66732839
229	237	25.7	4.81	0.8954875	1.32084047	0.96811154	0.9470275
80	243	28.6	4.93	0.98718923	0.85557391	0.9367972	1.10562646
41	544	60.6	4.88	1.06671411	1.36230623	1.1340737	0.87099562

39	665	78.9	5.69	1.00760182	1.06861477	1.04118015	0.97230742
113	197	21.6	4.68	0.99862034	0.98466111	0.97663251	1.02770102
43	780	88.3	6.1	0.94171491	0.86492297	0.91173078	1.0976128
54	208	24	4.51	0.77116147	0.30044528	0.60183558	1.47355194
54	154	16.4	5.99	1.25205859	1.44586301	1.12146978	0.80535227
44	285	32.3	5.52	1.05666189	0.75206931	1.01015081	1.05207831
45	292	32.1	4.98	1.42839744	1.38168823	1.46751499	0.67876031
33	872	99.9	5.02	1.02995447	1.0670979	1.00329679	0.97466678
47	391	44.9	9.22	0.89148217	0.67700213	0.79362857	1.19920229
53	219	24.6	5.22	0.92032618	1.03305968	0.95942892	1.0165293
37	288	32.1	4.96	1.06073641	1.00161233	1.01114042	1.00349363
48	691	76.5	8.92	1.07536066	0.89613109	1.02455738	1.02291824
77	199	23	5.55	0.79404499	0.90794499	0.82340681	1.15084421
145	178	19.8	9.54	0.96143082	0.78278873	0.88521376	1.09501012
160	166	17.7	9.88	0.94202411	0.79422007	0.97713632	1.06929059
133	392	43.2	4.61	1.0092935	0.93616587	1.02435491	1.01296678
38	171	18.7	4.77	1.15137602	1.22415661	1.21797221	0.8399175
244	217	23.8	4.42	0.83382682	0.69950593	0.80293245	1.13860082
216	169	19.3	4.93	0.97944406	0.72191905	0.90548745	1.15475547
73	413	48.8	9.35	0.84514071	0.85171275	0.85797562	1.15389515
44	266	29.7	7.55	1.00162084	0.84373201	0.95191734	1.08047853
37	302	34	9.03	0.99700923	1.47059129	1.04803403	0.86210371
58	626	72.2	4.65	0.72725711	0.72345346	0.74269593	1.21007808
70	260	29	8.05	1.06054534	0.9358383	1.05719144	0.99627741
50	397	43.6	5.01	1.06684158	0.89493439	1.00450171	1.01823616
56	408	45.8	4.91	1.09453233	0.93334981	1.02360873	1.01317043
92	252	27.5	5.19	1.04844997	0.99897192	1.01717268	0.9899255
43	182	20.4	9.72	0.76153399	0.97273892	0.96580324	1.017721
72	108	12.6	4.63	1.11535402	0.74542832	1.00735217	1.06449641
118	100	11.3	4.81	0.97611842	1.15416432	1.00453286	0.9880962
67	175	19.8	5.47	1.29906224	1.32252426	1.25438698	0.78483105
50	363	40.6	5.99	1.1267995	1.19632978	1.19215115	0.86803946
64	296	33.1	6.09	0.96405753	1.04331563	0.99887259	1.00218847
60	234	26.7	5.77	1.83793336	1.25579063	1.61671713	0.52041199
43	266	29.7	7.55	1.04717474	0.68373307	0.85180856	1.27350219
44	284	33.5	5.11	1.21287709	0.96021551	1.2594978	0.89235782
51	379	43.3	6.37	0.98289131	0.92011751	0.99283391	1.04397239
68	298	34	5.2	1.04221679	1.08165605	1.12646489	0.94702376
49	281	31.4	9.7	0.8582423	0.72037307	0.87104959	1.1613105
226	504	54.9	5.14	1.08320746	1.0948507	1.08636486	0.94577578
78	279	30.6	6.04	1.04532803	0.90980539	0.98274129	1.02991566
41	352	39.6	5.34	1.16208387	0.94168803	1.18652902	0.92683938
42	374	42.4	6.11	1.64714847	1.53853974	1.65950258	0.49759682
40	327	36	4.86	0.96886954	0.96618165	0.97920769	1.00203107
181	195	20.8	4.7	0.8481424	1.58152034	1.1840584	0.8155567
45	292	32.1	4.89	1.21825293	1.47887646	1.34626882	0.72959563
86	205	23.7	4.72	0.99768743	1.07792812	1.03629549	0.97561243
60	308	33.6	4.92	1.02186501	0.76011789	0.90503386	1.123798
41	669	76.8	5.39	0.84889802	2.77938471	1.02010078	0.63052371
48	421	48.5	6.23	1.10786707	0.98140166	1.04631056	0.98238531

261	48	5.7	5.15	1.5281485	1.97456485	1.29331991	0.61798771
132	295	31.8	5.21	0.93584523	0.61658421	0.81978971	1.2830869
34	384	43.2	6.25	0.95998792	0.8789921	0.89372019	1.09197796
52	348	38.1	6.55	0.93492962	1.71982044	1.15562602	0.77849399
35	459	52	5.81	1.59581837	1.48916646	1.42945309	0.61851762
79	183	20.5	6.27	0.94830208	1.16501921	1.02625684	0.98067864
77	168	20.2	9.22	1.07270581	0.81685	0.99169362	1.00201114
90	129	13.9	11.21	0.93070469	0.82400312	0.95928179	1.11130296
46	323	36.7	6	0.92261361	0.87145857	0.90964031	1.09620642
49	424	46.8	5.5	1.06584442	0.9427732	1.00871985	0.98304394
44	319	35.3	5.21	1.07930086	0.90943498	0.95761044	1.03951778
51	328	37.9	5.26	1.15149449	0.80559464	1.01079386	1.03928308
36	295	33.2	8.78	1.11599549	0.98580593	1.09510075	0.93386963
44	272	30.8	5.21	0.94367557	0.86644297	0.90970491	1.08852038
41	669	76.9	5.33	0.96288022	1.14485004	1.00176263	0.99717038
42	438	48.3	5.55	1.16247954	1.1208621	1.21625333	0.85126348
50	204	24.5	7.59	1.08211029	1.02557737	0.9837074	0.98784734
41	358	41.7	7.17	1.15299113	1.10147697	1.13629356	0.91044437
31	445	50.9	9.01	1.03114952	1.10597886	1.03032459	0.96118165
46	318	36.8	5.64	0.91802679	0.94355954	0.93577838	1.03839658
35	551	61.5	9.17	1.12457466	1.00525349	1.24122478	0.85935023
39	306	32.9	7.15	0.98389406	1.01079695	0.99824531	1.00424959
67	302	33.5	5.44	1.01806885	0.96206762	0.99552468	1.02189393
38	322	36.8	8.5	0.94188498	0.82852651	1.03523759	1.06837712
77	207	22.5	9.91	0.96811497	0.88609965	0.87197937	1.07673018
64	286	33.1	4.83	0.85025407	0.88540487	0.84902254	1.13092709
34	182	19.6	5.69	0.93195465	1.09583308	1.02703991	1.01948679
52	293	31.8	4.67	0.95720554	0.89546699	0.93592638	1.06065099
45	307	33.7	5.69	0.97720601	0.92017521	0.98448049	1.04614695
135	68	7.2	4.13	1.47301328	1.37746023	0.84355999	0.86656735
42	448	52.9	6.99	1.04696659	1.06285012	1.05214992	0.94267761
170	388	42.2	4.88	0.83623894	1.49096382	1.04735847	0.89516386
35	490	57.2	7.62	0.81850389	0.73725347	0.80217025	1.2053149
41	305	34.6	9.06	0.92950953	1.05629306	0.98267358	1.03916636
37	345	39.3	6.35	1.11796526	0.84413743	1.0464626	1.01520721
98	176	20.9	3.76	0.92831275	0.87462105	0.86951175	1.05967713
40	245	28	9.32	0.98667537	0.94913002	0.99956014	1.02565462
43	299	33.7	6.39	0.91785938	0.90558488	0.95258821	1.08010041
47	390	44.8	6.64	0.95063972	0.84972492	0.94568503	1.08735712
37	466	53.3	5.45	1.04613241	1.00646907	1.07918618	0.94947438
41	335	38.6	5.22	0.86940328	0.94354884	0.86800868	1.12108032
48	202	23	4.54	0.88999085	1.03010823	0.92862517	1.03520672
64	173	19.5	5.11	1.15291584	1.07298246	1.06088727	0.94281052
60	494	54.4	6.58	1.38723444	1.47695486	1.3827357	0.67049134
30	323	37.1	5.92	0.93455985	1.04649049	0.92945161	1.03019172
44	790	90.4	6.8	0.93453217	1.12930422	0.89823417	0.82358907
34	280	31.6	5.58	1.01381116	0.82612387	0.95508197	1.06456257
121	137	15.2	9.74	0.97862197	0.8194251	0.89535674	1.1007236
50	167	19.6	6.64	1.05874867	0.83294722	1.05299473	1.02785835
61	487	53.2	6.47	0.9261641	0.83519037	1.12878804	1.08028448

257	325	35.2	4.65	0.92265385	0.86940393	0.96813781	1.07165513
54	269	30	4.46	0.91139432	0.8517018	0.88204779	1.09759543
83	88	9.3	5.39	0.99332394	1.3867029	1.26286423	0.90205931
40	1217	141.1	5.27	0.94799107	1.13198975	0.99700671	0.99113398
37	230	25.6	6.44	1.6799009	1.06700758	1.39733375	0.70926224
69	299	34.3	6.71	1.11187042	0.99671771	1.0833526	0.95350993
127	117	12.8	9.92	0.84020781	0.77113858	0.89680118	1.12198785
31	179	20.6	5.99	0.76839913	0.83583863	0.82728218	1.13259471
65	328	36.7	7.42	1.00299656	1.02557131	0.97604729	1.01545432
57	1024	106.2	3.8	0.90677199	1.34639399	1.4840646	0.89570323
76	659	75.1	5.36	0.89269801	1.37129816	1.04411602	0.92494318
68	585	69.4	9.54	1.24745553	1.13868709	1.35163637	0.80395238
78	99	11.3	5.08	0.99098063	0.93430441	1.03712237	1.02051575
137	465	52.7	4.97	1.02751053	0.86803309	0.99945734	1.04056161
148	124	14.7	8.06	1.04175405	0.87375736	1.0576173	0.97727369
143	98	11.6	5.19	0.91170498	0.91746351	0.94160048	1.05884606
34	305	33.6	4.84	1.11562421	1.18745579	1.08231889	0.9173756
49	374	41.8	9.31	0.96861463	0.84298593	0.93955049	1.09152746
38	514	58.9	6.23	0.95467615	0.96195049	0.9332219	1.04365806
52	168	18.6	9.26	0.90047852	0.84062564	0.94354152	1.0811942
47	354	39.6	8.28	1.66577725	1.25847763	1.48808725	0.60010204
39	343	37.8	5.54	0.91295508	0.80353342	0.83637611	1.16942202
93	256	28	5.9	0.91912022	0.76510355	0.89517726	1.13583287
45	339	38.7	8.78	1.46713386	1.1656705	1.22166178	0.76850375
56	273	31.2	4.88	0.99085892	1.27969249	1.04752185	0.89680723
36	374	42.5	5.83	1.01529997	1.26498225	1.042909	0.93547577
55	266	31.3	9.98	0.88022794	0.79023582	0.91074491	1.10022101
42	384	42.8	5.99	1.17481757	1.02728721	1.09335421	0.97339805
121	507	54.7	4.75	1.04723813	1.32357225	1.14948731	0.85982407
38	491	54.1	5.87	0.9323179	1.39150824	1.0724029	0.96577185
36	608	69.9	5.72	1.12832116	1.01404866	0.99636561	0.97357579
29	1158	134.4	5.39	1.0034152	1.09618031	1.0212037	0.97745784
36	389	46	7.15	0.9220668	0.92713308	0.9348905	1.04524429
41	202	22.7	8.13	0.88188067	0.94165139	0.87540345	1.09335402
281	175	19.5	9.58	1.22137849	0.81955021	0.93880374	1.03651174
51	428	49.9	4.67	1.02041702	1.08205917	0.96415344	0.998323
40	410	47.7	9.57	0.85728879	0.80724981	0.89279254	1.10880524
46	209	23.4	6.58	1.12020974	1.00950596	1.16254887	0.92538266
55	426	46.3	5.85	1.00484589	1.11595776	1.01881781	0.98881277
44	303	34.8	5.91	0.92284015	1.10251082	0.96670467	1.00392778
52	267	29.9	4.68	1.01045279	1.11583822	1.07635108	0.94204963
50	154	16.3	6.55	1.20251635	0.96366453	1.15717153	0.92154903
39	453	52.2	5.24	1.18695725	0.93098143	1.12838324	0.9426197
46	342	38.3	5.49	1.26897544	1.19712825	1.26555504	0.81509674
119	83	9.4	4.59	0.96487113	1.13193523	0.94367821	1.00872426
50	562	66.1	6.77	0.97588937	0.78310005	0.92420687	1.11759548
511	92	9.8	5.14	1.14193716	0.76440766	0.97058879	0.95330934
35	265	29.9	6.62	0.93779829	0.95477907	0.99098185	1.04224721
52	302	33.3	5.33	0.95367741	1.00690415	0.9742782	1.03937354
33	341	36.9	5.16	0.99152245	0.96407606	1.01084532	1.02402471

26	313	35.8	8.65	1.29643319	1.0681766	1.1987866	0.84760939
37	351	40.5	9.36	1.6687959	1.11137138	1.38586674	0.67415707
33	344	39	5.03	1.1138897	1.08066049	1.07049972	0.91986339
57	277	31.4	5.21	1.11240356	1.00040475	1.03905321	0.9715007
70	108	12.6	4.67	0.95658715	0.7962554	0.92973056	1.06497408
50	408	45.8	4.94	0.94479489	1.02593668	0.94940941	1.05309274
82	146	15.6	10.29	0.87104978	0.76556503	0.78363582	1.14950276
140	166	17.7	4.83	1.02578266	0.69274042	0.86376411	1.12884952
86	180	20.1	9.8	1.11317498	0.87407227	1.17406438	0.98290538
56	289	31.5	8.98	1.32272455	1.33419299	1.36823149	0.7379583
180	118	12.8	5.85	1.04489957	1.21974961	0.97585707	0.91151747
48	219	25.8	8.13	1.13638882	1.05635734	0.99889396	0.97499059
33	399	44.1	6.01	0.89140374	0.92781354	0.88064508	1.10402612
111	105	11.6	5.1	0.99801881	1.16053181	0.95055622	0.99340305
46	419	45.1	5.85	0.92822483	0.82995266	0.88324171	1.1014311
31	431	50.6	5.94	1.0139711	1.10394729	1.06465983	0.95771528
85	662	75.2	7.65	0.99544143	1.25202552	1.05737697	0.95964586
51	283	31.2	4.92	0.81504907	0.78939579	0.78396445	1.16452735
33	238	27.2	7.99	1.13014065	1.04619248	1.13871463	0.90910337
39	283	31.4	5.4	0.74347344	0.68567084	0.83231886	1.17162269
46	154	17	4.82	0.92385034	0.91227388	0.90526265	1.08425405
81	279	32.4	5.34	1.04702906	1.1874876	1.07673613	0.92589037
51	123	13.9	4.41	0.82348931	0.6900518	0.82974966	1.23825369
32	281	31.8	6.21	1.00952736	0.97654977	0.99982679	1.01084194
40	557	63.1	6.49	1.0479746	0.95323249	0.9935997	0.99901012
113	414	45.7	5.38	0.88298548	1.13075857	1.02649371	0.96779271
43	309	34.9	9.29	1.11164275	1.052226	1.06741713	0.96614664
24	341	38.6	5.07	0.86107862	0.88287107	0.87150956	1.10170334
35	311	34.5	5.52	0.97155636	1.26870873	1.03694082	0.98477407
54	380	44.2	6.06	0.86185503	0.76821784	0.91345784	1.13166483
34	285	33	7.52	1.04154136	0.87549026	0.96334375	1.04287781
32	369	41	7.68	1.05248215	1.08221196	1.13311466	0.94312055
28	599	69.2	5.11	0.97469178	1.00652148	0.97080661	1.02517565
50	358	39.2	5.34	1.06381129	1.4617195	1.19569747	0.8429659
37	305	34.6	8.95	0.94739981	1.44190914	1.06613987	0.8900488
43	319	37.7	6.9	1.00932115	0.80692079	0.97428989	1.06877557
71	250	29.4	5.36	1.05887425	0.96597548	0.97682485	1.01207714
77	146	16	9.1	1.37658379	1.05940498	1.27412106	0.80582583
44	636	72.1	6.79	0.79928781	0.88143547	0.8142504	1.15457434
48	293	31.8	5.06	1.0234654	1.15039996	0.97947289	0.94423063
48	142	16.3	4.86	1.04294405	0.70841612	0.94230326	1.17394128
38	288	32.1	5.41	1.05870858	0.98742346	1.06880909	0.99208718
68	249	28.4	4.82	1.11286871	0.82923821	1.0495907	1.04637622
39	537	62.9	6.13	1.0574658	1.02170496	1.06416204	0.99125035
38	327	36.1	8.24	0.80032853	1.83014632	1.19512966	0.80916191
58	191	21.7	9.13	0.97534672	0.8522739	0.98731862	1.04307493
33	393	44.2	5.26	0.97593349	0.84160015	0.94133717	1.05124621
36	525	58.4	8.87	1.01746769	0.86752325	0.96452696	1.07137347
62	156	17.8	9.98	0.930266	0.7161514	0.91926942	1.14297683
24	443	48.6	8.69	1.15356439	1.07194412	1.04435277	0.99974802

30	398	43.5	5.24	2.3110167	0.98880752	1.44804185	0.4738509
30	319	34.9	4.83	1.02004482	0.8489746	0.95470724	1.08009277
34	426	50.2	7.24	0.88386975	1.13826648	0.92389222	1.03463269
24	876	101.7	5.11	1.31301618	1.0978144	1.1654289	0.86138635
63	103	11.3	4.79	0.91413571	0.90174887	0.94170726	1.09738509
97	103	11.8	4.51	1.17128915	1.35932191	1.08498193	0.86511119
57	117	13.5	6.2	1.02796064	0.96569751	0.99033909	1.0344438
49	310	34.6	6.11	1.10152232	0.92278888	0.97612206	1.00945125
44	388	42.9	5.66	0.97070407	1.01697777	1.04320544	0.98960994
59	183	21.4	5.45	0.95844473	1.00192713	0.98953936	1.03296927
29	247	28.8	5.07	0.85024366	1.19929885	0.86978077	1.04950993
73	172	20.2	5.05	1.05974444	0.91167949	0.9671209	1.03384223
53	108	12.2	4.41	1.01841108	0.97652048	1.04004778	0.98341978
32	231	25.8	6.24	1.14465472	0.82842091	1.13959467	1.01145735
34	294	33.4	9.14	0.97499335	0.97598109	0.98600211	1.02294804
33	729	79.4	4.83	1.22174254	0.99542396	1.0973229	0.91647312
56	66	7.5	4.77	0.9028803	1.62710151	1.22371067	0.72614905
36	466	52.1	4.69	0.95982782	1.07707033	1.02555366	1.01429202
86	102	11.3	9.77	0.89282957	0.65211385	0.81820294	1.16649104
71	351	39.6	5.07	0.97211133	0.83203087	0.98094636	1.0526725
79	50	5.9	4.26	1.41901876	1.56260392	1.15472147	0.72733392
50	97	11	4.46	0.94062832	0.97321176	0.99615869	1.03663944
28	288	33	5.01	1.00748913	1.11284332	1.08551915	0.95407779
40	329	36.2	5.08	1.26382894	1.19934113	1.2931035	0.83813144
35	246	28.3	7.08	1.52571459	1.48971709	1.23126846	0.70649147
41	228	25.6	5.01	1.27182515	1.10990592	1.20323269	0.85343833
43	588	67	5.01	1.506171	0.51799017	1.1013723	1.03490997
55	251	28.5	5.49	0.92086074	0.92105621	0.90537644	1.08787114
32	1482	170.8	5.34	0.94246455	0.99659821	0.9679239	1.03321948
38	213	23.9	5.62	0.90096217	0.95086415	0.93823616	1.07169558
42	271	29.8	5.25	1.05748308	0.92421085	0.96992975	1.03478427
28	2397	273.9	5.6	1.00058643	1.3086203	1.02591994	0.92348083
46	192	20.9	5.07	1.31536043	1.12854018	1.31868788	0.79954714
211	132	14.8	9.33	0.82349533	0.81391026	0.89808013	1.11835342
58	569	64.9	5.11	0.95581166	1.1925035	1.03799167	0.90825307
35	353	37.8	6.01	1.24985425	1.02080625	1.22872361	0.8701458
49	654	76	5.86	0.77478727	0.86287935	0.8543155	1.11696318
27	496	55.2	9.17	1.04365513	0.99285384	1.03865407	0.99619956
29	165	17.9	4.7	1.13623434	0.95803661	1.06338833	1.01617991
244	168	18.6	6.68	0.93804886	1.36906291	1.15088088	0.91085379
165	91	10.6	9.85	0.92749643	0.77567779	0.86889889	1.11476306
31	448	51	5.68	0.90929771	0.89678909	0.94679206	1.05241469
37	243	27.1	7.65	1.04057813	1.00983416	1.04251391	0.96956408
48	71	8.2	4.93	0.97693337	1.02372018	1.09794749	1.00131505
24	306	35.3	4.84	1.03072288	1.01631045	0.94518391	0.94140667
42	155	18.1	5.66	1.17566531	0.81311102	0.80583388	1.07943852
40	410	45.5	4.75	1.11699297	1.0837787	1.16741411	0.90373972
64	150	16.6	9.45	1.05443801	0.85934873	0.99668457	1.05611358
29	286	32.6	9.1	1.37447624	1.11047683	1.35091842	0.77076428
50	72	8.7	5.43	1.38820076	0.82394957	1.06714433	0.96316959

36	569	63.6	7.39	0.81522763	1.03722999	0.89673444	1.11203309
63	233	26.3	8.79	1.14709131	0.98288893	1.02783147	0.91919078
39	195	21.4	5	1.0297704	1.10367027	1.04250716	0.95712464
34	305	34.6	5.97	0.94644166	1.18775067	0.97267922	0.97268564
40	172	19.1	4.88	1.06666368	0.77310781	0.96315734	1.05233022
52	67	7.9	6	1.19023989	1.63178451	1.05811022	0.8032993
29	401	44.4	5.87	1.07007016	1.03196336	1.09300673	0.95616111
54	238	26.3	4.77	0.92922803	1.0894124	0.87455181	1.00683294
45	337	37.8	9.42	0.72030599	0.87662978	0.77695798	1.15696748
46	130	14.9	5.4	0.98802547	0.79934477	0.96771198	1.12198024
128	100	11.3	4.45	0.83831866	1.28635966	0.87805705	1.0154802
33	802	92.6	6.68	0.99872862	0.92730734	0.94477736	1.04814338
26	269	31.5	9.41	0.96039881	0.9775305	0.87113857	1.07732804
39	208	23.6	8.84	1.2578036	1.13949604	1.06353897	0.86098137
46	234	27.1	5.12	1.00766511	0.88828064	0.94229217	1.0766987
37	280	31	9.14	1.02850729	0.90465241	0.91198525	1.03893512
49	314	35.1	5.25	1.09525654	1.19708496	0.98894709	0.94780986
59	144	16.3	5.05	0.98037032	0.89833618	0.96174381	1.00487016
32	211	25.2	6.27	0.9804425	0.82824417	0.94448427	1.06394203
84	435	48.3	5.15	1.11315573	1.41528114	1.16263216	0.8394216
23	341	37.7	6.04	0.99102068	1.02824682	1.07283524	0.98229092
165	190	22.2	5.2	0.9263556	0.78779337	0.90318509	1.1035561
56	221	24	5.1	1.12532821	1.04555732	1.069639	0.93870674
57	156	17.5	5.86	1.02218955	0.75544117	0.92349236	1.13243133
251	94	10.3	5.91	1.04184531	1.01031933	0.91889736	1.01177502
35	407	46.2	7.28	0.97657844	0.87010232	0.9514024	1.06519164
37	335	37.5	5.81	0.94997469	0.86490298	0.93206704	1.08001268
35	544	60.5	5.1	0.98809872	1.21324566	1.06730932	0.98290781
29	504	56	9.39	1.05546916	0.97389563	1.03005252	1.01057782
147	414	45.5	5	0.77894866	0.37715429	0.62953149	1.44023737
47	211	23.6	5.73	1.01937405	0.92672573	1.02614611	0.9884034
48	167	18.5	5.14	0.95951392	0.86744765	0.91450362	1.07338371
34	423	48.1	6.11	1.14810814	1.32323613	1.17581001	0.84339116
38	384	44.1	8.76	0.96248696	0.9725085	0.949043	1.05159377
28	579	67.8	8.21	1.1011885	1.24991559	1.20762425	0.83728607
38	315	36	5.17	1.04802314	0.89503744	0.99415202	1.04833655
35	207	23.6	4.83	0.9665192	0.91400727	0.9324414	1.06001
82	151	17.2	5.68	1.10587439	1.10195583	1.03478935	0.98346844
33	448	51	9.47	1.04031698	0.8845381	1.0479834	1.02435771
40	342	37.8	9.31	1.09954496	1.08121631	1.1169233	0.91204085
35	408	46	6.64	1.32452753	1.34306161	1.15154331	0.80057877
82	662	75.2	8.03	1.15095938	0.87546598	1.22935299	0.90268569
51	266	31	5.81	1.10895521	1.13467459	1.07807703	0.92184202
46	223	25.4	5.87	1.04013737	0.92243081	1.03525434	0.99196156
140	143	15.3	9.23	0.89672204	0.87357392	0.88208191	1.09446841
34	582	65.9	5.97	1.06574274	0.97352287	1.04791499	0.96686385
40	172	19.1	4.88	0.95671528	0.8749819	0.86897364	1.1553559
36	515	59	7.02	1.00985875	0.94669154	1.02545342	0.98472684
35	295	32.6	4.92	1.32488682	1.12395767	1.28531449	0.79678938
166	66	7.4	4.54	1.50864026	2.08876255	1.01918835	0.6303287

22	439	50.2	6.84	1.00687617	1.13239935	1.01700584	0.95990687
36	339	39.3	5.2	1.09168251	1.01859893	1.04834724	0.96316835
28	274	31.7	6.1	0.95030543	1.22895293	0.9947299	0.92094664
41	430	47.2	5.16	0.92698864	1.05617034	0.92599927	0.99738194
28	170	19.2	4.7	1.23277224	1.39466958	1.20139515	0.80547201
25	453	50.2	6.49	0.74678433	0.984185	0.81875146	1.13547279
54	302	33.4	5.44	1.05891644	0.90998389	1.00711086	1.07664942
284	126	14.1	4.03	0.7893756	1.10837565	0.75106103	1.13068181
248	90	9.6	9.52	1.12750416	1.00643911	1.0650935	0.93634469
84	259	28	4.61	0.90288215	0.83929795	0.86631296	1.1112934
20	311	34.4	5.2	1.03816261	1.02510648	1.09284523	0.95868071
39	342	37	4.89	1.33766629	1.23009241	1.4902715	0.72171746
24	227	25.4	5.19	1.14284577	0.81587387	1.06994055	0.98635914
37	134	15.2	6	0.752743	0.67071785	0.73584175	1.21062925
37	266	30.5	5.47	0.80672932	0.9358041	0.85490404	1.07426515
77	84	9.7	8.44	1.03047562	0.64102067	0.90256121	1.14201514
68	605	71.2	6.4	0.88419391	1.00574865	0.89959944	1.08565951
28	281	31.9	5.9	1.11841996	0.97269998	1.19742435	0.9518022
276	135	14.7	5.35	1.63668264	1.51259766	1.0917556	0.63825278
41	306	35.5	7.77	0.93015698	1.00101928	0.95712512	1.0760333
37	655	73.7	5.26	0.95745209	0.91481552	0.96121658	1.04272046
37	118	13.6	5.97	0.98289778	0.77080742	0.96520954	1.05150148
27	414	48.4	4.16	1.01899485	0.98805983	0.97478869	1.02147047
49	215	23	7.23	0.50450532	0.48447622	0.48429501	1.36288768
34	327	37	9.66	1.26768998	1.11227229	1.19478512	0.86052739
31	290	33.8	9.06	0.9272013	0.90015704	0.89657264	1.084246
21	534	57.6	5.71	1.10656634	1.22669728	1.25111916	0.8588119
25	252	28.4	5.34	1.08920093	0.82617058	1.05667555	1.0293654
190	219	25.3	5.07	0.87859224	2.30031404	1.25039115	0.64004929
32	227	25	4.7	1.08688237	1.03503128	1.1180287	0.93828177
40	309	34.7	9.99	0.99107775	0.86341539	1.05282156	0.99110234
25	274	31.7	5.86	0.99430559	1.12852737	1.00044296	0.98107726
34	188	21.7	6.46	0.88696774	0.71779292	0.79764096	1.19557763
61	208	24	5.47	1.05115599	1.19792576	1.11409453	0.93863978
54	645	74.3	7.68	0.68783427	1.19705004	0.85427244	1.06507966
35	199	22.2	6.21	0.96134124	1.16238122	1.02087127	0.9457281
22	330	36.2	5.31	1.00012156	1.06841632	0.94769412	0.98772881
29	366	41.2	5.15	0.96334345	0.89318346	0.90741221	1.10696822
53	313	35.8	6.58	1.00287887	1.17192872	0.97199121	0.98392004
47	116	13.5	8.35	0.84701038	0.68866703	0.79979572	1.17685273
38	273	31.4	7.72	0.97995589	0.87854294	0.96301576	1.05319006
42	186	21.4	9.69	0.88625336	0.88814469	0.89224012	1.07528079
30	380	42.5	4.91	0.90464095	0.97079675	0.91057775	1.07498855
26	390	43.5	7.33	0.9012713	1.16377486	0.97583635	1.00975967
55	258	30.8	8.82	0.9987964	0.88524499	1.00557327	1.03183522
57	250	29.8	9.09	0.91851868	0.79939318	0.87965898	1.2196944
83	194	21.2	5.25	0.77046601	1.33587476	0.93716768	0.93466111
32	197	22.8	6.13	1.05199689	1.01695816	1.05105923	0.98135987
70	194	21.7	4.98	0.90146299	1.64644085	1.16370002	0.81664598
26	276	31.9	5.49	1.12150074	0.86323936	1.00829113	1.02458733

39	296	34.2	5.41	1.02464178	1.16416295	1.04407655	0.91545032
28	220	26.2	5.1	0.97106043	0.95048308	0.98068942	1.02496949
30	444	48.7	6.7	0.92693256	1.09612124	0.97046489	1.00283964
55	179	20.4	7.39	1.1675278	0.8917518	1.11273921	0.9646947
20	475	50.9	8.84	0.79623222	0.83376694	0.83833347	1.12852402
131	422	46.6	4.93	0.98160951	0.98666083	1.04403507	1.0413434
35	171	20	5.16	1.273449	1.13871554	1.0771806	0.88264786
45	436	49	5.3	1.17817455	1.0143945	1.13635571	0.92660368
40	352	40.1	5.72	0.93259711	0.97072501	0.89989542	1.07404951
22	324	37.5	5.27	0.88206161	0.9897388	0.91263858	1.0735106
39	305	34.7	6.73	1.06256814	1.35707317	1.12404453	0.90024827
38	124	13.4	6.06	1.09410664	0.89483535	1.05034381	0.99657138
31	313	35.1	4.97	0.99223319	0.9729745	0.97321887	1.02306865
38	247	28	5.26	0.90764611	0.96254381	0.99074614	1.05758937
86	145	16.1	4.61	1.06909498	1.00715979	0.97113624	1.01139252
124	104	11.5	9.61	0.95405755	0.84314656	0.92126137	1.06465076
76	87	9.9	4.84	1.57510937	1.45135065	1.68056546	0.54003257
18	262	30.2	7.77	0.99249284	0.82790139	0.94344592	1.09778332
24	422	47.6	4.83	0.88562442	1.02786536	0.9682844	1.04071538
22	309	36.1	6.98	1.12592034	0.9703981	1.05361142	0.98251237
29	496	58.3	6.81	1.0143685	1.08871844	0.99000606	1.01710182
28	448	51	9.6	1.17625239	0.9233231	1.15460407	0.88048933
26	658	76	6.15	0.83328775	0.94162708	0.87651559	1.08836376
29	478	55.3	6.51	1.07406686	0.96021996	1.07700506	0.98406816
39	275	31.9	5.81	0.92549115	1.08616707	0.95313665	1.03459586
25	504	56.1	5.53	0.84367362	1.40224231	0.87344866	1.03352603
213	124	13.3	4.37	1.05732749	0.78968656	1.03089779	1.03037058
28	792	88.5	8.75	0.87875818	1.16734617	0.99100953	0.99015818
54	640	71.8	5.62	0.9498371	1.74127064	1.07520886	0.81170445
27	247	28	8.24	1.05759622	0.93596527	1.07600268	0.98517673
28	432	47	5.52	1.00800538	0.91208817	1.00817101	1.02968302
22	760	82.3	5.81	0.89642791	1.6515164	0.95432482	0.91345913
21	236	27.1	6.73	1.11272277	0.97722368	1.05013524	0.97604248
89	182	21.1	9.58	1.07704678	0.92277918	1.06453187	1.01172882
26	332	36.9	7.05	1.17348913	1.05236948	1.18532114	0.89635771
38	188	20.8	6.04	1.01416117	0.90085296	1.02480749	1.03059919
36	1137	123.3	4.51	1.25893624	1.43386848	1.29164991	0.72940686
16	757	85.5	5.15	0.75531725	2.4210933	0.9896775	0.76823217
19	325	37.6	5.43	1.01350236	1.05763998	1.05258342	0.98605872
20	153	17.2	7.71	1.00841866	0.69448333	0.89208192	1.13350408
46	121	13.2	6.11	1.10004356	0.73691343	1.05334641	1.07483017
29	425	47.7	9.31	0.48389648	0.4831793	0.49551321	1.43438037
49	205	23.5	5	1.07266337	0.98922573	1.00554605	1.00704365
17	497	56.1	9.1	0.95065866	1.0035478	1.00267818	1.02291038
31	248	27.2	6.65	1.12467483	1.05186037	1.11011735	0.95853725
36	68	7.8	4.67	1.1186082	1.38792632	1.10349117	0.83738754
24	825	92.9	4.89	1.00350706	1.14064783	1.04382845	0.96563183
25	292	33.4	5.29	0.99008928	1.08617149	0.99865641	0.99557887
54	208	23.9	9.31	0.89981852	0.913127	0.84787867	1.09669693
29	254	30	6.46	0.91513786	0.83253449	0.92008412	1.12657894

24	338	39.4	9.35	0.93667784	0.93024979	0.98405768	1.03948642
496	66	7.3	4.56	1.36762084	2.27788889	0.76888964	0.70178159
22	188	21.1	5.41	1.63830757	1.49194983	1.47596358	0.56702688
75	415	45.4	4.92	0.83129327	1.37650844	0.95413023	1.01936577
30	358	40.2	6.52	1.08563409	0.91503873	1.07369842	0.96712633
49	292	33.5	5.24	0.92903854	1.02627105	0.92201929	1.04028203
25	193	21.5	9.2	0.85048762	0.89818142	0.91746797	1.08961155
62	102	11.3	4.75	0.96753891	1.19649861	1.10409263	0.94998997
79	122	13.1	9.99	1.08052944	0.64191764	0.84884334	1.17241536
35	304	34.6	7.31	0.94550562	1.08077034	1.02257423	1.02530063
33	570	64.9	5.43	0.92239959	0.87025603	0.93886076	1.10373342
23	267	29	5.96	1.03257976	0.888527	0.90901516	1.05212037
54	149	16.6	5.16	1.12422284	0.48500916	0.95257836	1.25122173
80	75	8.4	4.84	0.73543168	1.79685958	1.1657523	0.85501759
68	118	13.3	9.19	1.05717038	0.88381601	1.09848316	1.00691206
23	273	30.6	5.12	0.92144518	0.70496275	0.84017933	1.17617982
24	545	60.9	6.29	1.01305622	1.07714052	1.01221196	1.01775123
40	123	13.9	4.45	0.96824405	1.198428	1.12369658	0.89675425
252	49	5.8	5.01	0.97146669	1.72582125	1.07681853	0.74480467
26	305	35.3	9.28	0.98394178	0.91715288	1.02684746	1.02006931
20	174	19.9	4.91	0.9561197	1.1142557	1.12064435	0.98998832
39	209	23.5	5.68	1.05057208	1.22651398	1.10199462	0.90729488
29	337	37.9	5.87	1.03914652	0.95003069	1.05481504	0.99082987
25	193	22.8	5.6	1.05937104	1.00478624	1.02167504	0.98989644
84	88	9.5	4.67	1.26354299	1.02644878	1.16668686	0.90801555
34	154	17.9	5.21	1.12957802	1.04785836	1.06915092	0.95936853
34	157	17.4	8.85	1.00601022	0.67953262	0.87219531	1.13127446
28	197	23.4	5.94	1.03927751	0.9552562	1.02923071	1.01342881
25	325	36.1	7.05	1.22491167	0.80769566	1.100711	0.96947156
28	466	52.6	4.79	0.97009454	1.36704318	1.02936548	0.91589903
27	220	25.4	5.3	1.0820543	1.07100197	1.10500058	0.93697032
74	97	11	4.67	0.92602809	0.63414911	0.8915952	1.23511365
42	144	16.2	10.64	0.87341076	0.74320848	0.96784088	1.07255281
37	161	18.8	5.24	0.99949581	1.21510741	1.07479917	0.91977796
23	193	22.7	5.78	1.15644393	1.03767139	1.25152367	0.88335469
135	85	10.2	8.88	1.96099785	0.75940281	1.02490505	0.73147315
29	493	57.3	8.9	1.18089322	1.13323544	1.12470202	0.86116706
20	345	39.6	8.81	0.98645872	1.08570284	0.97168402	0.9877445
18	224	25.6	6.43	0.99942898	0.84607467	0.97885434	1.08343997
211	102	12	3.92	1.166805	1.06241829	1.0330085	0.89490828
29	335	37.6	5.97	0.99329494	0.94960783	0.91721627	1.05667066
29	701	79.8	5.4	1.03249254	1.15663845	1.07361561	0.93286446
21	243	27.9	7.2	0.97040336	1.14814125	0.95660917	0.96212153
30	304	34.8	5.86	1.07427589	1.11368574	1.1337054	0.92529521
24	251	28.5	6	1.13157242	0.83430575	1.0013373	1.05578574
27	160	17	5.94	1.37465792	1.33785364	1.34952321	0.72287362
27	207	23.1	4.91	1.11203718	0.75344886	0.96842293	1.07842505
27	140	15.9	4.97	0.94518612	0.7681982	0.90393525	1.13567362
19	174	19.9	4.86	0.94667951	1.22261406	0.99503257	0.96113558
25	374	42.9	5.36	0.85118949	0.98531298	0.89306369	1.06598566

38	313	36.2	7.09	1.00601926	1.39748156	1.02021592	0.90184248
26	257	28.7	7.9	1.00158632	1.1053842	1.00104924	0.98798451
25	148	17.3	5.6	0.97488009	1.19265284	0.99160754	0.95206983
22	100	11.5	4.63	1.02375688	0.92639033	1.03016151	1.01377915
70	984	111	5.01	0.95898817	1.41298184	1.0217341	0.89401792
34	309	35.5	5.86	1.08262281	1.50710911	1.31015629	0.71184772
31	266	30.4	5.24	1.08403412	1.00963879	0.92217209	1.08349281
25	98	11.5	4.34	1.0555698	0.93658645	1.02918085	1.01465601
21	263	29.4	5.06	0.90314408	0.95703501	0.9183466	1.08685272
35	207	23.5	6.15	0.94136442	0.88943519	0.95250648	1.07886496
57	62	7.2	8.4	1.09469057	0.93640324	1.01150947	1.01102927
16	189	22.1	5.49	1.08593671	0.85941433	1.02849483	1.04832547
30	72	8	5.97	1.0459915	1.55316096	1.98280004	0.56431805
33	289	33.5	5.86				
24	85	9.7	4.53	1.1096509	1.12503067	1.07349252	0.91912306
44	238	26.6	6.3	0.98887086	1.31996849	1.05642382	0.92013426
29	403	44.7	5.5	0.78721802	0.79411266	0.85708175	1.13743473
52	104	12.3	6.54	0.97225432	0.88183082	0.95529557	1.07102888
24	295	34.8	5.96	1.01984729	0.86375352	0.97083585	1.05519551
20	2189	238.6	5.27	1.15813718	1.35045165	1.27087736	0.82854858
32	268	30.1	5.62	1.05779221	0.93517839	0.99809565	1.03888795
19	444	52	5.85	0.90412099	0.93513651	0.93131013	1.07405232
42	130	14.6	10.56	0.84898461	0.88739784	0.87928584	1.15465285
42	221	24	5.33	1.25246886	0.7259435	0.94704746	1.0663273
25	281	31.8	5.9	0.8573857	1.11795485	0.9219311	1.02719432
86	310	32.9	5.35	1.11615186	1.15639007	1.14933972	0.93372263
95	62	7.3	4.84	1.23750327	1.02979785	0.97670269	0.99748525
15	266	30.3	6.38	0.98908021	0.96418781	0.95646643	1.03033956
31	127	14.9	8.32	0.98759821	1.26198629	1.05921345	0.93267773
20	284	32.8	7.77	0.83313719	0.85709641	0.88381299	1.12041039
24	226	26.6	8.54	1.03470124	1.00880469	1.05298424	0.98177413
127	119	13.1	9.95	0.91172972	0.7466933	0.85946477	1.14609408
19	742	85.1	5.69	1.19024054	1.05567422	1.13930807	0.90179484
25	279	32.2	6.92	0.95672796	1.02665085	1.07316176	1.01886921
21	473	51.1	5.5	1.02924778	0.94211541	0.98508147	1.05523003
30	106	12.1	5.07	1.76014931	1.1175699	1.4478564	0.6373984
126	94	10.5	5.07	0.97130683	0.84922201	0.9071977	1.08758215
20	208	23.3	5.08	0.82031655	0.92446767	0.81403843	1.14823367
84	62	6.9	6.52	1.27699564	1.00976099	1.03834701	0.95518866
26	161	18.5	9.16	0.91427039	0.9244933	0.95189539	1.05314681
26	256	28.9	8.41	0.94556954	1.0112742	0.99672496	1.02979071
36	239	27.3	6.8	1.08271484	1.0378302	1.07360147	0.94783767
22	366	41	8.02	0.97906482	0.91555058	0.97893255	1.02540075
29	119	12.5	4.63	0.97254664	1.05297494	1.02043941	1.01191877
25	282	31.8	4.92	1.10321691	1.10590789	1.03939453	0.93464005
24	185	21	5.1	1.05555228	0.84411833	0.90898132	1.08326347
48	146	16.1	5.97	0.97981178	0.63703074	0.94597058	1.09828135
22	228	24.5	4.92	1.10135333	0.80901213	0.83129421	1.1221392
16	272	29.3	5.11	1.09194764	0.9814767	0.92679523	1.04590789
18	269	30.8	8.07	0.91520412	0.98523334	0.98059654	1.01552981

26	224	26.1	5.14	1.07721154	0.91937642	1.04554838	0.9846244
36	288	33.6	8.85	0.87821025	1.00277624	0.88838957	1.11810238
23	428	48.1	9.07	1.00070366	0.78380768	0.93138563	1.0863352
262	258	28.8	4.59	0.77503609	1.191033	1.33395884	0.94553404
19	280	31.6	9.28	1.06989907	0.9208289	0.92978016	1.06085905
26	315	34.8	5.27	1.02572195	1.03594751	1.02976928	0.98971027
22	270	30.6	7.62	1.24117651	1.04927112	1.14752012	0.91952264
151	121	13.7	10.42	0.79765325	0.77850246	0.92600142	1.14213204
22	319	36.3	6.02	0.92139411	0.72937454	0.8868438	1.1536219
21	383	43.2	5.43	1.13211462	0.94763323	1.09029141	0.99087918
31	187	21	5.16	1.05024783	0.94835251	0.91212628	1.05411663
24	518	56.7	8.65	1.23955711	0.95677484	1.07597422	0.92427869
33	142	17.1	4.91	0.95690079	1.23648877	1.04635817	0.92185427
43	374	41.8	5.29	1.42098991	0.89418219	1.14829779	0.86470183
26	261	29.8	5.34	0.95863057	0.89890855	0.89231848	1.09184865
74	59	6.6	10.11	0.9002746	0.61507271	0.80875986	1.20989244
22	243	27.4	6.05	0.9854026	0.93120254	0.96047789	1.03625242
20	155	18	4.45	0.93339232	1.34794293	0.93725858	0.9991851
499	104	11.4	4.55	1.12718539	1.07614712	1.04505767	0.88460514
30	131	14.8	5.07	1.04379276	0.93058158	0.94651326	1.01346234
14	444	50.8	6.01	0.9747867	1.0732044	0.98655943	0.99306734
21	500	57.6	5.91	0.91643096	0.89129397	0.89611663	1.08765982
21	318	36.9	5.69	0.8010869	1.26665414	0.89198669	0.99033154
25	562	65.2	6.37	1.01496274	0.98086886	0.98932309	1.01647375
24	329	36.6	6.98	0.92859168	0.83164672	0.9432811	1.11000083
88	282	31.9	7.49	0.68829301	0.52010862	0.68931965	1.42478439
16	349	38.4	9.1	0.9903318	0.940395	0.97663873	1.04039928
19	478	54.7	5.52	1.09348469	0.95620501	1.0565516	1.00248661
18	453	51.7	6.93	1.01456676	1.0341306	0.99450791	1.0087098
29	647	75.1	4.98	1.09528669	0.75900672	0.99369269	1.09460759
20	313	34.8	9.35	1.19376796	1.18710585	1.2016148	0.8164448
38	349	38.8	5.34	0.94658866	0.87699452	0.90939155	1.07117977
21	840	96.7	8.79	0.78592796	0.92617867	0.84039533	1.1196092
16	307	35.9	4.73	0.98652112	1.0670826	0.9803867	1.00046438
21	290	33.5	8.21	0.94427617	1.07098987	0.9646298	1.01964746
43	378	42.2	5.44	1.10286123	1.03081923	1.02776528	0.97878051
27	645	71.1	9.07	0.72693594	0.79202509	0.65244329	1.21893984
23	675	79	9.09	1.06052626	0.88112318	1.01722042	1.06490467
34	288	33.5	8.28	1.02198106	0.93675538	0.93868171	1.07825568
25	157	17.5	9.74	1.19735164	0.85871149	1.0661114	0.94721396
22	247	27.9	5.02	0.79477884	0.80806321	0.78178565	1.19776553
23	230	27.2	6.19	1.23504706	0.80984187	1.12792124	1.00300063
18	365	40.6	8.09	1.06637098	1.06767578	1.11497937	0.94960017
29	240	27.8	8.02	0.93950579	0.90558447	0.9401507	1.07704036
23	120	13.3	7.43	1.00753012	0.68926726	0.97850932	1.07541334
115	102	11.6	9.64	0.83781457	0.7836191	0.92518218	1.09100268
46	83	9	10.51	1.05864476	0.71456143	0.85383286	1.16456111
18	585	67.2	6.16	1.01717183	0.96662334	1.05893731	0.98039809
23	494	56.5	7.72	0.95033731	1.1897345	1.04496488	0.96223551
40	72	8.1	6.02	0.89502476	0.88683667	0.90224546	1.0982757

106	60	6.7	8.35	1.168816	1.82405352	1.18806014	0.7410998
23	250	27.4	5.77	0.80824858	0.94521676	0.81529313	1.10220371
31	247	28	5.19	0.95974286	1.50861963	1.15173561	0.88107899
38	277	31.4	5.06	1.07972939	1.17780773	1.22617966	0.85244817
21	224	26	4.79	0.97938238	1.23389942	1.0300028	0.95257452
17	339	40	5.99	0.97063645	1.24017946	1.06058233	0.84594885
18	188	21	5.91	0.82558429	1.09721845	0.96743736	1.022625
35	168	18.5	5.62	1.14925066	1.06531272	1.16037183	0.90724778
29	149	17.2	6.21	0.82834964	1.36027501	1.0522822	0.9325224
28	901	102.6	6.29	0.73490753	1.01819566	0.81637004	1.13652515
23	257	29.3	5.24	1.2370896	0.77889703	0.99860633	1.06602001
24	553	60.6	6.43	0.98481805	1.07800468	1.08615277	0.95196419
22	330	36.7	9	0.90409128	0.9471884	0.91934788	1.05406394
29	435	50.1	6.14	1.01458722	1.08988665	1.06504687	0.96309602
24	249	27.7	9.74	0.84498187	1.03263914	0.88705631	1.07150735
24	217	25.7	5.12	0.80375385	0.78214639	0.81607546	1.21116498
22	250	28.6	5.12	1.06688983	1.01560923	1.10047701	0.95007137
49	77	8.4	4.49	1.0205732	1.312075	1.21196821	0.86637941
74	58	6.7	4.83	1.12943417	1.1474441	1.03048952	0.95808519
30	332	35.8	5.68	1.00434322	0.67297951	0.96422883	1.11119839
21	460	52.1	9.07	1.02971075	1.03294867	0.99936469	0.98109637
54	267	28.6	9.01	0.99924653	1.76187886	0.85228288	0.95532062
30	304	35.2	8.75	0.99121434	1.03147852	0.98706707	1.02206428
34	419	46.9	5.1	1.00322605	1.01664988	0.95483658	1.02084647
23	410	46	9.04	1.00162037	1.1894395	1.02291908	0.96255309
16	280	32.1	6	1.03045136	0.97132062	0.9904329	1.01719025
28	68	7.8	4.75	1.02527665	1.24022564	1.00832577	0.93048418
16	306	32.5	4.83	1.01056692	1.04370285	1.01405459	0.96675654
47	143	15.8	5.14	0.94814978	1.16807882	1.05971505	0.97925883
34	267	31	8.41	0.99109003	0.96218792	1.01857342	0.99390074
21	205	23.4	5.16	0.91930884	1.19812201	1.00173904	0.99048544
19	227	25.9	6.44	0.96894935	1.05250868	0.97766135	0.99532282
20	431	50.4	6.16	0.9107133	0.89877404	0.94705647	1.08452086
265	113	12.3	6.25	1.50421099	1.99486331	1.1513254	0.58557009
33	77	8.8	4.61	1.09299059	1.11108778	1.1298349	0.90559968
88	146	17	5	0.60329045	0.71172416	0.62479647	1.30304185
32	122	14.2	4.92	0.93732865	0.93315165	0.95072927	1.04839513
809	77	8.6	4.22	1.60725276	1.17427548	1.03107368	0.88564111
206	83	9.7	3.87	1.1747396	1.3061787	1.20889361	0.85479251
28	149	17.2	6	1.0745965	0.78781995	0.97858577	1.0511367
24	256	29.4	6.15	0.98756621	1.25131633	1.04276786	0.8935298
27	74	8.2	4.42	1.40665759	1.18071583	1.05418026	0.88959209
34	168	19.1	6.73	1.01046984	1.17339984	0.98865618	0.96105179
82	86	10	6.55	1.21175383	1.00069849	1.1521317	0.89892921
12	307	35.9	4.75	1.12430104	0.70277572	1.01276787	1.01045159
26	360	40.2	6.83	1.19979219	1.18448157	1.30712093	0.81795285
22	545	63.2	5.25	0.95348955	1.23669614	1.00034605	0.95001474
19	276	31.9	5.8	0.89542845	0.92639846	0.91958683	1.09759589
27	131	15.4	6.57	1.02030047	0.95699847	0.9731255	1.01306989
17	237	26.9	4.69	0.94240932	1.48138438	1.13345857	0.93349255

25	275	31.5	5.6	0.9308474	0.96094572	0.88956376	1.0514454
27	243	27.2	7.15	0.89006885	0.93824343	0.93208042	1.07911403
17	225	25	9.26	1.04844583	1.04837951	1.05682251	0.98111944
27	223	25.9	4.86	1.10268446	0.87316557	1.03414724	1.02250166
19	259	30.1	8.91	0.9951643	1.02462017	1.00375662	0.99821053
19	299	34	6.06	0.89379853	1.24628659	1.03093791	0.93221859
34	207	24	5.44	1.04881646	0.98976132	1.06335203	0.94476175
12	268	30.2	5.05	0.78498745	1.25108688	1.26149786	0.90786968
44	86	10.3	4.88	0.98205983	0.97470911	1.08405015	0.96973649
20	309	36.6	5.14	1.02442729	0.99616889	1.04919221	1.00608909
32	688	74.4	6.2	1.05502182	0.90159871	1.01588525	0.99321252
16	96	11.3	4.94	1.15502263	0.94737206	1.15881526	0.9334717
112	194	21.2	4.51	0.79145051	0.89708259	1.0350715	1.02722911
19	186	21.5	6.8	0.9732734	0.94553076	0.96429735	1.03688522
17	318	34.9	9.2	0.90444814	1.25801225	0.98150458	0.96480187
13	382	42.5	6.55	0.95071461	1.13451301	1.02512632	0.95146785
22	228	26.8	5.39	1.2268521	0.94710924	1.11705604	0.91725388
37	145	16.2	4.75	1.13935368	0.91991066	1.08439288	0.99130658
20	369	42.2	4.97	1.0205731	1.11152258	1.09724256	0.93799435
20	466	54.5	6.32	0.99956971	0.93783844	0.91044548	1.02518478
22	319	35	4.78	1.13814515	1.00157748	0.99940986	1.01942028
28	185	20.9	4.54	1.05026028	0.82186893	1.06831667	1.03300378
26	218	25	6.68	0.9186434	1.15955098	0.97316705	0.98815093
20	283	32.8	6	0.91038165	0.7816387	0.86221673	1.12797335
16	341	37.8	6.38	0.80066964	1.33528359	1.00652277	0.96915393
11	358	38.3	9.41	1.07267284	1.20034983	1.01172972	0.94063399
24	169	19.8	4.63	1.14440268	1.41676029	1.15017276	0.81019407
18	164	17.7	6.68	1.02562523	0.78291202	0.94100624	1.10137216
19	288	32.2	4.93	0.87294365	0.97987798	0.94805369	1.053909
16	251	29.2	6.74	0.9944575	1.18071412	1.0489087	0.96696933
29	65	7.5	4.36	1.04663028	1.01083353	0.90937359	1.07506417
32	94	10.6	5.45	1.07640982	1.51639706	1.18369321	0.78993932
31	115	13.5	4.37	0.80216626	0.99565725	0.85678897	1.10676079
18	175	19.2	8.37	1.00992448	0.99968762	1.01409776	1.01870698
19	255	28.5	5	0.89996022	0.88617011	0.90675744	1.11306726
609	77	8.6	4.12	1.50962074	1.31878363	1.06298957	0.80310242
19	156	18.2	7.78	1.04021246	0.89702507	0.99237527	1.03714652
19	242	27	8.22	0.86057832	0.66712494	0.81558744	1.23589176
24	159	18.3	9.16	0.86480559	0.60063347	0.80991225	1.22175295
25	548	60.5	9.16	0.95783018	0.78842969	0.94125007	1.10488803
11	234	24.9	6.01	0.81826534	0.92060423	0.80196568	1.13935878
14	253	29.1	5.15	0.95491431	0.87170724	1.01052556	1.06071175
32	188	20.9	5.38	1.1052825	1.14474329	1.125778	0.92059492
29	80	9.3	11	0.89002694	0.57646548	0.83227516	1.21588372
25	281	31.3	9.48	0.94418996	0.82025136	0.88272796	1.14393879
28	553	62.1	5.22	0.92474392	0.89551095	0.91430592	1.06051268
27	61	6.8	7.42	0.82036624	1.27392208	1.02811012	0.96310697
48	227	26.7	9.89	0.9894243	0.80718375	0.86597329	1.14817113
18	242	26.9	6.16	1.30645424	1.20111442	1.28721368	0.77648383
12	234	27.4	6.05	1.27529309	1.0951268	1.15929087	0.84724794

13	237	27.8	9.61	1.07213	1.02904604	1.04465833	0.96777433
16	208	22.5	4.48	1.02944553	1.11453563	1.47751357	0.82181315
25	244	27.4	5.68	0.94276072	1.01026671	0.95998548	1.02989247
31	117	13.4	4.79	0.94070768	0.94243697	0.90202296	1.09660065
18	480	51.2	8.84	0.8741582	0.61219888	0.8343501	1.13034057
14	104	11.4	4.91	1.15966358	1.27843466	1.0646442	0.90612666
17	662	72.9	5.06	0.93654855	1.33385533	1.0748739	0.9032006
12	578	64.8	7.23	1.11274519	0.81894591	0.90317092	1.07597009
99	116	13.4	11.5	0.81440666	0.5873273	0.9577964	1.12455857
16	967	112.1	6.64	0.89658736	0.97865153	0.94523067	1.04563288
86	154	16.4	4.81	0.73719023	1.22514726	0.90243321	1.13186978
26	144	16.8	4.94	0.86881168	0.87267542	0.88845495	1.10931409
20	80	8.7	4.48	1.1930707	1.22857879	1.08557706	0.88547262
25	221	24.8	5.24	0.97637062	1.26931646	0.97973533	0.95465655
19	213	23.9	6.15	1.03040773	1.14849861	1.08399678	0.9630254
27	334	37.6	5.6	0.90478175	1.09227663	0.990369	1.0072773
21	230	27.2	5.91	1.02405532	1.0255414	1.04218148	0.99487217
72	137	15.3	11.25	0.91096167	0.72214981	0.92050756	1.17776287
17	252	29.6	6.52	1.06095813	0.98778561	1.04095584	1.00949429
17	254	29.8	6.42	1.04086251	0.87748412	0.98547183	1.03418103
14	139	16.3	5.29	1.09907185	1.00079329	0.96602711	1.0102618
23	140	15.6	5.16	0.94147811	0.91878198	0.99079405	1.04113576
23	274	30.8	9.39	1.28421537	0.79655193	1.29347852	0.89125627
43	313	33.8	4.97	0.9671999	1.179451	0.89313175	1.08516655
19	293	32.8	5.07	1.00168727	1.00157326	0.97427825	1.02313994
86	541	57.5	4.65	0.88038188	1.00191884	1.02996141	1.03390339
25	154	17.8	7.3	0.92186885	0.88543688	0.8880052	1.07309432
21	325	36.8	9.28	1.23518558	1.03618147	1.4273385	0.78207166
23	57	6.5	10.01	0.93855472	0.70762244	0.86191427	1.15412934
32	133	14.8	4.51	0.85315688	0.92998772	0.90941556	1.07920137
209	77	8.9	3.93	1.49072906	1.52825618	1.07703544	0.75987926
86	497	56.1	6.04	1.04527002	0.96685171	1.08294342	0.93904968
17	237	26.9	4.74	1.11300539	0.81830063	0.94739288	1.08295306
16	246	28.1	4.72	1.0164418	1.0443145	0.99125658	1.0103341
17	200	22.3	6.23	1.03268325	0.83758754	0.96119927	1.04923749
22	267	30.2	5.01	0.99900468	0.97234294	0.99378385	1.00897396
22	160	17.7	8.7	0.9987637	0.99497709	1.01274663	1.00023089
33	397	45.1	4.74	1.02224255	0.99505295	1.04195357	0.97706915
26	232	27.2	9.2	1.01883063	1.10751243	0.90389421	1.04192698
25	190	22.1	5.39	1.08351201	0.89864901	0.98333858	1.02380263
15	153	17.4	5.63	1.08337694	0.96586754	1.1462917	0.95356034
12	214	25.3	6.8	0.99200098	0.75800115	0.94474651	1.03648994
18	432	48.5	8.48	0.85837069	0.9468388	0.91263978	1.0815972
36	407	45.2	4.65	1.08411608	1.28810105	1.0529019	0.91523496
24	339	38.4	5.73	1.05354309	0.9556212	1.00446661	1.02544322
10	412	45.5	5.41	0.99321541	0.79823277	0.95220353	1.10491255
14	214	23.9	5	1.13981857	1.19409474	0.98361659	0.92998528
76	64	7	5.22	1.26135116	2.02692718	1.23854915	0.67505699
22	290	33.4	9.32	1.09567923	0.93412318	0.97464397	1.02754874
17	237	27	4.65	1.23370678	0.90139157	1.14133402	0.94398104

17	397	46	9.66	1.05941727	1.029915	1.03911404	0.96095159
22	131	15.4	6.01	0.95080268	0.81412253	0.9591453	1.13103418
23	211	23.6	5	1.00518568	1.0179846	0.99212323	1.02012077
101	99	10.5	8.75	1.10844872	0.76985937	0.93784	1.12289426
20	355	38.8	7.03	0.98551152	0.98571661	1.00716914	0.99848827
15	167	19.1	4.97	0.86275155	0.99411674	0.87131807	1.05611787
33	203	22	5.15	1.80607543	1.40339298	1.71658107	0.46956106
19	140	15.9	4.64	1.07659126	1.02599048	1.08232974	0.97858107
28	603	69.1	9.51	0.83868932	0.77942717	0.92145813	1.11003052
22	59	6.3	5.17	1.03870942	0.99363651	1.00205613	1.00248985
18	151	17.7	7.06	0.9044054	0.71446436	0.76628597	1.20529374
19	126	14.9	9.26	1.17483401	0.94441735	1.04001933	1.00163908
71	166	19.4	6.02	1.03565374	0.72436736	0.98511126	1.09348869
21	121	14	9.74	1.08367022	1.17722982	1.12049973	0.8944808
17	314	35.9	8.76	0.95275757	1.01590521	1.07257492	0.96776982
16	341	38.5	6.14	1.07430155	1.10846679	1.02007373	0.94452559
13	462	49.5	9.28	1.13244499	0.79486963	0.97552839	1.08866493
108	91	10.2	9.92	0.83305898	0.65898567	0.80259038	1.16796487
13	532	56.6	9.14	2.01513871	0.63943873	1.60079563	0.60633553
27	264	29.7	9.19	0.92753157	0.78496409	0.9108571	1.11653268
17	364	41.3	4.74	0.92697154	1.25029301	1.00741575	0.96150373
13	265	28.2	6.62	1.0532628	1.24852428	0.96428509	0.96242449
24	358	39.2	5.58	0.89009407	0.75158239	0.86597223	1.14545301
73	71	7.4	8.57	1.03221622	0.62487353	0.91553345	1.13802259
24	168	20.1	5.21	0.91900852	1.27811619	0.86276605	1.04945007
18	283	32.6	6.98	1.01023242	0.77900919	0.97204868	1.13296015
14	120	13.5	9.55	0.90957066	0.93105541	0.94218061	1.06449784
15	346	40.4	7.87	0.92240299	0.91082084	0.92665242	1.06687514
15	457	54.4	5.34	1.06447184	0.81222751	1.03316216	1.0520899
303	44	5.2	5.11	1.55393856	1.99711803	0.95002529	0.7330814
23	610	69.4	8.18	0.88478251	0.98384996	0.98892241	1.01530014
14	400	46.4	5.38	0.97329708	0.79042366	0.90854953	1.12260372
40	144	16.2	10.54	0.9771017	0.85131238	1.09071403	1.04461509
17	177	20.1	9.04	1.03644896	0.98967426	1.00751369	0.98447901
32	78	8.8	4.7	0.90321804	0.97746437	0.79365401	1.17590788
14	412	47.3	7.08	0.90615152	0.89163491	0.85118038	1.100069
19	253	28.7	5.2	0.90154701	1.01007651	0.91262515	1.04048574
23	210	23.3	7.24	1.05711695	0.71835447	1.14343845	1.00202129
22	287	34.2	6.35	0.81325675	1.09362463	1.14458954	0.96024578
38	199	22.1	5.4	1.1356205	1.00729243	1.05045525	0.96569718
20	83	9	7.24	0.99925224	0.80490385	0.9396526	1.01020088
25	137	16.3	9.31	1.19308228	0.8959245	1.09135953	0.99789653
20	505	58.2	8.5	0.96770691	0.95617812	0.98600446	1.04476982
14	334	36.9	5.38	1.75592188	1.03314068	1.49100688	0.65913851
10	218	24.1	5.06	1.09569008	0.90135002	1.05902735	1.01412346
20	319	35.2	5.29	0.91167448	1.10213514	1.13853179	0.88219953
20	214	24.9	5.53	0.90782477	0.82752275	0.91517272	1.10477756
40	98	11.6	4.37	1.10064971	1.447124	1.15574199	0.86106078
19	177	20.5	7.15	1.13388072	0.94670074	1.06700614	0.96785851
24	239	25.2	4.86	1.23767573	1.59497994	1.28876919	0.73566641

23	493	56.3	7.05	0.99681153	0.89074744	1.01638495	1.0161266
17	263	29.9	6.7	0.86334121	0.82278394	0.82989581	1.16146174
16	560	64.3	5.58	0.83492429	0.83021068	0.82193536	1.1579015
16	134	15.4	4.82	0.89567363	1.14686324	0.98464763	0.98353763
18	167	19.4	5.27	1.02007066	0.79289709	0.92401701	1.12659934
45	473	52.8	4.83	1.00130602	1.28946987	1.08317359	0.86586707
22	58	6.8	5.01	1.02707403	1.48916932	0.95020395	0.93381267
21	216	25.8	5.07	1.19479356	0.94692562	1.09994558	0.95115794
35	123	13.1	4.64	0.84440287	0.77048489	0.86711459	1.1407355
14	266	30.5	5.31	0.8192354	0.88029223	0.80766111	1.14366159
16	354	40.3	6.32	0.97161446	1.04158623	1.00320428	1.02317271
12	334	38.1	8.76	0.97908432	0.9911298	1.06165277	1.02485648
15	530	56.2	9.31	1.19742275	0.75080582	0.99915517	1.01954529
51	58	6.6	9.94	2.21993411	0.73618687	1.26554431	0.59480013
39	133	14.8	9.6	0.87869992	0.68307022	0.87573402	1.15402109
23	77	8.9	4.56	1.16183704	1.34966597	1.11775202	0.85035072
17	319	36.1	9.25	1.11093935	0.88199418	1.00180866	1.0227198
14	111	12.1	4.54	0.67831248	0.30998598	0.61443012	1.50089094
20	249	27.7	9.72	0.59978191	0.81944399	0.6262725	1.16230698
19	353	41.1	6.92	0.98279899	0.95328763	1.02654386	1.02731234
23	230	26.6	7.15	1.04709526	0.87354963	1.00617513	1.02947511
17	324	37.9	6.46	1.04357674	1.02531756	1.02640024	1.01529436
14	222	24.9	5.4	0.84681196	0.94747524	0.95389743	1.01900596
14	196	22.7	7.42	1.10382241	0.95081437	1.02306747	1.00661265
17	204	22.4	4.91	1.04380844	0.99733351	0.93567434	1.04281683
19	869	95	6.05	1.38423901	1.28477166	1.29719313	0.75136955
12	644	75.5	9.07	0.96043235	0.89426359	0.98945423	1.05138336
15	312	35.5	4.78	1.17335009	1.0519991	1.15838607	0.90347554
29	281	30.9	5.24	0.96724728	1.13299731	1.02385217	0.96095822
17	126	14	6.55	0.97435467	0.89983585	1.03167675	1.01642308
16	136	15.9	6.13	0.94545587	1.42102217	0.96212225	0.94764408
15	373	40.1	5.3	0.83295796	1.10236021	0.5715132	1.23985592
32	452	52.2	5.83	1.05759852	1.04115079	1.00273043	0.9310918
16	106	12.4	4.82	1.00011838	1.1351957	1.05370238	0.93276666
15	405	45.6	8.92	0.97393793	1.07189619	1.06374682	0.96704312
90	78	9.1	4.03	0.85357576	1.08158568	0.97803295	0.99594595
14	252	29.3	6.35	1.06447858	1.02155569	1.0263945	0.98151993
17	246	27.3	5.33	0.88380824	1.09312329	0.9542443	1.02324728
17	185	22.5	5.64	0.87018234	0.81226315	0.90178092	1.1050825
14	207	23.9	5.62	0.99996155	1.04323276	1.00899795	1.01934893
18	176	19.8	5.05	0.96698846	1.07099547	0.97841078	0.96581213
10	2182	237.7	5.31	0.84194319	1.44694431	1.83048862	0.81761826
22	180	21.5	6.7	1.08210752	1.1074488	1.08299315	0.93525208
13	377	42.3	6.15	1.4635509	1.14117552	1.34189515	0.73979132
24	35	3.8	8.73	1.74404993	1.51868215	1.30173035	0.61458397
30	136	15.9	5.33	1.17989381	0.84118955	0.93442179	0.8994218
10	174	20.6	9.61	0.95339421	0.82002157	0.92001398	1.08178134
16	479	57	5.19	1.00197475	1.2601364	1.07270874	0.86993908
21	154	17.1	5.31	1.07641311	1.22704032	0.99100388	0.95186736
30	239	27.4	5.66	1.09840248	1.22290103	0.94335616	0.93263321

43	72	8.3	7.42	0.92765264	0.85091925	0.93742623	1.10430739
115	122	13.7	9.85	0.90268316	0.90764862	0.93850606	1.07156756
17	181	19.7	6.55	1.063298	1.19187313	1.06086769	0.87391868
18	328	37.9	6.34	1.02968369	1.00388505	0.9692754	1.02373524
11	271	31.3	4.88	1.09221626	0.91614812	1.12521861	0.97831335
17	240	27.5	9.01	1.53770801	1.38734059	1.23271765	0.68854801
12	319	35.9	8.35	1.29809637	1.04406252	1.24191113	0.8026965
23	467	52.4	5.15	1.04845121	1.27373292	1.15766676	0.86878086
11	485	53	9.01	1.35021453	1.0731061	1.25388962	0.82254683
24	271	31.7	8.98	0.95846998	0.86864312	0.97845811	1.04166166
80	73	8.6	9.77	0.90023615	0.77028594	0.89469391	1.11322122
25	158	18.4	5.1	0.99666478	0.92838994	1.07381751	0.99387779
31	215	24.9	7.84	1.10208879	1.26804426	1.0319912	0.93000641
24	130	14.9	5.38	0.99096702	1.08897227	0.93470941	1.05363087
14	383	43.9	7.84	0.96521027	1.15635772	0.99741857	0.98450709
18	167	19.7	10.04	1.00491366	1.03843365	0.9789405	1.00627589
35	147	15.8	7.65	1.48659437	0.87400833	1.07810872	0.57080248
20	294	33.1	9.57	1.02172175	1.00833247	0.96275109	1.01382287
13	273	32.1	5.63	1.21800931	0.84452085	1.11146305	0.98726305
12	336	37	8.57	0.722576	0.76067303	0.73847028	1.24159268
18	134	14.8	5.86	1.27145984	1.08154802	1.07507931	0.89257766
16	268	29.7	6.16	0.79473887	0.89628057	0.78200251	1.16335662
52	53	6.3	4.54	1.22269459	1.10393949	1.10449497	0.88838568
15	317	36	6.89	0.96124827	1.11392476	0.98650236	0.96636436
9	300	33.3	7.03	1.12446896	1.10914665	1.17914327	0.88889344
548	177	20	5.07	0.95174555	1.48570855	0.99179982	0.92561749
15	168	19.3	6.39	1.04234571	1.06034939	1.01290791	0.98046933
15	141	16.7	8.63	0.91707887	1.16535421	0.92903374	1.04680732
10	346	39	7.14	0.93044414	1.0608961	0.96993413	0.99868101
55	388	42.1	4.91	0.86645963	0.94355214	0.89952049	1.08556841
20	205	23.1	9.66	1.00459705	0.65495294	0.95196311	1.12908982
27	285	31.7	9.09	0.91125433	0.95330889	0.99626502	1.00807815
19	110	12.4	4.23	0.74789531	0.69447173	0.73675992	1.25189036
27	394	43	5.47	0.98085836	0.97082688	0.95853684	1.03318723
40	96	11	5.85	0.98797772	0.87142453	0.94769236	1.0454265
10	155	17.4	9.03	1.13196845	0.7936145	1.07477471	1.00396921
13	180	20.4	4.82	1.14490094	0.93389237	0.99894176	1.00630446
22	113	13.3	7.91	1.00829117	0.93510296	1.10116136	0.97611331
10	254	29.1	9.19	0.98788557	0.93407954	0.98136211	1.02875822
23	151	17.3	9.04	0.88429945	0.76540203	0.87583421	1.16085621
143	66	7.5	4.54	1.63612907	1.57986797	1.08145264	0.66023449
20	231	27.2	6.38	0.85094125	0.80768109	0.83638472	1.15831163
18	104	12.3	6.14	0.85544672	1.03853428	0.91134696	1.07741342
18	123	14.6	9.44	0.91654738	0.72400466	0.96211907	1.11056258
16	256	29.4	8.95	1.13810472	0.94036558	1.12486489	0.94758771
12	333	37.9	6.3	1.02999341	1.08022688	0.99990307	0.95922126
12	322	36.2	4.96	0.96082969	0.96135114	0.94146603	1.05550345
12	470	54.5	5.19	0.96656184	1.42783734	1.0409355	0.88253122
12	108	12.8	5.27	0.90834332	0.93357314	0.98508953	1.06847168
15	392	43	5.5	1.15401967	1.32352251	1.14049024	0.87155394

21	320	35	6.54	0.59434613	1.70930017	0.95933784	0.88946886
14	228	27	6.98	0.96282467	0.93110129	0.90116626	1.06606184
12	214	23.5	5.39	1.00548411	0.78581378	0.94323418	1.19435386
12	105	11.6	5.26	1.01808796	1.24710101	1.0545736	0.92827326
9	404	43.9	5.4	0.96254496	1.13589286	1.03973806	0.94348538
13	300	33.3	6.32	0.83273436	0.84755161	0.91330754	1.12023787
20	128	14.8	9.96	1.01279932	0.9796794	1.01233662	1.01904463
64	118	13.7	11.25	0.69526142	0.70648756	0.81070526	1.21101878
15	650	69.9	6.7	0.95576573	1.53982676	1.0604888	0.85935427
11	420	46.8	5.05	0.92483501	1.0261511	0.95290593	1.04201625
10	467	53.8	5.11	0.92391809	0.60737631	0.81422195	1.18784449
60	112	13.2	10.04	0.86973231	0.72878353	0.95598269	1.16980512
51	65	6.9	10.39	0.907923	1.12861256	0.97824668	0.89409776
12	239	26.5	9.13	1.06235119	0.70620823	0.94098063	1.11160889
20	207	23.7	7.14	0.96773136	0.97886093	0.97851263	1.02848411
11	106	12	7.75	1.01665439	1.14449458	0.97095399	1.01095975
14	191	22.4	5.62	1.33369626	1.2989296	1.18220754	0.80574049
18	347	39.6	5.59	0.9069618	1.02356315	0.91848236	1.03516011
21	166	20.1	4.39	0.99054486	0.95114504	1.01333928	1.00844433
9	73	8.3	5.06	1.13324649	0.99961537	1.0187496	1.05459455
16	265	29.5	6.04	0.94695221	1.06846206	0.93681398	1.00025405
17	153	17.8	6.33	0.91837465	0.92592293	0.85661882	1.08457622
14	267	31.3	8.22	1.08857339	0.88558585	1.04251938	1.04627224
11	356	39.7	7.65	1.09646222	1.27590895	1.09694089	0.90441327
17	198	22.1	5.14	0.97852033	0.87600069	0.93268086	1.03455977
28	120	13.3	5.27	1.19890451	1.26255308	1.20067523	0.83465565
17	593	68.6	9.33	1.04574278	1.31929072	1.04239959	0.88047385
12	431	51.3	8.98	0.98302396	0.8456857	0.94831845	1.05072992
13	397	45.6	5.33	0.96148944	1.24362617	1.00136934	0.94447496
9	127	15.4	5.24	1.02792466	0.88810531	1.01582529	1.03127544
23	170	19.9	6.9	1.11820955	1.01450142	1.16523963	0.90060076
21	539	63.1	4.84	1.10149271	1.09102876	1.11543472	0.93128397
12	450	51.9	6.61	1.06876283	0.83965778	1.01803402	1.03858198
12	242	28.8	9.58	0.9306025	0.99016537	1.01807262	0.98946317
12	387	43.5	6.02	1.11127209	1.06704524	1.14908826	0.91071276
17	72	8.8	11.53	1.06650692	1.47977125	1.06246912	0.83371702
14	167	19.3	5.33	0.92005792	0.84486449	0.89788239	1.10234299
17	346	41	7.08	0.95644851	1.05267426	0.96262085	1.02218264
13	240	26.6	5.45	1.14220009	1.26682515	1.19807124	0.86539444
12	337	37.2	6.02	1.0240083	0.99185477	1.02046947	1.00287604
48	154	17.1	4.56	0.87039576	1.08242189	0.99735946	1.04051142
167	116	12.6	6.25	1.11798101	3.82578513	0.73264058	0.52179464
13	200	22.5	5.48	1.25379367	0.91710163	0.9912357	0.99042744
21	427	46.2	5.1	0.97248206	1.02628884	0.89709195	1.01970099
15	159	18.3	6.33	0.92007028	1.54935446	0.90581731	0.90786564
61	91	10.3	10.05	1.233215	0.85470337	1.0509661	0.98044892
13	286	32.9	6.67	1.02811281	1.21612128	1.085644	0.93062253
16	488	53	8.88	0.92180838	1.23410004	1.03810662	0.95342103
52	54	6.4	4.84	1.26490195	1.34433407	1.05928518	0.86518083
34	315	34.2	4.88	1.04426883	1.05414441	1.04092498	0.95597256

66	184	20.3	5.06	1.05433117	1.09103824	1.09511574	0.87453584
8	118	13.8	5.92	0.91068593	0.91146333	0.9446296	1.03501063
10	431	48.1	9.16	1.00093801	1.04551469	1.06725048	0.95613824
13	142	16.1	5.83	0.95463475	0.88393107	0.9709063	1.04473008
7	307	35.2	9.26	0.7960663	0.89826117	0.85797369	1.11539992
63	122	14.4	5.01	1.05011046	0.85562119	1.04505336	1.03511473
13	291	33.9	8.19	0.94859033	1.03637464	1.00235013	0.99253127
34	106	12.1	4.7	0.98789256	1.21361641	0.99045912	0.96672168
16	207	23	7.49	0.92705638	0.88786126	0.90979559	1.09730973
18	341	38.4	9.92	1.32465634	0.8884531	1.39452855	0.82883021
22	282	32.2	4.74	1.0118449	0.898836	0.98204409	1.04800763
14	159	18.3	6.42	1.08208251	1.01459502	1.00484685	1.00483735
7	177	20.3	9.7	0.94110276	1.44031491	0.99532391	0.88009886
15	256	29.5	9.04	0.95076251	0.79722177	0.9253679	1.12619833
7	200	22.9	6.43	1.01654582	1.12549694	1.04926825	0.94887863
16	79	9.2	10.01	0.85711005	0.69868899	1.01253302	1.1184376
25	317	36	4.86	0.89495511	0.99887957	0.93975926	1.12597892
17	121	13.7	5.82	0.99729546	1.07195349	1.07533078	0.94685394
67	99	10.9	10.17	0.86601291	0.89177188	0.88436318	1.08897249
25	150	16.7	6.57	0.99239782	0.84654009	1.00985614	1.04245346
22	366	40.3	4.91	1.02638112	0.90475263	1.00394571	1.04733416
46	207	22.4	4.87	0.84981498	0.75960981	0.70781804	1.21691748
11	206	23.6	8.82	0.96732818	1.0241237	0.98857453	1.00289379
30	58	7	11.11	0.74781515	0.64002427	0.86947317	1.22110153
16	220	24.3	5.62	0.97756913	0.99841123	0.91857648	1.04324708
23	312	35.3	5.31	1.06971252	1.04142194	1.04450739	0.94926664
14	215	23.8	9.36	1.12755955	0.9987067	1.06816741	0.96959066
16	382	42.5	9.22	1.0605793	0.8911669	0.99116983	1.0291384
7	269	30	5.38	1.08207335	1.27697022	1.12645989	0.86215597
11	103	11.9	9.31	1.02092351	0.83223636	1.06502291	1.0380817
12	275	31.2	5.47	1.03415437	0.99387893	0.99129611	1.00815066
18	191	22.8	6.29	1.12808139	0.91411363	1.06938081	0.98533149
15	231	27.2	6.23	0.92276723	0.75749167	0.81551723	1.15778773
11	554	61.1	4.94	1.07763393	1.07848755	0.9943788	0.99453971
54	105	11.5	9.82	0.87216198	0.73747674	0.82914998	1.14206674
17	132	15.8	5.9	1.01634371	0.7832528	0.89681604	1.08624547
11	231	25.5	9.16	1.08709629	1.15005059	0.97930079	0.95628849
14	327	36.8	5.21	1.01107335	0.97903619	1.03962703	1.01227183
10	299	31	4.81	0.92300859	1.14372619	0.81865455	1.04013152
11	176	20.4	8.97	1.12254119	0.85314644	0.99671955	1.03054344
10	261	29.2	7.61	1.02523825	1.14101418	1.01667339	0.97569383
12	148	17.2	5.66	1.0568004	1.2473345	1.06974195	0.88860736
11	265	30.4	6.34	1.12617498	1.02050201	1.07035256	0.98876404
13	136	16	6.33	0.98908348	0.94553637	1.01075665	1.02315916
9	681	74.3	6.05	1.01325634	0.97766859	0.98420862	1.0358843
14	245	28.9	5.85	1.01647741	1.08016282	1.01742617	0.97537843
10	113	13.4	6.57	0.86685982	1.40009478	1.08211539	0.87207942
14	449	51.1	4.64	0.97523153	1.02021725	1.00474936	0.99566543
12	367	42.5	7.28	1.08990199	0.95465206	0.9864413	1.01457894
32	446	50.8	5.15	1.05656698	1.13183406	1.06017928	0.88821304

17	227	25	5.88	1.04102467	0.8410583	0.97672399	1.06467555
8	745	87.8	6.4	0.97736641	1.02060999	0.97342763	1.05431392
10	171	19.1	5.17	1.12486956	0.92662877	1.07884111	0.97096169
14	180	20.9	5.2	1.00111759	1.27564644	1.05061404	0.92086838
11	253	29.1	5.33	1.00328426	0.85324232	0.90396327	1.07685149
13	158	17.8	7.33	1.09096049	1.13464872	1.08804108	0.92011666
56	45	5.4	5.21	1.01897153	0.84850188	0.87617332	1.04795345
6	218	25	5.07	1.00463909	1.01240678	0.97411162	1.03645539
37	87	10.2	9.76	0.92970295	0.61082934	0.84880064	1.20004639
14	347	39	6.54	1.06079683	0.9516211	1.07730589	0.94617661
12	394	41.9	5.2	0.97070297	1.1799487	0.99187528	0.96413536
10	180	21.7	5.96	0.98934206	0.67015655	0.90569824	1.13993944
8	156	17.6	5.24	0.97441641	0.83801564	0.9987941	1.07808981
12	238	26.7	5.11	0.96355069	1.11405315	0.96657046	0.97582685
13	351	39.7	5.71	1.12121188	0.92167114	1.06795074	0.969734
12	151	17.6	4.2	0.91925736	1.12725138	0.97633746	1.00057276
13	91	9.7	4.75	1.62826173	1.17875294	1.16627928	0.81706899
11	701	81.8	6.52	1.10745596	1.63856219	0.95780352	0.86713012
18	93	11.1	8.97	0.86745511	0.93138405	0.86991544	1.08392574
6	162	18.4	4.86	0.95317747	0.91968686	0.98669403	1.15169861
16	87	10.8	5.88	0.97090995	1.08205539	0.99309371	0.98221979
12	174	20.2	5.63	1.09638696	1.17513827	1.14503393	0.89573273
14	246	28.1	4.68	1.04479585	0.80717371	0.96038534	1.08184468
12	466	51.2	7.2	1.03893671	1.06582155	1.09190289	0.9654441
10	501	56.1	5.52	0.84581508	0.83070052	0.85405652	1.09763803
9	246	28.1	9.67	0.80837399	0.60307205	0.81342505	1.15060548
13	199	23.1	8.97	1.14822955	0.95430823	1.1631966	0.92591261
23	484	55.7	5.1	0.9697222	0.99988988	0.94586391	1.00854644
37	276	30.1	10.9	0.93909818	1.06658742	1.03168025	0.98254405
11	225	25	9.28	1.04413041	0.97637464	1.01275276	0.98268208
13	88	10.3	8.46	0.99509341	0.94549609	1.00395111	1.02230336
10	156	17.1	8.09	0.9761067	1.03887691	0.97803032	1.03861828
9	318	35.7	9.41	1.056188	1.15045687	1.07096852	0.93612451
14	487	56.9	9.11	0.93667045	0.92940568	1.01233237	0.99046082
12	516	55.5	7.31	1.09936984	0.91973759	1.01492773	0.9797209
23	178	20.3	5.92	1.02743578	0.94946564	0.95446449	1.04709364
36	80	9.3	10.14	1.08088433	0.85797517	0.97717381	1.06698987
9	424	49.4	6.43	0.81227288	1.20277058	0.88913228	1.04481011
8	386	42.9	7.42	1.13812991	1.16057997	1.17178816	0.8738733
25	79	8.3	5.29	1.71441116	1.5008283	1.21488923	0.59812268
10	686	78.3	6.3	0.98532933	1.00472913	0.93582701	1.04874746
7	222	25.2	9.51	0.96865069	0.91134672	0.98674795	1.07033357
13	268	29.2	5.59	0.86985709	0.58926572	0.90520934	1.11408844
12	234	26.4	5.15	1.10545691	1.14119639	1.09416847	0.90673548
12	112	12.8	6.54	0.94100535	1.00822253	0.99543184	1.01440904
8	219	25.5	5.08	1.01008218	1.08788096	1.0613804	0.9514664
11	235	25.5	9.2	0.95689578	1.05477484	1.08972577	0.97218868
8	253	29.6	9.23	1.29083919	1.01392517	1.22701246	0.87844799
7	266	30.3	6.96	1.00734241	0.93664061	0.95854499	1.01285164
9	140	16	7.17	0.90026128	0.99021519	1.1090586	1.0905862

9	225	25.5	5.76	1.00381005	0.65126486	0.75866262	1.25636097
14	156	18.1	6.28	1.0648634	1.0767697	1.05407464	0.97259162
10	118	13.9	5.48	0.96046174	1.21212048	1.09278053	0.96437906
9	402	44.6	8.31	1.25761321	1.04900484	1.06910779	0.95803539
12	179	21.1	5.05	1.15872427	1.10728445	1.06982012	0.90101339
9	334	37.9	6.9	1.04914716	1.06412971	1.0905989	0.94171451
14	134	15.3	9.1	0.90561647	0.9351554	0.93896437	1.06542928
11	409	45.4	5.35	0.96202357	1.15594958	1.08281896	0.94307521
5	137	14.3	5.11	1.14791417	1.00958271	1.04868944	0.92695653
27	204	23.9	8.95	0.96955072	1.04704453	1.0106523	0.97920823
7	466	51.6	8.24	0.91025772	0.85537862	0.9679181	1.07479594
11	130	15.3	9.77	1.01499399	0.86648262	0.90315398	1.08193839
15	178	20.8	9.38	0.84960288	0.85027229	0.90779825	1.10311866
12	627	70	8.68	0.92784459	1.13201844	1.18748178	0.88385517
14	331	36.2	5.82	0.96499606	0.97707875	1.00744619	1.01185058
10	220	25.8	4.92	1.03798656	1.17529743	1.04723045	0.90942033
6	133	15.3	9.33	1.38968425	1.13882234	1.02683016	0.88709391
18	166	20.1	4.39				
9	378	42.7	9.26	0.95991473	0.96396017	0.94691726	1.06567108
18	347	40.1	6	1.0533711	1.10882592	1.11759146	0.91891825
9	641	73	8.35	0.91743871	1.06681945	0.88978974	1.05405619
8	140	15.9	5.14	1.02650654	1.06130769	1.04392909	0.96273533
11	270	29.5	6.54	1.2073975	1.12564903	1.20815837	0.87787532
15	304	34.8	6.04	0.74311586	1.27305313	1.39249051	0.7317645
8	301	33.5	5.15	0.99116345	1.05108151	0.91855512	1.0999595
16	174	18.9	6.62	0.85117515	1.06454146	0.86336056	1.02126358
12	95	11.2	4.63	0.95378962	1.38198551	1.06579635	0.88996713
11	74	9	5.12	0.98083697	1.082123	1.0023554	1.00903313
7	360	41.2	9.03	0.94922593	0.91516256	0.77080034	1.1502235
9	213	24.6	6.81	0.89371007	1.22464579	1.00762338	1.00252097
10	254	28.5	7.49	0.95295156	1.09977044	0.96078864	0.97872149
9	371	41.9	5.26	1.0591907	1.24768282	1.09170984	0.90255543
7	517	57.9	5.47	0.92938154	1.18947879	1.00002827	0.99015573
15	461	51.4	4.28	0.95642297	0.95277671	0.99555119	1.0199459
23	109	12.3	5.96	0.93926862	0.92754665	0.99975137	0.99903631
22	488	53	5.1	0.85249957	1.31268385	0.99975802	0.92728039
8	504	58	5.29	1.01064118	1.00970638	0.95521314	1.02766287
8	253	28.5	6.43	0.96653177	0.98904668	0.98674941	1.02114309
59	104	11.5	4.7	0.9546859	1.15149777	1.06067219	0.92095792
8	341	38.2	7.65	1.14126208	0.96880105	1.15626397	0.90943438
12	169	19.1	6.64	1.04216323	1.14086041	1.0464981	0.93290982
6	68	7.4	8.21	0.97536336	0.82858701	1.00308404	1.04376
11	130	15.7	8.06	0.89798322	0.77111037	0.94494442	1.12327731
9	116	13.7	5.02	1.09800592	0.87778043	1.0241346	0.99845354
28	149	16.8	4.79	0.43405748	0.49453538	0.48192598	1.38642084
12	178	20.6	4.92	0.9971877	0.99803413	0.99523122	0.95193198
18	165	18.8	9.8	0.96735538	0.83430704	0.93065222	1.09422305
14	338	38.4	4.87	0.96373073	0.92957084	0.98122675	1.03338506
9	96	10.8	9.41	1.05964867	1.00701317	1.02218692	0.92399082
12	516	59.4	6.29	0.84879593	0.84680642	0.87714288	1.10190829

14	181	19.5	6.2	0.98074532	0.96896157	1.04574667	0.98800545
8	222	25.4	5.66	0.99243615	0.95556595	1.07567913	1.04689435
24	585	69	9.54	1.16704441	1.17377366	1.14750573	0.91581781
12	113	13.6	8.75	0.93073156	0.90617252	0.90717778	1.07766489
15	351	40.2	8.69	1.28554558	0.9438278	1.26275625	0.86889346
22	916	104.6	5.31	1.09361413	0.86854954	0.92397432	1.07975664
11	308	35.5	5.68	0.90251145	1.12723025	1.00773226	0.95560911
10	348	40.6	5.64	1.02463642	1.00372434	0.98483695	1.02424702
13	144	16.6	7.18	0.98302638	1.0531581	0.99590623	0.98744225
15	363	40.7	9.79	0.99510286	0.78020815	0.9325288	1.09247156
7	72	8.3	5.36	1.02859284	0.84630558	0.94114897	1.1185273
10	215	24.5	9.58	1.08680508	1.04585024	1.03511628	0.97631455
13	157	16.8	9.04	1.52916914	1.49586869	1.16725102	0.71050282
11	265	29.4	7.15	0.86113147	0.79797401	0.8298576	1.14620282
14	160	18.4	6.68	0.8594961	1.15668996	0.90159785	1.03417933
13	184	21.4	5.22	0.98815995	0.96037203	1.06355055	0.97990112
13	131	14.4	7.75	1.04484272	1.49587009	1.0898655	0.84386715
7	715	81.7	5.22	0.84227865	1.08931568	0.91251485	0.87938324
22	101	10.7	5.43	1.87912061	1.18758887	0.8838996	0.82375157
19	261	30.2	8.25	0.88673506	1.03032378	1.01575549	1.07486977
33	65	7.2	5.97	1.37558987	1.01435359	1.21677659	0.76638588
9	202	24.1	5.35	0.94343553	1.12145534	0.99163913	0.96359832
19	934	105.5	5.58	0.45514511	1.07457266	0.62272057	1.15028045
22	112	11.9	4.31	0.4966444	0.73728098	0.60457442	1.38199955
16	122	14.2	8.18	0.89196013	1.03851005	1.02396223	0.9847122
12	427	47	6.87	0.80176155	0.80137661	0.82429044	1.15233519
12	85	9.8	4.81	1.15570021	1.31555318	1.10332147	0.93102896
42	252	26.7	5.16	1.01656891	1.66202715	1.12740751	0.82623535
11	351	38.9	7.14	0.71579413	3.20747676	0.81335231	0.69066519
13	120	13.6	5.99	0.88439127	0.9730007	0.97732004	1.06004574
13	256	29.8	9.13	1.03905997	0.94327074	1.07678852	1.00970949
14	69	7.8	4.42	1.09087775	1.49296667	0.81993927	0.93139339
18	83	9.4	4.18	1.11816888	1.10808411	0.95086139	0.98381871
13	190	21.7	6.55	0.94578013	1.0019753	0.95468801	1.05475302
8	868	100.4	9.64	0.89339432	0.94763649	0.94358449	1.07612755
15	783	88.5	6.1	1.132148	1.15040103	1.12253233	0.87518425
6	278	32.4	9.57	1.00079255	1.15401802	1.0117415	0.95930425
8	41	4.2	4.22	1.09833064	1.08479147	0.96731226	1.02021241
13	151	17.5	9.11	1.05606239	0.76701578	1.00285294	1.06537282
8	196	23.3	8.43	1.07104882	1.15896495	1.06238558	0.95296311
11	315	36	5.73	0.88834759	1.09958232	0.99483495	1.00624128
5	190	22.1	4.78	1.06109477	1.13832363	1.18513487	0.85843287
8	196	23.2	8.41	1.19214496	1.35592598	1.18476744	0.85073667
9	457	52.4	7.42	1.11879861	1.46343809	1.11141094	0.83209089
7	197	23	5.66	1.09655781	0.79183992	0.99336699	1.04379402
12	97	10.4	10.17	0.94064167	0.68822825	0.80155213	1.21390682
10	86	10.4	9.42	0.89968093	0.91634636	0.83274709	1.11722981
104	82	8.9	3.78	0.67363932	0.68911471	0.52096698	1.30588837
16	220	25.8	6.07	0.94860655	1.11693895	1.01890818	0.9905202
6	282	31.4	8.53	0.90413717	1.29819081	1.0298478	0.94103542

9	161	18.6	5.92	1.08048696	1.13204049	1.03750212	0.97692403
14	185	21.4	9.17	1.04020845	1.12056708	1.00613085	0.94973607
7	554	63.9	7.47	0.96059618	0.82225913	0.93970071	1.14872819
4	176	19.9	5.07	1.03616471	0.92659331	0.9151452	1.07314415
8	422	46.9	6.05	1.08086681	1.34165341	1.19008017	0.83334842
7	92	10.5	5.76	0.87272217	0.76640601	0.77497435	1.15780613
8	133	15.3	6.68	0.98051473	1.18353201	0.99836409	0.978736
12	497	54.2	9.07	0.9497545	0.78300583	1.00751636	1.06346844
8	165	19	5.25	0.92489313	1.2181886	0.93957535	0.98219535
7	328	38.4	6.9	0.99709257	0.79381262	1.0644197	1.01928789
18	260	30.1	8.91	0.98496307	1.34096322	1.16050636	0.81431915
8	246	27.6	5.03	0.99715712	0.84078669	0.92960045	1.05261366
16	79	9.2	6.79	0.92355617	0.96002838	0.90047027	1.07737447
13	291	32.3	9.33	0.67453898	0.69263975	0.71449777	1.23351273
8	434	47.1	4.81	0.82631441	1.83592684	1.06611763	0.81134077
10	222	24.5	4.96	0.93972779	1.05646279	1.03162759	0.98664684
16	351	40.5	9.45	1.80372318	1.46643851	0.72801192	0.79111441
42	358	40.2	4.93	1.12660537	1.34972094	1.08140049	0.87681844
8	291	32.7	8.75	0.92729795	1.4153431	0.97926157	0.96461457
20	77	9.2	4.35	1.13142513	1.52194522	1.05858606	0.86162271
6	222	25.4	6.55	0.95405196	1.17590283	1.01284465	0.95566851
9	177	20.5	5.47	0.99950256	0.96087925	0.9735081	1.00793709
9	204	23.4	8.53	1.22771063	0.91579831	1.02370797	0.99437816
3	486	52.1	8.91	0.91222062	0.98056236	1.01873805	0.94872871
13	391	43.6	5.99	1.02289034	0.94712282	0.96002574	1.01404404
4	497	55.2	4.94	0.60378494	0.6604751	0.43307638	1.52772458
14	207	24.4	9.57	0.80600394	0.69643102	0.82149767	1.18863431
21	409	47.6	8.65	1.21100314	0.89535941	0.91717242	1.06587579
19	121	14.5	9.96	0.95096592	1.00199334	0.97333982	1.05831578
5	520	57.2	9.42	0.73588888	1.14353033	0.89862377	1.07712881
42	189	21	4.67	0.91241645	1.13125081	0.94997543	1.02546958
17	152	17.4	5.66	0.99514071	0.90127344	0.94837858	1.05192054
12	242	27.7	8.97	0.91223944	0.74408365	0.92414357	1.08781421
13	452	53	5.94	0.96189009	1.09707934	1.01584144	1.03122053
11	134	14.9	5.49	0.88768391	0.98162436	0.90159118	1.07883018
19	329	38.1	5.35	1.26582563	0.57705838	1.02308325	1.07779883
5	374	43.5	8.29	1.04796817	0.95554977	0.996799	1.04916394
10	352	38.9	5.03	0.97700645	1.07729294	1.08249117	0.94733946
51	57	6.6	8.15	1.09982052	0.99878797	1.20915256	0.89882976
7	553	63.7	7.47	0.89421585	1.01300066	0.98858844	1.01739699
13	272	28.1	8.38	0.72786947	0.60376165	0.7305382	1.24402601
6	253	28.3	8.88	1.12174609	0.88087569	1.06433932	0.95914761
7	216	25.6	9.41	1.11753119	0.88596986	0.98681408	1.02734318
18	62	7	12.23	0.89418722	0.56736686	0.87584681	1.21026052
8	157	18.5	4.87	0.82232331	1.46812473	0.88107907	1.00779086
8	522	61.7	9.67	0.59730374	2.49672689	0.9973015	0.74101356
19	279	31.1	9.63	1.05552633	1.1511557	1.13291003	0.95497331
15	53	6.1	4.92	1.41507507	1.65528631	1.02560681	0.80662195
6	247	28.3	7.49	0.93374386	1.00393081	0.93611734	1.06657827
12	119	13.6	7.2	0.94417542	1.00893488	0.89225377	1.06033035

9	182	20.8	4.92	1.11685105	1.12851828	1.10723499	0.91560114
5	235	26	5.27	0.94620989	0.92258407	0.93105237	1.10122749
9	372	41.8	9.52	1.07874223	1.20809778	1.00782975	0.92411599
6	189	21.4	6.02	1.02320258	1.0173655	0.99209409	1.03259876
8	315	37.4	5.77	0.89269332	0.8264431	0.96169458	1.08056342
11	571	61.7	5.41	0.89131076	1.11375766	0.9073732	1.02644897
9	406	47.6	8.27	0.90403217	0.95879135	0.96335134	1.03590905
6	189	21.3	5.62	0.89348658	0.98445837	0.99071099	0.97182634
7	298	33.4	9.11	1.32837393	0.92002483	1.02585554	0.96312312
8	226	26.6	6.38	1.00645478	0.97031987	1.03468907	1.00094872
10	377	43.2	4.96	0.93213372	1.05830447	0.93679221	1.04304594
21	90	11	7.4	1.18456955	0.78623756	1.04693228	1.04418948
7	502	60.8	4.63	1.03537652	0.9031037	0.98293921	1.03766259
5	340	38.8	5.44	0.85602853	1.11425533	0.96264076	1.04329737
13	283	32.1	6.35	0.91769502	1.16075407	1.04463512	1.0076204
16	159	19	6.64	1.02662875	0.99436719	0.97908366	1.03434717
19	61	6.4	7.25	0.98240647	1.20097209	0.92025843	0.9298373
8	208	23	6.54	1.17472043	0.9472449	0.92302185	1.00679551
9	215	24.6	5.31	0.99228553	1.19508797	0.98503928	0.95288417
5	147	17	7.96	0.93245057	0.73260363	0.8859283	1.1005763
9	356	40.3	7.03	0.96491749	1.33363151	0.97741934	0.97863078
6	191	22.4	6.35	1.11585365	0.84478966	0.98927086	1.03583995
13	178	20.9	8.9	1.08554785	1.20226712	1.10924105	0.92284549
12	246	26.9	5.3	1.14735215	1.28299582	1.10467382	0.85321398
14	61	7.2	5.9	1.21388	1.50275985	1.04068674	0.85924361
7	624	72.9	6.8	1.1427406	1.38765908	1.18112136	0.81098832
14	370	42.4	6.48	1.09986824	1.21683742	0.94194107	0.97821729
10	376	42.7	4.97	0.83038751	0.83288851	0.9684774	1.05327729
65	115	12.3	4.79	1.01164673	1.28903767	1.16067901	0.85308557
14	166	19.6	5.36	1.12900959	1.18346346	1.1576915	0.87857659
6	171	18.1	8.87	1.04246498	1.12840736	1.09439008	0.99505719
8	556	60.9	9.67	0.88258366	0.94053602	0.95284417	1.06813794
7	286	34.8	9.57	1.07877129	0.98176232	1.01023692	1.01270285
7	369	42.2	5.41	0.86535146	0.95237004	0.89021312	1.06649456
22	85	9.9	9.54	0.92484539	1.2299179	1.00395871	0.96715476
10	417	47.6	8.34	1.05321643	1.10102604	0.99760789	0.92371117
11	186	21.2	6.1	0.86565442	0.93607703	0.83440389	1.1325167
7	824	90.8	6.74	0.80860476	1.45859182	0.97976739	0.94863312
10	484	50.6	8.87	1.09577573	1.25472906	1.02419068	0.95140756
9	199	22.5	5.92	1.10991171	1.13179298	1.029825	0.97593651
8	104	11.4	4.82	1.085752	1.76669681	1.2076067	0.81539497
10	348	38.4	5.17	1.08351792	1.04770154	1.01885072	1.00121836
5	127	14	5.47	0.92580567	1.11795197	0.8974698	1.03736811
14	91	10.7	5.57	2.22547393	1.43096575	1.09585712	0.56982595
6	295	34.1	9.04	1.0004651	1.13087441	0.97659411	1.00960468
7	498	54.8	9.39	0.79514556	0.54731507	0.79353254	1.2439718
9	319	35.1	8.69	1.16533112	1.19923276	1.15916272	0.90654104
10	553	61.9	9.38	0.75138416	0.65602301	0.68626232	1.28193985
7	357	41.4	7.99	1.12867845	1.22546342	1.04499675	0.90821127
33	93	10.7	9.99	0.96039273	0.88311507	0.92836805	1.08196674

7	67	7.7	5.74	1.06752622	0.94438015	0.96314757	1.03916239
8	502	58.6	6.68	0.9894837	0.81256953	0.88997426	1.1079558
7	148	17.4	8.75	1.19605375	1.25077963	1.26244978	0.79624742
5	330	37	5.59	0.78699976	1.12049237	0.95527891	1.04424657
15	87	9.9	9.7	0.92629584	0.95862752	0.907556	1.08789701
7	706	83.5	5.99	1.00989238	1.15560324	1.02476521	0.94234844
14	202	23.1	4.65	1.37734007	1.24357152	1.15192847	0.81730044
12	729	83.6	8.29	0.96994164	1.04982963	1.28862785	0.94433819
5	511	58	5.11	1.07683016	0.907821	1.04209051	1.00098884
4	105	11.5	9.67	0.92135867	0.91897174	1.01354408	1.06111929
14	158	18	5.07	0.90822662	1.10292285	0.99248831	1.00219072
6	217	25.1	8.6	0.95567403	1.0452574	1.05374296	1.02246376
8	208	23.4	9.28	1.08746845	1.07198672	0.99580008	0.98732375
11	140	16.1	5.49	1.14361759	1.0388293	1.02840459	0.96902338
9	178	20	6.71	0.88717872	0.94331148	0.89374503	1.04650676
7	302	33	6.14	1.07967911	1.239675	1.17880156	0.8584209
11	188	21.3	6.43	0.88052442	0.8362877	0.97668474	1.06780741
6	293	32.2	9.29	1.01781485	1.12952156	1.06582451	0.95063235
6	343	38.5	5.21	1.05277846	1.36931183	1.15401949	0.88780036
6	245	28.1	5.63	1.12755797	0.99409813	1.15391071	0.92181095
11	130	15.4	9.09	1.1053168	1.03502719	1.07486441	0.9132391
15	413	45.5	5.38	0.97105939	0.83310708	1.02299231	1.08806094
5	288	33.4	4.88	1.03609631	1.00654731	1.07073632	0.98532688
5	282	33.3	6.76	1.1316534	0.99784362	1.03176401	0.97414046
15	91	10.8	10.51	0.97055127	0.67765849	0.79089616	1.20592275
5	48	5.4	10.46	0.93150663	0.82249368	0.88950704	1.15233031
4	362	40	4.25	1.12341065	1.20342962	1.58962687	0.75889937
12	175	20.2	9.73	0.95550998	0.71024414	0.90714641	1.13115129
7	261	30.2	7.94	0.8309479	1.24300279	0.97533247	0.991069
6	374	42.8	6.16	0.90098663	1.04162086	0.95288654	1.03398364
7	76	9	9.52	1.01479211	1.04419047	1.03317571	0.98952748
18	150	16.4	4.49	0.96641976	0.88289216	1.06752663	1.02650368
19	72	8.6	4.67	0.91812651	1.21759212	1.07711465	0.8765803
10	448	51	6.2	0.81233866	0.91040371	0.92958847	1.06156635
7	123	13.6	10.24	1.05227706	0.94756487	0.99452635	1.01635854
8	177	20.7	6.44	0.94179984	0.67496356	0.88120456	1.17931291
6	380	41.3	5.83	0.95989816	1.01339851	1.00053897	0.99181183
43	66	7.3	4.59	1.41493547	1.21195098	0.97143732	0.87462709
20	156	17.6	5.66	0.75558658	1.40953844	1.30590597	0.85210817
10	452	48.1	8.7	0.76214702	0.78765954	0.8221856	1.14451592
4	113	13.4	9.55	0.96508857	0.86897695	1.00503687	1.07032819
7	464	52.4	6.55	1.04107752	1.43955082	1.3032419	0.74486869
6	258	27.9	9.54	0.90829376	0.86035947	0.89440569	1.08483229
5	231	24.1	5.27	0.98210113	1.55045148	0.99496021	0.88272463
5	158	18.2	5.76	1.03561642	1.37904224	1.05487873	0.89524445
8	630	70.7	8.35	0.91055314	0.92991317	1.0152413	1.02760356
8	99	11.5	5.45	0.92713174	0.92370123	0.86865822	1.0985022
4	202	22.6	6.2	0.84193781	1.28431658	0.90620623	1.02450695
22	82	8.9	3.65	0.86802108	1.18978395	0.93717635	1.02315886
5	123	13.9	5.07	0.9139704	0.67292033	0.88907031	0.98281869

10	232	25.2	8.82	0.73427562	1.20075096	1.81339149	0.77192429
10	216	26	9.25	1.11828143	1.0001877	1.03325827	0.99699003
5	83	9.8	4.83	0.83826496	1.12276448	0.94540705	0.98811848
10	133	14.7	4.64	1.23027021	0.98919474	1.19597547	0.91149249
6	593	68.2	6.98	0.95846989	1.13478217	1.06439041	0.93439226
5	140	15.5	4.96	0.73236295	1.05967494	0.85749375	1.14907338
10	238	27.3	6.06	1.02681238	0.98961211	1.03444921	1.00850243
5	802	92.7	7.12	0.97479032	1.19184859	0.98026206	0.95472174
6	78	8.7	4.08	1.2405532	0.92149675	1.07972556	0.94015569
17	63	6.7	6.8	1.67522837	1.5449349	1.10362548	0.69456069
14	201	23.1	6.39	0.84058486	1.81731829	0.83627989	0.96424823
9	267	30.7	5.38	0.99922063	1.37657952	1.16303933	0.86019903
4	251	27.8	8.72	0.98979985	1.17037638	1.07530352	0.93010724
370	77	8.6	4.12	1.12707035	1.36289713	1.17534319	0.85599531
17	425	47.5	9.48	0.84521261	1.13280282	0.94988174	1.02266193
8	298	35.3	9.57	0.98667744	0.96852382	1.02128044	1.01445268
9	153	17.5	5.19	0.98812644	0.88434778	0.92802782	1.09384314
9	692	79.3	9.01	0.97601847	1.04171168	1.02087451	0.96357102
9	125	14.7	9.03	0.84482718	0.90248414	0.88733072	1.11931662
8	115	13.3	5.55	0.96344315	0.91359729	0.87731361	1.08197874
7	304	33.2	5.71	1.29684122	0.94093602	1.2320373	0.87260335
6	144	15.8	9.29	1.14862774	1.60421468	0.96027184	0.83552211
7	51	5.7	4.72	1.07682508	0.94789342	0.96298851	0.99646605
8	313	35.4	5.44	0.92302201	0.95075923	0.9694444	1.04347393
8	431	50.3	9.14	0.99014855	0.8542549	0.92304978	1.0444898
8	214	24.5	5.63	1.1395424	0.87059724	1.13349167	0.95329462
8	162	18.9	5.11	1.04740316	1.12780517	1.06619372	0.93298308
7	460	53.9	8.12	0.97036858	1.04739916	1.03093294	0.98403832
8	226	24.6	5.19	0.90069925	1.00388187	0.88230432	1.06800336
20	357	40.1	4.83	0.96480136	1.03932819	1.00461045	1.05100024
12	96	10.8	5.78	1.05059469	1.06331339	0.99232067	0.99971699
6	430	47.2	9.86	0.97164208	0.78854534	0.97474892	1.06415036
6	144	16.5	5.08	1.47368083	0.86854903	1.10622358	0.91322299
10	84	9.8	5.17	1.23056796	1.25640596	0.95165533	0.82491297
6	801	89.3	9.13	0.8766992	0.72429128	0.97874753	1.14887679
7	301	34.5	9.28	0.83809899	0.95647198	0.88145906	1.06287094
13	270	31.5	9.28	0.99412801	0.6604645	0.98392095	1.14452611
7	292	33.5	5.64	1.01849837	0.9086402	1.04094688	1.02752301
6	512	56	9.31	0.76928355	0.68691889	0.94299504	1.141801
3	62	7.2	9.13	0.94362143	1.18290936	1.02999854	0.96184577
11	293	33	5.15	1.16829129	1.60464861	1.1720105	0.80315705
6	201	22.9	6.2	1.12496919	0.91339947	1.02329113	0.99459815
7	340	39	6.6	0.84998932	1.40068834	0.87627995	1.02939205
5	278	31.7	5	1.088541	0.81422963	0.95855842	1.06007632
3	129	14.5	4.98	1.00656932	0.75647082	0.95868324	1.11096574
6	328	37.4	9.25	0.93001946	0.96757522	0.96344699	1.04082189
12	121	14.3	5.76	1.00216471	0.88405067	0.90401349	1.11396173
10	222	25.7	4.78	1.03314167	1.12759962	1.02256905	0.91641428
5	243	28.7	4.88	0.795251	1.08399642	0.99255151	1.00914173
7	202	23.8	6.52	0.94592891	1.51141175	1.10188436	0.8353491

8	306	34.6	6.25	1.09738079	0.93450594	0.99997173	1.04050306
10	154	17.7	10.02	0.84874947	1.12441122	0.95006337	1.04685523
7	307	34.5	6.13	1.44633879	1.1671477	1.14569727	0.82746344
8	247	28.4	5.01	1.05969091	0.84306301	0.98364389	1.06225545
8	262	29.3	9.47	0.7952598	1.35967963	0.81828506	1.00813465
7	325	37	5.71	0.98176015	1.37427777	1.10387508	0.88789365
4	269	29.5	8.82	0.98716554	0.75321651	1.00473702	1.04964695
15	67	7.4	9.99	0.93228431	0.53881677	0.86534489	1.21709178
5	65	7.5	4.78	1.00712655	1.16943584	1.02240061	0.94674359
13	198	21.8	4.55	0.97104593	1.0053551	0.99484028	1.03226469
10	127	14	9.5	0.96582177	1.04395902	0.96524041	0.96789052
3	278	31.9	8.15	1.23981095	1.03069361	0.94632368	0.97373419
11	268	30.9	10.05	1.02981143	0.98818905	1.0587309	0.99198836
6	164	20	4.39	0.8761913	0.89197696	0.88475183	1.12074355
7	104	12.7	8.18	0.95206203	0.88154317	0.95264436	1.10725485
10	501	54.8	9.52	0.93590985	0.77640182	0.99923326	1.05294066
9	220	24.5	5.24	0.80092158	0.58489779	0.6977963	1.27717687
6	642	71.8	8.88	0.93850171	1.34854987	1.84708083	0.66474531
2	153	16.9	9.79	0.94629296	0.67310981	0.86188919	1.16526367
6	150	17.1	4.87	1.04311421	1.01304867	1.037109	0.97465614
9	169	20.2	6.54	1.05750361	0.96677202	1.13259964	0.96492922
9	40	4.6	9.58	1.56876626	1.0233175	1.40100382	0.69912559
7	126	14.3	6.29	0.91187454	1.01983803	0.90621014	1.05647134
5	474	52	9.09	0.84699578	0.75666951	0.84555281	1.22145507
5	139	16.2	6.33	0.98364033	0.87571705	0.89966917	1.12475184
7	609	67.6	9.48	0.91343232	0.9654765	0.99523592	1.00262642
6	243	27.8	9.09	1.02984126	1.07871688	1.00383143	0.975669
20	292	33.4	5.29	1.00651214	1.53693178	1.13724434	0.80332538
5	469	52.6	9.35	0.71386183	0.68974832	0.79255408	1.24811164
9	238	27	5.17	1.06848702	0.97112151	1.02545922	0.95441077
7	185	21.4	4.42	0.9576072	0.79813468	0.72749586	1.14212588
3	453	50.5	9.51	0.82791995	0.63378888	1.00190816	1.12865447
11	437	49.3	5.2	1.05411875	0.84547474	0.91935153	1.13286482
34	37	4.3	10.3	0.89366341	0.86529319	0.94315312	1.10075069
5	120	14.4	6.19	1.02284817	1.21075729	1.14889892	0.87365571
5	230	25.8	5.06	0.79454354	0.9140888	0.84768469	1.12768551
11	165	18.8	9.8	1.04312415	0.89255591	0.92901192	1.07570801
8	75	8.7	5.76	0.77096299	2.93935645	1.15447789	0.57668871
4	219	25.2	8.43	1.11366168	0.96290002	1.05396551	0.99827646
12	91	10.4	5.17	0.98469209	1.02127981	1.01377174	1.01640891
6	234	26	4.94	1.11245664	0.8663505	0.99611488	1.02115503
6	67	7.8	9.85	1.00606622	1.12370935	0.97409924	0.9865232
4	379	43.3	9.38	1.02361908	1.01232607	1.00297384	0.98878272
9	239	28.2	4.93	0.92827506	0.94974001	0.85750027	1.07548372
6	184	22	7.37	1.19493365	0.94212493	1.0707119	0.94282517
5	384	42.9	5.4	1.12188921	1.01353173	1.14374281	0.94517615
5	383	41.6	6.65	1.0891822	1.27068779	1.15262153	0.8486863
4	509	55.7	5.88	1.22624252	1.29474873	1.29880678	0.77548901
7	86	10.2	4.32	0.95560814	1.04444646	0.98084713	0.92937104
3	450	49.6	7.87	0.95964272	0.76474655	1.03739391	1.05340271

5	140	15.4	4.92	1.01092353	1.4863448	1.19418462	0.79716438
5	218	25.4	9.54	1.07692066	0.73456651	0.97699333	1.06974428
8	316	35.5	6.86	1.05267092	0.98957028	0.97686004	1.00854948
16	283	31	5.16	0.93235496	1.41132813	0.9892707	0.89243742
3	142	16.7	6.95	0.95420368	0.97240498	0.91750968	1.02120939
5	212	24.5	5.78	0.92956597	1.12709414	0.93824374	1.02756315
5	535	59.6	6.32	0.88042383	1.14120101	0.98063104	0.99368651
5	94	11.2	4.72	0.89783647	1.07351548	1.02300487	0.93012491
12	321	34.9	5.99	1.05277395	1.11072388	1.1736268	0.87893243
5	502	58.2	5.29	0.97725537	1.11117768	1.00051812	0.96630456
5	311	33.8	4.97	0.99275025	0.8938488	0.95168313	1.04019395
8	91	10.4	5.06	0.93221549	2.18533818	0.65168156	0.890944
5	312	34.3	9.55	0.90240965	0.57293578	0.85839796	1.19533327
7	158	18.1	7.33	1.02475458	0.81228683	0.93259807	1.01255516
5	629	71.6	9.48	0.97495683	0.73863153	0.9079093	1.12486214
16	310	34.7	4.79	1.05796713	0.73641554	0.95431832	1.09155233
5	322	36.9	8.63	1.02930084	1.28935055	1.11846946	0.83497171
5	121	14.1	7.62	1.04148114	1.07672321	0.93871379	0.99854888
2	328	37.1	7.99	1.54288227	1.40217502	1.17855798	0.78365431
8	207	23.3	5.22	1.24891455	0.94007272	1.08417508	0.97292666
7	456	49.9	7.97	1.27071263	1.18677262	1.23819101	0.83839692
5	391	44.5	8.38	0.98570678	0.94203432	1.15269194	0.95501551
4	445	51	6.32	0.96902645	1.30602408	0.99604484	0.96419593
9	94	10.4	4.53	1.01594749	1.13630975	1.23327841	0.85453675
6	92	10.2	6.04	0.93765709	0.91383419	0.96161656	1.10784672
4	97	11.2	6.3	1.1547871	1.04671622	1.13789821	0.87591141
9	314	36.3	5.07	0.73312714	0.90689707	0.79206711	1.15629739
41	257	29.3	5.02	1.48202638	1.36416991	1.15374614	0.64901315
6	153	17.7	5.22	1.03348298	1.07727648	1.00856004	0.97028459
6	302	34.8	6.46	1.00573601	0.82854998	0.90195375	1.08712635
6	270	28.8	9.89	0.91191603	0.902399	0.95750336	1.08576071
4	831	95.5	5.49	1.0970588	1.10997889	1.11597949	0.93040366
5	157	18.1	5.4	0.9403985	1.01535762	0.87922573	1.07037504
5	157	18	5.06	1.02445887	1.05056649	1.03524734	0.98685453
5	406	44.1	9.47	1.34139926	1.09420523	1.12171015	0.87298793
4	295	34	5.81	0.89837752	1.00036055	0.92024534	1.05819895
5	91	10.3	4.67	0.89130283	0.90193871	0.92166558	1.07619417
8	141	15.1	7.18	1.1166331	1.04413874	1.02259454	0.9741142
6	209	24.1	9.09	1.6911586	0.97610181	1.05047354	0.78478401
6	557	63.3	8.54	1.13713482	0.80979891	1.03220926	1.09021493
5	447	49.3	9.42	0.87113201	0.87798701	0.84106808	1.13435711
8	643	67.4	5.4	0.93665821	0.92535613	1.01606283	1.01327472
6	124	13.5	6.89	1.00727083	1.09835523	1.05057318	0.96758043
9	411	48.2	9.5	1.01257111	0.80909769	0.95080504	1.0316461
4	77	8.7	4.88	1.03716909	1.12789131	1.19401052	0.86406092
5	61	7.1	8.97	1.22351434	1.08752769	1.34309185	0.7902734
4	242	28.4	5.3	1.00003845	0.78712682	1.02775773	0.98914798
4	58	6.6	3.56	1.40249995	1.27683197	1.20620835	0.80714582
8	282	31.6	5.08	0.97692685	0.93523666	0.89698157	1.14454768
8	448	50.1	6.2	1.003091	1.06972639	0.97796689	0.99124996

6	164	19.3	8.92	1.07097888	0.90627491	1.11778603	0.98240452
5	223	25.5	8.88	0.81467332	1.12770172	0.96203887	0.98394607
4	255	28.5	5.69	0.87552608	1.05888272	0.99776705	1.00694653
5	614	68.7	5.08	1.01234044	0.80708745	0.93364982	1.07333661
4	186	21.4	8.25	1.02934329	0.87033408	0.97751714	1.06335444
4	233	26	6.05	1.03214599	1.43008704	1.04831622	0.87166879
4	294	33.9	6.73	0.99740935	0.86896267	0.97819031	1.06541405
7	217	24.5	5.01	1.13767449	0.9906524	1.0822416	0.93793854
2	31	3.1	4.75	1.1445808	0.6685496	1.12232459	1.00933374
5	492	55.3	6.13	0.97141237	1.00046165	0.96546132	1.0082586
4	78	8.2	8.34	1.03612511	1.02994973	0.98088625	0.96729519
20	214	23.5	4.83	0.78037326	1.35769376	0.89713529	1.04389869
10	91	10.4	5.48	1.03720514	1.18626746	1.17090343	0.89050755
4	234	26	6.06	0.93686997	0.96219357	0.77879105	1.2479758
5	149	17.7	9.03	1.10251683	1.15338463	1.10662762	0.92656535
6	257	30.1	8.75	0.90029171	0.95540581	0.9541323	1.04302193
3	75	8.7	9	1.08034161	0.4387073	0.90722877	1.25163206
3	87	10.5	9.35	0.91329788	1.75622306	0.95538347	0.86017058
8	109	12.2	9.36	1.15274492	0.90069996	0.91161039	1.07195891
18	94	11.1	9.57	0.81577369	0.75464075	0.92423255	1.12021323
6	501	56.9	9.16	0.91357749	1.05921661	0.98390722	1.00515138
8	317	34.8	8	0.94672941	1.21758676	1.13705424	0.93611484
7	61	7.1	9.47	1.09182114	0.91245015	1.00657908	1.04758613
8	298	35	8.56	1.05816923	1.06738069	1.12379234	0.93017383
10	38	4.5	11.21	0.85265336	0.5423782	0.59878695	1.11298554
15	396	43.7	5.12	1.07373082	0.9542124	1.16167144	0.98517066
4	119	13.2	8.65	0.96239855	0.81037827	0.85296865	1.16731861
8	410	43.7	9.94	0.46653847	0.493856	0.54857694	1.32550171
3	184	20.5	9.7	0.88474466	1.28470023	0.96244607	0.97541089
7	132	15.1	7.43	0.9701224	0.90251331	0.82548731	1.20901065
12	60	6.9	9.99	0.79909792	1.17342599	2.12864574	0.5848615
6	345	35.6	6.55	0.84663018	0.66125133	0.76693794	1.1960327
4	157	17.7	4.89	1.01061561	0.94081805	1.01915827	0.99353642
5	268	31.3	7.02	0.84048006	0.79514373	0.87678046	1.16891841
16	46	5.4	9.52	0.98858483	1.26840833	1.13618186	0.91706492
5	67	7.9	8.69	0.72132608	3.06625849	0.94692947	0.61773208
6	531	62.1	4.94	0.893216	0.88473901	0.96301786	1.08789197
5	94	10.9	6.04	1.0026739	1.03123671	0.93091601	0.97360734
12	358	40.6	5.15	0.91465795	1.23824348	0.91263065	0.87860371
3	245	28	8.88	0.87710469	1.03545059	0.98021824	1.05256276
7	224	26	6.42	0.98851953	1.02446593	1.10847284	0.94123027
2	295	33.6	9.47	0.89949068	1.11411959	1.14475426	0.93732896
5	1188	137	5.26	1.24049685	1.70394971	0.97049364	0.86718825
9	143	17.2	4.98	1.14252789	1.203941	1.1322032	0.87251716
5	294	35	9.04	1.06654236	0.93711841	1.03828442	1.01707698
6	248	28.4	6.81	0.95337124	1.27000284	1.00947078	0.94877842
173	398	43.9	4.97	1.1005849	0.99810569	0.92293441	1.09514892
44	1379	150.8	9.58	1.12543196	1.32940611	1.29479363	0.811385
6	338	37.4	7.96	1.0319151	0.84440084	0.99072189	1.05719103
2	256	27.2	9.54	1.01513356	0.68229995	1.02105411	1.11005556

3	441	49.7	9.63	0.93009199	0.92302872	0.82491989	1.11726916
7	459	50.3	9.35	0.54721583	0.51070133	0.51291006	1.27867602
7	383	41	5.16	0.99424429	1.00660506	1.02009518	1.02174528
11	97	11.2	6.52	1.02656532	1.43528527	1.03161825	0.90811011
5	244	28.3	5	0.93126939	1.19595824	1.03603161	0.98160223
4	160	18.8	7.56	0.84312751	1.28368027	1.08759293	0.95650279
6	361	42.9	9.42	1.08027292	0.88857164	0.99050183	0.99675292
23	447	49.3	5	0.88742598	1.14275412	0.83660803	1.06690609
4	381	43	5.99	0.86473223	1.09744069	1.0190745	0.98422665
6	288	31.5	5.49	0.80228277	0.58330436	0.77498927	1.40181881
5	536	61.3	7.37	1.01148759	0.93251653	0.97735689	1.05666899
6	122	13.1	9.47	0.88462778	0.83113906	0.87812927	1.15694229
5	241	28.1	7.09	1.01184032	1.33645035	1.01136288	0.93390151
8	217	24	9.7	0.91874277	1.61749404	0.96793649	0.7675401
15	84	9	9.19	1.25315058	1.20324313	1.34271864	0.7734005
5	425	49.1	5.83	1.01605586	0.98046726	0.90105921	1.06218502
3	398	43.7	5.58	1.12369444	1.00457014	0.924067	1.09243662
3	178	20.3	5.2	0.85771785	1.56168169	1.08903474	0.83748551
4	228	25.3	8.59	0.92049129	1.21708306	1.04520392	0.94815045
5	315	35.4	5.64	1.10941801	1.11229759	1.09674292	0.94412663
4	711	81.7	9.35	1.33130556	1.68376614	1.32091669	0.63596924
4	181	20.8	5.58	1.06523085	0.99253873	1.28686025	0.81953161
2	164	18.9	5.3	1.24043941	0.90548118	1.1430744	0.94820269
3	99	12.4	4.48	0.98567959	0.89177292	1.02556417	1.02827123
5	91	10.7	5.06	0.96001558	1.17327943	0.96505936	0.95815171
4	218	25.5	5.49	0.92620471	1.33517962	0.97477215	0.8989939
4	150	17.3	5.36	1.03443269	1.0181725	1.00262019	1.00516259
4	221	24.6	5.67	1.21677623	1.30200062	1.07220329	0.8735163
4	355	38.9	9.26	0.83476838	0.36394066	0.66639302	1.26852025
5	68	7.9	9.04	1.30155137	1.08376136	1.23493	0.87828979
4	357	39.9	8.05	1.1298639	1.28711955	1.28941723	0.79404252
4	552	60.4	4.69	1.00844169	1.31007647	1.05506908	0.90769267
3	159	18.9	9.06	0.98864151	0.59534696	0.87662066	1.21807795
3	223	25.4	4.87	0.8593979	0.66493834	0.9411035	1.10282627
3	172	20.5	9.23	0.9766106	1.18361353	0.99858918	0.97282346
3	559	64.3	5	1.03022765	1.26524049	1.06528792	0.89916371
3	146	16.7	6.61	0.92309936	0.94449797	0.93133049	1.03377368
17	229	26.7	4.72	0.84006984	1.33904501	1.03556635	0.85742802
2	202	22.2	10.05	0.73992721	0.36827448	0.58766171	1.46498244
4	118	14.2	4.92	0.9511223	0.83108598	0.95403869	1.0068423
4	335	39.5	9.14	1.02507497	1.09710738	1.07658346	0.97494682
2	57	6.7	8.76	1.01975563	1.3254302	1.04262349	0.91473934
3	242	28	5.26	1.21095908	1.06498122	0.96985109	0.93753744
5	763	88	9.07	1.07457698	1.15494876	1.12557092	0.89826421
3	316	34.4	6.93	0.98866799	1.59735802	0.94476331	0.90067911
3	287	32.6	9.55	1.04093378	0.57845962	0.83633247	1.1868937
5	141	15.5	9.23	1.1520035	1.06475555	0.90960652	0.98984638
2	154	17.4	8.07	0.82285461	1.00934214	0.9338404	1.08283494
19	152	16.6	5.55	0.98313066	0.774629	1.01533281	1.02127775
7	47	5.4	9.55	1.20004167	1.66156664	1.21398096	0.77939827

3	192	22.8	5.31	0.84180313	1.01749192	0.95536003	1.02947507
5	212	24.4	9.06	1.05046996	1.19286038	1.08238666	0.88297383
6	91	10.8	9.85	0.89522313	0.96474615	1.15870499	0.99407085
5	138	16.5	7.33	0.95868129	1.0515392	0.9597789	1.001391
4	67	7.8	9.52	0.99901194	0.90021901	0.94972629	1.04044886
7	439	50	5.76	1.0949224	1.18277621	1.07731485	0.90970564
3	273	31.6	8.4	0.80199041	1.26599566	1.00591821	0.97303945
7	116	13.1	9.33	1.11399683	1.12750444	1.39955851	0.80176783
5	110	13.6	4.91	1.08684085	0.97479514	1.24993948	0.9154843
3	110	12.2	6.79	0.99534	1.04638342	1.08024866	0.98133342
3	314	35.7	9.1	1.01060175	1.19483374	1.21842183	0.83815189
4	234	27	7.55	0.96548144	1.16935787	1.03166986	0.96843289
5	246	28.1	6.23	1.10032594	0.9901719	1.10402353	0.95101071
2	139	16.2	6.14	0.80788129	1.53037368	1.12822779	0.86858562
4	598	68.3	9.44	0.98608771	1.45824113	1.06718745	0.91585541
3	204	23.6	9.28	0.71143487	0.87809985	0.82286534	1.10971396
3	462	49	9.44	1.09954345	0.72831367	1.00747082	1.03060125
2	197	22.4	8.57	0.98684046	0.81919867	1.02453265	1.09123229
7	239	28.3	4.59	0.95270013	1.02987056	0.8734119	1.04401472
5	279	31.6	9.52	0.93693613	0.72365211	0.94404462	1.11268357
4	55	6.6	8.51	1.04885829	1.10999814	1.07568582	0.94091855
3	165	19	7.33	0.94091865	0.91519015	0.91932665	1.08421583
3	591	67	9.03	1.13315517	1.67750139	1.24453114	0.73326708
4	308	35.3	5.41	1.12723261	1.19487668	1.0447985	0.93377521
5	104	11.7	10.33	0.93348213	0.77330959	0.77988572	1.07079502
9	215	24.8	5.82	0.625874	0.56531212	0.61670604	1.25087355
2	284	32.3	5.5	1.10165787	1.26655512	0.95400566	0.89014272
2	89	10.2	8.16	1.0211744	0.96670659	0.97596617	1.01207396
3	339	39	8.29	1.06195324	1.08418597	1.13264224	0.89458598
6	107	12.6	4.7	0.96691886	0.96550841	0.98502827	0.9770591
4	184	21.9	8.56	1.04317499	0.98864117	0.97987322	0.97869615
2	344	38.2	6.84	0.93310124	0.9661267	0.94924635	1.03502676
5	65	7.6	7.42	0.92063449	1.26148265	1.01465146	0.95844165
4	72	8.1	4.51	1.02373259	1.3057546	1.13764483	0.83629001
4	88	9.9	4.61	0.85094073	0.85933079	0.82773993	1.19256012
3	266	30.1	6.54	0.90603662	1.0603684	0.99397598	1.01889906
5	321	35.2	8.92	0.6436231	2.44335732	0.77315053	0.88152142
6	150	17.2	9.39	0.97735755	0.97062776	1.0582524	0.99210393
2	140	15.4	8.98	0.97651487	0.69579834	0.97438517	1.10409286
3	630	74.3	8.09	1.1067429	1.13369336	1.20188597	0.86744467
3	152	17.2	9.94	0.40862622	4.43733105	0.83851144	0.45461721
3	402	47	9.47	1.06019831	1.27244237	1.06114144	0.91574847
4	150	16.4	9.8	1.07987598	1.49644648	1.11503378	0.82988353
2	253	27.4	5.86	1.01711563	1.21594137	1.03778097	0.92217475
7	87	10	7.05	0.88384118	1.35231429	1.00304746	0.92606555
2	231	26.5	6.54	1.04989548	0.96308713	1.11042346	0.9982127
3	446	50.2	9.35	1.16550176	1.24233491	1.09068856	0.87646616
3	189	21.1	6.55	0.8644901	1.60220577	1.01314396	0.87617585
2	518	61	7.68	1.05295818	1.10103218	1.13899845	0.90386465
2	188	21.4	8.03	0.85710496	0.68902742	0.85850554	1.15533096

2	509	54.9	8.69	1.40373044	0.82209397	0.90939781	0.97724544
2	288	32	9.51	0.78100615	0.77063839	0.92534722	1.11822984
6	142	15.8	6.57	0.97822085	0.84797267	0.95844444	1.08620489
3	213	24.5	4.63	1.04398314	1.2563956	1.09675159	0.92521481
3	494	55.3	9.89	1.00981589	1.08332153	1.02900199	0.96943519
3	353	39.1	4.42	0.55857442	3.66304674	0.82701926	0.6095071
5	104	11.9	5.69	0.73662379	1.12533585	0.89670171	1.06461027
2	50	5.8	5.02	0.76075796	0.91157567	0.84972146	1.07608453
3	237	26.8	5.47	1.01797514	1.03332741	0.94598231	0.97998621
2	509	56.3	5.21	1.08019961	1.34247592	1.00943	0.92603253
3	202	22.7	9.61	0.9774673	1.30161966	1.09992822	0.90339391
4	138	16	5.06	1.05301325	1.10828196	1.20387771	0.91348799
4	58	7.1	9.13	0.98228443	0.84472579	1.00286579	1.05019323
13	166	20.1	4.3	1.0343777	0.86423329	0.95852431	0.99216189
2	317	37.9	5.38	0.989564	0.82264489	1.00215154	1.08264309
4	207	24.5	7.03	0.84680066	1.32522658	0.94043331	0.99998156
8	351	39.7	4.93	0.89441575	1.43523253	1.09862521	0.81281505
3	136	15.2	5.22	0.85882782	1.41029254	1.01448523	0.88464701
4	212	23.1	5.17	1.01718	1.07452133	1.04300459	0.95278221
3	437	46.3	8.98	1.05213166	0.93667926	1.03317044	1.00588609
3	482	51.8	9.36	1.29597588	0.8644445	1.15401208	0.93819486
3	263	30.7	6.54	1.31423583	0.91067985	0.92201493	1.11364369
4	138	16.4	5.63	0.95121434	1.19259782	1.00900829	0.97490377
10	65	7.5	4.36	1.33547793	0.84068027	0.91832818	1.0535895
5	254	27.9	8.73	0.984778	0.83810806	1.06787061	1.02095285
5	275	31.3	5.48	1.15132674	1.31763823	1.13517996	0.83814845
1	88	9.6	4.22	1.01853593	0.92482456	1.07103324	1.04209403
2	280	32.1	6.95	1.05980719	0.94145141	1.0490003	0.97799111
2	347	39.8	8.73	0.91762332	1.41662248	1.05197225	0.89637718
4	452	50.2	9.23	1.13243066	1.11659279	1.17533161	0.8842733
4	264	29.5	4.97	1.03565418	0.89673756	0.98889004	0.96692886
3	210	24.7	4.78	0.97659343	0.91752317	0.96908736	1.0484905
3	407	48.5	9.95	0.99441289	0.88143257	0.9059631	1.10462407
13	317	34.9	5.48	1.20071434	1.3152523	1.24357956	0.7911013
1	46	4.9	4.61	1.21048492	1.21067004	0.90117402	0.91952652
3	126	14.9	5.92	1.06105358	1.06965026	1.10297422	0.99318189
3	203	22.3	4.97	1.03356947	0.82965558	1.07125025	1.02290366
3	123	14.5	9.57	1.33256267	0.81533672	1.18499813	0.87340805
5	190	21.6	6.73	1.0218181	1.1746292	1.03234344	0.89257668
5	286	32.4	6.01	1.12389864	0.97851937	1.08598189	0.97834553
6	383	45.9	9.33	1.05941517	0.82445807	0.92113818	1.08543379
2	97	10.6	7.52	0.79606197	2.06050809	0.93361072	0.84792695
1	228	25	9.07	1.18991624	1.79412324	0.77830109	0.79039442
4	109	11.9	4.7	1.08497526	1.13796091	1.06892354	0.8583376
2	592	64.9	5.55	1.12005711	1.36345811	1.45026712	0.73303278
3	248	28.2	8.78	1.02647804	0.80861464	1.12273217	1.00016195
5	103	11.5	8.41	0.98281488	1.38918625	1.04449301	0.84715276
2	249	29.4	8.1	1.06919198	1.18692491	1.02516793	0.92136667
3	402	43.3	8.63	0.991471	1.21945636	1.02597771	0.9085075
3	517	61.2	4.86	0.91515435	1.26815533	0.96809312	0.93605795

1	100	11.4	5.45	1.09351682	1.08543194	1.23189511	0.86892683
4	172	19.2	5.19	1.667854	1.68356904	1.6807902	0.42682644
3	230	25.8	5.06	0.91391633	0.92819622	0.96160504	1.05722255
6	238	27.9	6.18	0.94850922	1.11993808	1.01112727	0.98294401
3	175	20.8	6.06	1.06600661	0.79299186	1.01480975	0.99080865
2	234	26.4	9.01	1.25948203	1.20426217	1.17404169	0.78067632
3	57	6.5	4.94	0.75552462	1.7874791	0.97904442	0.83380714
2	278	31.7	5.3	1.04451847	0.74089568	0.96769486	1.09570848
4	71	8	9.76	0.87004692	0.78844733	0.87748684	1.09490071
3	381	44	6.95	0.83205895	0.69280787	0.8107037	1.1836449
3	91	10.3	5.48	1.19901327	1.03103191	1.16526307	0.89918378
3	292	33.1	6.92	1.2351801	1.27486957	1.17551933	0.79700091
1	176	20.4	9.61	0.69099189	0.84379361	0.83708151	1.1241053
5	371	42.6	4.93	1.12117432	1.16568447	1.58483732	0.74495049
3	111	13	5.57	0.93345479	0.84675042	1.01743832	1.02698779
6	977	114.5	5.81	0.92950771	1.3517213	1.02319554	0.95180983
2	149	17.4	7.36	1.0663414	0.92582856	1.00366073	1.04061971
2	139	15.5	9.42	1.19859719	1.46148637	1.03088076	0.93416889
2	374	42.9	5.88	1.00762558	1.13332445	1.05065313	0.92093987
2	197	21.8	4.96	0.93093845	1.06674009	1.00072782	0.96663394
2	327	37.5	6.24	0.98639351	1.21284591	1.01504231	0.98523207
4	259	31.1	9.7	1.01887397	1.15823061	1.15795838	0.91608942
3	406	45.1	9.06	0.91778759	0.67566745	0.90339772	1.14825704
2	513	56	9.25	1.24859253	0.76681625	1.17254706	0.94185612
5	341	38.8	5.34	0.78920604	1.72264953	0.98848905	0.84690169
2	109	12.3	7.43	0.96918894	0.77435849	0.88784673	1.14878581
3	211	24.4	9.69	1.06797426	0.60073602	0.86493973	1.18733123
2	403	43.3	9.89	1.0945497	0.75984149	1.01548041	1.00966982
3	334	39.2	9.07	1.06312508	0.82504061	1.05449374	1.01645624
2	185	21	8.05	0.86794842	1.22835493	0.92722195	1.00154204
2	333	37.2	6.11	1.01676102	0.79546307	0.89530932	1.17234621
3	318	34.9	5.12	1.02547284	1.18623257	1.09463631	0.98337416
3	260	29.1	8.98	1.13039279	0.86733566	1.12015909	0.97541736
2	166	18.7	5.58	0.98483759	1.10410598	1.04931112	0.94214273
2	192	21.5	5.71	0.94422661	1.15666399	0.98986757	0.97830109
2	100	11.3	6.06	0.88086653	1.29962757	0.97835456	0.94492231
3	484	53.4	9.55	1.01623394	0.68549615	0.96230279	1.10193634
2	201	22.5	6.2	1.01901983	1.02539642	0.98608081	1.02793363
2	122	14.8	5.4	0.9375609	1.27010415	0.95972669	0.90789693
3	243	28	8.88	1.02000845	1.10198631	0.98543548	0.95674381
1	275	30.5	4.7	0.83433253	1.02861751	1.30749224	0.80782562
2	260	29.1	5.92	0.76562323	0.88187021	1.16801044	1.12165847
11	885	102	7.69	1.05528565	0.91180075	0.94672904	1.05519747
6	213	23.8	5.44	0.84775525	0.87493131	0.94685977	1.02360226
2	208	24.4	9.31	1.15493756	1.16893261	1.11113571	0.8846815
3	287	31.6	9.11	0.99034162	0.80699439	0.90413188	1.06724354
5	573	64.2	7.03	1.24483501	1.32389258	1.17398319	0.78721149
2	437	50.4	9.09	0.99685204	1.15558536	1.02281243	0.92718513
2	143	16.6	5.02	0.78436298	1.64156983	0.9932438	0.9105763
2	361	41.4	6.44	1.11470179	1.16605865	1.12373734	0.90961545

1	67	7.5	4.7	1.01019694	1.46015921	1.157095	0.7969898
2	84	9.6	5.97	0.96522754	1.58418812	1.17885111	0.75974972
6	451	48.8	5.78	0.98689198	0.91227459	0.97774498	1.02045491
12	166	20.1	4.36				
1	169	17.7	6.24	1.14892884	1.49047021	1.15351801	0.7803205
1	73	8.9	4.56	0.89187505	1.32749347	0.91782537	1.00265255
2	149	16.7	9.79	0.95637276	0.88893638	1.12427104	1.00871203
2	254	29.7	6.7	0.90231703	1.06445799	0.9798056	1.00645837
10	66	7.8	12.38	0.85659415	0.48164379	0.77413359	1.3013328
3	142	16	6.8	1.04396023	0.88036266	1.02880552	1.01714556
3	298	32.2	6.32	1.18186716	0.83861744	1.12488717	0.99644724
2	135	16	5.38	1.03790309	1.17518034	0.99730399	0.9644574
3	251	27.5	9.63	0.96391404	0.85393303	0.92089381	1.18173187
2	264	29.8	6.55	0.91597883	0.88607075	0.87975897	1.09843204
3	562	60	6.29	1.10610151	1.30024923	1.27335128	0.78893476
1	256	28.9	9.5	0.88320884	0.44122592	1.13321544	1.15429163
2	184	21.3	5.1	1.0387441	0.66618383	0.86016675	1.15141254
4	293	31.3	10.14	0.5712729	0.77549745	0.66711305	1.27413233
1	79	9	9.91	0.91584253	1.47683982	1.09360212	0.84006944
1	136	15.4	7.49	0.88256557	0.6338203	0.86425629	1.17162205
3	238	27.7	4.51	0.90477433	1.77285221	0.89561425	0.90716642
1	499	55.4	9.31	0.99164911	1.03747655	1.17597125	0.89335595
3	174	20.2	5.12	1.04916856	0.83228326	1.21346965	1.01577233
1	191	21.3	7.74	1.04742704	1.0604803	1.05955884	0.94756111
3	364	41	4.77	1.16303263	1.01654284	1.6235959	0.64756082
3	424	46.5	5.15	1.1158765	0.98484755	1.02605428	0.97003787
2	110	12.3	8.6	0.92439048	0.79663153	0.84471817	1.16285062
1	179	20.7	4.77	1.21779548	1.33000052	0.93363691	0.90323351
2	103	11.6	9.94	0.99624128	0.88989777	0.91602995	1.1017406
3	195	23.2	6.1	0.98301231	1.20316132	0.95782328	0.96195542
2	52	6	10.1	0.87259255	0.59915112	0.76586406	1.21489816
2	279	32.1	6.73	1.02478817	1.25362397	1.0764239	0.89967204
3	325	36	5.67	1.01657617	0.70796502	0.93907726	1.13007782
5	353	41.5	6.06	0.83652647	0.93196158	0.93252637	1.07298552
3	604	69.6	9.31	0.93256734	1.06888936	1.03154751	0.98493797
1	226	25.7	8.94	1.10260463	1.45070777	1.13580978	0.86070493
2	163	18.6	7.65	0.77663356	0.79105573	0.89503638	0.85389402
3	311	35.9	6.81	1.11247761	1.21782837	1.00099658	0.94128944
3	422	47.6	9.5	1.06315588	0.88035637	1.12852484	0.97547448
2	138	16.4	5.22	0.96624733	0.95035585	0.93502551	0.95708676
3	105	11.2	8.16	0.95121034	1.00141137	0.88888809	1.04714325
2	395	45.5	4.93	1.00677905	1.07823201	0.98321038	0.96505308
2	292	33.3	9.35	0.89713965	0.88608472	0.91369638	1.00763151
3	343	37	4.74	1.18253124	1.09305437	1.16987374	0.8846258
3	116	13.3	10.64	0.95413095	0.61126103	0.98886633	1.13664445
2	163	18.4	7.49	1.01855485	0.83988047	0.95675258	1.05552292
1	59	6.7	9.11	1.04375061	1.15110087	1.11545062	0.87261496
1	120	13.7	5.97	1.04861205	0.5401557	0.88417047	1.24690265
1	185	21	9.29	1.00579847	1.16452394	1.12515707	0.91096923
1	69	8.6	9.63	0.79705538	1.06605077	0.92657112	1.02696618

1	249	29.9	9.73	1.34402603	0.83157085	1.0385117	0.93384877
2	167	18.1	10.02	0.83824931	0.79004204	0.9417031	1.12197397
4	162	18.1	4.75	0.95485131	1.45543837	1.09349876	0.88984572
2	306	35.4	9.06	0.96844935	1.16859771	0.97611423	0.96844716
4	145	17	5.12	1.0241215	1.54385034	1.04280233	0.84485005
1	319	36.2	6.6	1.13676994	1.25175416	1.21774733	0.80779329
1	148	15.5	4.55	0.91447833	1.20920905	1.43151475	0.85011268
1	680	76.4	8.53	0.9065113	0.88347124	1.06097314	0.99896537
13	220	23.7	9.86	1.09177409	1.006002	0.98652737	1.00260529
1	48	5.8	4.22	1.12026511	1.26921619	0.89402634	0.92968856
1	143	16.9	10.23	0.9857528	0.49572487	0.85250382	1.27125715
2	172	17.9	5.06	0.81141563	1.0072322	0.87559977	1.06589815
1	284	32.3	5.22	0.89954378	0.9192224	0.96213309	1.0163497
5	293	31.1	4.81	1.06508151	1.20294245	1.08705831	0.86713512
2	227	25.7	9.35	0.83473298	0.50801243	0.77917235	1.23946438
3	356	40.2	9.13	1.19734355	0.99462786	1.12310729	0.92078883
3	483	57.7	7.08	0.99020369	0.89138954	1.02181106	0.97493334
2	94	10.4	5.19	0.95071303	0.64629822	0.88170171	1.1226866
3	193	22.1	6.68	1.00639393	0.87571771	0.93369944	1.05953677
2	391	45.9	9.44	1.01610254	1.3407388	1.13951992	0.85127398
1	455	55.1	8.78	0.81787738	1.86847738	1.24847835	0.74284401
3	104	12.3	8.81	1.44265262	1.14345023	1.31158881	0.75350725
1	342	38.6	5.53	0.81388761	1.31330405	0.9771434	0.86731935
5	1572	180.3	5.63	1.28539266	1.23802624	1.22113669	0.76056199
5	325	36.8	9.39	0.95632855	1.04935988	0.95674864	1.02465483
1	55	6.5	5.24	1.0466521	0.7543661	0.89583056	1.07805294
1	152	18	9.14	0.87631045	1.10292198	1.02929055	0.97688945
1	134	15.3	9.26	0.67252938	0.64766162	0.7867927	1.17349815
1	109	11.8	6.52	0.913357	1.96864347	1.06707286	0.74230814
2	278	30.7	8	0.90914619	1.12536399	1.05123041	0.96238165
2	420	48.5	8.9	0.79008922	1.45107537	0.93975287	0.96389004
1	468	54.1	6.34	0.8255669	1.32196633	0.94244355	1.01252887
44	166	19.6	4.7	1.32856595	1.05814898	1.27830898	0.86275628
3	89	10.6	10.76	0.92546985	1.06515064	0.95762678	0.9839393
2	111	13.5	4.83	0.97796811	1.06731308	0.99895135	0.99721169
2	177	20.5	9.45	0.91664493	1.07831066	0.98789249	0.97496763
2	326	36.6	5.07	1.18303277	0.95812502	1.13843961	0.91864574
3	280	30	5.73	0.74995725	0.93693863	0.91157869	1.22417017
1	268	30.9	9.83	0.85973578	1.01420898	1.20531612	0.92301358
1	133	14.6	5.48	0.95868741	1.57388314	1.078701	0.81905643
1	78	9.3	9.94	1.11667468	0.7252749	1.01830483	1.0798367
1	227	26.2	9.88	0.9649115	1.08994949	1.05681831	0.95215285
2	112	13.1	6.8	0.93709686	1.1438609	1.14675571	0.93310054
3	43	4.8	7.53	0.97656936	1.23046847	1.1815131	0.85731132
6	307	33.4	5.64	1.31407611	1.2919047	1.18512981	0.78402439
1	308	35.5	5.74	1.03490018	0.99543264	1.09803849	0.98026071
1	322	34.4	9.77	0.75544651	0.83746368	0.95103001	1.14337728
2	263	29.3	4.98	0.9535497	0.78084871	0.97465999	1.0495805
2	58	6.7	4.65	0.99556059	1.21027771	0.97990643	0.88701816
2	159	18.3	8.85	0.89087131	0.87827495	0.93390621	1.11495519

1	56	6.3	5.19	1.44637801	1.63870912	1.08205404	0.70012135
4	80	9.2	4.7	0.87726874	1.02644425	0.93533682	0.99725844
2	174	20.1	9.83	1.0600046	0.88314791	0.8851261	1.05368544
3	157	17.7	8.94	1.34917877	1.09715068	1.29281296	0.76313527
4	425	46.1	8.97	0.87528982	0.71322005	0.80589509	1.18026622
2	119	14.2	8.37	1.0647286	1.28704112	1.12321028	0.87096541
3	336	36.4	4.89	0.89960151	1.03578094	1.05754905	1.01632345
3	138	15.9	8.88	0.92912813	1.14720632	1.09057634	1.05061727
2	83	9.4	4.75	1.21147742	1.97443148	1.40453255	0.57085101
2	166	19.7	9.54	0.87242179	2.05244386	0.9229028	0.84607982
1	592	68.9	5.4	0.98590255	1.05101038	0.96367899	1.01296659
1	155	17.9	9.86	0.76358285	0.93575915	0.84437135	1.09753298
1	65	7.7	9.31	1.26879589	1.11141989	1.16205749	0.91873831
2	651	74.5	6.57	0.8449875	1.38136766	0.99380081	0.9370883
1	404	44.1	5.2	0.88286016	1.24406695	1.04709317	0.95612192
2	78	9.1	9.2	1.10134124	1.04850974	0.97830321	0.96684292
5	43	5	10.24	1.05142218	0.82813127	1.01135568	1.08795162
2	2276	238.4	4.11	0.7905682	0.9989906	0.93342017	1.04362379
2	428	47.5	5.69	1.31432192	1.07728536	1.22540012	0.84457115
1	187	22.3	4.58	1.10608224	0.71700556	0.90978704	1.07598762
1	345	40.6	6.8	0.87664677	1.48148819	1.0648708	0.87952093
3	331	35.7	9.85	0.9246368	0.52937374	0.82596735	1.12215808
1	216	23.9	8.91	0.9979198	1.00583462	1.06741362	0.98070005
6	135	15.4	4.83	0.91917652	0.99828664	0.95631867	1.07385492
2	219	25.5	4.87	1.19098457	0.76582602	1.03479902	1.0656259
1	148	17.2	6.54	1.02276396	1.16620245	1.07433044	0.97040347
2	354	38.8	5.39	0.85510475	1.49925585	1.06181385	0.8733353
1	351	39.5	9.09	0.9757896	1.23988361	1.15424879	0.87900953
2	61	6.7	9.54	0.76410341	1.08195124	0.85718683	1.11745512
8	127	13.9	4.56	1.41742286	1.26658036	1.31543075	0.74268998
1	77	8.8	6.25	0.81962567	1.4205591	0.90233482	0.95088496
5	1200	131.5	4.79	0.93325524	1.34189236	1.44419968	0.88825998
2	238	26.9	9.51	0.74320409	1.48779726	1.07014441	0.99765842
1	86	9.8	5.19	1.00478106	1.00654449	1.03501796	0.94779609
2	79	8.4	4.88	0.99483093	1.00011012	0.93694446	1.00758774
1	378	42.5	5.14	1.20523439	0.67625839	0.97968209	1.07489371
2	253	28.8	6.95	1.01511947	0.93346507	0.95145679	1.03405975
1	115	13.5	9.09	1.14816533	1.32021764	1.28570751	0.8053771
2	99	11	5	0.98795113	1.06542898	0.82988974	1.0998548
3	960	110.7	6.15	1.24177909	0.93065203	1.20717927	0.89086847
14	144	16.2	4.7	0.77085009	1.48611498	1.03241879	1.00881458
1	160	18.9	9.03	0.83070133	0.90833569	0.98875879	1.06160635
2	451	49.7	9.55	1.09289945	0.65022414	0.92012714	1.07811425
1	56	6.4	4.34	1.54944322	0.88052448	0.69205986	1.10100467
2	107	12.5	5.19	1.10774822	1.04350269	1.08597965	0.93544749
2	342	37.3	10.17	0.88585402	0.59157844	0.94217592	1.16315371
1	277	29.9	8.66	0.88008025	1.19498346	1.07036514	0.90689477
4	243	27.4	5.44	1.11002933	0.99008695	0.96110043	0.9986021
1	85	10	9.91	0.95463476	1.57648734	1.1560159	0.80743376
1	79	9	5.22	1.05926657	1.25294851	1.09960229	0.92437792

1	166	19.1	10.18	0.93102458	0.63766397	0.88793804	1.18454632
1	323	35.9	5.11	0.86223168	0.95796578	0.97930126	1.01805142
3	449	52.7	9.6	1.0047928	0.91262986	0.96007845	1.05206921
4	72	8.5	9.32	0.809376	1.76883317	1.06747187	0.78117853
1	106	12.5	8.84	0.81197581	0.92065357	0.84861123	1.11567116
1	106	12.7	5.22	0.90995121	0.88224438	0.88794529	1.06864488
1	578	65.9	6.01	0.87207194	1.03072491	1.03191918	1.01272341
1	116	13.9	9.67	1.07853539	1.08736764	0.90415631	1.03080422
1	209	23.3	9.33	0.87468124	0.52996462	0.90728111	1.19517091
2	231	24.9	9	1.11589626	0.94177996	1.23502692	0.90179269
13	70	7	6.46	1.1778795	0.96644436	1.12831852	0.97380302
1	44	5	8.92	1.30594421	1.09651696	1.08710105	0.91641812
2	267	29.7	6.8	0.95580623	0.82191324	1.00021714	1.04211597
2	179	20.7	8.56	0.83053859	0.90935719	0.91757488	1.08809599
1	225	26.1	4.82	1.76931588	0.80476297	1.19673736	0.42911754
1	341	38.1	6.11	0.94957958	1.054417	0.79561425	1.07439273
1	452	53.3	8.63	1.23283674	0.97818332	1.10068986	0.95324766
1	148	17.5	6.93	1.04137332	0.8725536	1.01377674	1.05025008
1	400	44.6	9.03	0.96699612	0.94076797	0.99485042	1.02611052
1	382	42.6	5.24	0.89744473	1.10617897	0.96276073	0.96408002
1	219	25.2	6.95	1.40008017	0.92787955	1.17773572	0.85908266
2	154	16.4	10.4	1.09608466	0.75178905	0.99902281	1.09366208
1	319	35.2	5.1	1.11380734	1.15579789	1.02542695	0.88837115
2	422	44.8	9.13	1.15424183	1.42314907	1.22198205	0.80714393
1	92	10.4	10.4	0.68636898	0.67396758	0.96184878	0.9906734
1	351	38.6	9.22	1.08822086	0.81465856	0.836371	1.07245154
1	326	36.9	7.77	1.11329504	0.70429387	1.05331562	1.02999316
12	61	7.3	10.33	0.83018933	0.88393173	0.90685133	1.09066246
2	362	39.5	8.13	2.08251646	0.81088903	0.78956315	0.81528997
1	102	11.3	6.54	1.68485063	1.71201962	1.07569275	0.53301075
1	294	35.3	8.88	1.11016015	0.6268498	0.91174983	1.14267175
1	192	21.5	5.81	0.77396768	0.88522039	0.88136286	1.05353456
1	194	22.8	9.09	0.85580305	0.66964267	0.82711381	1.13539239
1	305	33.9	8.47	0.78120397	0.74542671	0.90287417	1.0962745
1	226	25.4	8.78	0.92368908	0.89923719	0.8524048	1.14435748
1	335	38.7	9.42	1.36742063	1.01664744	1.38697202	0.76462602
1	188	21.4	5.64	0.9224686	1.3411372	1.0392429	0.88461532
1	168	19.9	8.65	0.86218359	0.9656744	0.98458372	1.01606624
2	148	16.1	7.46	1.05071682	0.79262978	0.95427699	1.07725749
1	46	5.3	8.02	1.15620752	1.37095759	0.93198459	0.90130341
3	151	17.4	9.16	0.68134957	1.40064223	0.79333777	1.09469571
3	353	37.3	9.32	0.89806424	0.62910993	0.87902198	1.13866882
1	113	13.4	4.41	0.9117807	1.65039562	0.99538984	0.8567234
1	97	10.5	4.88	0.94740913	1.70422828	1.23485605	0.69403507
1	223	24.6	5.11	0.89970789	1.25594651	1.13500988	0.86158663
1	279	30.2	9.99	1.04141762	0.99729522	0.99706296	1.00814479
1	99	11.1	8.41	1.04929916	0.95483489	1.04187598	0.9718864
1	288	31.6	6.37	0.81335427	2.059526	1.03910412	0.74499805
2	106	11.4	5.01	1.13546273	0.95259131	1.06900393	0.98647262
7	34	4	9.52	0.85520759	1.07106912	0.92299659	1.01710949

1	128	14.8	9.41	1.00228327	0.87026352	0.92849323	1.09185877
1	256	29.7	6.23	0.85555045	1.14097745	0.96996535	0.99805873
13	199	22.3	5.06	1.04415811	0.8921601	1.10447205	0.97001784
1	292	32	5.08	1.0614366	1.48674432	1.00751555	0.85656064
1	79	8.5	9.01	0.9439704	0.94674343	0.92468672	0.96990196
1	452	49.9	9.2	0.93695157	1.44435539	1.0002481	0.89399991
1	420	48.1	5.14	0.98593002	1.59398858	1.2024153	0.79214661
1	747	85.8	6.35	0.9105667	1.47881888	1.10213342	0.84202067
1	120	13.1	9.48	0.9901099	0.99070501	1.13951474	0.94571531
1	337	38.2	5.68	0.97645689	1.09139207	0.93497001	0.97162773
1	780	89.8	5.03	1.08843815	1.45650816	1.07591439	0.81731368
1	566	66.4	5.29	1.14935044	0.86577691	0.97885413	1.02872886
2	301	33.5	9.79	0.87789684	0.97859196	0.91698266	1.06867239
4	72	8.3	4.61	0.91080257	1.3013343	1.02011448	0.90717523
1	334	35.2	10.15	1.00761532	0.70159986	0.92585803	1.09495259
1	68	7.9	9.54	0.92244836	0.79811434	0.94407947	1.10017232
1	67	7.8	4.22	0.89486684	1.84269896	0.92707115	0.72581886
1	371	44	9.38	1.09677346	0.92708413	0.92947697	0.98086114
1	86	9.9	5.92	0.9809628	0.82459935	0.78817312	1.13341657
1	101	11.1	11.59	1.01189478	1.11782134	1.00602849	1.02239961
2	166	18.8	5.12	1.05762975	0.98237079	1.1160446	0.95021183
1	126	14.1	4.02	1.01258684	1.07839164	0.92614476	1.11635297
2	336	35.4	5.52	1.07712871	1.28595616	1.02999894	0.95002889
1	73	8.9	9.6	0.92041796	1.61572521	0.92584451	0.81248923
1	114	12.5	5.29	0.97236135	0.92258261	1.00869459	0.98594379
3	132	13.9	6.67	0.81835019	1.0454022	0.92644039	0.93704794
2	382	45.7	9.39	1.38955204	2.00110869	0.95770304	0.7043336
2	828	95.1	7.28	1.03566869	1.15387124	1.07871505	0.99042513
2	335	35.8	5.47	1.03559689	0.93689336	1.05448338	0.99686743
1	147	16.3	4.92	1.41886282	0.86151671	1.35893545	0.8420302
1	261	30.6	6.54	0.76162634	1.40136001	1.01385346	1.13690757
1	51	5.9	8.47	1.01387958	1.18445149	0.87181506	0.93994942
1	301	32.6	9.58	1.25486228	0.900653	1.36934532	0.82517965
1	192	21.9	9.23	1.04492861	1.0270203	1.01238538	0.96000729
3	464	52.3	7.14	1.1711151	1.67248699	1.49099422	0.67602831
1	319	35.6	9.32	0.89139609	0.78410214	0.82139656	1.12784194
1	132	14.7	9.58	0.64176719	0.72199804	0.73354846	1.28636316
1	128	12.4	12.25	0.53506346	3.05598341	0.90518738	0.50168596
1	147	15.8	9.33	0.66263354	1.69995241	2.01759072	0.50290212
1	146	15.9	8.97	1.01583335	1.10371569	1.00602225	0.99806806
1	403	46.9	8.69	1.04295131	0.97080746	1.07143209	0.93910479
1	506	56.2	5.02	0.99331373	0.86991812	0.88636032	1.04293085
3	210	22.2	4.65	1.10220691	0.96791268	1.02228622	0.98243593
1	438	47.3	6.68	0.95986847	0.75591789	1.01174665	1.06732738
1	259	28.9	9.07	1.08975148	1.17874046	1.15265187	0.88890163
1	238	27.9	9.32	1.01582556	0.80348637	1.04231485	1.00147841
1	88	10.7	9.66	1.06902776	0.72354602	1.01910411	1.03756063
4	234	26.7	6.46	0.95384572	1.14549198	0.90311217	0.99849459
1	325	36.1	6.74	0.95180813	0.962347	0.99764692	1.09655024
1	119	13.6	9.31	0.74749621	0.53273618	0.88169909	1.23842095

1	99	12.3	9.31	0.77043557	1.85679192	1.07898938	0.84125992
1	153	15.5	12	1.12446699	1.19802117	1.07757314	0.95306261
1	359	42.6	8.62	1.12321591	0.86276619	1.14810757	1.00069374
1	238	27.5	5.17	1.09228813	1.33870731	1.034668	0.87864837
1	255	29.3	7.01	1.01496367	1.21767794	0.94072963	0.94995708
1	95	10.8	9.76	1.0897756	1.01839148	1.04824645	0.99500688
1	456	53.6	7.2	0.80974496	0.96504864	0.96780051	1.08506954
1	157	18.1	7.9	0.8536868	0.60626949	0.968346	1.16814336
1	171	19.6	5.35	1.20379132	0.84376622	1.11329367	0.99159162
6	44	4.8	8.84	0.90157889	1.20391414	1.0729601	0.94963802
1	170	17.4	11.69	0.87111405	1.27877359	0.97066706	0.92603742
1	231	25	9.74	1.10965792	0.96815653	1.00443465	0.98961705
2	230	27.1	4.83	0.89389121	1.23509771	0.98949431	0.96984021
1	67	8	9.16	0.98699307	1.24361949	1.19884583	0.87457018
1	338	41	9.11	0.78465919	1.42976946	1.25807506	0.87971345
7	1204	140.5	7.77	0.94948467	1.03146075	0.86638893	1.04479082
1	98	10.7	8.16	0.80220334	0.74104094	0.72340702	1.24262684
1	185	21.2	9.04	1.14094588	1.48097182	1.20096336	0.74254343
1	147	15.2	8.57	1.03161499	1.38351883	1.102891	0.85114194
1	176	20.8	9.26	0.96556218	1.19968988	0.81964291	1.14053142
1	316	34.5	7.81	1.09519718	0.75638751	1.06027784	1.00755558
1	281	31.7	5.44	0.80214087	1.1241178	0.91222787	1.03743522
1	666	76.8	9.82	1.32236731	0.40956917	0.80844916	1.22905294
1	546	60.6	4.93	1.13519273	1.11311576	1.23080063	0.86187043
1	105	12.4	5.06	0.90557446	1.14539729	0.9282631	1.02059129
1	84	8.9	11.53	0.99959666	1.31232588	0.95901266	0.9303321
1	2459	260.5	4.74	1.19811934	1.31564419	1.29472949	0.76502531
1	271	31.5	7.36	1.02585122	0.82571258	1.00487217	1.07040906
1	97	10.5	5.36	0.82321746	1.60122447	1.01319711	0.82044468
2	33	3.8	10.18	0.8122214	1.7167477	1.17414922	0.79443308
1	138	15.9	6.92	0.97992442	1.67131898	0.98391027	0.84762371
1	127	13.4	11.43	0.90415891	0.81993973	0.91158268	1.07829524
1	369	41.8	6.71	0.92548026	1.12945486	1.05401179	0.97669127
2	183	20.1	9.82	1.43305562	0.99347161	0.97644267	0.93093096
2	254	27.6	6.96	1.11192552	1.16581254	1.24738476	0.81959763
1	444	47.4	8.48	1.21573685	0.73006697	1.1478954	0.98849595
3	211	22.5	8.88	0.91554566	1.0877517	0.94062628	1.00772864
3	185	21.3	6.58	0.93047726	1.11848532	1.06400987	0.93639204
45	458	51.2	7.12	0.88490231	1.24719252	0.98473404	0.96125557
1	455	53.1	9.47	0.65781207	1.37209946	0.84674912	1.13349892
2	166	17.5	5.12	0.89803176	1.05777848	0.94705908	1.01841612
2	354	40.9	9.32	0.63330192	0.81632121	0.82864134	1.24577947
2	242	28.3	7.56	0.63007709	4.3521984	1.23833503	0.33399866
2	310	33.7	4.93	1.03820682	1.05554565	1.0877052	0.93375251
1	118	13	8.51	0.8744162	0.63895282	0.84507985	1.15074872
1	356	40.3	8.9	0.74169438	1.78572328	0.9983418	0.90839522
1	273	31.6	6.81	0.95398501	1.3776767	1.10310236	0.87884376
2	1458	156.6	9.92	0.8559796	1.95015435	0.89479488	0.91489102
2	72	8.4	8.92	0.84272337	0.98143138	0.86384339	1.08653885
1	409	46	8.94	0.86904021	1.08257499	1.13833695	1.00310763

1	284	32.9	9.1	0.7076625	0.97131625	0.70121757	1.09407356
1	453	49.4	4.79	0.9759606	0.93393746	0.87956636	0.99580313
1	351	40.3	6.64	0.91606639	1.34092894	1.09592341	0.91076592
1	256	29.7	4.92	0.92244234	1.26713587	0.98283148	1.00437264
2	343	41	8.31	0.95811241	0.97031058	0.99159315	0.97679457
1	487	56.9	5.81	0.97518981	1.06646294	0.98201266	0.97450874
1	133	15.9	5.63	0.8637934	1.10823118	0.9709831	0.875071
1	306	34.1	9.17	1.1672155	1.07178466	1.22537327	0.87747729
1	122	12.4	4.49	1.15395996	0.92484964	0.86968803	1.04261739
1	432	46.7	5.17	1.17349091	1.28199436	1.29720782	0.8085907
1	141	14.7	10.67	1.25546453	1.03195939	1.18007037	0.90811781
1	281	33.7	9.04	1.9422775	0.51357147	1.09034329	0.87499265
1	391	42.6	5.64	1.04525405	1.45579004	1.08606979	0.88672463
1	394	43	7.09	1.03189408	0.93835887	1.06508588	0.98526655
1	234	26.5	7.93	1.0571419	0.92269835	0.98934836	0.97247253
1	193	23.7	5.57	1.42925929	0.72785798	1.04281477	0.9948296
1	95	10.7	4.79	1.07744665	0.91925343	1.15055902	0.94558578
1	132	13.9	12	1.01690591	0.70741592	0.89933309	1.16850496
1	475	56.7	7.2	0.83397873	1.12490184	1.02267465	0.99389866
1	50	5.8	9.19				
1	70	8.4	4.92	0.83026437	1.13419845	1.1376764	0.76817913
1	201	23.5	7.24	0.91203272	1.47551282	1.11954979	0.86807131
1	517	58.1	9.23	0.80835877	0.64148253	0.73929159	1.25748017
1	122	14.3	9.23	0.95425955	1.29136608	1.04760689	0.98736117
1	149	17	5.92	0.82377204	0.73677694	1.12669237	1.06726901
1	807	92	7.65	0.84659431	1.59924369	1.16667598	0.75152365
1	269	29.5	5.92	1.01726623	1.34284177	1.10859908	0.819987
1	375	45.2	9.25	0.91853795	1.7327232	0.90527616	0.85592071
1	193	21.2	5.48	0.97651644	0.84220047	0.92226932	1.12773518
1	416	46.1	5.21	0.79757826	1.45133932	1.03129865	0.90358241
1	100	11.6	10.21	0.87154065	0.8426588	0.97190311	1.10816669
1	620	72.4	6.23	0.99781351	0.82295468	0.89897572	1.0874549
1	114	13.4	10.35	0.81718376	1.40497577	1.01992352	0.98203037
1	476	51.7	9.13	1.11740963	1.63397776	1.08474177	0.81611332
1	412	47.2	5.92	0.79133502	0.87430208	0.72621454	1.09103736
1	222	26.1	4.97	1.49188248	1.01873213	0.99734913	0.90813086
1	345	37.2	5.78	0.59713266	1.22642024	0.88582804	0.58434746
2	209	23.9	6.55	1.05084894	0.88054538	0.94775986	1.03493362
1	143	16.1	5.19	0.8644284	1.41889707	1.12513281	0.86164376
2	501	57.8	5.4	0.99824928	1.12516146	1.10439602	0.90190256
1	475	53.6	8.25	0.97922752	0.95280184	0.99647691	0.99646957
1	441	49.3	5.34	0.95350067	1.01781694	1.00530112	1.0191731
1	88	10.5	9.6	1.02393695	1.10698711	1.11324079	0.91034793
1	450	49.4	9.09	0.72921789	0.78631742	0.71521251	1.22473667
1	145	16.3	9.7	1.04489002	0.60778251	0.98275269	1.0931826
1	815	93.3	5.36	0.81513711	1.56642746	1.10553001	0.83835313
1	862	98.8	5.62	1.36302735	1.17360812	1.19151931	0.78840686
2	502	58.3	8.22	0.92045826	0.87275897	0.91715615	1.01776745
1	352	39.9	5.17	1.02186855	1.10446271	1.00269275	1.01741997
1	148	15.7	4.58	0.82954138	1.25005429	1.44213082	0.84791626

1	156	17.4	4.87	0.91097115	1.18476121	0.99768741	0.93426455
1	159	16.8	5.02	1.03709982	1.44396121	1.11035239	0.79660977
1	367	41	5.3	0.86029167	1.19600028	1.00902642	0.79596423
1	104	11.5	5.2	1.22475367	1.25491343	1.14529415	0.87223379
1	522	60.1	8.91	0.47839286	0.46440031	0.37569481	1.39957963
1	430	46.9	4.72	1.21056279	1.20056602	1.12618333	0.81834628
1	304	32.6	5.33	0.76895864	0.7767732	0.7992846	1.22220314
1	1154	125.7	9.91	0.82203874	0.9155001	0.83372203	1.19416757
1	235	27.2	7.9	0.77570176	0.67054217	0.74618233	1.16945086
1	123	14.3	4.86	0.94307412	1.03948283	0.91998458	1.02999139
1	331	37.1	7.47	1.20133676	1.23134158	1.1071949	0.79898782
1	310	33.8	5.24	1.04821741	1.06084853	0.98832346	1.01686252
1	608	71.1	6.02	1.0068744	1.0569518	1.0745423	0.91255785
1	864	96.1	6.58	0.81122934	1.99646986	1.04604364	0.8318072
1	570	62.4	7.8	1.22376591	0.82716037	1.07130668	0.9534784
1	1499	166.3	6.02	0.91540353	0.85023237	0.90817471	1.0173041
1	391	45.7	9.44	0.98749071	1.13828692	1.07770202	0.93393326
1	404	47.7	9.69	0.70767873	0.95224038	0.85171173	1.07194103
1	89	10	6.8	0.86067121	1.25075961	1.17216137	0.86350479
1	378	44.1	9.48	0.9270899	2.00676762	1.21541398	0.69632568
1	400	45.7	6.32	0.81632926	3.06314904	0.69045633	0.79596174
1	66	7.3	6.51	0.83898316	1.10286952	0.95534142	1.03387163
1	86	10.1	7.37	1.38975649	1.11309253	1.36459442	0.71122027
1	91	10.8	5.24	0.98558298	0.85883939	0.85380662	1.08155357
1	426	49.8	5.26	1.15070104	1.28358303	1.18948224	0.83918469
1	388	44.4	9.25	0.97326227	0.84872838	0.88876977	1.08070344
1	70	7.6	4.48	1.03578303	1.27455709	1.04462685	0.92661322
1	819	91.8	7.74	1.47139083	1.43588213	1.10304035	0.68191954
1	444	49.3	6.02	0.91637354	1.40286994	1.21673144	0.76653504
1	1509	165.4	9.61	0.85738594	1.49077438	0.98783016	0.87808486
1	109	13.1	6.52	1.03800293	1.09189333	1.05255969	0.96616764
1	187	22.4	5.17	1.01955237	1.1774173	1.07517413	0.90024167
1	459	52.7	6.95	1.05054116	0.74309669	1.04345176	1.06585014

Treat-02	Treat-03	Identified or Quantified
0.75182249	0.6828552	Quantified
0.7255034	0.57544947	Quantified
1.08495373	1.09832755	Quantified
0.81207051	0.90973759	Quantified
1.00992739	0.96147494	Quantified
0.72174092	0.64921453	Quantified
0.91212773	0.7563195	Quantified
0.76213995	0.62222217	Quantified
0.74810858	0.75737126	Quantified
0.97164291	1.00033886	Quantified
1.00053511	1.01319865	Quantified
0.86367685	0.82293087	Quantified
1.33638245	1.24068731	Quantified
1.08384714	1.0929393	Quantified
1.00725268	0.98171213	Quantified
0.83490095	0.81238793	Quantified
0.68059744	0.58588085	Quantified
1.05957535	1.03914951	Quantified
1.16560924	1.13567509	Quantified
1.14805102	1.0991005	Quantified
1.11620736	1.07393997	Quantified
0.90950941	0.87893362	Quantified
1.0169992	0.9970211	Quantified
1.07221738	1.08445723	Quantified
1.25960534	1.20690491	Quantified
1.00572943	1.01395479	Quantified
1.06033218	1.02119522	Quantified
1.18457065	1.19496107	Quantified
1.21450739	1.21537281	Quantified
1.04399248	1.05790553	Quantified
0.79338065	0.80628353	Quantified
0.99262172	0.96751113	Quantified
0.95895739	0.9330886	Quantified
1.12888439	1.08528599	Quantified
1.03572513	1.11442226	Quantified
0.98637497	0.97199491	Quantified
1.05774326	1.03818571	Quantified
0.59516788	0.58172653	Quantified
0.96755015	0.95876298	Quantified
1.03439554	1.01160245	Quantified
0.87094614	0.82394344	Quantified
0.96029054	0.92836739	Quantified
1.05708778	1.00826977	Quantified
0.93449851	0.94655056	Quantified
0.73540317	0.73860606	Quantified
1.12973464	1.14082229	Quantified
0.92313067	0.85862894	Quantified

0.90454086	0.91253201	Quantified
1.03051883	1.00243558	Quantified
1.01373235	0.99173885	Quantified
0.72201719	0.72241366	Quantified
0.82111119	0.93703226	Quantified
0.73556155	0.68902157	Quantified
1.30242541	1.32143457	Quantified
1.09970503	1.05130785	Quantified
1.03148522	1.06131299	Quantified
0.66558097	0.6244262	Quantified
0.97144535	0.99756755	Quantified
1.04424298	1.04245631	Quantified
0.98444054	0.96327758	Quantified
0.91071718	0.85053312	Quantified
0.83993082	0.76559162	Quantified
1.09314112	1.01159779	Quantified
0.83013951	0.81761215	Quantified
0.90007695	0.87474125	Quantified
1.01221689	1.00982642	Quantified
0.5231706	0.54695193	Quantified
0.95662554	0.95380844	Quantified
1.07704503	1.05735188	Quantified
1.02057967	0.9844649	Quantified
0.86574667	0.89465103	Quantified
1.01274724	0.97582653	Quantified
1.04842037	1.03210779	Quantified
0.90474627	0.90409635	Quantified
1.02688026	0.96875529	Quantified
1.06648317	0.9900583	Quantified
0.93044129	0.93954252	Quantified
1.02016548	1.01073949	Quantified
0.98162378	1.00012852	Quantified
0.49354207	0.49451511	Quantified
1.05690892	0.95968392	Quantified
1.19356704	1.1259541	Quantified
1.12057465	1.04585841	Quantified
0.88670772	0.94862388	Quantified
1.20907088	1.10752287	Quantified
1.27947235	1.28445404	Quantified
1.07890004	1.04997216	Quantified
1.05688761	1.02422181	Quantified
1.10045681	1.09239253	Quantified
0.63271936	0.62159562	Quantified
1.1330524	1.0777804	Quantified
0.90991956	0.89068191	Quantified
1.06636909	1.08390899	Quantified
1.01060017	0.98030136	Quantified
0.83441494	0.80702023	Quantified
1.11637025	1.10498988	Quantified
1.09139319	1.08391244	Quantified

1.03919202	1.03333977	Quantified
1.11594341	1.14678744	Quantified
0.84656208	0.80596424	Quantified
1.0809667	1.05320425	Quantified
0.93717355	0.89708242	Quantified
0.91494894	0.99757598	Quantified
1.13330474	1.07360154	Quantified
0.97919375	1.0517652	Quantified
0.64282229	0.53265303	Quantified
0.90594126	0.90358076	Quantified
1.19219577	1.11442784	Quantified
0.98814061	0.96648687	Quantified
1.02264177	1.0641598	Quantified
1.08968974	0.97678355	Quantified
1.07371874	1.04311256	Quantified
0.92066732	0.92278599	Quantified
1.00170046	0.99657469	Quantified
0.90183191	0.78285065	Quantified
1.17437864	1.18930939	Quantified
0.95140271	1.12730952	Quantified
1.01491855	1.00932085	Quantified
1.12381312	1.08238558	Quantified
1.02042879	1.01741058	Quantified
0.96525952	1.00850182	Quantified
0.95686856	0.9458119	Quantified
1.03817322	1.0465048	Quantified
1.19537619	1.17255598	Quantified
1.25404666	1.21257979	Quantified
1.23568268	1.22759207	Quantified
0.91222842	0.81618674	Quantified
0.97801442	0.94445596	Quantified
0.97617483	0.97373536	Quantified
0.80610716	0.78977891	Quantified
0.9928951	0.84894323	Quantified
1.08127115	1.10467974	Quantified
1.03996222	0.98664613	Quantified
0.99525261	0.99326906	Quantified
1.19649348	1.15442081	Quantified
0.92315039	0.89495468	Quantified
1.29343915	1.29222017	Quantified
1.14737789	1.09485713	Quantified
1.00407053	0.96125544	Quantified
0.99829492	1.00068318	Quantified
1.07732767	1.01436108	Quantified
0.93657744	0.76677125	Quantified
1.02619714	1.01824651	Quantified
1.00007042	0.99447885	Quantified
0.97458261	1.03155873	Quantified
1.06108588	0.99713639	Quantified
1.33251759	1.37008922	Quantified

0.99997109	0.9883293	Quantified
1.02668867	1.03201447	Quantified
1.08822703	1.11198354	Quantified
0.90483911	0.92179094	Quantified
1.0127831	1.01285587	Quantified
1.05264213	1.02961403	Quantified
1.08330717	1.06244414	Quantified
1.25472781	1.10436579	Quantified
0.81647668	0.76588425	Quantified
0.94932021	0.93489932	Quantified
0.77150975	0.70500798	Quantified
0.91443591	0.96002304	Quantified
1.07925374	1.07422841	Quantified
1.25060535	1.21409593	Quantified
1.04014597	1.07515729	Quantified
1.39090865	1.42981667	Quantified
1.10656827	0.99598111	Quantified
0.94714564	0.96606548	Quantified
0.92054499	0.9613716	Quantified
0.82703619	0.8917634	Quantified
0.992449	1.01095809	Quantified
0.92729459	0.94087489	Quantified
0.83127313	0.82075347	Quantified
1.09056179	1.05610879	Quantified
1.01049149	0.99025917	Quantified
1.023537	1.00807547	Quantified
1.27913123	1.27579909	Quantified
1.02843707	1.03783606	Quantified
1.00602756	1.16840852	Quantified
1.05342509	1.05615181	Quantified
1.17658421	1.21146871	Quantified
0.91152565	0.8766196	Quantified
0.62445566	0.55656462	Quantified
1.17315361	1.16615912	Quantified
1.00642435	0.99995576	Quantified
0.94641509	0.9558994	Quantified
0.51584588	0.5808222	Quantified
0.98282049	1.00518908	Quantified
0.93129581	0.89726595	Quantified
1.13320715	1.13185612	Quantified
0.84686621	0.78245055	Quantified
0.99112514	1.01815625	Quantified
0.92948201	1.01899122	Quantified
1.34535481	1.37328482	Quantified
1.068273	1.07228941	Quantified
0.97263199	0.9990745	Quantified
1.0297144	1.04239792	Quantified
0.87816104	0.8717406	Quantified
1.13348539	1.13344786	Quantified
1.03469133	1.06812595	Quantified

0.752027	0.92889267	Quantified
1.28460294	1.05841006	Quantified
1.04772305	1.02175903	Quantified
0.93430473	1.07706652	Quantified
0.974212	0.95648875	Quantified
1.02988349	0.99771778	Quantified
1.16561206	1.13437505	Quantified
0.95001583	0.98046212	Quantified
0.9272179	0.89589978	Quantified
0.84170466	0.84850087	Quantified
1.01889787	1.03282881	Quantified
0.9888415	1.0005606	Quantified
0.97381664	0.94800299	Quantified
0.9841094	1.18486769	Quantified
1.04044941	1.02390232	Quantified
0.69474765	0.61607466	Quantified
1.0186371	1.08409347	Quantified
1.02388446	1.00004424	Quantified
1.12106684	1.08237315	Quantified
1.11491157	1.09499074	Quantified
1.09247036	1.0835391	Quantified
0.89539217	0.7944888	Quantified
1.13980022	1.15765832	Quantified
0.95279841	0.9153252	Quantified
1.05005531	1.02715225	Quantified
0.93480196	0.94618099	Quantified
1.08124134	0.95117896	Quantified
0.95232758	0.91638388	Quantified
0.96510566	0.83961437	Quantified
1.05767607	1.01871721	Quantified
1.04218337	0.94375717	Quantified
0.99123234	0.99242875	Quantified
0.77059727	0.71902081	Quantified
0.9892446	0.98642873	Quantified
0.98464289	0.99021978	Quantified
1.07275048	1.04384249	Quantified
1.03148814	1.08087445	Quantified
1.07842701	1.04234503	Quantified
1.02251783	1.0846557	Quantified
1.04464851	1.02866641	Quantified
0.98843873	0.93211113	Quantified
1.06302551	1.03099425	Quantified
0.83324801	0.83270983	Quantified
0.98067559	0.95158224	Quantified
0.9597193	0.96450521	Quantified
0.78365073	1.11293558	Quantified
0.89372408	0.91371071	Quantified
0.97374854	0.98540938	Quantified
0.97474631	0.94786053	Quantified
0.97372102	0.95648384	Quantified

1.04649399	1.04388958	Quantified
0.96559049	0.95273835	Quantified
1.06396809	1.05356214	Quantified
1.10522381	1.0751648	Quantified
1.0191582	0.99913523	Quantified
0.93265917	0.81210775	Quantified
1.01229191	1.01335496	Quantified
1.09397033	1.09131642	Quantified
1.01291529	1.04846594	Quantified
1.08618364	1.07525398	Quantified
0.48227977	0.45998461	Quantified
0.99934394	0.99278782	Quantified
1.02360625	1.04400113	Quantified
1.06149855	1.10227587	Quantified
0.48473797	0.46042642	Quantified
1.05660409	1.05891762	Quantified
1.05472354	1.03115707	Quantified
0.53599243	0.4507023	Quantified
1.15442148	1.20440015	Quantified
0.92856389	0.90822782	Quantified
1.03529526	1.03212618	Quantified
0.93297105	1.34541897	Quantified
0.96895454	0.94881747	Quantified
1.08972471	0.998196	Quantified
0.82236698	0.78587496	Quantified
0.9736814	0.9700213	Quantified
1.00850316	0.93800473	Quantified
1.0452173	1.01585452	Quantified
1.0514165	1.03139179	Quantified
0.96643376	0.9593453	Quantified
1.03011865	1.04419097	Quantified
1.02735408	0.98185555	Quantified
0.98528352	0.98265232	Quantified
1.11408243	1.13506963	Quantified
0.91887759	0.95042394	Quantified
0.96603341	1.05885263	Quantified
1.23720281	1.24261429	Quantified
1.0080563	1.02993383	Quantified
0.99194963	0.93764934	Quantified
1.11129789	1.10245071	Quantified
1.25993426	1.13971861	Quantified
0.98146525	0.96948697	Quantified
1.04477053	1.08046899	Quantified
0.92591585	0.90920309	Quantified
0.9795951	0.99472265	Quantified
0.9618779	1.01657895	Quantified
0.93359704	0.90206399	Quantified
0.89658578	0.90478445	Quantified
1.18900893	1.21691299	Quantified
0.97399477	0.92558601	Quantified

1.05087413	1.04620064	Quantified
1.07333329	1.06012564	Quantified
0.80152036	0.78821502	Quantified
1.06669	1.05037191	Quantified
1.02887057	1.07244819	Quantified
1.0339136	1.02960854	Quantified
0.99081973	0.84126005	Quantified
0.7492451	0.64082519	Quantified
1.00667422	1.01669718	Quantified
1.01734701	0.91326082	Quantified
1.17302413	1.13844299	Quantified
1.25007239	1.22923126	Quantified
1.00144442	0.99423818	Quantified
0.98706954	0.98679188	Quantified
0.57788354	0.56857999	Quantified
1.01377779	1.00973758	Quantified
0.95501246	0.97849883	Quantified
0.9875102	1.00496653	Quantified
0.96321113	1.03425168	Quantified
0.86266149	0.80965491	Quantified
1.11726347	1.13650256	Quantified
0.99717351	0.98935748	Quantified
0.75907129	0.75836099	Quantified
1.05941757	0.99721263	Quantified
1.01188875	0.95722459	Quantified
1.24784854	1.26542453	Quantified
0.96951189	0.97699841	Quantified
1.07688895	1.05976841	Quantified
0.93554806	0.94296362	Quantified
1.15841126	1.04288085	Quantified
1.00686946	0.73185051	Quantified
1.09465549	1.08595658	Quantified
1.16152941	1.04028081	Quantified
0.99940017	0.99101524	Quantified
0.96566816	0.92878526	Quantified
0.75051004	0.6990474	Quantified
1.04506445	1.0860435	Quantified
1.07564706	1.06986495	Quantified
1.0331479	1.03872896	Quantified
1.01130049	0.99411623	Quantified
0.87248016	0.78802514	Quantified
1.3074383	1.28003117	Quantified
0.94849714	0.83916537	Quantified
1.12119815	1.07804893	Quantified
0.99317727	0.98054365	Quantified
1.03644094	1.04801154	Quantified
1.08278361	1.17580947	Quantified
1.01292142	1.02837681	Quantified
1.06873417	1.07014209	Quantified
0.95868443	0.95091954	Quantified

0.60197537	0.60604104	Quantified
0.43727164	0.41816621	Quantified
0.89699836	0.83670913	Quantified
0.99052186	0.98406	Quantified
1.2325251	0.98216153	Quantified
0.94973713	0.92757415	Quantified
1.01026637	1.01967755	Quantified
1.09165265	1.11020498	Quantified
1.09665934	1.14680917	Quantified
1.01646201	1.01668294	Quantified
1.04294125	1.02840162	Quantified
1.00291136	0.97634542	Quantified
1.02280214	1.02611332	Quantified
1.08165307	1.08637083	Quantified
1.19812505	1.20430883	Quantified
0.59461261	0.55838736	Quantified
1.16209536	1.14664909	Quantified
0.8816268	0.87095289	Quantified
0.85662315	0.78991595	Quantified
1.1137932	1.14531	Quantified
0.95693542	0.94355115	Quantified
0.99937239	0.99908421	Quantified
0.73120192	0.70737416	Quantified
0.94501801	0.93775716	Quantified
0.90674778	0.90202083	Quantified
0.99962317	1.10255186	Quantified
1.03788874	0.99919522	Quantified
0.98298413	0.98382115	Quantified
0.96563934	0.95464694	Quantified
1.00383364	0.97928812	Quantified
0.99598751	0.96246863	Quantified
1.19499734	1.18715139	Quantified
0.29517811	0.29629193	Quantified
0.90360989	0.91431405	Quantified
1.06222169	1.06734097	Quantified
1.06058439	1.04627301	Quantified
1.00145524	1.00027938	Quantified
1.02799212	0.91763572	Quantified
1.02918701	1.07636197	Quantified
0.57707925	0.52513337	Quantified
0.87756148	0.94578583	Quantified
0.96313836	0.94180976	Quantified
1.00863985	1.02543864	Quantified
1.00057349	0.98236705	Quantified
1.06345015	1.08320549	Quantified
1.01134049	0.97043619	Quantified
1.18219701	1.18095513	Quantified
0.97809358	0.97656333	Quantified
0.928544	0.85599926	Quantified
1.00033713	1.14201959	Quantified

0.51514055	0.48775279	Quantified
0.95898531	0.93835421	Quantified
0.95151975	0.93843436	Quantified
0.66411631	0.73182886	Quantified
1.18546228	1.21029007	Quantified
1.05820538	1.08000139	Quantified
1.01326748	1.04717065	Quantified
0.81654928	0.78927424	Quantified
1.02337654	1.03716923	Quantified
0.9753814	0.90176858	Quantified
1.03316447	1.02207828	Quantified
1.00742156	0.99657768	Quantified
1.00338298	1.06499945	Quantified
1.07763716	1.0899832	Quantified
0.94915801	1.02533147	Quantified
1.00204605	0.99404101	Quantified
1.08373168	1.04967385	Quantified
1.02974621	0.96264792	Quantified
1.01433044	1.04300313	Quantified
0.92471826	0.99850724	Quantified
0.9446374	0.92563983	Quantified
1.03915895	0.97881468	Quantified
1.11422228	1.06881549	Quantified
1.03012663	1.03384326	Quantified
1.04980313	1.08270895	Quantified
1.1131734	1.10883322	Quantified
1.02272632	0.99621693	Quantified
0.96872885	0.87865522	Quantified
0.85694168	0.9160856	Quantified
1.07205976	1.06092465	Quantified
0.56349683	0.54227868	Quantified
1.01875911	1.00782438	Quantified
1.04856986	1.04721465	Quantified
0.79987086	0.77858531	Quantified
0.8678886	0.7675171	Quantified
1.0720866	1.11357339	Quantified
1.06637612	1.01733747	Quantified
0.82886167	0.80431294	Quantified
0.99910878	0.96143993	Quantified
1.05344607	1.05526504	Quantified
0.97624948	0.95327143	Quantified
0.6595963	0.56686	Quantified
0.95654717	1.06071052	Quantified
0.97498124	0.99047466	Quantified
1.07992161	1.10189766	Quantified
1.0791481	1.11664324	Quantified
1.02978873	1.03825307	Quantified
1.30783759	1.255855	Quantified
0.89380615	0.84753217	Quantified
0.96809349	0.98865297	Quantified

0.68147964	0.66714552	Quantified
0.99958903	0.95237655	Quantified
1.00934828	0.99156664	Quantified
0.99430922	1.11887187	Quantified
0.95393353	0.94050542	Quantified
1.0222111	0.88897312	Quantified
1.24455288	1.26196364	Quantified
1.07935557	1.12954134	Quantified
1.00952254	0.9782053	Quantified
0.99057372	0.98476031	Quantified
0.93425629	0.92685016	Quantified
1.0085939	1.00123691	Quantified
1.01310881	0.99136251	Quantified
0.98389989	0.98840514	Quantified
0.93877001	0.84566504	Quantified
1.01922472	1.02441496	Quantified
0.98031507	1.02050383	Quantified
1.02354337	0.95332127	Quantified
1.01308252	0.95077445	Quantified
1.0523694	1.04273672	Quantified
1.06240033	1.03571679	Quantified
0.95230239	0.9943489	Quantified
0.90984252	0.85813905	Quantified
0.95937407	0.95080606	Quantified
1.04110411	1.01994671	Quantified
1.00307027	1.00392232	Quantified
1.08224841	1.02005379	Quantified
0.7123685	0.70511168	Quantified
1.11772024	1.12949441	Quantified
0.90605999	0.88962372	Quantified
0.94211699	0.9739269	Quantified
0.9898529	0.95375201	Quantified
1.0372847	0.95355186	Quantified
0.4926853	1.04803676	Quantified
1.00826727	1.00043024	Quantified
0.98910296	0.99675582	Quantified
1.03380199	1.02977774	Quantified
0.99186283	1.00662353	Quantified
1.03051766	1.02901793	Quantified
0.91445851	0.88351497	Quantified
1.01331566	0.99348717	Quantified
1.01780738	1.0026798	Quantified
0.63082039	0.63188021	Quantified
0.96831965	0.98425193	Quantified
0.94069077	0.95242946	Quantified
0.85472543	0.90032994	Quantified
0.60942898	0.59429575	Quantified
0.84235047	0.8222118	Quantified
1.07111188	1.05617791	Quantified
0.89847602	0.9135578	Quantified

0.92418132	0.71994864	Quantified
0.90826288	0.9086683	Quantified
1.01819133	0.9930942	Quantified
1.00938756	0.99576025	Quantified
0.65107359	0.66911413	Quantified
0.57890506	0.54158153	Quantified
1.0540039	1.10009385	Quantified
0.65486921	0.64393146	Quantified
0.8239941	1.14042149	Quantified
0.80880117	0.74778832	Quantified
0.83539677	0.81384419	Quantified
1.06161095	1.02864839	Quantified
0.98978443	0.98005306	Quantified
1.42277318	1.38768743	Quantified
0.99399379	1.01404784	Quantified
0.95537211	0.94636643	Quantified
0.99483135	1.06066354	Quantified
1.0515894	1.0192622	Quantified
1.12273831	0.9606896	Quantified
0.9406633	0.94427725	Quantified
0.83506254	0.81476882	Quantified
0.88825616	1.01152399	Quantified
1.12330623	1.16208762	Quantified
0.78365699	0.78540957	Quantified
0.96986038	0.94171533	Quantified
1.02756605	0.99104695	Quantified
1.04437616	1.0292417	Quantified
1.09871409	1.0982734	Quantified
1.01819176	0.96960318	Quantified
1.07697657	1.10833689	Quantified
1.14255322	1.22606496	Quantified
0.94984052	0.92864168	Quantified
1.06893379	0.99081909	Quantified
1.05783904	1.02064718	Quantified
1.4236382	1.00440109	Quantified
1.19281699	1.24587935	Quantified
1.00147914	0.97805982	Quantified
1.00907591	1.01924325	Quantified
0.83044679	0.80180914	Quantified
0.97978587	0.89556739	Quantified
0.8737706	0.87837106	Quantified
0.97967339	0.97915368	Quantified
0.94508139	0.83575158	Quantified
1.00346288	1.01942066	Quantified
1.04405262	1.03087824	Quantified
0.92991336	0.81331877	Quantified
1.14676956	1.13694159	Quantified
1.05613856	1.06340152	Quantified
1.00807649	0.98845059	Quantified
0.76828123	0.77155764	Quantified

1.04862167	0.99953771	Quantified
1.02613391	1.05432038	Quantified
1.02574709	1.01952112	Quantified
1.00078425	0.9894287	Quantified
1.15621374	1.15181457	Quantified
0.89442841	0.90166752	Quantified
0.99179791	1.02938851	Quantified
1.01354913	1.05225787	Quantified
0.90694473	0.92265245	Quantified
0.97248026	0.99283639	Quantified
1.0338095	1.0347913	Quantified
1.09368412	1.0352809	Quantified
1.08651316	0.99282836	Quantified
0.99567385	0.98794134	Quantified
1.31267885	1.37728427	Quantified
1.00364957	1.00645063	Quantified
0.97748097	0.96274857	Quantified
0.91987381	0.93525643	Quantified
0.83312811	0.90454736	Quantified
1.08125322	1.08396875	Quantified
1.0212466	1.00382138	Quantified
1.00917281	1.0129172	Quantified
1.19266237	1.11573595	Quantified
0.77136353	0.77194731	Quantified
0.86357097	0.87263424	Quantified
0.99043717	0.99560845	Quantified
0.93609113	0.96917411	Quantified
0.89759573	0.89459048	Quantified
1.04841973	1.06697584	Quantified
1.04433372	1.13995014	Quantified
0.99389532	1.01352959	Quantified
1.01687376	1.0099423	Quantified
0.85793243	0.82966928	Quantified
0.97919692	0.96634195	Quantified
1.01836543	0.96790276	Quantified
0.98766594	0.88933075	Quantified
0.9884944	1.00954499	Quantified
1.09151474	1.05998878	Quantified
1.10080291	1.09377161	Quantified
1.08328436	1.09167585	Quantified
0.97662962	0.97030907	Quantified
0.9212726	0.83133385	Quantified
1.06447775	1.07343784	Quantified
1.04775205	1.10669512	Quantified
1.15042407	1.13254672	Quantified
1.1344859	1.08625594	Quantified
1.0092825	1.01483846	Quantified
1.06361364	1.07856246	Quantified
1.07645721	1.00656023	Quantified
0.96953893	0.90138722	Quantified

0.84103246	0.69752729	Quantified
0.94863718	0.82350546	Quantified
0.8647029	0.90356976	Quantified
0.98351883	0.96643687	Quantified
1.03873655	0.98039989	Quantified
0.93474071	0.89119247	Quantified
1.07474356	1.10191118	Quantified
1.01337622	0.96922701	Quantified
1.01992411	1.02165985	Quantified
0.92196553	0.94654826	Quantified
0.94265898	0.93024607	Quantified
0.97763842	0.88089241	Quantified
0.70827759	0.71526683	Quantified
1.24867264	1.0081464	Quantified
0.95519426	0.93803636	Quantified
1.04561704	1.04794773	Quantified
1.10251927	0.98694216	Quantified
1.08909742	1.05983559	Quantified
1.06622235	1.08563878	Quantified
0.93444263	0.94132371	Quantified
0.99250492	1.11978873	Quantified
0.96152701	0.94851731	Quantified
1.07634875	1.06481746	Quantified
1.0248595	1.0002573	Quantified
0.97205782	0.94384591	Quantified
0.97152332	0.97899572	Quantified
1.03866251	1.07463515	Quantified
0.8999674	0.88146271	Quantified
0.99460142	0.99791878	Quantified
1.04925782	1.04650341	Quantified
0.88968535	0.88910587	Quantified
0.97891733	0.99367149	Quantified
0.96666662	0.9640075	Quantified
0.65221547	0.62867414	Quantified
1.022475	1.00361318	Quantified
1.02155917	0.98295243	Quantified
1.03670051	1.07194846	Quantified
1.02423074	1.01268893	Quantified
1.2415535	1.23947605	Quantified
0.89984822	0.88505966	Quantified
1.00583315	1.01615932	Quantified
1.17844424	1.17939585	Quantified
1.09959996	1.08894341	Quantified
1.05405881	1.04608128	Quantified
1.0988181	1.26823001	Quantified
0.97940444	0.97145912	Quantified
0.66577588	0.65016003	Quantified
0.97511168	1.04464261	Quantified
1.06639619	1.00193826	Quantified
0.898927	0.86340975	Quantified

0.96875527	1.00333617	Quantified
1.02536556	1.00462168	Quantified
1.06518716	1.07373108	Quantified
1.28806962	1.2222313	Quantified
0.83521532	0.79596926	Quantified
0.98480877	1.0775578	Quantified
0.68216034	0.63605113	Quantified
0.99697577	0.98902085	Quantified
1.15142314	1.14678789	Quantified
1.04563458	1.05546445	Quantified
0.98315943	0.97477699	Quantified
0.98359443	0.99086929	Quantified
1.14306671	1.12745647	Quantified
1.08700308	1.11350957	Quantified
1.06793165	1.08244381	Quantified
0.99343745	1.02823187	Quantified
0.87803537	0.84608013	Quantified
1.17218461	1.2253013	Quantified
1.10139726	1.03005337	Quantified
1.13301015	1.0879838	Quantified
1.0403278	1.03764089	Quantified
0.94021491	0.91279639	Quantified
1.20068696	1.25572617	Quantified
0.98376098	0.9779006	Quantified
1.01192086	0.99513759	Quantified
0.99832437	0.94662391	Quantified
1.0103999	0.96884655	Quantified
1.09228887	1.18301822	Quantified
0.99280881	1.007424	Quantified
0.99068426	0.97397802	Quantified
0.79721735	0.7609015	Quantified
0.85190242	0.90681371	Quantified
1.02031136	1.01259759	Quantified
0.53505504	0.52676909	Quantified
1.02857526	0.98357307	Quantified
0.8832548	0.84658023	Quantified
1.02925539	1.02035685	Quantified
0.94285561	0.9346817	Quantified
1.13378404	1.13563964	Quantified
0.91316595	0.96134982	Quantified
1.01474093	1.01087597	Quantified
0.91260651	0.9069686	Quantified
0.53162953	0.51329674	Quantified
1.03523211	1.06058145	Quantified
0.93677611	0.90269106	Quantified
0.77598534	0.73244308	Quantified
0.99356772	0.98155849	Quantified
1.05596445	1.05127941	Quantified
0.73500711	0.74338743	Quantified
0.95643524	0.96147421	Quantified

0.6283508	0.47402014	Quantified
1.12815176	1.044413	Quantified
1.06856657	1.06903416	Quantified
0.87289682	0.87473037	Quantified
0.58706256	0.62239711	Quantified
1.00171062	0.96818222	Quantified
1.02954103	1.05174741	Quantified
1.07997391	1.03051129	Quantified
1.07385298	1.08187786	Quantified
0.9973101	1.01822247	Quantified
0.99108954	1.01857905	Quantified
0.98692427	0.96845628	Quantified
0.93039843	0.98633887	Quantified
1.0888984	1.05859615	Quantified
0.99400097	0.98075474	Quantified
0.88775961	0.8787944	Quantified
0.96757933	1.00205388	Quantified
0.89726266	0.90071943	Quantified
0.98462023	0.96526465	Quantified
1.05920884	1.09753385	Quantified
0.88174486	0.95819191	Quantified
1.00983863	1.02390336	Quantified
1.02792479	0.98599375	Quantified
1.02911219	1.0449835	Quantified
1.07736649	1.08727293	Quantified
1.12808988	1.10301359	Quantified
1.00618752	0.97595874	Quantified
1.06361326	1.06078436	Quantified
1.01686061	1.04444466	Quantified
0.91319197	0.75904038	Quantified
0.99287228	0.96870093	Quantified
0.9527447	0.99754029	Quantified
1.17354061	1.13751133	Quantified
1.03045656	0.99884952	Quantified
0.98590104	0.96873868	Quantified
1.08812841	1.1422003	Quantified
1.01982118	1.0232222	Quantified
1.08874047	1.02504036	Quantified
1.07342521	1.04433613	Quantified
0.98113413	0.98222662	Quantified
1.10055743	1.0703965	Quantified
1.04391045	1.09619401	Quantified
0.9325935	0.91910297	Quantified
0.69844942	0.69157732	Quantified
1.03536468	1.05877766	Quantified
1.39423588	0.90051839	Quantified
1.03010086	1.06354154	Quantified
1.07741163	1.06800305	Quantified
0.99325771	1.0014464	Quantified
0.98837333	0.9909113	Quantified

1.07515234	1.05187756	Quantified
1.11289271	1.08944649	Quantified
0.87595109	0.77528334	Quantified
0.99693525	1.01124816	Quantified
0.66550873	0.65301771	Quantified
0.95258579	0.94882691	Quantified
1.13101851	1.14344101	Quantified
1.1893788	1.16488932	Quantified
1.00519414	1.01103906	Quantified
0.84602836	0.69836825	Quantified
0.89801945	1.04657627	Quantified
0.81232836	0.78837593	Quantified
1.03898492	0.97679273	Quantified
1.01256384	1.0266999	Quantified
0.99395124	1.04509669	Quantified
1.06877314	1.08002981	Quantified
0.92420428	0.89886756	Quantified
1.06395272	1.043209	Quantified
1.05186806	1.05632699	Quantified
1.14143031	1.03404686	Quantified
0.62260662	0.62767218	Quantified
1.12034994	1.07237298	Quantified
1.09193739	1.10101038	Quantified
0.78787416	0.76544517	Quantified
0.96897208	0.96788395	Quantified
0.95020848	0.93389902	Quantified
1.09879492	1.13985407	Quantified
0.94698578	0.84429908	Quantified
0.88241615	0.92065105	Quantified
0.96201938	0.85413073	Quantified
0.99383755	0.94289088	Quantified
0.94500476	1.02892267	Quantified
1.03205612	1.12561153	Quantified
1.09235303	1.09357762	Quantified
1.05390435	0.89976828	Quantified
1.01453525	0.9815468	Quantified
1.13062147	1.12480926	Quantified
0.94110997	0.89880003	Quantified
0.98050137	0.96699302	Quantified
1.01965983	1.04439641	Quantified
0.94911642	0.99187831	Quantified
0.88356427	0.92193978	Quantified
0.89737425	0.94648155	Quantified
0.82026369	0.79657938	Quantified
1.01605038	1.00837218	Quantified
1.05476249	1.06867687	Quantified
1.05114904	1.07564754	Quantified
1.0380782	1.03486201	Quantified
1.03009098	1.01599401	Quantified
1.00539262	1.01520265	Quantified

0.87932741	0.81865225	Quantified
0.69011069	0.6618814	Quantified
0.93598414	0.96386763	Quantified
0.96747463	0.95367044	Quantified
1.06897089	1.11901386	Quantified
1.0386639	1.01213136	Quantified
1.1745079	1.15369083	Quantified
1.08958906	1.08980245	Quantified
0.96314177	0.88847583	Quantified
0.75927556	0.71249374	Quantified
0.9655836	1.01357286	Quantified
0.94940695	0.95156794	Quantified
1.07837316	1.09051854	Quantified
1.00629575	0.98107454	Quantified
1.12334262	1.07085927	Quantified
0.96297533	0.97493023	Quantified
0.94823285	0.91985586	Quantified
1.1635715	1.18419861	Quantified
0.92003237	0.93160986	Quantified
1.21226585	1.20741383	Quantified
1.08184059	1.06503956	Quantified
0.95361459	0.92689702	Quantified
1.1427848	1.12898128	Quantified
1.00575995	1.0167223	Quantified
1.02935955	0.99094593	Quantified
1.0470986	1.01692058	Quantified
0.93543909	0.93354896	Quantified
1.11327284	1.12174784	Quantified
0.98779733	0.88097113	Quantified
1.12221319	1.10664654	Quantified
1.02756418	1.02665785	Quantified
0.93361851	0.93247934	Quantified
1.02776982	1.01925886	Quantified
0.8778077	0.79711211	Quantified
0.94711591	0.91870477	Quantified
1.03530886	1.05013564	Quantified
0.99221174	1.0135828	Quantified
0.78200113	0.82598189	Quantified
1.17019253	1.11545313	Quantified
0.9936669	1.00554607	Quantified
1.02595402	1.00021501	Quantified
0.96396229	0.96140954	Quantified
1.0139005	0.91388855	Quantified
0.96856453	0.94185437	Quantified
0.87766489	0.84977233	Quantified
1.03637881	1.06887138	Quantified
1.03807332	1.10966621	Quantified
1.03318496	1.0136216	Quantified
1.07504698	1.10678238	Quantified
0.90163941	0.90202525	Quantified

0.4964053	0.52045689	Quantified
1.04626422	1.00874742	Quantified
1.04128511	1.04226511	Quantified
0.85953569	0.82291856	Quantified
1.06409419	1.04760277	Quantified
0.87772219	0.84692277	Quantified
1.04524323	0.94691822	Quantified
0.99973362	0.99707469	Quantified
1.00400927	1.01046703	Quantified
1.03051034	1.00646965	Quantified
1.0480734	1.0647023	Quantified
1.02428934	0.9973668	Quantified
1.02406081	0.98097879	Quantified
0.95435214	0.89846876	Quantified
1.02457437	1.0288842	Quantified
0.91704447	0.91472911	Quantified
0.89126268	0.93594406	Quantified
0.99024678	0.98628679	Quantified
1.17509249	1.14970993	Quantified
1.06540286	1.04916485	Quantified
0.80788928	0.65497855	Quantified
1.02945601	1.03113783	Quantified
0.98619124	0.93428053	Quantified
0.77663432	0.79157352	Quantified
0.73259945	0.62915532	Quantified
0.86089891	0.82343116	Quantified
0.82090548	0.9104557	Quantified
1.08758887	1.05192226	Quantified
1.04442696	1.03121563	Quantified
1.05709212	1.06974547	Quantified
0.98693597	1.02705056	Quantified
0.94773835	0.95369228	Quantified
0.79438175	0.78896261	Quantified
1.11730037	1.14948923	Quantified
0.98101971	1.03838013	Quantified
0.86046347	0.85507908	Quantified
1.11145247	1.21531508	Quantified
0.9931764	0.96648496	Quantified
0.94875489	0.90160725	Quantified
0.90736035	0.90596308	Quantified
1.10250707	1.1262923	Quantified
1.08207585	1.08348673	Quantified
0.99419182	0.98475994	Quantified
1.01101694	0.92478899	Quantified
0.96383608	1.15180086	Quantified
1.01849084	1.06624045	Quantified
0.92765691	0.88974511	Quantified
1.01281325	0.99167772	Quantified
0.75578222	0.78762154	Quantified
0.919892	0.84119589	Quantified

1.13375233	1.00861796	Quantified
0.95544674	1.01735561	Quantified
0.98399558	0.96119063	Quantified
0.9854195	1.03565949	Quantified
1.00311142	1.08252537	Quantified
0.87868168	0.75652866	Quantified
0.95364927	0.95150854	Quantified
1.09326193	1.05786903	Quantified
1.19094278	1.20403671	Quantified
1.0491501	1.00463115	Quantified
1.0514906	1.04891626	Quantified
1.03735109	1.03586189	Quantified
1.05269939	1.06326	Quantified
0.91031689	0.89659815	Quantified
1.06345401	0.99381026	Quantified
1.01427651	1.09033368	Quantified
0.94742603	0.94507029	Quantified
1.06480948	1.07544413	Quantified
1.07195161	1.06026222	Quantified
0.82348554	0.87500566	Quantified
0.98707278	0.98139383	Quantified
1.07185741	1.13082512	Quantified
0.94466546	0.94507718	Quantified
1.03434562	1.04819138	Quantified
1.04332734	1.00592631	Quantified
1.04332037	1.05940582	Quantified
1.06145622	1.07020499	Quantified
0.97141765	0.88951221	Quantified
0.99128413	0.96130365	Quantified
1.21668919	1.25327476	Quantified
1.01247666	1.0315722	Quantified
1.04858345	1.09862318	Quantified
0.85277127	0.85173858	Quantified
1.02451632	1.04626774	Quantified
0.88199437	0.88553421	Quantified
1.01335717	0.98625664	Quantified
1.05179885	1.05724186	Quantified
0.95975759	0.89455712	Quantified
1.00895375	0.97870426	Quantified
0.93613037	0.93933149	Quantified
0.80284385	0.80302619	Quantified
0.94959259	0.90507946	Quantified
0.9410786	0.9193534	Quantified
0.98985557	1.02567071	Quantified
1.07171023	1.13628428	Quantified
0.97018923	1.00728884	Quantified
1.0668747	1.02732679	Quantified
1.00873662	1.03676563	Quantified
0.80006589	0.81360211	Quantified
0.71887154	0.58471194	Quantified

0.98785707	0.98251865	Quantified
0.95896154	0.97079078	Quantified
1.03608132	0.99109179	Quantified
1.05072304	1.08515931	Quantified
0.83105912	0.77022562	Quantified
1.14976866	1.1388358	Quantified
1.02480573	0.90887809	Quantified
1.21686193	1.02424577	Quantified
0.94225061	0.97740344	Quantified
1.0994846	1.11898423	Quantified
0.95679853	0.97918753	Quantified
0.74002346	0.68113199	Quantified
0.94578628	1.01582633	Quantified
1.23275241	1.23764733	Quantified
1.08145279	1.2208335	Quantified
1.07200675	1.08229659	Quantified
1.0931171	1.03526562	Quantified
0.91710936	0.88319691	Quantified
0.72143194	0.74289871	Quantified
1.03443036	1.01131377	Quantified
1.0519131	1.05635733	Quantified
1.09115686	1.0675078	Quantified
0.99915402	1.02037663	Quantified
1.39540133	1.48642922	Quantified
0.83259398	0.85577559	Quantified
1.05532401	1.10615806	Quantified
0.84613884	0.86436961	Quantified
0.9994178	0.96564264	Quantified
0.86454153	0.63838829	Quantified
0.94690336	0.93696753	Quantified
1.03910738	1.03951649	Quantified
1.0028722	0.97265283	Quantified
1.13994072	1.13771219	Quantified
0.91311074	0.90704783	Quantified
1.13790478	1.11887607	Quantified
1.0330373	0.97084427	Quantified
1.00804695	1.04444736	Quantified
1.04799582	1.04758967	Quantified
0.99696079	0.96889726	Quantified
1.16245972	1.18877853	Quantified
1.07538485	1.01958535	Quantified
1.08992907	1.1298142	Quantified
1.07889126	1.05470453	Quantified
1.00940969	1.02083231	Quantified
1.01428767	1.04466933	Quantified
1.06926665	1.02188797	Quantified
1.00316001	1.16646901	Quantified
0.97646923	0.96615411	Quantified
0.8594237	0.91333244	Quantified
1.01423394	0.95088919	Quantified

0.98209695	0.97696493	Quantified
1.03071219	1.04497849	Quantified
1.0410844	1.01955951	Quantified
0.94464235	0.92915239	Quantified
1.09909847	1.22927132	Quantified
0.95967351	1.00505541	Quantified
0.88567242	0.86975212	Quantified
0.8853363	0.92569937	Quantified
1.06590625	1.05437173	Quantified
1.07707889	1.06526573	Quantified
0.90114208	0.84416448	Quantified
0.98820519	0.97440207	Quantified
1.02889265	1.02292743	Quantified
1.05647253	1.02085553	Quantified
1.02786741	0.94726835	Quantified
1.03862891	1.1327799	Quantified
0.55113098	0.54347889	Quantified
1.0517915	1.03223982	Quantified
1.05339446	1.04560387	Quantified
0.9465602	0.95465021	Quantified
0.96988105	0.98231556	Quantified
0.93973826	0.96264049	Quantified
1.0837437	1.15224132	Quantified
0.97302181	0.95586159	Quantified
1.0384258	1.01028689	Quantified
1.02396089	0.98399071	Quantified
0.98059206	1.0623532	Quantified
1.06115663	0.99253001	Quantified
0.90331778	0.85718796	Quantified
0.99664642	0.96109321	Quantified
0.99870386	1.0356742	Quantified
0.92794316	0.93917781	Quantified
0.94978079	0.97017495	Quantified
0.99836834	0.93013644	Quantified
0.88786339	0.88984926	Quantified
0.99761473	1.02048337	Quantified
0.74686153	0.80852931	Quantified
0.82597272	0.82857413	Quantified
0.9563482	0.99043317	Quantified
1.07370912	1.08776519	Quantified
1.01694189	0.94349536	Quantified
1.39892752	1.40815976	Quantified
0.97580531	0.98072428	Quantified
0.99613024	1.04743814	Quantified
0.94180837	0.88121998	Quantified
0.87979997	0.88924419	Quantified
0.96858641	0.96730002	Quantified
0.96742283	1.02532845	Quantified
1.09429884	1.11643849	Quantified
1.05179253	1.09062198	Quantified

1.0597879	1.03852394	Quantified
0.80385297	0.67394254	Quantified
0.59266035	0.58895589	Quantified
0.93742246	1.03856208	Quantified
0.99707193	0.97116179	Quantified
1.05021232	1.05658664	Quantified
1.10724151	1.09644296	Quantified
0.96464985	0.92767276	Quantified
1.02942362	1.09865656	Quantified
1.02635762	0.94932686	Quantified
1.06209467	1.05725845	Quantified
1.02645619	1.07146702	Quantified
1.00799276	0.983796	Quantified
0.92273702	0.85908047	Quantified
0.99283396	0.95041103	Quantified
1.11055926	1.12492026	Quantified
0.98178901	0.95529212	Quantified
0.93979957	0.99434999	Quantified
0.89036642	0.93676959	Quantified
1.03855125	1.0052803	Quantified
1.00418858	0.88500121	Quantified
0.91898232	0.93206129	Quantified
0.98119729	1.00019078	Quantified
1.00712519	0.95410175	Quantified
0.87343192	0.84315986	Quantified
0.93727811	0.92393627	Quantified
1.09939017	1.09476793	Quantified
0.99641608	0.980032	Quantified
0.90446052	0.97758375	Quantified
0.94346282	0.95514696	Quantified
0.96343967	0.91602166	Quantified
1.1195103	1.0356583	Quantified
1.1525156	1.09416504	Quantified
0.9661362	0.9493607	Quantified
0.92514781	0.82450672	Quantified
0.77010477	0.82215405	Quantified
0.87725131	0.94505924	Quantified
1.00733177	1.02300412	Quantified
1.04099681	1.00713167	Quantified
0.90234985	1.02834763	Quantified
1.0269852	1.05544817	Quantified
0.9385683	0.97049829	Quantified
0.98283211	1.06941217	Quantified
0.93459614	0.91175809	Quantified
1.02728956	0.91660019	Quantified
0.72335816	0.73631282	Quantified
1.01456633	1.00523517	Quantified
1.10070443	1.05719506	Quantified
1.00174228	0.98751392	Quantified
1.07917005	1.12266668	Quantified

0.93910918	0.93298457	Quantified
0.97992037	0.99595686	Quantified
1.00567672	0.99005962	Quantified
1.004815	1.00226333	Quantified
0.9731578	0.93843962	Quantified
0.83746027	0.83201781	Quantified
0.98970287	0.93714236	Quantified
0.9741644	0.99873818	Quantified
1.07731904	1.04479435	Quantified
1.06121464	1.04395905	Quantified
1.00911837	0.94777637	Quantified
0.99785426	0.95557737	Quantified
0.58980063	0.59745275	Quantified
		Identified
0.96421344	0.90812661	Quantified
0.92629354	0.95340963	Quantified
1.11420693	1.21741783	Quantified
1.07417036	1.01287454	Quantified
1.04706414	1.01168685	Quantified
0.81119625	0.7915025	Quantified
0.9885521	0.98527425	Quantified
1.08514554	1.05130626	Quantified
1.11657798	1.05721595	Quantified
0.98725206	0.95695882	Quantified
1.11193255	1.01564743	Quantified
0.91201031	0.844693	Quantified
0.98051638	0.83803456	Quantified
1.01743936	1.05168604	Quantified
0.95693647	0.94124032	Quantified
1.11990515	1.12265186	Quantified
0.98397711	0.97692247	Quantified
1.10448645	1.12989275	Quantified
0.88991304	0.90979693	Quantified
0.9977414	0.95993932	Quantified
0.99821183	0.99110666	Quantified
0.63527124	0.61221922	Quantified
1.05377066	1.08445988	Quantified
1.14847143	1.09954668	Quantified
0.90440877	0.88190023	Quantified
1.08731786	1.05006794	Quantified
1.03228429	1.00715779	Quantified
0.94123865	0.97830284	Quantified
1.03065612	1.06077788	Quantified
1.02745114	0.95831293	Quantified
0.93372382	0.97417821	Quantified
1.06465513	1.0016194	Quantified
1.1260914	1.08213818	Quantified
1.03260425	1.04745141	Quantified
0.97391792	1.0032167	Quantified
1.01403633	1.10344873	Quantified

0.97919993	1.002816	Quantified
1.09231426	1.01694815	Quantified
1.06516052	1.06356404	Quantified
0.87998722	0.9735885	Quantified
1.01440101	0.99854492	Quantified
0.97803225	0.98843893	Quantified
0.89180987	0.83592652	Quantified
1.16124143	1.09368093	Quantified
1.10741354	1.09261324	Quantified
0.94026514	0.92343161	Quantified
1.00215016	1.03784715	Quantified
0.94251707	0.90789886	Quantified
0.93598784	1.03307181	Quantified
0.83945472	0.88514431	Quantified
1.07796224	1.04946709	Quantified
1.1371465	1.16493585	Quantified
1.04745868	1.03323308	Quantified
0.9728276	0.9651954	Quantified
0.95654027	0.99921135	Quantified
1.04653755	1.02113264	Quantified
1.02380608	1.00320252	Quantified
1.08475406	1.08714009	Quantified
1.04961048	1.11297549	Quantified
0.99504572	1.02422433	Quantified
1.01876578	1.10965344	Quantified
1.26054046	1.15952068	Quantified
1.02178957	1.02898788	Quantified
0.95428273	0.95918404	Quantified
0.99794105	0.99203348	Quantified
0.98356919	1.00579659	Quantified
0.87710579	0.87476889	Quantified
1.08361695	1.07535159	Quantified
1.11243772	1.17516909	Quantified
0.9815602	1.03825883	Quantified
1.03192467	1.01520113	Quantified
0.96016957	0.95393674	Quantified
1.21836435	1.27432053	Quantified
1.00163058	0.95344568	Quantified
1.03371883	0.98334259	Quantified
0.97037569	0.96212572	Quantified
1.19139083	1.12542395	Quantified
0.88487617	0.92130187	Quantified
0.94644436	0.92582579	Quantified
1.06005171	1.05125483	Quantified
1.07232602	1.07454977	Quantified
1.11851228	1.15870614	Quantified
1.04372467	1.06245997	Quantified
0.99550219	1.0030471	Quantified
0.98508472	0.9711036	Quantified
1.09031019	1.08537423	Quantified

0.78717082	0.69481669	Quantified
1.1031107	1.19778888	Quantified
0.88093482	0.86014284	Quantified
0.92682634	0.86711148	Quantified
0.9717533	0.9568479	Quantified
0.99626206	1.0286979	Quantified
1.06975896	1.06228134	Quantified
0.92332728	0.87903101	Quantified
0.99254866	0.99636379	Quantified
1.13734248	1.14274763	Quantified
0.92983335	0.9473194	Quantified
1.00921276	0.95461875	Quantified
1.08092021	1.0834484	Quantified
0.97686742	0.96188641	Quantified
1.09665827	1.07991313	Quantified
1.17347131	1.1023879	Quantified
0.95993682	0.95768862	Quantified
0.85403716	0.91231616	Quantified
0.95107888	0.88727989	Quantified
1.07506213	1.05744029	Quantified
0.98062216	1.02430894	Quantified
0.92292306	0.83361695	Quantified
1.03077812	0.97256972	Quantified
1.03848578	0.9962658	Quantified
0.99049333	0.93988111	Quantified
1.00102725	1.00765505	Quantified
1.00031017	0.92777892	Quantified
0.96949558	1.04898464	Quantified
1.00170634	0.9346316	Quantified
1.02020015	1.02851934	Quantified
0.96270275	1.0284045	Quantified
1.00754201	1.04495563	Quantified
1.09968833	1.02475824	Quantified
0.67164327	0.61741852	Quantified
0.95583139	0.90051705	Quantified
1.25446846	1.33055202	Quantified
1.0448047	1.07482317	Quantified
0.83079533	0.63954689	Quantified
0.8387809	0.8062362	Quantified
1.03750401	1.01572761	Quantified
0.98942332	0.9757202	Quantified
0.88423542	0.73681899	Quantified
0.97170276	0.99752971	Quantified
0.89345396	0.91115134	Quantified
0.98782806	1.08860108	Quantified
0.83150824	0.81170221	Quantified
0.98078478	1.00211104	Quantified
1.10034492	1.03311486	Quantified
1.01782505	1.03039596	Quantified
0.88020346	0.85125304	Quantified

1.08215412	1.08162536	Quantified
1.10255633	1.03733994	Quantified
0.94996594	0.97219209	Quantified
0.97858238	0.97619769	Quantified
1.00802778	1.00826317	Quantified
1.01399721	1.00639527	Quantified
0.98309481	1.0094526	Quantified
0.93993124	0.97987071	Quantified
0.9983316	1.01474783	Quantified
0.98904142	0.96448652	Quantified
1.00199407	1.03008231	Quantified
0.93171253	0.90977881	Quantified
1.04214478	1.17531567	Quantified
1.01298263	1.06722517	Quantified
0.95028583	1.06780358	Quantified
0.9809652	1.0420355	Quantified
0.93309699	0.90273647	Quantified
0.96538707	0.91304304	Quantified
0.96764187	0.94956358	Quantified
0.9907044	1.13864585	Quantified
0.93475711	0.94747198	Quantified
1.0126303	0.97452199	Quantified
1.02176759	1.02222702	Quantified
1.10604808	1.12594152	Quantified
1.0247232	1.00678883	Quantified
0.94286366	0.95427697	Quantified
0.83703753	0.87740272	Quantified
1.05185845	1.02800666	Quantified
1.11585264	1.02644386	Quantified
0.98138615	0.93044765	Quantified
1.03519082	0.94562374	Quantified
0.85277649	0.85129497	Quantified
1.10494337	1.12311708	Quantified
0.99942563	0.98512344	Quantified
1.08254439	1.06769235	Quantified
0.84536088	0.68802792	Quantified
1.02170952	0.99822492	Quantified
1.15667711	1.11134927	Quantified
1.12959822	1.19973958	Quantified
1.06418379	1.06985291	Quantified
1.14188608	1.13318862	Quantified
1.02001232	1.0491001	Quantified
0.92740136	0.88463458	Quantified
1.13362889	1.17182001	Quantified
1.07763443	1.06138519	Quantified
1.04098764	1.13633806	Quantified
1.01521439	1.02251248	Quantified
1.07964168	1.03749038	Quantified
0.79015459	0.81447182	Quantified
0.86873792	0.87233795	Quantified

0.94560127	0.99470671	Quantified
0.84363452	0.82533485	Quantified
1.0230205	1.05673333	Quantified
1.02185436	1.08263357	Quantified
1.16484539	1.22868398	Quantified
0.88427497	0.87401563	Quantified
0.99626445	0.92060472	Quantified
1.00533176	1.04134113	Quantified
1.15018627	1.1982749	Quantified
1.08702595	1.04818965	Quantified
1.01715532	1.05703823	Quantified
1.11369412	1.09475905	Quantified
0.90884096	0.8516271	Quantified
0.98382221	0.97253782	Quantified
0.94722534	0.92293301	Quantified
1.02484238	1.03448427	Quantified
0.97962237	0.97641219	Quantified
1.09807664	1.05488072	Quantified
0.97976607	0.95003259	Quantified
1.03043948	1.01101698	Quantified
0.99209343	0.96767135	Quantified
1.04126759	1.05213142	Quantified
0.89484971	0.83831292	Quantified
0.90961245	1.05013056	Quantified
1.00510512	1.02019758	Quantified
0.99693391	1.07247078	Quantified
1.06772485	1.12877837	Quantified
0.79372587	0.83332953	Quantified
1.13704983	1.08348823	Quantified
1.0934889	1.10870205	Quantified
0.82282851	0.63427129	Quantified
1.01661112	0.97884396	Quantified
0.99192551	1.00331405	Quantified
0.97047726	1.01421096	Quantified
1.02290467	1.05823076	Quantified
1.03287099	1.00873066	Quantified
0.99895044	1.02173349	Quantified
0.99864377	0.99809119	Quantified
1.01955626	0.97125098	Quantified
1.02138581	0.98211158	Quantified
0.95867579	0.92510595	Quantified
1.08561333	1.11087532	Quantified
1.06680475	1.1154301	Quantified
0.91330731	0.90829423	Quantified
0.99728827	0.97647153	Quantified
1.00005693	1.0876044	Quantified
0.93661027	0.94324375	Quantified
0.70675446	0.59338517	Quantified
0.99182765	0.9841178	Quantified
0.89629359	0.90844778	Quantified

0.97039131	0.99327104	Quantified
0.99059887	1.08945683	Quantified
1.02700342	0.96930239	Quantified
0.9847166	1.00852484	Quantified
1.04008707	1.00404329	Quantified
1.15587168	1.05888884	Quantified
0.47431265	0.48711737	Quantified
0.95929482	0.92785853	Quantified
1.15203939	1.10678631	Quantified
0.99504777	0.9978407	Quantified
1.15386682	1.12904473	Quantified
0.95660045	0.90649118	Quantified
1.02979688	1.04375613	Quantified
0.9406306	0.90597616	Quantified
0.99823074	1.02987101	Quantified
0.95030384	0.98962075	Quantified
0.97708008	0.9809122	Quantified
1.18148207	1.20776863	Quantified
0.64726074	0.54687237	Quantified
1.08235047	1.09769161	Quantified
0.98435206	0.99389738	Quantified
0.97319043	0.9321358	Quantified
1.12122426	1.12342727	Quantified
1.04800184	1.10777281	Quantified
1.01908206	0.98943596	Quantified
1.03115605	0.99870064	Quantified
1.05679948	1.07894408	Quantified
1.06099945	1.08809056	Quantified
0.98835236	1.00592436	Quantified
0.74498367	0.52230516	Quantified
1.0285355	1.1090898	Quantified
1.0531913	1.07795611	Quantified
1.04250493	0.95629414	Quantified
1.00458323	1.00787149	Quantified
1.06221065	1.06880727	Quantified
1.08326732	1.12844777	Quantified
1.06896734	1.08151695	Quantified
0.96843462	1.04057121	Quantified
1.00124453	1.04366758	Quantified
0.9347055	0.95778991	Quantified
1.07708101	1.11946917	Quantified
0.9485832	0.88151036	Quantified
1.01298383	1.03483157	Quantified
0.62861073	0.6076081	Quantified
0.9814124	0.94691283	Quantified
1.02074112	1.02376725	Quantified
1.07785148	1.10334339	Quantified
0.88070562	0.78804209	Quantified
0.95626567	0.95558511	Quantified
0.73168633	0.7388336	Quantified

1.05515311	1.00748637	Quantified
1.12619362	1.11572499	Quantified
1.12960903	1.14582049	Quantified
1.05998973	1.00502542	Quantified
1.00019526	1.0689231	Quantified
0.92581457	0.99795174	Quantified
0.92226406	0.9066055	Quantified
0.92843057	0.9150222	Quantified
1.17537972	1.10109372	Quantified
1.1374094	1.15155986	Quantified
0.99260015	1.00714387	Quantified
0.98384778	0.9807059	Quantified
0.98397465	0.99805565	Quantified
0.65577248	0.6365521	Quantified
1.14482691	1.13227787	Quantified
0.8969647	0.82586429	Quantified
0.98370975	0.98970105	Quantified
1.37411943	1.16697702	Quantified
1.25538379	1.42765194	Quantified
1.01821471	0.99699945	Quantified
1.00468689	1.01964067	Quantified
0.9484888	0.98332017	Quantified
1.08383345	1.13938116	Quantified
0.97284158	0.96211865	Quantified
0.99343063	1.01192037	Quantified
0.74652972	0.75543152	Quantified
1.01483384	1.06723956	Quantified
0.89501315	0.90130918	Quantified
0.99995001	0.99794554	Quantified
1.06746459	0.99394336	Quantified
0.95187699	0.96548075	Quantified
1.1909632	1.06740102	Quantified
1.00656614	1.02090953	Quantified
0.98623607	0.98363155	Quantified
0.9995341	0.98353091	Quantified
1.08606889	1.04949615	Quantified
0.97972593	0.9727738	Quantified
1.0468424	1.04766462	Quantified
1.11372852	1.12290506	Quantified
0.99099814	0.98027722	Quantified
1.00261272	1.07397181	Quantified
0.66341908	0.63234046	Quantified
0.94438511	0.93720476	Quantified
0.75710508	0.72978249	Quantified
0.60927309	0.57579527	Quantified
1.00151197	1.14362715	Quantified
1.06373369	1.10274447	Quantified
1.00068795	0.94255589	Quantified
0.9598514	0.9238475	Quantified
0.96750793	0.96805609	Quantified

1.06437296	1.06288364	Quantified
1.06443891	1.08736448	Quantified
0.88003004	1.06296845	Quantified
0.99571506	1.00708742	Quantified
0.96082355	0.93807288	Quantified
0.70904868	0.72644123	Quantified
0.82574589	0.89783472	Quantified
0.92429817	0.88803469	Quantified
0.82507413	0.79714247	Quantified
1.04257034	1.07818308	Quantified
1.09305447	1.14082584	Quantified
1.01437399	0.99550327	Quantified
0.95326162	0.86587431	Quantified
0.99333454	0.99181153	Quantified
0.99501476	0.98940326	Quantified
0.99215706	1.02273453	Quantified
0.82479192	1.26476396	Quantified
1.0107463	1.01387637	Quantified
0.92232253	0.91063142	Quantified
1.16984425	1.23677579	Quantified
0.91904168	0.86279101	Quantified
1.13116485	1.17346972	Quantified
0.92014279	0.86813502	Quantified
1.04003641	1.00423615	Quantified
0.91032136	0.89076752	Quantified
0.95063146	0.91644427	Quantified
0.98795202	0.97495199	Quantified
1.05457559	0.96131977	Quantified
1.0518038	1.03245749	Quantified
1.09590212	1.08785458	Quantified
1.04399219	1.09266124	Quantified
1.03998905	1.09277051	Quantified
1.29140456	1.11653246	Quantified
1.02793042	1.03868844	Quantified
1.05600459	1.06139448	Quantified
0.96300314	0.99635731	Quantified
0.9734892	0.95855773	Quantified
0.99836504	0.99077281	Quantified
1.03495758	1.03015905	Quantified
1.11790931	1.0958371	Quantified
0.77728966	0.62715142	Quantified
1.11660182	1.14392386	Quantified
1.09940609	1.03251606	Quantified
1.08978967	1.0939094	Quantified
0.92005998	0.95956539	Quantified
0.98319919	1.01663784	Quantified
1.03760104	1.0440253	Quantified
0.93423539	0.95731954	Quantified
1.03446754	1.05452552	Quantified
0.85335928	0.84799897	Quantified

0.96437052	1.1665453	Quantified
1.06215987	1.06324141	Quantified
1.05356616	0.93256782	Quantified
0.95225497	0.93709414	Quantified
0.98757501	1.01797013	Quantified
1.14955185	1.06850308	Quantified
1.01127116	0.98388937	Quantified
1.22438358	1.20189512	Quantified
0.92265916	0.91677315	Quantified
1.03737499	1.04118676	Quantified
1.13368212	1.17176579	Quantified
1.15167142	1.00616621	Quantified
0.98734535	1.19213824	Quantified
1.05062217	1.03154336	Quantified
1.02535465	1.03400753	Quantified
0.98279659	0.95784792	Quantified
0.8048589	0.7834306	Quantified
1.06095923	1.07684827	Quantified
1.01677324	1.02785548	Quantified
0.95287532	0.87388489	Quantified
1.05025602	1.04536419	Quantified
1.10295625	1.08753579	Quantified
0.95365914	0.97167897	Quantified
0.90580882	0.88101464	Quantified
1.08255572	1.06694039	Quantified
0.8274615	0.85480889	Quantified
0.88750766	1.00232378	Quantified
1.0270099	1.10583688	Quantified
0.98377854	0.99265159	Quantified
1.0087625	1.01220038	Quantified
0.90150267	0.96538908	Quantified
0.92391108	0.92335251	Quantified
1.00464554	0.99845195	Quantified
1.05813592	1.03257001	Quantified
0.89056425	0.95477966	Quantified
0.87813065	0.92686028	Quantified
1.09607986	1.08209031	Quantified
1.04268942	1.00314791	Quantified
0.84946913	0.84815924	Quantified
1.00246266	0.98587699	Quantified
1.05649681	0.99341591	Quantified
0.52388074	0.47557603	Quantified
0.96790904	0.90054708	Quantified
0.99731406	1.12102645	Quantified
0.9899777	0.9709622	Quantified
0.97726853	0.90070826	Quantified
0.94179669	0.92447574	Quantified
0.9918255	0.97967582	Quantified
0.91804558	0.75296282	Quantified
0.97685249	0.98926546	Quantified

0.96492639	1.01004301	Quantified
1.00144512	1.18036051	Quantified
0.97974973	1.00514495	Quantified
1.06385086	1.05370881	Quantified
1.14021933	1.14136038	Quantified
0.96630373	1.02310686	Quantified
0.99564931	1.06497302	Quantified
0.99960616	0.95540186	Quantified
1.07863751	1.06102138	Quantified
0.83821691	0.77472504	Quantified
1.02356922	1.01901821	Quantified
0.974876	0.96037889	Quantified
0.94878893	1.00609549	Quantified
1.05594098	1.07062826	Quantified
0.96199066	0.98575382	Quantified
1.0990147	1.0954114	Quantified
0.99382291	1.04717541	Quantified
0.97988685	0.99453498	Quantified
1.1060618	1.12118213	Quantified
1.03036616	1.04125031	Quantified
0.98134853	1.02525911	Quantified
1.23408802	1.11158391	Quantified
1.0115987	1.04024431	Quantified
1.17709877	1.17433655	Quantified
1.02894562	1.05160187	Quantified
0.98652667	0.96776606	Quantified
0.94564555	0.93686056	Quantified
1.02363761	0.99157424	Quantified
0.92536897	0.89271448	Quantified
1.01087966	0.99435203	Quantified
1.0188136	0.98102617	Quantified
0.9746449	0.93928719	Quantified
1.10183321	1.14603399	Quantified
0.96356572	0.95882523	Quantified
1.17381922	1.1341573	Quantified
1.10426785	1.04291735	Quantified
0.96215598	0.96632409	Quantified
1.00285082	0.97536881	Quantified
1.06057664	1.08112798	Quantified
0.95583822	1.02239362	Quantified
0.99934302	0.93002459	Quantified
0.93961086	0.94551098	Quantified
0.93991709	0.90602714	Quantified
1.01570471	1.01930124	Quantified
1.00746162	0.99724937	Quantified
0.99654657	0.97879179	Quantified
0.94298425	1.02858768	Quantified
1.01812575	1.02076509	Quantified
1.00711926	0.96379261	Quantified
0.94657285	1.02075121	Quantified

1.03877472	0.99815789	Quantified
0.99939632	1.00132548	Quantified
0.95522278	0.96214257	Quantified
0.95597643	0.9435428	Quantified
1.09027883	1.02966728	Quantified
0.93470146	0.93475993	Quantified
1.09789093	1.07152389	Quantified
0.98674442	1.01469773	Quantified
1.12581132	1.12380035	Quantified
0.95468357	1.0366143	Quantified
0.9599492	1.03573538	Quantified
1.09981293	1.0720318	Quantified
1.00239667	1.06224504	Quantified
1.01316123	1.03924339	Quantified
0.94035636	0.99659646	Quantified
1.02024577	1.02587116	Quantified
0.76274868	0.63371263	Quantified
0.90763872	0.82419497	Quantified
1.0947169	1.12729812	Quantified
0.98365992	0.97687848	Quantified
1.00377519	1.02843334	Quantified
0.93247268	0.87816191	Quantified
1.02526781	1.02647253	Quantified
0.96802369	0.93421743	Quantified
1.12632244	1.17694738	Quantified
1.1851189	1.27113484	Quantified
0.9100534	0.93894241	Quantified
1.04311277	1.05611483	Quantified
1.00856298	1.02352891	Quantified
1.03607402	0.97279132	Quantified
1.0285592	1.00803073	Quantified
1.04138413	0.9604843	Quantified
0.95067488	0.93858199	Quantified
1.01550644	1.11450169	Quantified
0.96789459	1.03033385	Quantified
1.02701867	0.99789294	Quantified
0.98508162	1.00416082	Quantified
1.03693135	1.09546993	Quantified
0.89929447	0.88237695	Quantified
0.68434657	0.64120202	Quantified
1.00727055	1.03967118	Quantified
1.01634545	1.02800848	Quantified
1.18432409	1.17461205	Quantified
0.92463684	0.93731937	Quantified
1.05031685	1.01348138	Quantified
0.95636712	1.00609487	Quantified
1.02792343	0.94814581	Quantified
0.83606511	0.83928172	Quantified
1.0532031	1.0326729	Quantified
1.09467103	0.81349162	Quantified

1.08664107	1.0958182	Quantified
0.96071473	0.94059211	Quantified
0.93040464	0.95505674	Quantified
0.87292635	0.87433634	Quantified
0.95446457	0.90892669	Quantified
0.95219161	0.97143945	Quantified
1.1003373	1.03629678	Quantified
0.95476456	0.99788131	Quantified
0.9071424	1.02087997	Quantified
1.00508178	1.03620156	Quantified
1.06512345	1.07924114	Quantified
1.0764938	1.01996813	Quantified
1.10994404	1.11836571	Quantified
0.93241617	1.03231502	Quantified
1.00145302	1.05327298	Quantified
0.9642862	0.98029144	Quantified
0.87421354	0.81952911	Quantified
		Identified
1.06436889	0.99776111	Quantified
0.95818462	0.93278564	Quantified
1.05280685	1.05534572	Quantified
0.98394419	0.98284371	Quantified
0.84190499	0.85972964	Quantified
0.99230863	1.02365485	Quantified
0.98490242	0.98647546	Quantified
1.04800762	1.18760163	Quantified
0.94428542	0.95375596	Quantified
0.99885227	0.98395113	Quantified
1.14010431	1.03600692	Quantified
1.01321465	0.96234328	Quantified
1.00299837	1.07109361	Quantified
0.93235166	0.91255022	Quantified
1.00969452	0.97710771	Quantified
1.03193455	1.04677033	Quantified
1.0286665	1.10199577	Quantified
0.97233982	1.0837359	Quantified
1.03638487	0.98767046	Quantified
1.04466252	1.00870963	Quantified
0.9755911	1.03337568	Quantified
0.86706912	1.00768112	Quantified
0.98405343	0.95057888	Quantified
1.00027145	1.10517673	Quantified
1.10541376	1.06745899	Quantified
0.99063416	1.00292238	Quantified
1.45804787	1.45419161	Quantified
1.01380208	1.07933563	Quantified
1.09011331	1.02800583	Quantified
1.05320286	1.03082635	Quantified
0.98681332	1.05002734	Quantified
1.07400696	1.19117643	Quantified

0.96850587	1.06799348	Quantified
0.97953811	0.95552842	Quantified
0.86627948	0.85763604	Quantified
1.07451654	1.07597414	Quantified
0.8480351	0.85087123	Quantified
1.01184757	0.99588578	Quantified
0.98258039	1.10198075	Quantified
0.99766091	0.99365568	Quantified
1.00454118	1.02564731	Quantified
1.03349542	1.09606402	Quantified
1.07580212	0.94023162	Quantified
0.9612782	0.95370923	Quantified
0.69639723	0.71954433	Quantified
1.12357811	1.15385551	Quantified
1.03814236	1.0793534	Quantified
1.01394741	1.0102992	Quantified
0.9188183	0.85474216	Quantified
0.94158937	1.40838622	Quantified
0.74155281	0.69317284	Quantified
1.03916041	0.97085408	Quantified
0.82878565	0.90903066	Quantified
0.97963646	1.07768205	Quantified
1.33675464	1.33327827	Quantified
1.31264967	1.28008919	Quantified
1.07419707	1.02070727	Quantified
1.16129659	1.16525774	Quantified
0.90022834	0.76999775	Quantified
0.83151574	0.85175201	Quantified
0.74231963	0.72709068	Quantified
1.04325489	1.06026573	Quantified
0.93166584	1.01275412	Quantified
1.08803402	0.80365254	Quantified
0.99421229	0.92652262	Quantified
1.02075232	1.03789073	Quantified
1.08317693	1.04077895	Quantified
0.93665999	0.90260931	Quantified
0.97230379	0.99694172	Quantified
0.94277964	0.95482564	Quantified
1.0170012	1.02666084	Quantified
0.9168252	0.94416415	Quantified
1.04295678	1.02275012	Quantified
0.91483708	0.95573184	Quantified
0.83190964	0.79506455	Quantified
0.90899124	0.80927204	Quantified
1.0188432	1.00635638	Quantified
1.11562537	1.10702893	Quantified
1.111934	1.08719881	Quantified
1.41055593	1.21575093	Quantified
1.01010702	0.98506224	Quantified
0.98970008	0.98075855	Quantified

0.93732843	0.92769422	Quantified
1.00404237	0.96479445	Quantified
1.01623534	1.0471489	Quantified
1.02592497	1.01428234	Quantified
0.86419573	0.8877052	Quantified
1.11593338	1.21231215	Quantified
1.00085347	0.95786679	Quantified
1.06420777	1.06266936	Quantified
1.03854666	1.00254713	Quantified
1.05662905	1.01471238	Quantified
1.04184341	0.84128642	Quantified
1.06560967	1.07170222	Quantified
1.0624652	1.06860362	Quantified
1.21880457	1.30554167	Quantified
0.9913583	0.82876198	Quantified
1.03656974	0.99506582	Quantified
0.86008774	0.65703802	Quantified
0.90184561	0.85875385	Quantified
0.93168441	0.97020633	Quantified
0.89732245	0.79167219	Quantified
0.96128498	1.04114245	Quantified
1.04901633	1.01991029	Quantified
0.94019412	0.91760586	Quantified
1.06892912	1.09093108	Quantified
1.04264398	1.02000167	Quantified
1.23396966	1.31389907	Quantified
1.16447373	1.18427593	Quantified
0.95278773	0.95582549	Quantified
1.03625411	0.9939775	Quantified
1.0827292	1.1077947	Quantified
1.05108346	0.99457073	Quantified
1.02022334	1.06553054	Quantified
1.11829982	1.11969712	Quantified
0.96481717	0.98864125	Quantified
1.07165804	1.07571362	Quantified
0.9764545	0.95992585	Quantified
0.9586037	1.00247323	Quantified
0.95736641	1.02593365	Quantified
0.92752087	0.92299279	Quantified
1.04149193	1.06636358	Quantified
1.27193724	1.22772101	Quantified
0.90552325	1.07427522	Quantified
0.96072218	1.01493915	Quantified
1.14835132	1.12117464	Quantified
1.01242687	0.99691814	Quantified
0.8675288	0.90743491	Quantified
0.93908682	0.86713591	Quantified
0.7518841	0.69631018	Quantified
1.00200376	1.07232783	Quantified
1.05009732	1.05999706	Quantified

0.93790163	0.89739035	Quantified
1.03016342	1.04684333	Quantified
0.94859186	0.96090233	Quantified
1.01881559	0.94718213	Quantified
1.1043978	1.07172547	Quantified
1.02750921	1.09128343	Quantified
1.06005281	1.07537438	Quantified
1.07497082	1.10055749	Quantified
0.90324098	0.89512876	Quantified
0.97235326	1.03532997	Quantified
1.04047016	1.02594808	Quantified
0.9645222	0.93180874	Quantified
1.02852186	1.00053646	Quantified
1.04310548	1.03230268	Quantified
1.00647574	0.94439015	Quantified
1.01706989	0.97122693	Quantified
1.05768195	1.02027988	Quantified
1.01499195	0.95422123	Quantified
0.97687398	1.0084128	Quantified
1.1121571	1.13802381	Quantified
0.96853903	0.93334762	Quantified
0.98634295	1.00295163	Quantified
0.91950266	0.88905774	Quantified
0.92542387	0.86076058	Quantified
0.88830013	0.75768521	Quantified
0.85477302	0.84663535	Quantified
0.96756299	0.91881129	Quantified
1.10642664	1.14807599	Quantified
0.92390823	0.9281088	Quantified
0.89250828	0.89222869	Quantified
0.98394813	0.83803815	Quantified
1.07386719	1.06506342	Quantified
0.97727371	0.96693796	Quantified
1.07959943	1.13202343	Quantified
1.0098953	0.97859514	Quantified
1.00806521	0.99958203	Quantified
1.06957315	1.131447	Quantified
0.98314367	1.01663691	Quantified
0.93833734	0.87865675	Quantified
0.92927623	0.91797257	Quantified
0.80596654	0.68168122	Quantified
0.97899632	0.92419497	Quantified
1.03531535	1.04576082	Quantified
0.5743159	0.48067509	Quantified
1.00757467	0.95117331	Quantified
1.16675971	1.24884525	Quantified
0.8571273	0.85206016	Quantified
1.23247929	1.21495227	Quantified
0.9251617	0.90995167	Quantified
1.05849689	1.05371435	Quantified

0.99825591	0.99489889	Quantified
1.05738827	1.08031871	Quantified
0.8332886	0.84164743	Quantified
1.06192876	1.07857954	Quantified
1.0418679	1.06932222	Quantified
1.0069775	0.95773695	Quantified
0.79491732	0.80506701	Quantified
0.90216176	0.90533165	Quantified
1.00049227	0.97238967	Quantified
1.00313537	1.06477746	Quantified
1.04247936	1.00985726	Quantified
0.97243504	0.99113596	Quantified
0.97637916	0.94729272	Quantified
0.99519771	0.8850918	Quantified
1.13662126	1.07720112	Quantified
0.91709941	0.87904325	Quantified
1.09489588	1.08667392	Quantified
0.96028537	0.96527327	Quantified
0.89633647	0.83513741	Quantified
0.94161642	0.91391752	Quantified
0.93511324	1.00463345	Quantified
0.99468796	1.04075836	Quantified
0.96413457	0.97607713	Quantified
0.96791014	0.94124691	Quantified
1.17208127	1.04649207	Quantified
1.06007511	1.07365375	Quantified
0.75245901	0.74009112	Quantified
1.04555596	1.14410463	Quantified
1.02862965	1.0381708	Quantified
1.05467598	1.04466764	Quantified
0.96666794	1.00215131	Quantified
1.02127314	1.0129902	Quantified
1.0199179	1.01426508	Quantified
1.05647103	1.1980292	Quantified
1.0047258	0.9953565	Quantified
1.10958357	1.08129863	Quantified
1.02919639	1.03622893	Quantified
0.93795326	0.75311537	Quantified
0.90095397	0.96935332	Quantified
1.17319874	1.20910515	Quantified
1.06187244	0.99161232	Quantified
0.86151737	0.85584979	Quantified
1.1390879	1.0617984	Quantified
0.93901294	0.90684754	Quantified
0.90624683	0.92485497	Quantified
1.02395335	1.08328137	Quantified
1.0718183	1.0854034	Quantified
1.03886985	1.02151642	Quantified
1.06801128	0.99656153	Quantified
1.11087908	1.3277141	Quantified

0.80270214	0.80953393	Quantified
0.93076381	0.96317637	Quantified
1.08235986	1.08346129	Quantified
0.86809602	0.86953657	Quantified
0.97127282	1.02555073	Quantified
1.06038283	1.14449122	Quantified
1.01016238	0.95606511	Quantified
1.01218029	0.99204613	Quantified
0.91468609	0.93616775	Quantified
0.70620984	0.62569074	Quantified
0.98375028	0.8869714	Quantified
0.90429773	0.89477515	Quantified
0.95948565	0.98081572	Quantified
0.84057844	0.84497167	Quantified
1.0490615	1.06116356	Quantified
0.98080297	1.04350584	Quantified
1.03247967	1.04125092	Quantified
1.00060704	1.04626377	Quantified
1.11743599	1.08378412	Quantified
1.06088465	1.08028877	Quantified
0.85604487	0.86018922	Quantified
0.89371757	0.85665856	Quantified
1.02207849	1.00885866	Quantified
1.07389198	1.03339594	Quantified
1.08100746	1.06951934	Quantified
0.94829111	0.95693845	Quantified
0.97055684	0.94836781	Quantified
0.98440822	1.03111575	Quantified
1.10698518	1.04440846	Quantified
0.98371935	0.99041891	Quantified
0.97957724	0.97247079	Quantified
1.08995438	1.04380987	Quantified
0.82573129	0.85256459	Quantified
0.86901555	1.04579986	Quantified
1.07576157	1.08411292	Quantified
1.11388917	1.13147052	Quantified
1.05755119	1.03470117	Quantified
0.99139629	1.00560309	Quantified
1.14467349	1.17810628	Quantified
0.96231441	1.02097535	Quantified
0.76261182	0.80241898	Quantified
0.98122324	0.97094941	Quantified
1.01369583	0.99192488	Quantified
1.0234756	1.01069141	Quantified
1.0755688	1.00836715	Quantified
1.05390583	1.04653138	Quantified
1.0432655	1.0176926	Quantified
0.90019255	1.09865973	Quantified
1.02504322	1.13627915	Quantified
0.87930436	0.97589715	Quantified

0.96362461	0.97163242	Quantified
1.06222747	1.02094961	Quantified
0.84613132	0.72965582	Quantified
1.01416966	1.00148079	Quantified
1.07853119	1.08255273	Quantified
0.9364703	0.90534475	Quantified
1.03908096	1.09210115	Quantified
1.10862079	1.14826308	Quantified
0.97178828	0.98594304	Quantified
0.99397965	1.02668668	Quantified
1.03250134	1.07124142	Quantified
0.95180462	0.92357932	Quantified
0.973321	0.98794847	Quantified
1.08346143	1.09809934	Quantified
1.05263827	1.01488101	Quantified
1.06924393	1.09481617	Quantified
1.19966612	1.24232771	Quantified
0.72829461	0.69775871	Quantified
1.0806061	1.14448228	Quantified
0.9854336	0.98910478	Quantified
0.95691097	0.95074842	Quantified
0.73243696	0.72066348	Quantified
1.03310205	1.09145088	Quantified
1.1444411	1.0675555	Quantified
1.02126878	1.05497824	Quantified
1.00467652	1.12770698	Quantified
0.96060833	1.01845572	Quantified
0.85864332	0.92765047	Quantified
1.21197886	1.1832388	Quantified
0.9336075	1.08172143	Quantified
1.16531184	1.12770645	Quantified
1.12725569	1.13200307	Quantified
0.95601959	1.04844574	Quantified
1.08234515	1.06476047	Quantified
0.93491621	0.943032	Quantified
1.14116813	1.12783819	Quantified
1.02309965	1.01531051	Quantified
0.69183179	0.68903035	Quantified
0.9487488	0.94966622	Quantified
0.98619187	1.01184551	Quantified
0.98521266	1.00402507	Quantified
1.00370502	0.98391587	Quantified
1.0003068	1.00956683	Quantified
1.07652049	1.1010197	Quantified
0.91672843	0.96913344	Quantified
0.8825152	0.95220714	Quantified
0.88003747	0.92736753	Quantified
0.820158	0.78788191	Quantified
1.06000823	1.08062488	Quantified
1.0316284	1.08115022	Quantified

0.89819121	0.863877	Quantified
1.03030277	1.03446004	Quantified
0.96946157	1.03256815	Quantified
0.94516299	1.02784143	Quantified
1.05183723	1.09207023	Quantified
1.01774741	1.02573507	Quantified
1.07535049	0.99868067	Quantified
0.99465264	1.14162159	Quantified
0.88722014	0.9974398	Quantified
0.96292542	1.05846289	Quantified
1.03021611	1.07220687	Quantified
0.95142312	0.8756298	Quantified
1.12029272	1.17445316	Quantified
0.99625096	1.18074228	Quantified
1.06930447	1.08990182	Quantified
1.05876228	1.01810992	Quantified
0.94092655	0.95693418	Quantified
1.02015611	0.98466839	Quantified
0.71820214	0.64617251	Quantified
0.89263024	0.89746185	Quantified
0.81417806	0.80810004	Quantified
0.98534696	0.99406604	Quantified
0.91898256	0.99723234	Quantified
0.94230182	0.92619253	Quantified
1.06199791	0.98844786	Quantified
0.93203606	0.93531255	Quantified
1.19939188	1.15001876	Quantified
0.84028163	0.77906324	Quantified
0.98604299	0.99074288	Quantified
1.04530225	1.08029719	Quantified
1.03829374	1.07416665	Quantified
0.90393403	0.93710177	Quantified
1.05831173	1.05213892	Quantified
0.97858456	0.97771658	Quantified
0.85676933	0.8320601	Quantified
1.10953322	1.01957279	Quantified
1.08824068	1.08782377	Quantified
0.99623297	0.90548188	Quantified
0.74931355	0.86998289	Quantified
0.95748807	0.92942747	Quantified
1.11003346	1.11135227	Quantified
1.04532791	1.05536105	Quantified
0.96205589	0.98818585	Quantified
1.04050564	1.10696873	Quantified
0.93033183	0.95262616	Quantified
0.86740836	0.80896959	Quantified
0.97859957	1.17007867	Quantified
0.77625139	0.73823676	Quantified
0.98366116	1.04316329	Quantified
1.00002891	1.01498381	Quantified

0.95016002	0.97777192	Quantified
1.11520771	1.05579555	Quantified
1.0499588	1.04895919	Quantified
1.07721859	1.03839616	Quantified
0.99773847	1.0345818	Quantified
0.95052117	0.8834427	Quantified
1.00700925	1.05210235	Quantified
0.92311246	0.97906786	Quantified
0.99684193	0.97364324	Quantified
1.00798665	1.07194947	Quantified
1.01751009	1.01592971	Quantified
1.08258321	0.97287351	Quantified
0.90544306	0.93455932	Quantified
1.02432218	1.01818725	Quantified
0.91822102	0.90363444	Quantified
1.06516135	1.07650336	Quantified
1.03330209	1.07150516	Quantified
0.90952635	0.93836424	Quantified
1.05172112	0.90055727	Quantified
1.13773518	1.14392398	Quantified
1.05176143	1.02764198	Quantified
0.88778123	0.99786704	Quantified
0.96393687	0.97540907	Quantified
0.96391004	0.92898842	Quantified
1.24641359	1.46192972	Quantified
0.95206575	0.89612735	Quantified
1.06726104	1.06420672	Quantified
1.39518684	1.49512315	Quantified
1.01135864	1.01165266	Quantified
1.02302557	1.02490324	Quantified
0.68601263	0.79236772	Quantified
1.18739996	1.19054283	Quantified
1.00923508	1.03525906	Quantified
1.09279007	1.13381558	Quantified
0.95305724	0.88165379	Quantified
0.78429781	0.71761434	Quantified
1.03312923	1.0999507	Quantified
1.01179778	1.09462394	Quantified
1.1291274	1.05149441	Quantified
1.0469888	1.029759	Quantified
0.97004413	1.01618152	Quantified
0.9947248	0.98454936	Quantified
0.80431774	0.75644066	Quantified
0.89825396	0.8933829	Quantified
0.98034613	0.96984475	Quantified
0.98105505	0.97339502	Quantified
0.92878009	0.97892733	Quantified
0.84431765	0.79607977	Quantified
1.00657365	1.03312091	Quantified
1.04315992	1.01979029	Quantified

1.11991913	1.05428734	Quantified
1.31697721	1.5825645	Quantified
1.00035476	0.98476707	Quantified
0.92633649	0.88509588	Quantified
0.99688476	0.95877768	Quantified
0.96439517	0.9971575	Quantified
0.97542967	1.06414365	Quantified
1.042302	1.08475745	Quantified
1.02630264	1.06484484	Quantified
1.11467276	1.10942418	Quantified
1.02946374	0.98664769	Quantified
1.10100008	1.07443209	Quantified
0.96509293	0.91000435	Quantified
0.88921626	1.13687086	Quantified
0.82403538	0.77481174	Quantified
1.02036386	1.03179763	Quantified
0.94310377	0.94046905	Quantified
0.88373904	1.03157036	Quantified
0.99847664	0.98358649	Quantified
0.93213385	0.897781	Quantified
0.71025703	0.70320449	Quantified
0.94613356	0.9538815	Quantified
0.88325039	0.9067552	Quantified
1.01245935	1.03879436	Quantified
1.05377392	0.98470977	Quantified
0.9594678	1.07300739	Quantified
0.97819679	1.00049927	Quantified
0.83843212	0.88518578	Quantified
1.24966787	1.3382433	Quantified
0.82161901	0.79313491	Quantified
0.82805766	0.86115333	Quantified
0.94421145	0.9384859	Quantified
1.04905636	1.11112859	Quantified
1.04324824	1.26271993	Quantified
1.00169953	0.967186	Quantified
0.96850651	0.92056856	Quantified
1.10181065	1.05720908	Quantified
1.05221417	1.04007352	Quantified
1.25626523	1.26623283	Quantified
1.01680194	1.20055594	Quantified
0.96673033	0.93303671	Quantified
0.94098028	0.92605797	Quantified
0.9839059	0.91355066	Quantified
0.90119027	0.96040663	Quantified
0.92712182	0.91319142	Quantified
1.10401835	1.09205421	Quantified
0.94105927	1.01260722	Quantified
1.14426593	1.0048667	Quantified
1.11869001	1.0209835	Quantified
0.75191292	0.73442143	Quantified

1.0911765	1.07877343	Quantified
0.99737054	0.91855696	Quantified
1.01145224	0.98446323	Quantified
1.05725954	1.0134831	Quantified
1.03346354	1.06067102	Quantified
0.93835652	0.91993812	Quantified
1.03197538	1.03741106	Quantified
0.8644239	0.81843444	Quantified
0.8866548	0.93292195	Quantified
0.98079884	0.9663597	Quantified
0.95045463	0.91979689	Quantified
1.00448328	0.95551727	Quantified
0.93548554	0.96385687	Quantified
0.94532031	0.95699287	Quantified
0.92600726	0.86389831	Quantified
1.17829893	1.23374527	Quantified
1.04883858	1.01305178	Quantified
1.02093278	1.0020184	Quantified
1.03920108	1.08830055	Quantified
1.08303645	1.0980033	Quantified
0.95626242	0.95451575	Quantified
1.02755158	1.09062551	Quantified
0.80255815	0.75602924	Quantified
0.92336005	0.90279318	Quantified
1.18845148	1.17182224	Quantified
1.42251237	1.29317638	Quantified
0.97800996	0.96544763	Quantified
1.04900292	0.98898669	Quantified
0.9657287	0.94562113	Quantified
1.01850824	1.10366455	Quantified
0.9831393	1.05924215	Quantified
1.0435678	1.07641487	Quantified
0.9946718	0.97820484	Quantified
0.95351133	0.91785206	Quantified
1.14887245	1.04636437	Quantified
1.04620314	1.01368225	Quantified
0.90129655	0.9229772	Quantified
1.00628887	1.01301449	Quantified
1.07035572	1.07192971	Quantified
0.88990073	0.91551081	Quantified
0.65544211	0.58154532	Quantified
0.94282769	0.90026786	Quantified
0.85661774	0.87866618	Quantified
0.95539422	0.97807839	Quantified
0.9777878	1.02235194	Quantified
0.954073	0.94650084	Quantified
0.88494786	0.89901691	Quantified
0.96180683	0.94850592	Quantified
0.98207817	0.9090074	Quantified
1.138658	1.17068186	Quantified

0.95154187	0.93591621	Quantified
1.13811213	1.16681474	Quantified
1.04532293	1.0381514	Quantified
0.94197029	0.87960195	Quantified
0.95672005	1.02026779	Quantified
0.71108632	0.70064717	Quantified
1.12229454	1.09332314	Quantified
1.16560931	1.19288488	Quantified
1.04212264	1.02476058	Quantified
1.00827741	0.80868768	Quantified
0.94636661	0.93030173	Quantified
0.89328863	0.92191778	Quantified
1.07117386	1.00672079	Quantified
1.04340217	1.08747455	Quantified
0.99988664	1.05187188	Quantified
1.01280377	1.01319743	Quantified
1.0043384	0.96896525	Quantified
0.997028	1.02564297	Quantified
0.9508953	1.03050057	Quantified
1.03904986	0.93972625	Quantified
0.87849709	0.88688508	Quantified
0.84729414	0.90157538	Quantified
1.00084021	0.97309	Quantified
0.94508921	0.89635193	Quantified
1.04620718	1.00393481	Quantified
0.87720124	0.87323665	Quantified
0.94841409	0.9936891	Quantified
0.98175174	1.00664694	Quantified
0.97804269	0.93626173	Quantified
0.91741064	0.88046692	Quantified
0.91183913	1.20354221	Quantified
1.03539951	1.03979705	Quantified
1.04197497	1.03676989	Quantified
0.81806185	0.83784282	Quantified
0.98422739	0.91197834	Quantified
0.92412823	0.91428858	Quantified
1.01318399	0.99330034	Quantified
0.8905857	0.9155451	Quantified
1.01690851	0.9746892	Quantified
0.92798795	0.94349835	Quantified
1.04468476	1.01672912	Quantified
0.93767374	0.86241478	Quantified
0.99006035	0.83242667	Quantified
0.93974495	1.02389387	Quantified
0.79133674	0.77135058	Quantified
1.0023044	0.99951271	Quantified
0.99632245	0.9387299	Quantified
0.98259033	0.9330473	Quantified
1.01582979	0.963601	Quantified
1.015777	1.0294242	Quantified

0.87437236	0.94260322	Quantified
0.5306465	0.4654501	Quantified
1.03700526	1.08662868	Quantified
0.99389086	1.01694615	Quantified
1.00280026	1.0909851	Quantified
0.83824459	0.91343387	Quantified
0.98759665	0.98931687	Quantified
1.04941958	1.01936493	Quantified
1.14210944	1.1441747	Quantified
1.13954481	1.20532985	Quantified
0.87830614	0.90645905	Quantified
0.86622971	0.84170401	Quantified
1.26304853	1.15389141	Quantified
0.66696865	0.87686922	Quantified
1.00035696	1.13759415	Quantified
0.96066223	0.94849215	Quantified
0.93408169	1.03303556	Quantified
0.85717614	0.75304826	Quantified
1.0005961	0.97955232	Quantified
1.01915739	1.06925229	Quantified
0.97489066	0.93720004	Quantified
0.93598633	0.92004482	Quantified
1.15255793	1.07173259	Quantified
0.92870947	0.91612867	Quantified
0.98200202	0.98003095	Quantified
1.06999608	1.06433053	Quantified
1.09304146	1.03559384	Quantified
1.00943839	1.05577382	Quantified
1.00368368	1.00314447	Quantified
1.07406547	1.00119357	Quantified
1.05746099	0.98550148	Quantified
0.91726832	0.9011019	Quantified
0.99790554	0.90274864	Quantified
0.98731211	1.00956281	Quantified
0.99889391	1.019274	Quantified
1.0240007	1.01215104	Quantified
1.0651871	1.06172188	Quantified
1.04131805	0.93374917	Quantified
0.99443649	1.07111663	Quantified
1.02442864	0.9861407	Quantified
1.03714556	1.04169466	Quantified
1.02422896	0.98485406	Quantified
1.03226086	0.98853753	Quantified
1.07689754	1.19293766	Quantified
0.89637603	0.91272172	Quantified
1.10885503	1.06203404	Quantified
0.88677068	0.79336958	Quantified
1.00305298	0.99352343	Quantified
0.93277711	1.00829378	Quantified
0.88275382	0.92442918	Quantified

0.9032243	0.91291116	Quantified
0.85988921	0.94400632	Quantified
1.02124492	1.07245551	Quantified
		Identified
0.85274897	0.84192395	Quantified
0.92395334	1.08321779	Quantified
1.06004766	0.94164492	Quantified
1.05041296	1.03875787	Quantified
1.1909314	1.16039292	Quantified
0.99691269	1.01819329	Quantified
0.93777497	0.90733457	Quantified
0.98244787	0.94715265	Quantified
1.01630168	1.00456184	Quantified
1.13722143	1.03937107	Quantified
0.82233333	0.9029049	Quantified
1.1375387	1.02967911	Quantified
1.08981831	1.07156125	Quantified
1.2882446	1.27596048	Quantified
0.94312828	0.9608939	Quantified
1.12104228	1.17559608	Quantified
0.9331569	0.91596373	Quantified
1.00991927	0.9514035	Quantified
0.93106355	0.92994149	Quantified
0.96364476	0.98746155	Quantified
0.83318522	0.83113562	Quantified
0.98507907	0.95635375	Quantified
1.09766376	1.08934191	Quantified
0.90744858	0.89788172	Quantified
1.00051174	1.06675154	Quantified
1.03086945	0.97168263	Quantified
1.15665544	1.21696408	Quantified
0.93818576	0.95308923	Quantified
1.04773083	1.05617984	Quantified
1.10812568	1.09154955	Quantified
1.00603031	1.02813416	Quantified
0.89427774	0.79002567	Quantified
0.90723021	1.73915573	Quantified
0.95275949	0.90610057	Quantified
0.97084732	0.97677464	Quantified
0.99855065	1.20800263	Quantified
1.09611172	1.02844976	Quantified
1.02828788	1.0017278	Quantified
1.09268811	1.17542509	Quantified
0.89446254	0.87815001	Quantified
1.08999976	1.07251401	Quantified
1.00350191	1.08741186	Quantified
0.98448861	0.94350578	Quantified
1.01906504	1.08081051	Quantified
1.00077588	0.8986665	Quantified
1.10772721	1.10394211	Quantified

0.9563278	0.90157448	Quantified
1.13518259	1.0841734	Quantified
0.9375438	0.8863301	Quantified
1.02325903	0.98896905	Quantified
0.91631611	0.8923468	Quantified
0.8476747	0.91187321	Quantified
0.82497295	0.90491609	Quantified
1.03520006	1.09188684	Quantified
0.96587923	0.98718852	Quantified
1.0150367	0.9215043	Quantified
1.09516208	1.08984378	Quantified
1.05457855	1.18913127	Quantified
1.09557177	1.09086768	Quantified
0.94995968	0.96245002	Quantified
1.21493342	1.20574605	Quantified
0.9365777	0.88640273	Quantified
1.05470114	1.05617734	Quantified
1.10601389	1.16017827	Quantified
1.04984631	1.04574394	Quantified
0.92227779	0.91657767	Quantified
0.8791226	0.83188456	Quantified
0.80176883	0.71493482	Quantified
1.04943507	1.13017393	Quantified
0.8460922	0.83643515	Quantified
1.023231	1.02882235	Quantified
1.07613494	1.07316813	Quantified
1.05087405	1.02316452	Quantified
1.26727929	1.28210812	Quantified
0.86636289	0.87627873	Quantified
1.02060697	1.00562075	Quantified
1.04896277	0.99134394	Quantified
1.0217082	1.00886327	Quantified
0.83306012	0.74690443	Quantified
0.99727327	1.12128015	Quantified
1.01214954	0.99902794	Quantified
1.05272225	1.04297878	Quantified
0.92909309	0.9175427	Quantified
1.02868675	1.09707417	Quantified
0.99101553	1.04390123	Quantified
0.90192909	0.94327526	Quantified
0.9763617	1.00735722	Quantified
0.98962075	1.01525339	Quantified
0.92791103	1.00448525	Quantified
0.92508241	0.9702221	Quantified
0.85759641	0.77275159	Quantified
0.97628757	0.94995698	Quantified
1.11523236	1.1177423	Quantified
1.06146916	1.11239096	Quantified
0.90681486	1.15144684	Quantified
1.08128635	1.05303377	Quantified

0.72890829	0.76951106	Quantified
1.04101011	1.15074815	Quantified
0.99829491	1.10121776	Quantified
0.81488467	0.82401514	Quantified
1.14747068	1.15300102	Quantified
0.91732824	0.90389297	Quantified
0.95072578	1.0750743	Quantified
0.89379692	0.96506902	Quantified
0.6550186	0.6822401	Quantified
0.90156273	0.84782155	Quantified
0.99962532	1.03201382	Quantified
1.16000256	1.16142108	Quantified
0.84069306	0.81171994	Quantified
0.96443126	1.0505241	Quantified
0.99540012	0.99352062	Quantified
0.93800118	1.03092586	Quantified
0.97880699	0.99812928	Quantified
1.11706972	1.1158689	Quantified
0.82394519	0.83170475	Quantified
1.04909739	1.05861365	Quantified
0.95374769	0.96580013	Quantified
1.16290826	1.25387455	Quantified
0.99696665	0.98590593	Quantified
1.0339992	1.0269626	Quantified
0.95074103	0.94036358	Quantified
0.98970312	0.87523266	Quantified
0.91971357	1.02067211	Quantified
0.99280627	0.89569991	Quantified
1.12609661	1.06909959	Quantified
0.81819866	0.65255789	Quantified
1.01045668	1.07625905	Quantified
0.79178735	0.78235659	Quantified
0.96827352	0.92706203	Quantified
0.99799079	1.04870421	Quantified
1.04782307	1.03785096	Quantified
0.96042271	1.01739254	Quantified
1.05940766	1.00334607	Quantified
0.84765743	0.79338893	Quantified
1.06150979	0.98847677	Quantified
0.91722434	0.85662903	Quantified
1.01013124	0.88320175	Quantified
1.07811736	1.10039205	Quantified
1.08176162	1.06540498	Quantified
0.97435425	0.81384747	Quantified
0.91203179	0.9844426	Quantified
1.19491123	1.0532024	Quantified
1.02734945	1.02720827	Quantified
0.98938636	0.98559855	Quantified
0.90700274	0.87631454	Quantified
0.86943605	0.942175	Quantified

1.08675514	1.12584291	Quantified
1.10138651	1.07580489	Quantified
1.02851655	1.02890264	Quantified
1.01471703	0.89557229	Quantified
1.1036922	1.16015559	Quantified
1.08748922	1.12620435	Quantified
0.99424443	1.08762695	Quantified
0.99647949	0.96571089	Quantified
1.10735599	1.1919521	Quantified
0.93183351	0.91000684	Quantified
0.89328002	0.90074434	Quantified
0.83264136	0.87281978	Quantified
1.0624886	1.06666131	Quantified
1.07883295	1.13945082	Quantified
0.77060155	1.1504171	Quantified
1.07466595	1.08083627	Quantified
0.87621115	0.91213532	Quantified
1.01149582	0.98660661	Quantified
1.01613813	1.05469203	Quantified
1.07336643	1.0592934	Quantified
0.82396277	0.87679465	Quantified
0.99147017	0.99703207	Quantified
0.92979186	1.00508735	Quantified
0.84127884	0.79164244	Quantified
1.44912567	1.09912744	Quantified
1.01226797	1.13021377	Quantified
0.98529326	1.03723074	Quantified
1.11102316	1.12951907	Quantified
0.82310884	0.75944877	Quantified
0.68808547	0.74714177	Quantified
1.00711595	1.07597886	Quantified
1.14694816	1.21147278	Quantified
1.14045968	1.23669698	Quantified
1.12270579	1.246656	Quantified
1.02067794	1.12065621	Quantified
0.82448328	0.75165942	Quantified
1.00620597	0.97580665	Quantified
1.03083108	1.1423738	Quantified
1.02466556	1.04218902	Quantified
0.96630166	0.87101806	Quantified
1.05795091	1.1079888	Quantified
1.12323994	1.18550858	Quantified
0.91244926	0.96700903	Quantified
0.94368241	0.81752155	Quantified
0.99172966	0.99654554	Quantified
1.02430264	0.96064008	Quantified
1.01646599	0.98562568	Quantified
0.91950963	0.88055687	Quantified
0.90663818	0.97905349	Quantified
1.0285873	1.14549055	Quantified

1.02382163	1.04796063	Quantified
1.00570966	1.10072247	Quantified
1.02500985	0.96025921	Quantified
0.93768747	0.89276802	Quantified
1.05153107	1.17045177	Quantified
0.97783894	0.95570213	Quantified
0.8716566	0.8451053	Quantified
0.90384592	0.99511119	Quantified
0.96659532	1.00325541	Quantified
0.97412327	1.1190993	Quantified
0.89189827	0.91232507	Quantified
1.01768099	0.94453899	Quantified
1.10325705	1.04980865	Quantified
0.97151642	1.04091479	Quantified
1.07163813	1.09734325	Quantified
1.04424854	1.12012764	Quantified
1.07426054	0.91232879	Quantified
1.03698782	1.03940571	Quantified
1.10220277	1.10554025	Quantified
0.99792471	0.9146568	Quantified
0.97199334	0.9586423	Quantified
0.96375697	0.94519524	Quantified
0.88870687	0.92440116	Quantified
0.93214447	1.08096468	Quantified
0.92883802	1.18599382	Quantified
1.13722261	1.17090231	Quantified
0.70718938	0.73723745	Quantified
0.88493931	0.95371195	Quantified
0.98134047	1.00485294	Quantified
0.81344034	0.75088417	Quantified
0.92565842	0.90328178	Quantified
1.05754041	1.03834356	Quantified
0.83936266	0.85975473	Quantified
1.01059212	0.99392685	Quantified
0.73689725	0.61361277	Quantified
1.13890365	1.14826651	Quantified
1.13691616	1.32314612	Quantified
0.96269184	0.8849291	Quantified
0.74619358	0.73157139	Quantified
0.98124329	0.96574586	Quantified
1.02564576	0.98042921	Quantified
1.08289863	1.09326732	Quantified
0.98200852	0.97215942	Quantified
1.08452242	1.04017772	Quantified
0.92713626	0.88799316	Quantified
1.05461511	1.03628312	Quantified
1.04511494	1.0284244	Quantified
1.04369389	1.0333836	Quantified
0.97710046	1.01156283	Quantified
1.20811898	1.17678549	Quantified

0.92919137	0.88511397	Quantified
0.89037029	0.8829358	Quantified
0.91572661	0.94146501	Quantified
0.93410734	0.90793249	Quantified
1.01355248	0.98236087	Quantified
0.95204842	0.9430153	Quantified
1.12055774	1.0331592	Quantified
1.12035557	1.12029164	Quantified
0.9271928	0.91224045	Quantified
0.9738867	1.00574092	Quantified
1.02908483	1.05852282	Quantified
1.00408287	0.95135285	Quantified
1.0039795	1.02150169	Quantified
0.88189035	0.96045147	Quantified
0.9353173	0.90930012	Quantified
1.06902223	1.0649761	Quantified
1.23862826	1.11691578	Quantified
0.80360061	0.90357079	Quantified
0.94247871	0.89092818	Quantified
0.96183349	0.9929624	Quantified
1.03759864	0.9853745	Quantified
1.04543053	1.13064503	Quantified
1.03433421	0.99009219	Quantified
0.90817017	0.86047182	Quantified
1.0383337	1.03283175	Quantified
1.00312558	0.95282369	Quantified
0.84084057	0.79523448	Quantified
0.99313309	1.03506484	Quantified
0.86424286	1.1548135	Quantified
0.89774802	0.92676326	Quantified
0.91663845	0.90860788	Quantified
1.07620423	1.14761816	Quantified
1.00404039	0.98599097	Quantified
0.9299509	0.81113276	Quantified
0.89167545	0.89895194	Quantified
0.91302727	0.95637241	Quantified
1.06581336	1.03339652	Quantified
0.99153473	1.0383242	Quantified
1.02687876	1.01285363	Quantified
1.13549054	0.96740667	Quantified
1.05822906	1.05747215	Quantified
1.16491616	1.19459995	Quantified
0.41807426	0.42121151	Quantified
0.99223319	0.95689934	Quantified
1.15870924	1.18368227	Quantified
0.89645278	0.99312147	Quantified
0.93805178	0.93850825	Quantified
0.93234953	0.84266034	Quantified
1.11870227	1.0965557	Quantified
0.98349414	0.97413123	Quantified

1.17148145	1.3257758	Quantified
1.04478715	1.17097229	Quantified
0.94786824	0.95623574	Quantified
0.96750202	0.97996002	Quantified
1.05941195	1.05945237	Quantified
1.00516798	1.05282479	Quantified
0.94287961	1.32190302	Quantified
0.84377949	0.91187023	Quantified
1.08739805	0.92276058	Quantified
0.86856278	0.75668592	Quantified
0.8711153	0.83611852	Quantified
0.8064335	0.72275986	Quantified
0.89276146	0.86083473	Quantified
0.9607481	1.0319521	Quantified
1.00887354	1.05831186	Quantified
0.95857515	0.8101794	Quantified
0.9108921	1.01513029	Quantified
1.04437556	1.05474955	Quantified
1.04859984	1.03771104	Quantified
		Identified
0.8793864	1.36131618	Quantified
0.93040579	0.92013359	Quantified
1.16612454	1.21680595	Quantified
0.93966685	0.91978725	Quantified
1.01739286	1.13380104	Quantified
0.99399207	0.92463231	Quantified
0.91416736	0.99003286	Quantified
0.94428342	0.96664422	Quantified
1.07171185	1.00336264	Quantified
1.01204734	1.00185206	Quantified
1.03367391	1.1132837	Quantified
1.13997124	0.9939915	Quantified
0.98191358	0.96460353	Quantified
0.84274247	0.81876177	Quantified
1.15830242	1.30169359	Quantified
0.89584064	0.7825467	Quantified
0.79472297	2.07363926	Quantified
1.04598268	1.02030883	Quantified
0.94639348	0.98462589	Quantified
0.93837683	1.02731879	Quantified
0.96073481	1.12708407	Quantified
1.03250241	0.99929026	Quantified
0.94240174	0.9920703	Quantified
1.21964846	1.20497023	Quantified
1.09619058	1.04102406	Quantified
0.92299184	1.00908895	Quantified
0.8374948	0.81132257	Quantified
1.12714167	1.11068874	Quantified
0.96046774	0.96239535	Quantified
0.83848741	0.93549891	Quantified

1.00413546	1.07030141	Quantified
0.9101814	0.93802668	Quantified
0.93531854	1.33216867	Quantified
0.85524953	0.81805526	Quantified
1.55188566	1.42410378	Quantified
0.81921432	0.98496505	Quantified
1.19529677	1.11883521	Quantified
1.06681985	1.11646227	Quantified
1.18085498	1.30880394	Quantified
1.0367204	1.06356712	Quantified
0.9171945	0.91250129	Quantified
0.96416126	0.9764915	Quantified
0.99645271	1.01833077	Quantified
0.85469567	0.88233693	Quantified
0.96263911	0.95315838	Quantified
1.03683356	1.23410863	Quantified
1.02396411	0.92827949	Quantified
1.15812713	1.22715662	Quantified
1.00246638	0.98518768	Quantified
0.80090952	0.81394672	Quantified
0.7740339	0.69149043	Quantified
1.05020894	1.06595796	Quantified
0.74815091	0.83553234	Quantified
1.09385652	1.08338817	Quantified
0.90225646	0.81328788	Quantified
1.08961379	1.07162462	Quantified
0.89826395	0.97229557	Quantified
0.73854252	0.8635119	Quantified
0.97679512	0.93380958	Quantified
0.98319802	1.02540275	Quantified
1.00534712	0.91790434	Quantified
0.99939241	0.94177043	Quantified
0.98636257	1.03789433	Quantified
