# Supplementary material for: Antibacterial Activity and Multi-Targeted Mechanism of Action of Suberanilic Acid Isolated from Pestalotiopsis trachycarpicola DCL44: An Endophytic Fungi from Ageratina adenophora
Source: Molecules. 2024 Sep 4;29(17):4205. doi: 10.3390/molecules29174205 (PMC11396930; doi:10.3390/molecules29174205)
Supplement: Supplementary file 1 [file molecules-29-04205-s001.zip › Supporting Information S2-1 List of target peptides and daughter ions for PRM quantification.pdf.pdf]

Peptide	Protein	Replicate	Precursor Mz	Precursor Charge	Product Mz
NADTNAIVSSVTK	Q6GH18	1	660.343706	2	1134.600094
NADTNAIVSSVTK	Q6GH18	1	660.343706	2	733.445431
NADTNAIVSSVTK	Q6GH18	1	660.343706	2	620.361367
ISAGQAGTGAGFQK	Q6GH18	1	646.833308	2	1179.575276
ISAGQAGTGAGFQK	Q6GH18	2	646.833308	2	1179.575276
ISAGQAGTGAGFQK	Q6GH18	1	646.833308	2	836.426093
ISAGQAGTGAGFQK	Q6GH18	2	646.833308	2	836.426093
ISAGQAGTGAGFQK	Q6GH18	1	646.833308	2	607.319837
ISAGQAGTGAGFQK	Q6GH18	2	646.833308	2	607.319837
GDGSSTVAPIVEK	Q6GH18	1	630.327524	2	755.466166
GDGSSTVAPIVEK	Q6GH18	1	630.327524	2	656.397753
GDGSSTVAPIVEK	Q6GH18	1	630.327524	2	585.360639
ALDAGVDSYILK	Q5HEP0	1	632.84281	2	1080.557166
ALDAGVDSYILK	Q5HEP0	1	632.84281	2	965.530223
ALDAGVDSYILK	Q5HEP0	1	632.84281	2	894.49311
GYSNQEIASASHITIK	Q5HEP0	1	573.628447	3	1040.609871
GYSNQEIASASHITIK	Q5HEP0	1	573.628447	3	769.456664
GYSNQEIASASHITIK	Q5HEP0	1	573.628447	3	698.419551
EIYEAPAAEVILK	Q6GIC7	1	723.39795	2	1203.661966
EIYEAPAAEVILK	Q6GIC7	1	723.39795	2	911.556044
EIYEAPAAEVILK	Q6GIC7	1	723.39795	2	840.51893
LFIDSTQQYVSGDVR	Q6GIC7	1	864.433583	2	1051.516699
LFIDSTQQYVSGDVR	Q6GIC7	1	864.433583	2	632.336215
LFIDSTQQYVSGDVR	Q6GIC7	1	864.433583	2	533.267801
DVAHFKPIIEK	Q6GIC7	1	648.869162	2	874.539666
DVAHFKPIIEK	Q6GIC7	1	648.869162	2	727.471252
DVAHFKPIIEK	Q6GIC7	1	648.869162	2	599.376289
GWYLSEYSK	A0A660A2T8	1	566.769114	2	889.430175
GWYLSEYSK	A0A660A2T8	1	566.769114	2	726.366846
GWYLSEYSK	A0A660A2T8	1	566.769114	2	613.282782
QALDEATNDITQNIK	A0A660A2T8	1	837.420673	2	1046.547664
QALDEATNDITQNIK	A0A660A2T8	1	837.420673	2	945.499986
QALDEATNDITQNIK	A0A660A2T8	1	837.420673	2	603.346051
NAPQTLEEVEANAAK	A0A660A2T8	1	792.896834	2	1174.595009
NAPQTLEEVEANAAK	A0A660A2T8	1	792.896834	2	960.463266
NAPQTLEEVEANAAK	A0A660A2T8	1	792.896834	2	831.420673
TITVLVETYK	A6QJ83	1	583.836996	2	952.534974
TITVLVETYK	A6QJ83	1	583.836996	2	752.418882
TITVLVETYK	A6QJ83	1	583.836996	2	411.223811
ETTAIDIPFAAR	Q2FK94	1	652.845884	2	789.425364
ETTAIDIPFAAR	Q2FK94	1	652.845884	2	674.398421
ETTAIDIPFAAR	Q2FK94	1	652.845884	2	561.314357
VAQEAFESWSLTSK	Q2FK94	1	791.89102	2	1155.568065
VAQEAFESWSLTSK	Q2FK94	1	791.89102	2	1084.530952
VAQEAFESWSLTSK	Q2FK94	1	791.89102	2	937.462538
GSESGNAIFNHDGVDK	Q2FK94	1	549.585019	3	931.426821
GSESGNAIFNHDGVDK	Q2FK94	1	549.585019	3	784.358407
GSESGNAIFNHDGVDK	Q2FK94	1	549.585019	3	262.139747
TVEALGLK	A8Z339	1	415.752735	2	630.382102

TVEALGLK	A8Z339	1	415.752735	2	501.339509
TVEALGLK	A8Z339	1	415.752735	2	317.218332
TNSSVVVEDNPAIR	A8Z339	1	750.886269	2	1012.542185
TNSSVVVEDNPAIR	A8Z339	1	750.886269	2	913.473771
TNSSVVVEDNPAIR	A8Z339	1	750.886269	2	814.405357
YWGAQTER	Q6GFK5	1	505.738148	2	661.326378
YWGAQTER	Q6GFK5	1	505.738148	2	604.304915
YWGAQTER	Q6GFK5	1	505.738148	2	405.209223
LGQEISGWR	Q6GFK5	1	523.274898	2	932.458455
LGQEISGWR	Q6GFK5	1	523.274898	2	618.335821
LGQEISGWR	Q6GFK5	1	523.274898	2	505.251757
TIDYNVSLEGALK	A0A2S6D4J7	1	711.877381	2	1208.615744
TIDYNVSLEGALK	A0A2S6D4J7	1	711.877381	2	930.525472
TIDYNVSLEGALK	A0A2S6D4J7	1	711.877381	2	717.414131
DSYIAEIDASDAEK	A0A2S6D4J7	1	763.846475	2	977.442196
DSYIAEIDASDAEK	A0A2S6D4J7	1	763.846475	2	848.399603
DSYIAEIDASDAEK	A0A2S6D4J7	1	763.846475	2	735.315539
SLTITNVAGSTLSR	A0A2S6D4J7	1	710.39373	2	904.48467
SLTITNVAGSTLSR	A0A2S6D4J7	1	710.39373	2	691.373329
SLTITNVAGSTLSR	A0A2S6D4J7	1	710.39373	2	620.336215
VIEISGSELVR	Q6GDG7	2	601.342977	2	989.526201
VIEISGSELVR	Q6GDG7	2	601.342977	2	860.483607
VIEISGSELVR	Q6GDG7	2	601.342977	2	660.367515
LAAESIENPQVR	Q6GDG7	2	663.85424	2	1142.580027
LAAESIENPQVR	Q6GDG7	2	663.85424	2	942.50032
LAAESIENPQVR	Q6GDG7	2	663.85424	2	742.384228
ALFATLSNQELVDK	Q6GDG7	2	774.917038	2	1217.637208
ALFATLSNQELVDK	Q6GDG7	2	774.917038	2	1146.600094
ALFATLSNQELVDK	Q6GDG7	2	774.917038	2	932.468351
LQAQFDAVK	A0A0U1MI5	2	510.279649	2	778.40938
LQAQFDAVK	A0A0U1MI5	2	510.279649	2	579.313689
LQAQFDAVK	A0A0U1MI5	2	510.279649	2	317.218332
TVGLELLEK	P64225	2	501.297507	2	801.471646
TVGLELLEK	P64225	2	501.297507	2	389.239461
TVGLELLEK	P64225	2	501.297507	2	276.155397
ELLVQVR	P64225	2	428.766177	2	614.398421
ELLVQVR	P64225	2	428.766177	2	501.314357
ELLVQVR	P64225	2	428.766177	2	402.245943
VGWFDSVVLR	P65884	2	589.321848	2	1078.568006
VGWFDSVVLR	P65884	2	589.321848	2	835.467229
VGWFDSVVLR	P65884	2	589.321848	2	688.398815
ILDDAFVADEK	P65884	2	618.311343	2	708.356282
ILDDAFVADEK	P65884	2	618.311343	2	462.219454
ILDDAFVADEK	P65884	2	618.311343	2	391.18234
EITEYPANLDQLK	P65884	2	767.393395	2	1291.652858
EITEYPANLDQLK	P65884	2	767.393395	2	1061.562586
EITEYPANLDQLK	P65884	2	767.393395	2	898.499258
FPETSGIGIKPVSK	Q6GG12	2	730.411391	2	728.466501
FPETSGIGIKPVSK	Q6GG12	2	730.411391	2	430.26601
FPETSGIGIKPVSK	Q6GG12	2	730.411391	2	234.144832

AAIQYAIDNNR	Q6GG12	2	624.820201	2	1177.596012
AAIQYAIDNNR	Q6GG12	2	624.820201	2	865.416256
AAIQYAIDNNR	Q6GG12	2	624.820201	2	702.352928
AAIQYAIDNNR	Q6GG12	2	624.820201	2	403.204807
ITDSIEDTIASK	Q6GG12	1	646.832639	2	963.499317
ITDSIEDTIASK	Q6GG12	2	646.832639	2	963.499317
ITDSIEDTIASK	Q6GG12	1	646.832639	2	763.383225
ITDSIEDTIASK	Q6GG12	2	646.832639	2	763.383225
ITDSIEDTIASK	Q6GG12	1	646.832639	2	305.181946
ITDSIEDTIASK	Q6GG12	2	646.832639	2	305.181946
GPLTTPIGGGIR	Q6GG12	2	569.83258	2	871.499592
GPLTTPIGGGIR	Q6GG12	2	569.83258	2	770.451913
GPLTTPIGGGIR	Q6GG12	2	569.83258	2	669.404235
RPNTDELGLEELGVK	Q5HGY8	2	557.296698	3	844.477459
RPNTDELGLEELGVK	Q5HGY8	2	557.296698	3	545.329339
RPNTDELGLEELGVK	Q5HGY8	2	557.296698	3	416.286745
TIEADYVLVTVGR	Q5HGY8	2	718.393198	2	331.208829
TIEADYVLVTVGR	Q5HGY8	2	718.393198	2	232.140415
TIEADYVLVTVGR	Q5HGY8	2	718.393198	2	175.118952
GEAYFVDNNSLR	Q5HGY8	2	692.828223	2	964.48467
GEAYFVDNNSLR	Q5HGY8	2	692.828223	2	817.416256
GEAYFVDNNSLR	Q5HGY8	2	692.828223	2	718.347842
VITPELNGSILPGITR	Q6GJB4	2	840.488162	2	1467.816569
VITPELNGSILPGITR	Q6GJB4	2	840.488162	2	1140.673534
VITPELNGSILPGITR	Q6GJB4	2	840.488162	2	1027.58947
LGYDQVLWLDGVEQK	Q6GJB4	2	881.954152	2	1087.578236
LGYDQVLWLDGVEQK	Q6GJB4	2	881.954152	2	974.494172
LGYDQVLWLDGVEQK	Q6GJB4	2	881.954152	2	788.414859
DGEVALFRPDENFK	Q6GJB4	2	818.901919	2	1052.51597
DGEVALFRPDENFK	Q6GJB4	2	818.901919	2	537.266738
DGEVALFRPDENFK	Q6GJB4	2	818.901919	2	408.224145

Product Charge	Fragment Ion
1	y11
1	y7
1	y6
1	y13
1	y13
1	y9
1	y9
1	y6
1	y6
1	y7
1	y6
1	y5
1	y10
1	y9
1	y8
1	y10
1	y7
1	y6
1	y11
1	y9
1	y8
1	y9
1	y6
1	y5
1	y7
1	y6
1	y5
1	y7
1	y6
1	y5
1	y9
1	y8
1	y5
1	y11
1	y9
1	y8
1	y8
1	y6
1	y3
1	y7
1	y6
1	y5
1	y10
1	y9
1	y8
1	y8
1	y7
1	y2
1	y6

1	y5
1	y3
1	y9
1	y8
1	y7
1	y6
1	y5
1	y3
1	y8
1	y5
1	y4
1	y11
1	y9
1	y7
1	y9
1	y8
1	y7
1	y9
1	y7
1	y6
1	y9
1	y8
1	y6
1	y10
1	y8
1	y6
1	y11
1	y10
1	y8
1	y7
1	y5
1	y3
1	y7
1	y3
1	y2
1	y5
1	y4
1	y3
1	y9
1	y7
1	y6
1	y6
1	y4
1	y3
1	y11
1	y9
1	y8
1	y7
1	y4
1	y2

1	y10
1	y7
1	y6
1	y3
1	y9
1	y9
1	y7
1	y7
1	y3
1	y3
1	y9
1	y8
1	y7
1	y8
1	y5
1	y4
1	y3
1	y2
1	y1
1	y8
1	y7
1	y6
1	y14
1	y11
1	y10
1	y9
1	y8
1	y7
1	y8
1	y4
1	y3
