# Supplementary material for: Antibacterial Activity and Multi-Targeted Mechanism of Action of Suberanilic Acid Isolated from Pestalotiopsis trachycarpicola DCL44: An Endophytic Fungi from Ageratina adenophora
Source: Molecules. 2024 Sep 4;29(17):4205. doi: 10.3390/molecules29174205 (PMC11396930; doi:10.3390/molecules29174205)
Supplement: Supplementary file 1 [file molecules-29-04205-s001.zip › Supporting Information S2-2 Skyline analysis results of PRM data of target peptide segment.pdf.pdf]

| Peptide | Protein | Replicate | Precursor Mz | Precursor Charge | Product Mz |
|---|---|---|---|---|---|
| NADTNAIVSSVTK | Q6GH18 | 1 | 660.343706 | 2 | 1134.600094 |
| NADTNAIVSSVTK | Q6GH18 | 1 | 660.343706 | 2 | 733.445431 |
| NADTNAIVSSVTK | Q6GH18 | 1 | 660.343706 | 2 | 620.361367 |
| ISAGQAGTGAGFQK | Q6GH18 | 1 | 646.833308 | 2 | 1179.575276 |
| ISAGQAGTGAGFQK | Q6GH18 | 2 | 646.833308 | 2 | 1179.575276 |
| ISAGQAGTGAGFQK | Q6GH18 | 1 | 646.833308 | 2 | 836.426093 |
| ISAGQAGTGAGFQK | Q6GH18 | 2 | 646.833308 | 2 | 836.426093 |
| ISAGQAGTGAGFQK | Q6GH18 | 1 | 646.833308 | 2 | 607.319837 |
| ISAGQAGTGAGFQK | Q6GH18 | 2 | 646.833308 | 2 | 607.319837 |
| GDGSSTVAPIVEK | Q6GH18 | 1 | 630.327524 | 2 | 755.466166 |
| GDGSSTVAPIVEK | Q6GH18 | 1 | 630.327524 | 2 | 656.397753 |
| GDGSSTVAPIVEK | Q6GH18 | 1 | 630.327524 | 2 | 585.360639 |
| ALDAGVDSYILK | Q5HEP0 | 1 | 632.84281 | 2 | 1080.557166 |
| ALDAGVDSYILK | Q5HEP0 | 1 | 632.84281 | 2 | 965.530223 |
| ALDAGVDSYILK | Q5HEP0 | 1 | 632.84281 | 2 | 894.49311 |
| GYSNQEIASASHITIK | Q5HEP0 | 1 | 573.628447 | 3 | 1040.609871 |
| GYSNQEIASASHITIK | Q5HEP0 | 1 | 573.628447 | 3 | 769.456664 |
| GYSNQEIASASHITIK | Q5HEP0 | 1 | 573.628447 | 3 | 698.419551 |
| EIYEAPAAEVILK | Q6GIC7 | 1 | 723.39795 | 2 | 1203.661966 |
| EIYEAPAAEVILK | Q6GIC7 | 1 | 723.39795 | 2 | 911.556044 |
| EIYEAPAAEVILK | Q6GIC7 | 1 | 723.39795 | 2 | 840.51893 |
| LFIDSTQQYVSGDVR | Q6GIC7 | 1 | 864.433583 | 2 | 1051.516699 |
| LFIDSTQQYVSGDVR | Q6GIC7 | 1 | 864.433583 | 2 | 632.336215 |
| LFIDSTQQYVSGDVR | Q6GIC7 | 1 | 864.433583 | 2 | 533.267801 |
| DVAHFKPIIEK | Q6GIC7 | 1 | 648.869162 | 2 | 874.539666 |
| DVAHFKPIIEK | Q6GIC7 | 1 | 648.869162 | 2 | 727.471252 |
| DVAHFKPIIEK | Q6GIC7 | 1 | 648.869162 | 2 | 599.376289 |
| GWYLSEYSK | A0A660A2T8 | 1 | 566.769114 | 2 | 889.430175 |
| GWYLSEYSK | A0A660A2T8 | 1 | 566.769114 | 2 | 726.366846 |
| GWYLSEYSK | A0A660A2T8 | 1 | 566.769114 | 2 | 613.282782 |
| QALDEATNDITQNIK | A0A660A2T8 | 1 | 837.420673 | 2 | 1046.547664 |
| QALDEATNDITQNIK | A0A660A2T8 | 1 | 837.420673 | 2 | 945.499986 |
| QALDEATNDITQNIK | A0A660A2T8 | 1 | 837.420673 | 2 | 603.346051 |
| NAPQTLEEVEANAAK | A0A660A2T8 | 1 | 792.896834 | 2 | 1174.595009 |
| NAPQTLEEVEANAAK | A0A660A2T8 | 1 | 792.896834 | 2 | 960.463266 |
| NAPQTLEEVEANAAK | A0A660A2T8 | 1 | 792.896834 | 2 | 831.420673 |
| TITVLVETYK | A6QJ83 | 1 | 583.836996 | 2 | 952.534974 |
| TITVLVETYK | A6QJ83 | 1 | 583.836996 | 2 | 752.418882 |
| TITVLVETYK | A6QJ83 | 1 | 583.836996 | 2 | 411.223811 |
| ETTAIDIPFAAR | Q2FK94 | 1 | 652.845884 | 2 | 789.425364 |
| ETTAIDIPFAAR | Q2FK94 | 1 | 652.845884 | 2 | 674.398421 |
| ETTAIDIPFAAR | Q2FK94 | 1 | 652.845884 | 2 | 561.314357 |
| VAQEAFESWSLTSK | Q2FK94 | 1 | 791.89102 | 2 | 1155.568065 |
| VAQEAFESWSLTSK | Q2FK94 | 1 | 791.89102 | 2 | 1084.530952 |
| VAQEAFESWSLTSK | Q2FK94 | 1 | 791.89102 | 2 | 937.462538 |
| GSESGNAIFNHDGVDK | Q2FK94 | 1 | 549.585019 | 3 | 931.426821 |
| GSESGNAIFNHDGVDK | Q2FK94 | 1 | 549.585019 | 3 | 784.358407 |
| GSESGNAIFNHDGVDK | Q2FK94 | 1 | 549.585019 | 3 | 262.139747 |
| TVEALGLK | A8Z339 | 1 | 415.752735 | 2 | 630.382102 |

| | | | | | |
|---|---|---|---|---|---|
| TVEALGLK | A8Z339 | 1 | 415.752735 | 2 | 501.339509 |
| TVEALGLK | A8Z339 | 1 | 415.752735 | 2 | 317.218332 |
| TNSSVVVEDNPAIR | A8Z339 | 1 | 750.886269 | 2 | 1012.542185 |
| TNSSVVVEDNPAIR | A8Z339 | 1 | 750.886269 | 2 | 913.473771 |
| TNSSVVVEDNPAIR | A8Z339 | 1 | 750.886269 | 2 | 814.405357 |
| YWGAQTER | Q6GFK5 | 1 | 505.738148 | 2 | 661.326378 |
| YWGAQTER | Q6GFK5 | 1 | 505.738148 | 2 | 604.304915 |
| YWGAQTER | Q6GFK5 | 1 | 505.738148 | 2 | 405.209223 |
| LGQEISGWR | Q6GFK5 | 1 | 523.274898 | 2 | 932.458455 |
| LGQEISGWR | Q6GFK5 | 1 | 523.274898 | 2 | 618.335821 |
| LGQEISGWR | Q6GFK5 | 1 | 523.274898 | 2 | 505.251757 |
| TIDYNVSLEGALK | A0A2S6D4J7 | 1 | 711.877381 | 2 | 1208.615744 |
| TIDYNVSLEGALK | A0A2S6D4J7 | 1 | 711.877381 | 2 | 930.525472 |
| TIDYNVSLEGALK | A0A2S6D4J7 | 1 | 711.877381 | 2 | 717.414131 |
| DSYIAEIDASDAEK | A0A2S6D4J7 | 1 | 763.846475 | 2 | 977.442196 |
| DSYIAEIDASDAEK | A0A2S6D4J7 | 1 | 763.846475 | 2 | 848.399603 |
| DSYIAEIDASDAEK | A0A2S6D4J7 | 1 | 763.846475 | 2 | 735.315539 |
| SLTITNVAGSTLSR | A0A2S6D4J7 | 1 | 710.39373 | 2 | 904.48467 |
| SLTITNVAGSTLSR | A0A2S6D4J7 | 1 | 710.39373 | 2 | 691.373329 |
| SLTITNVAGSTLSR | A0A2S6D4J7 | 1 | 710.39373 | 2 | 620.336215 |
| VIEISGSELVR | Q6GDG7 | 2 | 601.342977 | 2 | 989.526201 |
| VIEISGSELVR | Q6GDG7 | 2 | 601.342977 | 2 | 860.483607 |
| VIEISGSELVR | Q6GDG7 | 2 | 601.342977 | 2 | 660.367515 |
| LAAESIENPQVR | Q6GDG7 | 2 | 663.85424 | 2 | 1142.580027 |
| LAAESIENPQVR | Q6GDG7 | 2 | 663.85424 | 2 | 942.50032 |
| LAAESIENPQVR | Q6GDG7 | 2 | 663.85424 | 2 | 742.384228 |
| ALFATLSNQELVDK | Q6GDG7 | 2 | 774.917038 | 2 | 1217.637208 |
| ALFATLSNQELVDK | Q6GDG7 | 2 | 774.917038 | 2 | 1146.600094 |
| ALFATLSNQELVDK | Q6GDG7 | 2 | 774.917038 | 2 | 932.468351 |
| LQAQFDAVK | A0A0U1MI5 | 2 | 510.279649 | 2 | 778.40938 |
| LQAQFDAVK | A0A0U1MI5 | 2 | 510.279649 | 2 | 579.313689 |
| LQAQFDAVK | A0A0U1MI5 | 2 | 510.279649 | 2 | 317.218332 |
| TVGLELLEK | P64225 | 2 | 501.297507 | 2 | 801.471646 |
| TVGLELLEK | P64225 | 2 | 501.297507 | 2 | 389.239461 |
| TVGLELLEK | P64225 | 2 | 501.297507 | 2 | 276.155397 |
| ELLVQVR | P64225 | 2 | 428.766177 | 2 | 614.398421 |
| ELLVQVR | P64225 | 2 | 428.766177 | 2 | 501.314357 |
| ELLVQVR | P64225 | 2 | 428.766177 | 2 | 402.245943 |
| VGWFDSVVLR | P65884 | 2 | 589.321848 | 2 | 1078.568006 |
| VGWFDSVVLR | P65884 | 2 | 589.321848 | 2 | 835.467229 |
| VGWFDSVVLR | P65884 | 2 | 589.321848 | 2 | 688.398815 |
| ILDDAFVADEK | P65884 | 2 | 618.311343 | 2 | 708.356282 |
| ILDDAFVADEK | P65884 | 2 | 618.311343 | 2 | 462.219454 |
| ILDDAFVADEK | P65884 | 2 | 618.311343 | 2 | 391.18234 |
| EITEYPANLDQLK | P65884 | 2 | 767.393395 | 2 | 1291.652858 |
| EITEYPANLDQLK | P65884 | 2 | 767.393395 | 2 | 1061.562586 |
| EITEYPANLDQLK | P65884 | 2 | 767.393395 | 2 | 898.499258 |
| FPETSGIGIKPVSK | Q6GG12 | 2 | 730.411391 | 2 | 728.466501 |
| FPETSGIGIKPVSK | Q6GG12 | 2 | 730.411391 | 2 | 430.26601 |
| FPETSGIGIKPVSK | Q6GG12 | 2 | 730.411391 | 2 | 234.144832 |

| | | | | | |
|---|---|---|---|---|---|
| AAIQYAIDNNR | Q6GG12 | 2 | 624.820201 | 2 | 1177.596012 |
| AAIQYAIDNNR | Q6GG12 | 2 | 624.820201 | 2 | 865.416256 |
| AAIQYAIDNNR | Q6GG12 | 2 | 624.820201 | 2 | 702.352928 |
| AAIQYAIDNNR | Q6GG12 | 2 | 624.820201 | 2 | 403.204807 |
| ITDSIEDTIASK | Q6GG12 | 1 | 646.832639 | 2 | 963.499317 |
| ITDSIEDTIASK | Q6GG12 | 2 | 646.832639 | 2 | 963.499317 |
| ITDSIEDTIASK | Q6GG12 | 1 | 646.832639 | 2 | 763.383225 |
| ITDSIEDTIASK | Q6GG12 | 2 | 646.832639 | 2 | 763.383225 |
| ITDSIEDTIASK | Q6GG12 | 1 | 646.832639 | 2 | 305.181946 |
| ITDSIEDTIASK | Q6GG12 | 2 | 646.832639 | 2 | 305.181946 |
| GPLTTPIGGGIR | Q6GG12 | 2 | 569.83258 | 2 | 871.499592 |
| GPLTTPIGGGIR | Q6GG12 | 2 | 569.83258 | 2 | 770.451913 |
| GPLTTPIGGGIR | Q6GG12 | 2 | 569.83258 | 2 | 669.404235 |
| RPNTDELGLEELGVK | Q5HGY8 | 2 | 557.296698 | 3 | 844.477459 |
| RPNTDELGLEELGVK | Q5HGY8 | 2 | 557.296698 | 3 | 545.329339 |
| RPNTDELGLEELGVK | Q5HGY8 | 2 | 557.296698 | 3 | 416.286745 |
| TIEADYVLVTVGR | Q5HGY8 | 2 | 718.393198 | 2 | 331.208829 |
| TIEADYVLVTVGR | Q5HGY8 | 2 | 718.393198 | 2 | 232.140415 |
| TIEADYVLVTVGR | Q5HGY8 | 2 | 718.393198 | 2 | 175.118952 |
| GEAYFVDNNSLR | Q5HGY8 | 2 | 692.828223 | 2 | 964.48467 |
| GEAYFVDNNSLR | Q5HGY8 | 2 | 692.828223 | 2 | 817.416256 |
| GEAYFVDNNSLR | Q5HGY8 | 2 | 692.828223 | 2 | 718.347842 |
| VITPELNGSILPGITR | Q6GJB4 | 2 | 840.488162 | 2 | 1467.816569 |
| VITPELNGSILPGITR | Q6GJB4 | 2 | 840.488162 | 2 | 1140.673534 |
| VITPELNGSILPGITR | Q6GJB4 | 2 | 840.488162 | 2 | 1027.58947 |
| LGYDQVLWLDGVEQK | Q6GJB4 | 2 | 881.954152 | 2 | 1087.578236 |
| LGYDQVLWLDGVEQK | Q6GJB4 | 2 | 881.954152 | 2 | 974.494172 |
| LGYDQVLWLDGVEQK | Q6GJB4 | 2 | 881.954152 | 2 | 788.414859 |
| DGEVALFRPDENFK | Q6GJB4 | 2 | 818.901919 | 2 | 1052.51597 |
| DGEVALFRPDENFK | Q6GJB4 | 2 | 818.901919 | 2 | 537.266738 |
| DGEVALFRPDENFK | Q6GJB4 | 2 | 818.901919 | 2 | 408.224145 |

| Product Charge | Fragment Ion | Retention Time | Area |
|---|---|---|---|
| 1 | y11 | 19.1 | 9138554 |
| 1 | y7 | 19.1 | 10741362 |
| 1 | y6 | 19.1 | 15703128 |
| 1 | y13 | 10.87 | 1784700 |
| 1 | y13 | 51.6 | 0 |
| 1 | y9 | 10.87 | 7798468 |
| 1 | y9 | 52.25 | 35294 |
| 1 | y6 | 10.87 | 3486958 |
| 1 | y6 | 52.03 | 1633574 |
| 1 | y7 | 13.89 | 7609655 |
| 1 | y6 | 13.89 | 14323408 |
| 1 | y5 | 13.89 | 14087077 |
| 1 | y10 | 31.3 | 34994356 |
| 1 | y9 | 31.3 | 17388726 |
| 1 | y8 | 31.3 | 28214074 |
| 1 | y10 | 20.25 | 5352267 |
| 1 | y7 | 20.25 | 3392400 |
| 1 | y6 | 20.25 | 4149955 |
| 1 | y11 | 34.84 | 20264050 |
| 1 | y9 | 34.74 | 41892456 |
| 1 | y8 | 34.84 | 1.03E+08 |
| 1 | y9 | 30.64 | 1012654 |
| 1 | y6 | 30.64 | 1064312 |
| 1 | y5 | 30.64 | 1824788 |
| 1 | y7 | 17.51 | 3190039 |
| 1 | y6 | 17.51 | 1865382 |
| 1 | y5 | 17.51 | 3864873 |
| 1 | y7 | 21.78 | 2997070 |
| 1 | y6 | 21.78 | 2196000 |
| 1 | y5 | 21.78 | 17865678 |
| 1 | y9 | 25.64 | 454381 |
| 1 | y8 | 25.64 | 197533 |
| 1 | y5 | 25.64 | 441266 |
| 1 | y11 | 23.14 | 5674179 |
| 1 | y9 | 23.14 | 4532367 |
| 1 | y8 | 23.14 | 2362714 |
| 1 | y8 | 28.14 | 1.62E+08 |
| 1 | y6 | 28.14 | 1.23E+08 |
| 1 | y3 | 28.14 | 97494928 |
| 1 | y7 | 34.7 | 6.57E+08 |
| 1 | y6 | 34.7 | 4.85E+08 |
| 1 | y5 | 34.7 | 9.07E+08 |
| 1 | y10 | 31.99 | 89508168 |
| 1 | y9 | 31.99 | 1.08E+08 |
| 1 | y8 | 31.99 | 75632296 |
| 1 | y8 | 13.03 | 64708676 |
| 1 | y7 | 13.03 | 27082298 |
| 1 | y2 | 13.03 | 56342984 |
| 1 | y6 | 16.16 | 4.59E+08 |

| | | | |
|---|---|---|---|
| 1 | y5 | 16.16 | 1.35E+08 |
| 1 | y3 | 16.16 | 2.7E+08 |
| 1 | y9 | 17.2 | 46079536 |
| 1 | y8 | 17.2 | 38801164 |
| 1 | y7 | 17.2 | 23169406 |
| 1 | y6 | 10.74 | 1.48E+08 |
| 1 | y5 | 10.63 | 11555373 |
| 1 | y3 | 10.63 | 8769355 |
| 1 | y8 | 19.07 | 2.06E+08 |
| 1 | y5 | 19.07 | 2.68E+08 |
| 1 | y4 | 19.07 | 4.27E+08 |
| 1 | y11 | 32.62 | 85469760 |
| 1 | y9 | 32.72 | 39531856 |
| 1 | y7 | 32.72 | 80967856 |
| 1 | y9 | 24.28 | 7400366 |
| 1 | y8 | 24.28 | 5579480 |
| 1 | y7 | 24.28 | 6556217 |
| 1 | y9 | 27.93 | 58176080 |
| 1 | y7 | 27.93 | 1.5E+08 |
| 1 | y6 | 27.93 | 63470264 |
| 1 | y9 | 26.58 | 5.11E+08 |
| 1 | y8 | 26.58 | 2.93E+08 |
| 1 | y6 | 26.58 | 2.12E+08 |
| 1 | y10 | 15.67 | 85469424 |
| 1 | y8 | 15.67 | 1.86E+08 |
| 1 | y6 | 15.67 | 1.98E+08 |
| 1 | y11 | 34.3 | 45392440 |
| 1 | y10 | 34.3 | 68952960 |
| 1 | y8 | 34.3 | 1.19E+08 |
| 1 | y7 | 15.39 | 1.39E+09 |
| 1 | y5 | 15.39 | 5.37E+08 |
| 1 | y3 | 15.39 | 7.24E+08 |
| 1 | y7 | 30.82 | 1.84E+08 |
| 1 | y3 | 30.82 | 42432100 |
| 1 | y2 | 30.82 | 36818344 |
| 1 | y5 | 19.47 | 3.26E+08 |
| 1 | y4 | 19.47 | 4.08E+08 |
| 1 | y3 | 19.47 | 4.15E+08 |
| 1 | y9 | 43.94 | 8293468 |
| 1 | y7 | 43.94 | 20149044 |
| 1 | y6 | 43.86 | 17612956 |
| 1 | y6 | 24.43 | 9596523 |
| 1 | y4 | 24.43 | 21413884 |
| 1 | y3 | 24.43 | 11101584 |
| 1 | y11 | 28.48 | 1382186 |
| 1 | y9 | 28.48 | 2677513 |
| 1 | y8 | 28.48 | 11342127 |
| 1 | y7 | 20.53 | 11206843 |
| 1 | y4 | 20.53 | 24487390 |
| 1 | y2 | 20.53 | 13285683 |

| | | | |
|---|---|---|---|
| 1 | y10 | 15.78 | 992935 |
| 1 | y7 | 15.78 | 3.69E+08 |
| 1 | y6 | 15.78 | 3.34E+08 |
| 1 | y3 | 15.78 | 2.41E+08 |
| 1 | y9 | 20.35 | 34590372 |
| 1 | y9 | 20.07 | 23425846 |
| 1 | y7 | 20.35 | 64279300 |
| 1 | y7 | 20.07 | 44816196 |
| 1 | y3 | 20.35 | 63906024 |
| 1 | y3 | 20.07 | 37758152 |
| 1 | y9 | 22.92 | 5.09E+08 |
| 1 | y8 | 22.92 | 4.61E+08 |
| 1 | y7 | 22.92 | 1.51E+09 |
| 1 | y8 | 28.31 | 3.67E+08 |
| 1 | y5 | 28.31 | 1.38E+08 |
| 1 | y4 | 28.31 | 1.47E+08 |
| 1 | y3 | 37.27 | 1E+08 |
| 1 | y2 | 37.27 | 2.03E+08 |
| 1 | y1 | 37.27 | 1.33E+08 |
| 1 | y8 | 21.26 | 3.38E+08 |
| 1 | y7 | 21.26 | 3.09E+08 |
| 1 | y6 | 21.26 | 2.73E+08 |
| 1 | y14 | 43.32 | 4610241 |
| 1 | y11 | 43.32 | 13436543 |
| 1 | y10 | 43.32 | 26080342 |
| 1 | y9 | 44.27 | 9948375 |
| 1 | y8 | 44.27 | 9407750 |
| 1 | y7 | 44.27 | 6179992 |
| 1 | y8 | 29.64 | 923791 |
| 1 | y4 | 29.85 | 622610 |
| 1 | y3 | 29.71 | 363167 |
