# Supplementary material for: Antibacterial Activity and Multi-Targeted Mechanism of Action of Suberanilic Acid Isolated from Pestalotiopsis trachycarpicola DCL44: An Endophytic Fungi from Ageratina adenophora
Source: Molecules. 2024 Sep 4;29(17):4205. doi: 10.3390/molecules29174205 (PMC11396930; doi:10.3390/molecules29174205)
Supplement: Supplementary file 1 [file molecules-29-04205-s001.zip › Supporting Information S2-3 Example of secondary mass spectrum matching of candidate peptide of target protein (9).pdf]

YAS09280051-1-PRE1 - GYSNQEIASASHITIK, Charge 3

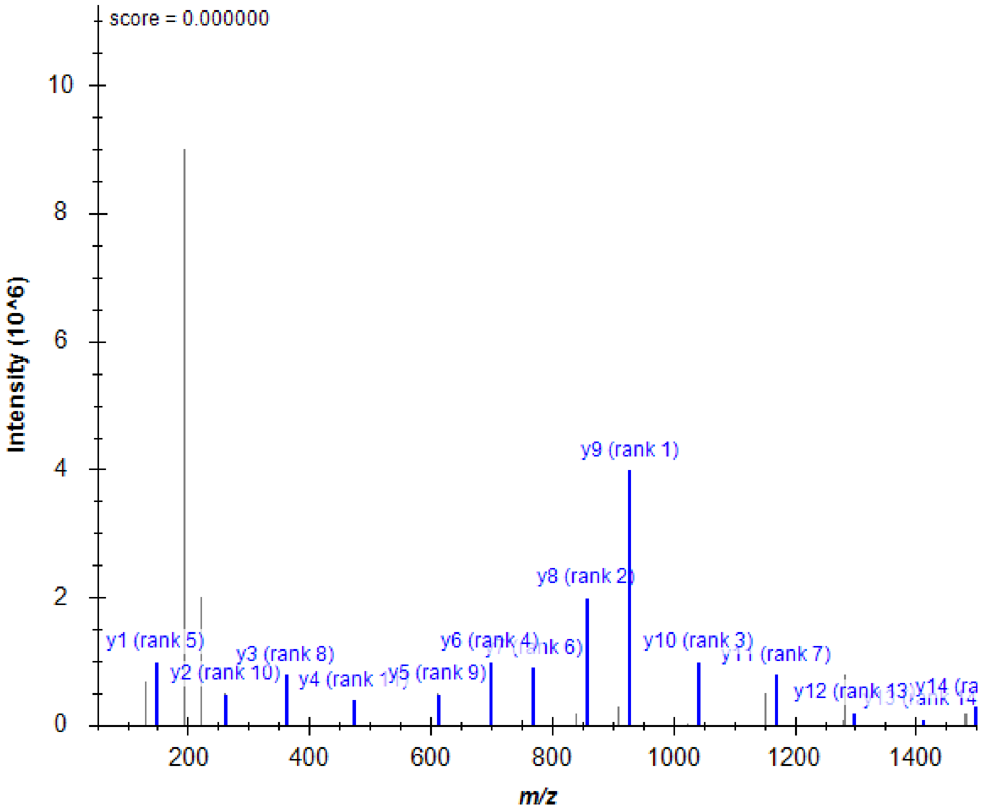

YAS09280051-1-PRE1 - EIYEAPAAEVILK, Charge 2

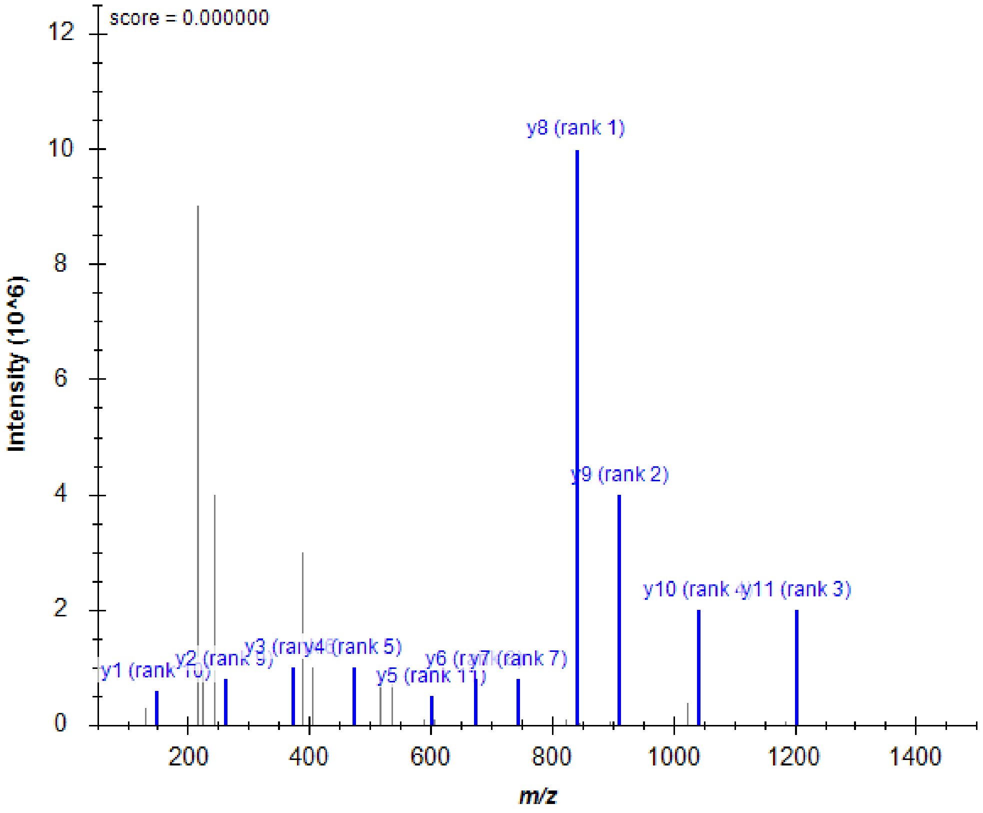

YAS09280051-1-PRE1 - VAQEAFESWSLTSK, Charge 2

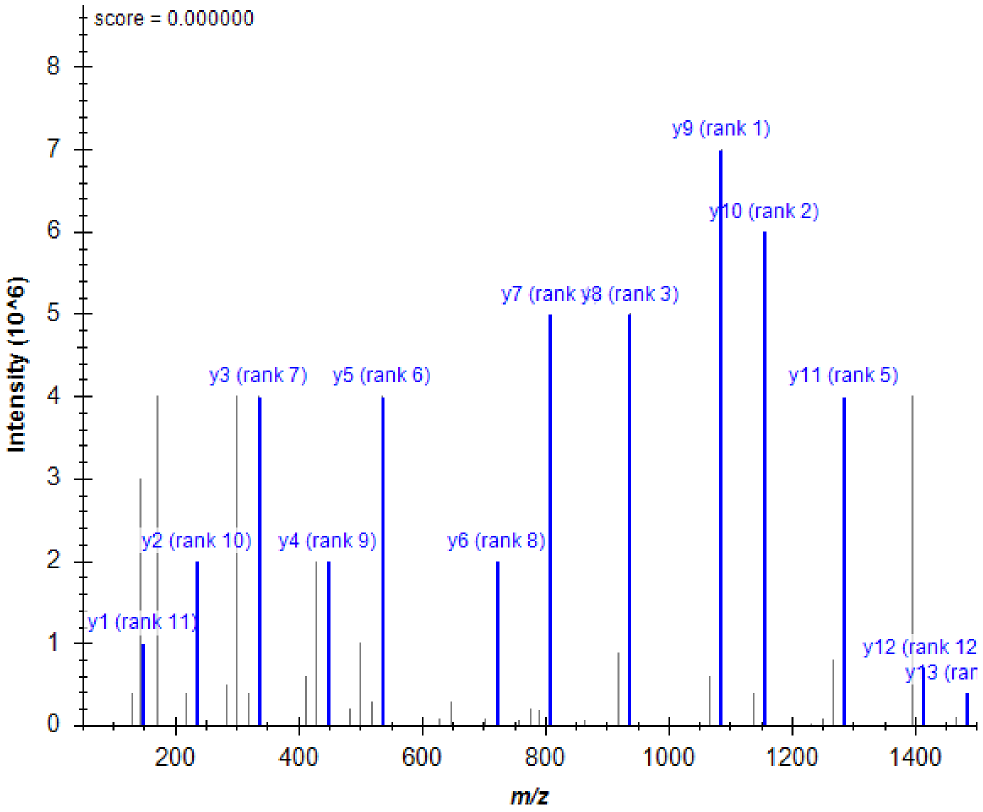

YAS09280051-1-PRE1 - ETTAIDIPFAAR, Charge 2

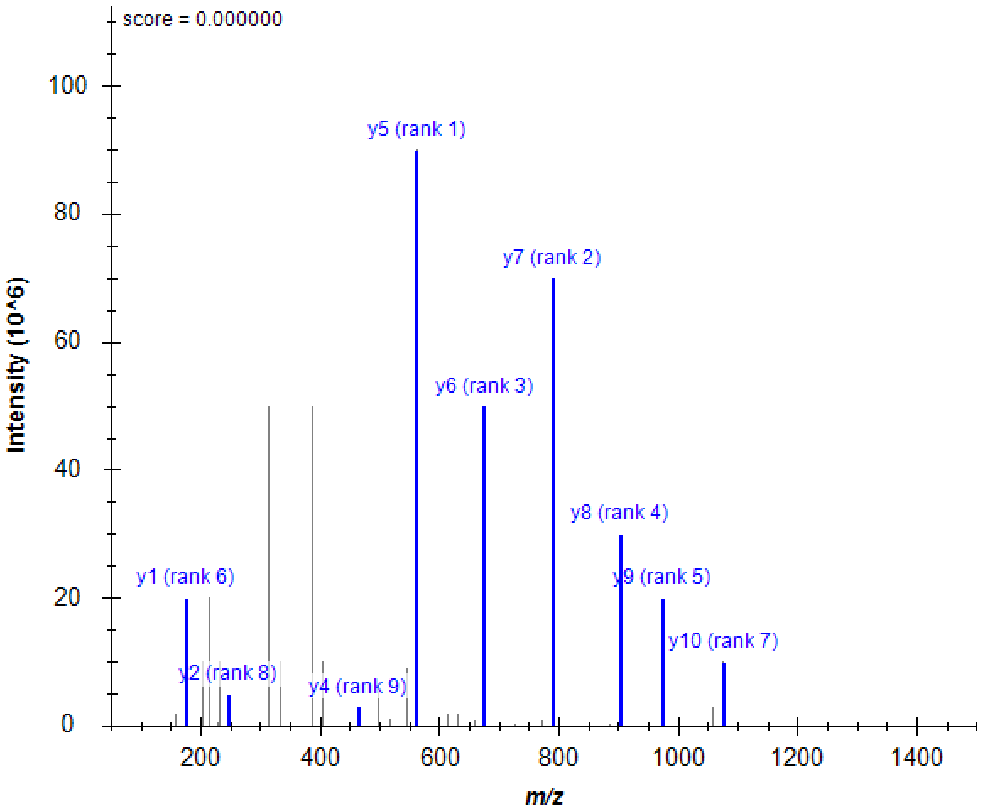

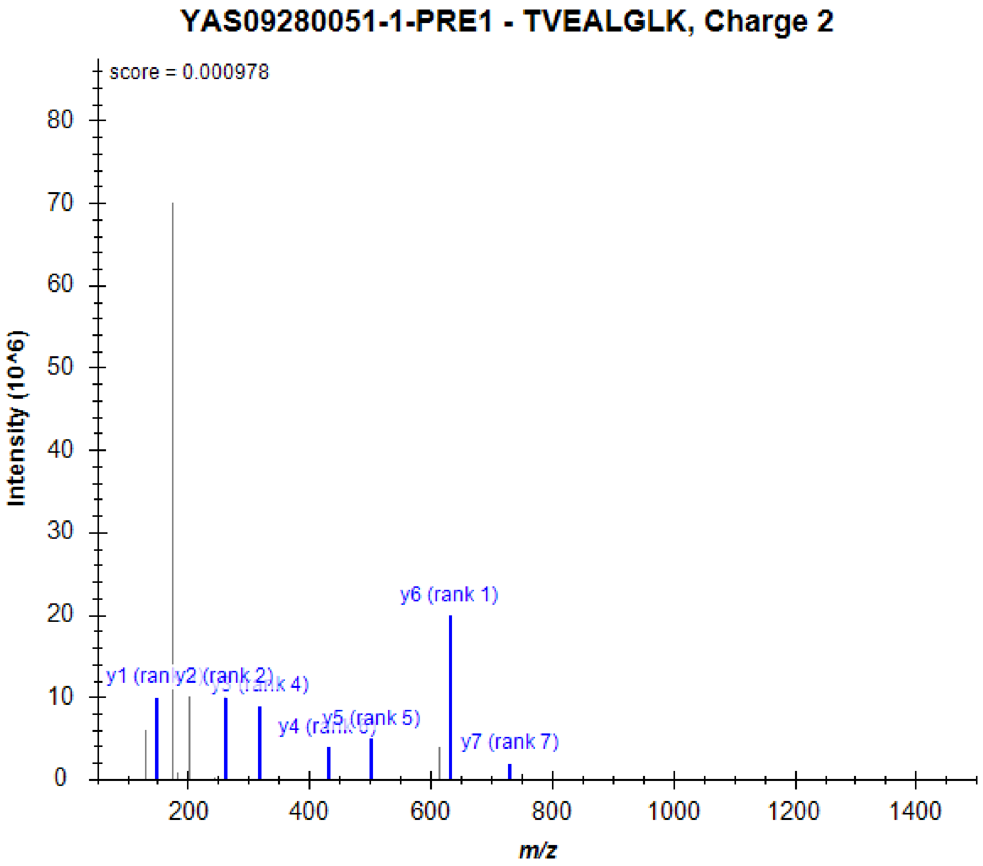

YAS09280051-1-PRE1 - TVEALGLK, Charge 2
score = 0.000978
Intensity (10^6)
m/z
y1 (ran
y2 (rank 2)
y6 (rank 1)
y5 (rank 5)
y7 (rank 7)
