# Supplementary material for: Antibacterial Activity and Multi-Targeted Mechanism of Action of Suberanilic Acid Isolated from Pestalotiopsis trachycarpicola DCL44: An Endophytic Fungi from Ageratina adenophora
Source: Molecules. 2024 Sep 4;29(17):4205. doi: 10.3390/molecules29174205 (PMC11396930; doi:10.3390/molecules29174205)

1.蛋白名称：Q5HEP0

肽段序列：GYSNQEIASASHITIK

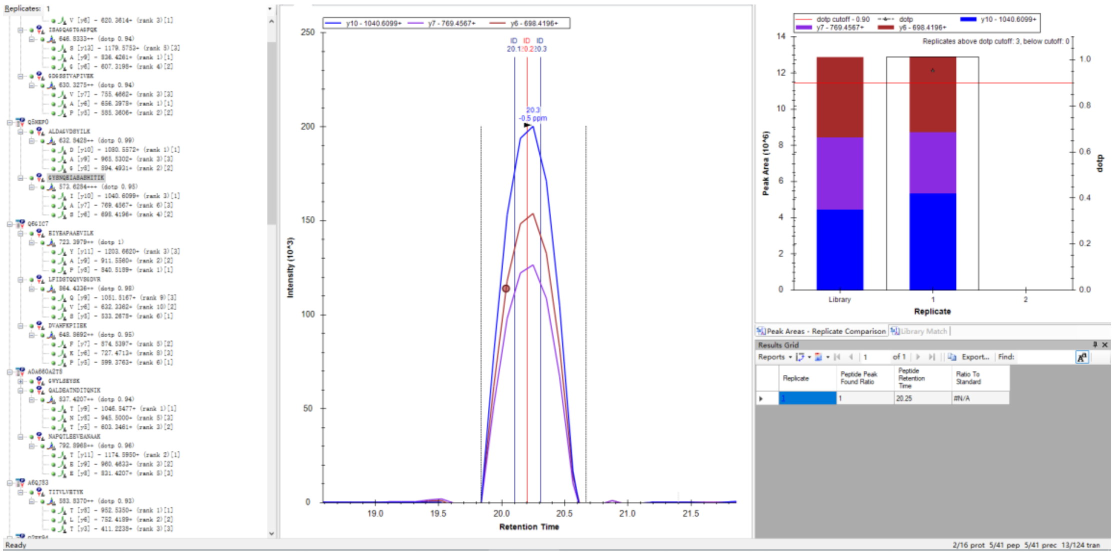

2.蛋白名称：Q6GIC7

肽段序列：EIYEAPAAEVILK

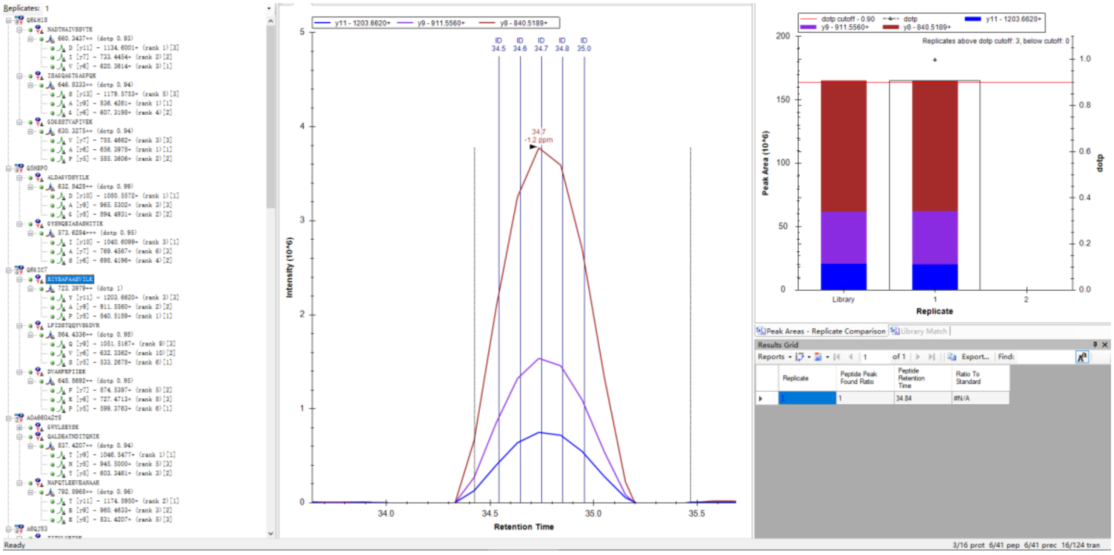

3.蛋白名称：Q2FK94

肽段序列：VAQEAFESWSLTSK

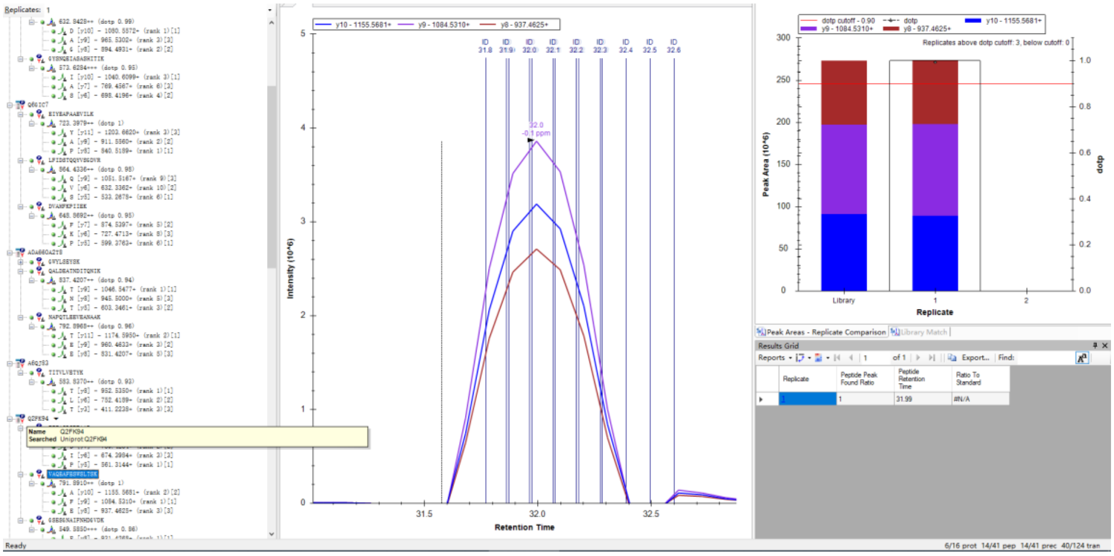

4.蛋白名称：Q2FK94

肽段序列：ETTAIDIPFAAR

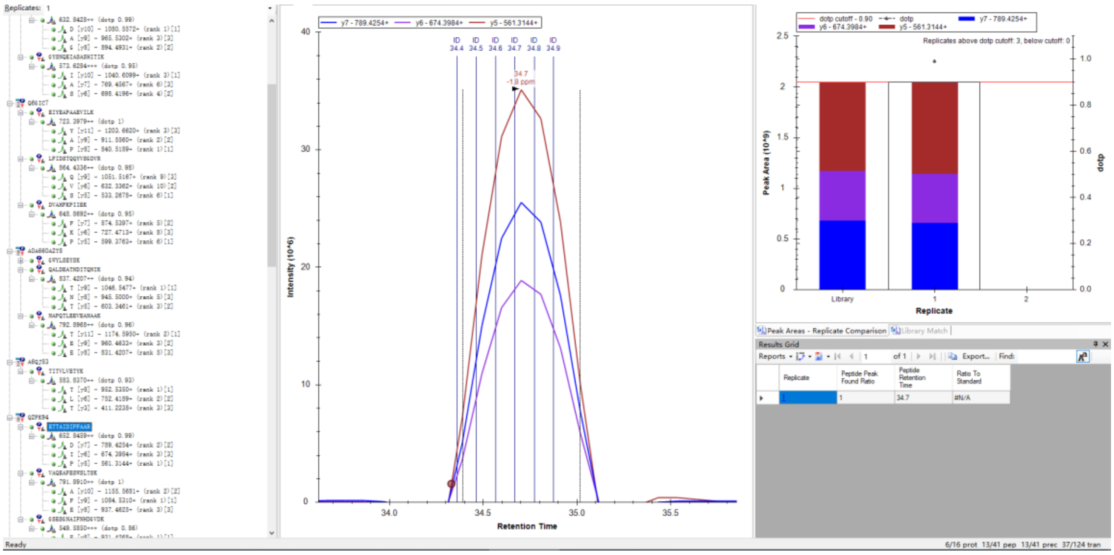

5.蛋白名称：A8Z339

肽段序列：TVEALGLK

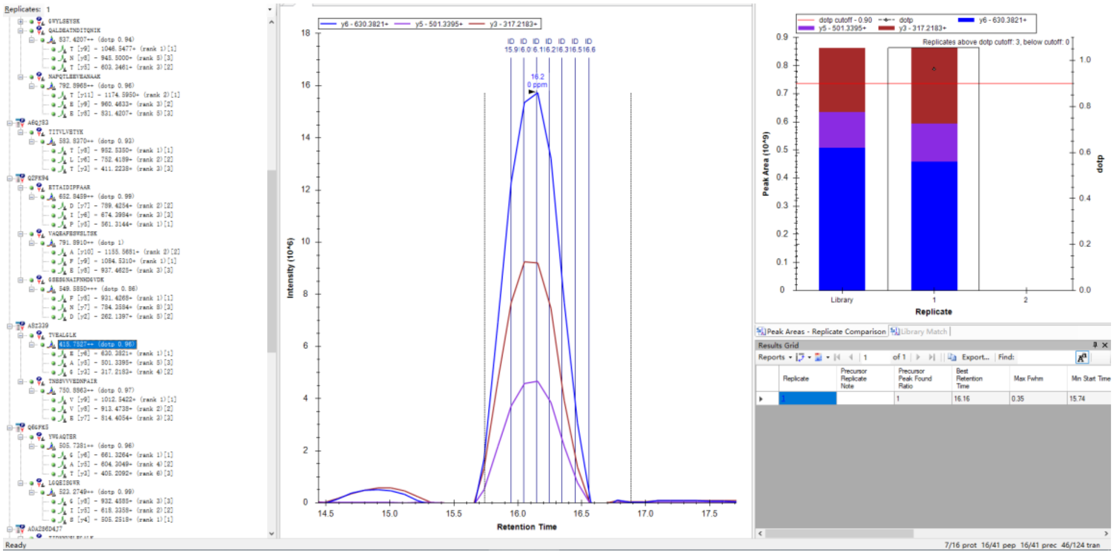

Supplement: Supplementary file 1 [file molecules-29-04205-s001.zip › Supporting Information S2-4 Example of Skyline analysis of PRM results of target peptide segment.pdf]
